# Therapeutic effects of total saikosaponins from *Radix bupleuri* against Alzheimer’s disease

**DOI:** 10.3389/fphar.2022.940999

**Published:** 2022-07-21

**Authors:** Juan Li, Bin Zou, Xiao-Yu Cheng, Xin-He Yang, Jia Li, Chun-Hui Zhao, Rui-Xia Ma, Ji-Xiang Tian, Yao Yao

**Affiliations:** ^1^ School of Pharmacy, Ningxia Medical University, Yinchuan, China; ^2^ Ningxia Engineering and Technology Research Center for Modernization of Characteristic Chinese Medicine, and Key Laboratory of Ningxia Ethnomedicine Modernization, Ministry of Education, Ningxia Medical University, Yinchuan, China; ^3^ Department of Neurology and Clinical Research Center of Neurological Disease, The Second Affiliated Hospital of Soochow University, Suzhou, China; ^4^ Institute of Chinese Materia Medica, China Academy of Chinese Medical Sciences, Beijing, China; ^5^ School of Basic Medical Sciences, Ningxia Medical University, Yinchuan, China

**Keywords:** total saikosaponins, Alzheimer’s disease, Aβ, p-tau, Nrf2, autophagy, gut microbiota

## Abstract

Alzheimer’s disease (AD) is a neurodegenerative disease characterized by memory loss and cognitive dysfunction in the elderly, with amyloid-beta (Aβ) deposition and hyperphosphorylation of tau protein as the main pathological feature. Nuclear factor 2 (Nrf2) is a transcription factor that primarily exists in the cytosol of hippocampal neurons, and it is considered as an important regulator of autophagy, oxidative stress, and inflammation. Total saikosaponins (TS) is the main bioactive component of *Radix bupleuri* (Chaihu). In this study, it was found that TS could ameliorate cognitive dysfunction in APP/PS1 transgenic mice and reduce Aβ generation and senile plaque deposition via activating Nrf2 and downregulating the expression of β-secretase 1 (BACE1). In addition, TS can enhance autophagy by promoting the expression of Beclin-1 and LC3-II, increasing the degradation of p62 and NDP52 and the clearance of phosphorylated tau (*p-*tau), and reducing the expression of *p-*tau. It can also downregulate the expression of nuclear factor-κB (NF-κB) to inhibit the activation of glial cells and reduce the release of inflammatory factors. *In vitro* experiments using PC12 cells induced by Aβ, TS could significantly inhibit the aggregation of Aβ and reduce cytotoxicity. It was found that Nrf2 knock-out weakened the inhibitory effect of TS on BACE1 and NF-κB transcription in PC12 cells. Moreover, the inhibitory effect of TS on BACE1 transcription was achieved by promoting the binding of Nrf2 and the promoter of BACE1 ARE1. Results showed that TS downregulated the expression of BACE1 and NF-κB through Nrf2, thereby reducing the generation of Aβ and inhibiting neuroinflammation. Furthermore, TS can ameliorate synaptic loss and alleviate oxidative stress. In gut microbiota analysis, dysbiosis was demonstrated in APP/PS1 transgenic mice, indicating a potential link between gut microbiota and AD. Furthermore, TS treatment reverses the gut microbiota disorder in APP/PS1 mice, suggesting a therapeutic strategy by remodeling the gut microbe. Collectively, these data shows that TS may serve as a potential approach for AD treatment. Further investigation is needed to clarify the detailed mechanisms underlying TS regulating gut microbiota and oxidative stress.

## 1 Introduction

Alzheimer’s disease (AD) is a progressive and multifaceted neurodegenerative disorder of the central nervous system with dementia, loss of memory, and cognitive disturbance (Luo et al., 2016). At present, cholinesterase inhibitors (ChEIs) and N-methyl-d-aspartate receptor antagonists such as donepezil, rivastin, galantamine, and memantine have been approved for clinical treatment of AD, which are single-target drugs that can only show mild and temporary improvement in learning and memory dysfunction accompanied by hepatotoxicity or cholinergic crisis, indicating that improving cognitive function through a single target is not a feasible therapeutic approach (Yang et al., 2017; Liu et al., 2018; Osama et al., 2020; Scheltens Philip, 2021). Therefore, developing multi-target, low-toxicity, and effective drugs for the treatment of AD is necessary.

The neuropathological features of AD include the deposition of Aβ plaques in the neocortex and neurofibrillary tangles (primarily composed of tau aggregates) in the marginal and cortical joint areas (Busche & Hyman, 2020). Genetic studies have shown that mutations in amyloid precursor protein (APP) or enzymes that produce Aβ can cause autosomal dominant hereditary AD; thus, Aβ is considered as the key initiator of the disease. The Aβ cascade hypothesis indicates that Aβ can induce a series of harmful responses, including increased inflammation, synaptic dysfunction, and neuronal loss, and promote tau protein phosphorylation (Tsai et al., 2015; Pereira et al., 2019; Busche & Hyman, 2020; Zetterberg & Bendlin, 2021). The hyperphosphorylation of tau protein (a microtubule-associated protein) is related to the tangles of nerve fibers, which can also aggravate the inflammation of the nervous system, affect the mitochondrial function of neurons, and promote the decline of cognitive function (Tsai et al., 2015; Wang Z. Y. et al, 2016). Recently, the therapeutic effect of Nrf2 on AD has been reported: Nrf2 is highly expressed in astrocytes, providing a neuroprotective effect (Cuadrado et al., 2018; Osama et al., 2020). In NRF2-deficient mice, the levels of insoluble *p-*tau and Aβ increased significantly, which aggravated cognitive impairment in APP/PS1 mice (Branca et al., 2017; Rojo et al., 2017; Osama et al., 2020). In addition, considerable literature has shown that NRF2 promotes the clearance of APP and tau by upregulating the expression of autophagy genes, which plays an important role in maintaining cellular redox dynamic balance and regulating neuroinflammation (Pajares et al., 2016; Pajares et al., 2018; Osama et al., 2020). Therefore, Nrf2 plays a central role in the pathological process of AD.

Recently, several studies have focused on elucidating the two-way communication pathway between intestinal bacteria and the central nervous system: microbe–gut–brain axis. Clinical and laboratory studies have revealed the changes in intestinal flora and its metabolites related to the occurrence and development of AD (Wang Z. Y. et al, 2016; Cryan et al., 2020), and some studies have proven that abnormal microbiota may cause AD. Based on previous reports, the level of trimethylamine N-oxide produced by dietary choline metabolism is increased in patients with dementia, which is positively correlated with *p-*tau, a biomarker of AD (Vogt et al., 2018). In addition, intestinal microbiota-bile acid (BA) can significantly decrease the concentration of primary bile acid (CA) in patients with AD, which is closely related to cognitive decline (MahmoudianDehkordi et al., 2019). Furthermore, scientists have transplanted feces of AD animals into aseptic APP transgenic mice. The accumulation of Aβ is accelerated after transplantation, demonstrating that intestinal microflora contributes to the development of AD pathology. Considering the vital role of intestinal flora in AD, more studies have focused on the intervention of AD based on microbiota. For example, *Clostridium butyricum* (CB) could improve cognitive impairment and Aβ deposition in APP/PS1 mice, reduce neuroinflammation mediated by microglia, and reverse the abnormal changes of gut microbiota (GM) and butyric acid, indicating that CB can play the role of anti-AD by regulating the GM–gut–brain axis (Sun et al., 2020). Therefore, the therapy based on intestinal microbiota may provide a new direction for the treatment of AD (Harach et al., 2017).

*Radix bupleuri* (Chaihu) is a traditional Chinese medicine widely used to treat fever, influenza, inflammation, chronic hepatitis, cancer, nephrotic syndrome and other diseases (Park et al., 2015; Lin et al., 2016). It was recorded that Chaihu show ability to nourish liver and blood, promote blood circulation and Qi flow in the brain (Liang et al., 2012), thus it could be used to treat brain disease. Many traditional prescriptions with Chaihu as the main drug such as Chaihu Shugan San and Xiaochaihu Tang have been used in AD treatment and reported to show potent anti-AD effects (Zhao et al., 2012; Liu et al., 2019a; Zeng et al., 2019; Sohn et al., 2021). Saikosaponins, a group of oleanane triterpenoid saponins, are the main bioactive component in Chaihu. More than 100 different saikosaponins have been identified, of which the main components are saikosaponin a and d. Saikosaponins show various bioactivities such as anticancer, antiviral, antipyretic, hepatoprotective, neuroprotective, immunomodulatory, and antibacterial effects (Park et al., 2015; Lee et al., 2016; Lin et al., 2016; Kim, 2018; Li et al., 2020). Recent studies have shown that saikosaponins have multiple effects on inflammation, antioxidant balance, and injury: SSd can inhibit the production of reactive oxygen species (ROS) and upregulate the expression of antioxidant enzymes, namely, superoxide dismutase (SOD) and malondialdehyde (MDA); SSa and SSd can also inhibit the expression of inflammatory factors in mouse macrophages induced by LPS, indicating that saikosaponins may be used to treat AD through antioxidation and inhibition of neuroinflammation (Kim, 2018). At present, few studies have been conducted on the use of saikosaponins in the treatment of AD. Lee et al. have shown that SSc can inhibit the secretion of Aβ_1-40_ and Aβ_1-42_ and the phosphorylation of tau and promote nerve growth factor-mediated axonal growth (Lee et al., 2016; Kim, 2018). Based on these evidence, total saikosaponins (TS) may have a therapeutic effect in AD treatment, but the effect and mechanism need to be further elucidated.

The present study aimed to investigate the therapeutic effect of TS on APP/PS1 transgenic mice and underlying mechanisms. The results reveal that TS downregulated the transcription and expression level of BACE1 through the Nrf2 pathway to reduce Aβ production and senile plaque deposition. The inhibitory effect of TS on BACE1 transcription was achieved by promoting the binding of Nrf2 and the promoter of BACE1 ARE1. In addition, TS promoted autophagy by regulating the expression of autophagic proteins, thereby reducing the level of *p-*tau. In the aspect of neuroinflammation, TS could downregulate NF-κB transcription and expression through Nrf2, thus inhibiting the activation of glial cells and reducing the production of inflammatory factors. TS was found to ameliorate synaptic loss and alleviate oxidative stress. Furthermore, TS could reverse the GM disorder in APP/PS1 mice and improve the diversity of GM. Collectively, this study can provide a new insight and potential approach for the treatment of AD.

## 2 Materials and methods

### 2.1 Preparation of total saikosaponins

*Radix Bupleuri* was purchased from the Chinese Medicinal Material market in Bozhou, China (Lot No. 20190311). Total saikosaponins were prepared using a modified method based on previous report (Li et al., 2013). Briefly, 3 kg air-dried roots were sliced and reflux-extracted by 80% ethanol (adjusted to pH 9 with KOH) for two times, 90 min each. The extract solution was concentrated under vacuum to 3 L, and then subjected to column chromatography using 4.5 L AB-8 macroporous absorption resin. It was eluted with water, 30% ethanol, 70% ethanol and 95% ethanol successively. The 70% ethanol elution was collected and evaporated under vacuum, and then dried using vacuum freeze dryer to yield TS. Three main monomeric saponins in TS were determined using HPLC, and the content of SSa, SSd, SSc was 17.11, 12.52, 3.36%, respectively.

### 2.2 Animal experiment procedure

Eight-month-old APP/PS1 mice and wild-type (WT) C57BL/6J mice (half male and half female, weighing 25 ± 5 g) were purchased from Beijing Huafukang Experimental Animal Technology Co., Ltd. All animal procedures were performed in accordance with the Provision and General Recommendation of Chinese Experimental Animals Administration Legislation and approved by the Ethic Committee of Ningxia Medical University (No. 2016–037). Throughout the study, all mice were kept in a pathogen-free environment with a light/dark cycle of 12 h and free access to food and water under constant temperature and humidity (22 ± 1°C and 45–55%, respectively). APP/PS1 mice were randomly divided into five groups (n = 10): model group; low, middle, and high-dose TS groups; and donepezil group (positive control). C57BL/6J WT mice were used as blank control. The mice of TS groups were intragastrically administrated with different doses of TS (20, 40, and 80 mg/kg), and the positive group was given donepezil (5 mg/kg). The blank group and model group were given the same amount of normal saline as control. The administration lasted for 30 days, and behavioral tests including Y maze test, novel object recognition (NOR) test, and Morris water maze test were performed on days 22–30. Then, mice were sacrificed by cervical dislocation on day 31, and samples were collected for further assessment. 

### 2.3 Behavioral experiments

**FIGURE 1:**
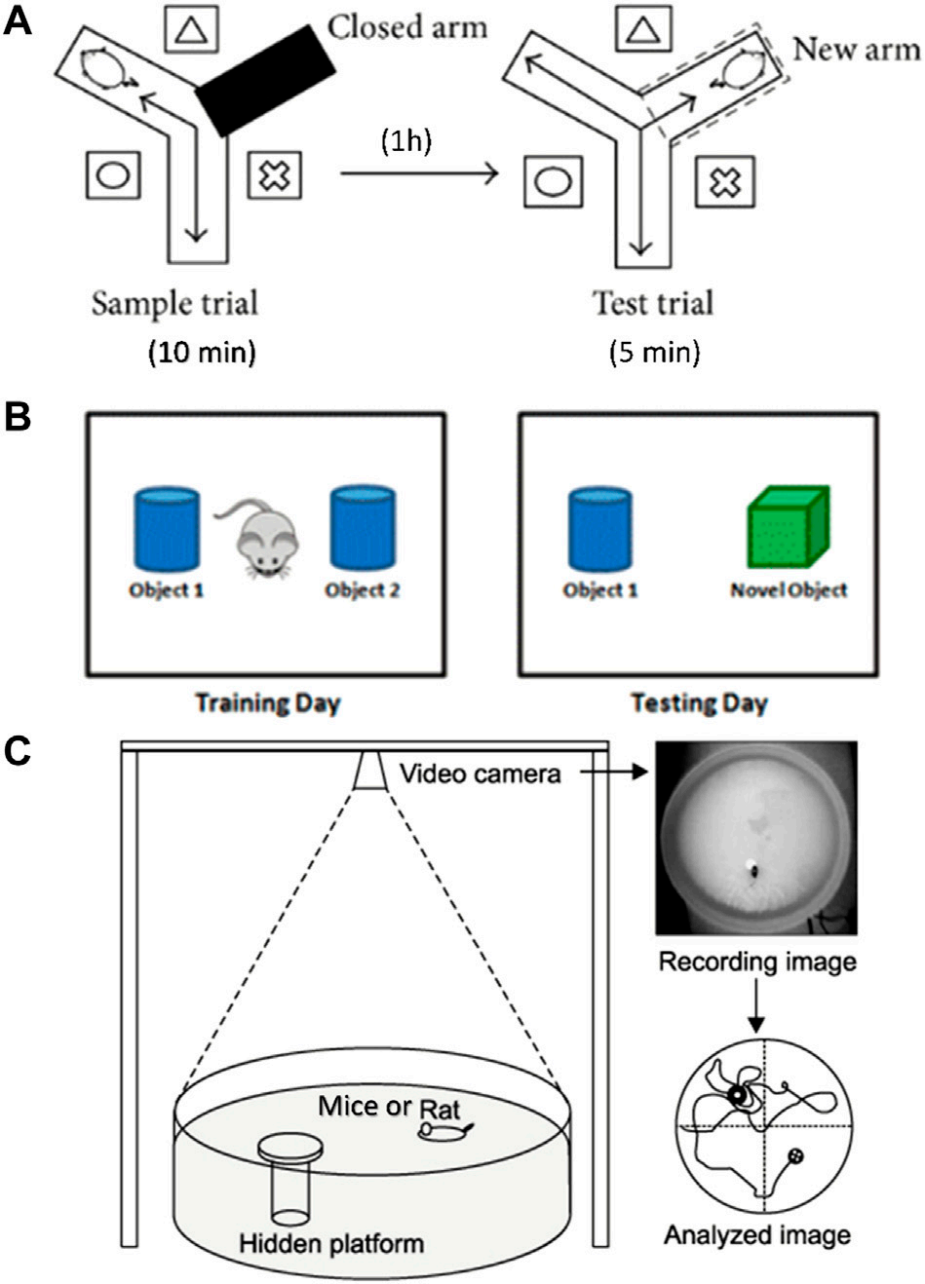
Diagram of behavioral experimental equipment. **(A)** Schematic diagram of Y-maze experimental device. **(B)** Schematic diagram of NOR experiment. **(C)** Schematic diagram of Morris water maze device.

One month after drug treatment, Y-maze test was performed to evaluate the working memory ability as previously described (Liu et al., 2019b). The Y maze consists of three identical arms, each with an angle of 120° and a size of 30 cm × 8 cm × 15 cm (length × width × height). A movable partition was placed in the center, and different geometric figures were affixed to each arm of the maze as a visual marker. The three arms of the Y maze were randomly set as the novel arm, the start arm, and the other arm. A camera was placed 1.5 m above the maze to record the movement track of the mice. The diagram of the Y maze is shown in Figure 1A. The Y maze experiment consists of two stages with an interval of 1 h. The first stage is the training period. After blocking the new arm with a partition, the mice were placed into the starting arm to move freely for 10 min in the maze. After 1 h, during the second stage of detection, a new different arm septum was opened, and similarly, the mice were placed into the starting arm to move freely for 5 min in the three arms. Before each trial, the maze was thoroughly cleaned with 75% ethanol to eliminate scent cues. The residence time of mice in the new arm was recorded.

The NOR test was conducted to evaluate the ability to distinguish new object as previously reported (Scott et al., 2017). The schematic diagram of the NOR test is shown in Figure 1B. During habituation, each mouse was allowed to explore freely for 5 min in the test box (50 cm × 50 cm × 30 cm). During training, which started 24 h later, two identical objects were placed in the test box. Each mouse was placed in the box and allowed to explore freely for 5 min, and the touching times of mice to each object were recorded. After 24 h, one of the original objects was replaced by a new object similar in size but different in shape and color. Each mouse was placed in the test box and allowed to explore freely for 5 min. The touching times of mice to each object were recorded. After each test, the test box and objects were cleaned with 75% ethanol. The times of exploring new and old objects were statistically analyzed, and the discrimination index (DI) was calculated. DI = (TN−TF)/(TN+TF), where TN is the number of times to explore new objects, and TF is the number of times to explore old objects.

Morris water maze test was carried out to evaluate spatial learning and memory ability as previously described (Liu et al., 2019). In brief, the test was carried out in a round stainless-steel pool with a diameter of 1.2 m, which was divided into four quadrants. A 10 cm diameter hidden platform was placed at the center of the IV quadrant, 1 cm below the water surface. The water temperature was kept at 22–24°C. The schematic diagram of the water maze system is shown in Figure 1C. The experiment lasted for 5 days, and each mouse was trained two times a day. During the place navigation test, mice were released into the water facing the pool wall from the edge of each quadrant and allowed to swim freely for 60 s until they found the hidden platform. If a mouse could not find the platform within 60 s, then it will be placed onto the platform and stayed for 30 s. Mice were trained two times a day for 3 days. Twenty-4 hours after the training period, the place navigation test was carried out. The time each mouse spent to find the hidden platform was recorded as escape latency. Afterward, the spatial probe test was performed, in which the platform was removed, and the mice were released into the water to swim freely for 60 s. The following data were recorded: 1) latency, the time mouse spent to reach the hidden platform area for the first time; 2) number of crossing, the number of times the mouse passed through the hidden platform area; 3) time in the target quadrant, the time mouse spent in the quadrant of the former hidden platform. All experimental data were analyzed using the WMT-100S analysis system (Techman Software, Chengdu, China).

### 2.4 Brain tissue preparation

After the behavioral test, the mice were anesthetized and perfused with PBS containing heparin (10 U/mL) precooled at 4°C. The brain was removed and divided into two parts along the sagittal plane. The left hemisphere was fixed with 4% paraformaldehyde, and paraffin-embedded sections were prepared for immunohistochemical experiments. The right hemisphere was stored at −80°C for further ELISA and Western blot experiments.

### 2.5 Immunohistochemical staining

The brain tissue soaked in 4% paraformaldehyde (Solebo) was trimmed, dehydrated, waxed, and then embedded. The repaired wax block was cut into a cross-section with a thickness of 4 μm and then dewaxed and rehydrated after baking. Antigen repair was performed with 0.01 m sodium citrate solution (pH 6.0). Endogenous peroxidase was blocked with 3% hydrogen peroxide solution (Solebo) and then blocked with 3%BSA for 30 min. The slices were then incubated overnight at 4°C with antibodies *p-*tau (Ser396, 1:200, Affinity, AF3148), Iba-1 (1:200, GeneTex, GTX101495), GFAP (1:200, CST, 3670), Synaptophysin (1:200, Abcam, ab32127), PSD-95 (1:200, Abcam, ab12093), and NeuN (1:200, Abcam, ab177487). On the second day, after washing with PBS three times, the slices were incubated with HRP-sheep and rabbit anti-IgG (1:200, Abbkine, A21020), HRP-sheep anti-mouse IgG (1:200, Beijing Zhongshan Biological Engineering Co., Ltd.), Alexa Fluor 488-donkey anti-rabbit IgG secondary antibody (1:200, Abcam, ab150073), and Alexa Fluor 594-donkey anti-sheep IgG (1:200, Abcam, ab150132), followed by coloration with DAB (Beijing Zhongshan Jinqiao Biotechnology Co., Ltd.), and finally dehydrated. The second anti-rabbit IgG antibody was incubated with the second antibody of donkey anti-rabbit IgG (1:200, Beijing Zhongshan Bio-Engineering Co., Ltd.), followed by coloration by DAB (Beijing Zhongshan Jinqiao Biotechnology Co., Ltd.), and then sealed using a neutral gum (Beijing Zhongshan Jinqiao Biotechnology Co., Ltd.). All sections were observed by using an optical microscope (DP73-ST-SET, Olympus, Japan) or sealed with an anti-fluorescence quenching agent.

### 2.6 Quantification of cerebral Aβ levels

Aβ40 and Aβ42 are markers of amyloid deposition in the cerebral cortex of patients with AD (Olsson et al., 2016). The levels of Aβ in mouse brain were determined using Aβ40 and Aβ42 Elisa kits (China Peptide Biochemistry Co., Ltd. and IBL Co., Ltd.) according to the instructions of the manufacturer. The sample to be tested was properly diluted with dilution buffer and coated in an ELISA plate at 100 μL per hole at 4°C overnight, and the coating solution was discarded the next day and washed nine times for 60 s each time. In addition, the labeled antibody working solution, 100 μL per well, was incubated at 4°C for 1 h, and the plate was washed nine times for 60 s each time. Afterward, TMB substrate chromogenic solution was added, 100 μL per well, and light reaction was avoided for 20 min at 37°C. Finally, absorbance was determined at 450 nm after termination.

### 2.7 Measurement of oxidative stress and inflammatory cytokines

TNF-α, IL-6, and IL-1β in the samples were evaluated using ELISA kit according to the manufacturer’s protocol (Biolegend), and oxidative stress was assessed by using MDA, SOD, and GSH kits according to the manufacturer’s protocol (Shanghai Biyuntian Biotechnology Co., Ltd.).

### 2.8 Cell culture

PC12 cells from the Library of Chinese Academy of Sciences cells were incubated in DMEM (Basal Media, J210908) containing 10% fetal bovine serum (Gibco, 10099141C) and 1% penicillin/streptomycin (Hyclone) at 37°C. The cells were cultured in a Petri dish or cell culture plate to 70–80% fusion.

### 2.9 Cell viability assay

The cells were seeded in a 96-well plate (5 × 10^4^ cells/well) and treated with different concentrations of TS (0.01, 0.1, 0.1, and 10 μg/ml), 1 μM of Aβ, 10 μM of CQ (MCE), or 0.1–3.2 μM of Rapa (MCE) for 24 h. Then, the cells were incubated with 5 mg/ml of MTT (50 μL/well) for 4 h. The supernatant was removed, and 150 μL of DMSO was added. The absorbance was measured at 570 nm using a microplate reader. The absorbance values of each group were normalized to the ratio in the control group.

### 2.10 Thioflavin T fluorescence assay

Thiosulfonate T (ThT) was used for immunofluorescence assay of amyloid fibers *in vitro*. In addition, 10 μM of Aβ42 was mixed with 0.2 μg/ml of TS and incubated at 37°C. Then, 180 μL of ThT (5 μM) and 20 μL of samples were mixed in a 96-well plate. The fluorescence value of ThT in the solution was detected at different time points using a fluorescence microplate reader (excitation wavelength of 448 nm and emission wavelength of 485 nm).

### 2.11 Small ribonucleic acid interference

PC12 cells were cultured in a six-well cell culture plate (3 × 10^5^ cells/well). The liposome transfection reagent (LipoFiterTM3, HB-TRLF3) and Nrf2-siRNA (Santa Cruz, sc-155128) were mixed at 1:1 and added to the serum-free DMEM. After incubation in the six-well plate for 6 h, total RNA was extracted and incubated with fresh DMEM containing serum for 36 h. Then, RT-qPCR was performed as described in Section 2.13.

### 2.12 Chromatin immunoprecipitation

The ChIP was performed using ChIP Assay kit (Beyotime Biotechnology, P2078) according to the manufacturer’s protocol. Briefly, PC12 cells treated with TS were cross-linked for 10 min in 10 ml of 1% formaldehyde solution at room temperature and then quenched for 5 min with glycine solution. Cells were washed two times with cold PBS containing PMSF. Then, the cells were scraped, collected, and centrifuged at 4°C and 1000 g for 2 min, and the supernatant was removed. The precipitate was re-suspended with SDS lysis buffer containing PMSF and incubated on ice for 10 min for full cleavage. Chromatin fragments were treated with SONICS (1 min, 5 s on/5 s off) ultrasound. Then, the ultrasonically treated sample was centrifuged at 4°C and 12000 g for 5 min. The supernatant was placed in an ice bath, and the ChIP dilution buffer containing 1 mM PMSF in 1.8 ml was added to dilute the sample to a 2 ml final volume. A 50 μL sample solution was used as Input for follow-up detection. The rest of the sample solution was mixed with 70 μL Protein A+G Agarose/Salmon Sperm DNA and rotated slowly at 4°C for 30 min to reduce the non-specific binding to the target protein or target DNA sequence. Then, the sample was centrifuged at 4°C and 1000 g for 1 min, and the supernatant was transferred to a new centrifuge tube. Subsequently, 1.43 μL of Nrf2 antibody was added and rotated slowly at 4°C to mix overnight. Afterward, 60 μL of Protein A+G Agarose/Salmon Sperm DNA was added and mixed slowly at 4°C for 60 min to precipitate the protein or the corresponding complex recognized by the primary antibody. Then, the solution was centrifuged at 4°C and 1000 g for 1 min. The supernatant was removed carefully, and low-salt immune complex wash buffer, high-salt immune complex wash buffer, and LiCl immune complex wash buffer were used to wash the precipitate once; TE buffer was used to wash the precipitate two times. Then, the solution was centrifuged for 1 min at 4°C and 1,000 g, and the supernatant was removed carefully. The freshly prepared 250 μL of elution buffer was added, vortexed, mixed well, rotated at room temperature, and continuously eluted for 5 min. Afterward, the solution was centrifuged for 1 min at 1,000 *g*, and the supernatant was removed and combined, obtaining a total of 500 μL of supernatant. Twenty microliters of 5M NaCl was added to 500 μL of supernatant and 2.5 μL of 15M NaCl to obtain a 50 μL input and then heated at 65°C for 4 h to remove the cross-linking between protein and genomic DNA. The 520 μL sample was purified by DNA. Forty microliters of TE was used to re-suspend DNA precipitates for qPCR detection of the target gene.

### 2.13 Quantitative real-time polymerase chain reaction

After homogenization of PC12 cells, total RNA was extracted using a total RNA extraction reagent (RNAsimple Total RNA Kit, TIANGEN, DP419) and homogenized in accordance with the regulations of manufacturers, and Promega deoxyribonuclease I (Promega) was used to remove pollution. Five hundred nanogram of each sample were used for first-strand cDNA synthesis (RevertAid First-strand cDNA Synthesis Kit, Thermo, K1622). Then, CFX connect (Biorad) were used for real-time RT-PCR. PCR amplification was performed with SYBR PreMix Ex TaqTMII (Takara). The amplification conditions of all genes were as follows: amplification at 95°C for 30 s, 40 times amplification at 95°C, amplification for 5s, and amplification for 30 s at 60°C. The differences between the Ct values for experimental and reference genes were calculated as ΔΔCt. The qRT-PCR primer sequences are listed in Table 1.

**TABLE 1:** qPCR primer information.

Primer name	Sequence (Forward)	Sequence (Reverse)
BACE1	GTC​CTT​CCG​CAT​CAC​CAT​CCT​TC	ACT​GTG​AGA​CGG​CGA​ACT​TGT​AAC
BACE1 ARE1	ACA​GGT​TCA​GAT​GGG​AGA​AGA​CC	AGG​AGT​AGG​GAT​TTT​GGA​GGG​AC
NF-κB	GGA​TGG​CTT​CTA​TGA​GGC​TGA​ACT​C	CTT​GCT​CCA​GGT​CTC​GCT​TCT​TC

### 2.14 Western blot analysis

Tissue and cell lysates were obtained using a whole protein extraction kit (Key GEN, Nanjing, China). The protein concentration in the supernatant was determined by bicinchoninic acid assay (Key GEN, Nanjing, China). Then, the proteins were separated by SDS-polyacrylamide gel electrophoresis and electro-transferred to polyvinylidene fluoride. After blocking with 5% skimmed milk powder, the film was incubated at 4°C overnight with different primary antibodies: Nrf2 (1:1,000, Proteintech, 16396-1-AP), BACE1 (1:1,000, Proteintech, 12807-1-AP), *p-*tau (Ser 396, 1:500, Affinity, AF3148), NDP52/CALCOCO2 (1:1,000, Proteintech, 12229-1-AP), p62 (1:200, Santa Cruz, sc-48402), LC3 (1:200, Santa Cruz, sc-398822), Beclin-1 (1:200, Santa Cruz, sc-48341), mTOR (1:200, Santa Cruz, sc-517464), *p*-TFEB (1:1,000, Affinity, AF3708) and β-actin (1:1,000, Affinity, AF7018). The membrane was washed in TBST (0.5% Tween-20) three times, incubated with anti-rabbit or anti-mouse IgG second antibody (1:20,000, Abbkine, A21010) at room temperature for 1 h, and developed using an enhanced chemiluminescence kit (Key GEN, Nanjing, China). Imprinting was captured using an image analyzer (Amersham Imager 600, General Electric Company, United States), and the relative intensity of the bands was quantified by ImageJ software.

### 2.15 Fecal sample collection and deoxyribonucleic acid extraction

After intragastrical administration for 4 weeks, mouse fecal samples were collected in a clean cage. Uncontaminated feces were collected and placed in a sterile and enzyme-free EP tube, immediately stored in dry ice, and then frozen at −80°C. A Total DNA was isolated using the MagPure Soil DNA LQ Kit (D6356-03, Personal Biotechnology Co., Ltd., Shanghai, China). The NanoDrop 2000ultraviolet spectrophotometer was used to measure the quality of the DNA. The purified samples were stored at −20°C for further analysis.

### 2.16 16S rRNA gene high-throughput sequencing and sequencing data analysis

16S rRNA can be used as the characteristic nucleic acid sequence of species, and it is considered as the most suitable index for bacterial phylogenetic and taxonomic identification. In this experiment, bacterial 16S rRNA at the V3-V4 region was amplified using gene universal primers: 338F (5′-ACT​CCT​ACG​GGA​GGC​AGC​A-3′) and 806R (5′-GGACTACHVGGGTWTCTAAT-3′). The amplification products were recovered by 2% agarose gel electrophoresis method and quantified by Quant-iT PicoGreen dsDNA Assay Kit. The sequencing libraries were constructed on the Illumina Nova Seq system (Personal Biotechnology Co., Ltd., Shanghai, China).

### 2.17 Analysis of gut microbiota

ASVs (amplicon sequence variants) were clustered and dereplicated by DADA2, and the similarity was 100% (Callahan et al., 2016) (Personal Biotechnology Co., Ltd., Shanghai, China). Compared with Greengenes database (Release13.8, http://greengenes.secondgenome.com/), ASVs were annotated with a taxonomic identifier. QIIME2 (2019.4) software was used to show the specific composition of each group at different species taxonomic levels. Chao1 index and Observed species index were used as indicators to measure Alpha diversity according to species richness. R programming language was used to count the number of ASVs in each group according to the grouping of samples, and bray-curtis clustering algorithm and average clustering method was used to calculate the distance matrices of each sample to evaluate the similarity between samples. The clustering results of each sample were calculated and presented in the form of heat map. Finally, the importance index of marker species was analyzed by Random Forest algorithm. The random forest analysis and nested hierarchical cross test were used in QIIME2 and https://www.genescloud.cn/home online software.

### 2.18 Statistical analysis

All the experiments were repeated three times, and all the data in each experiment were represented as means ± SEM and processed using GraphPad Prism (version 8, GraphPad Software Inc., CA, United States). Using one-way ANOVA or two-way ANOVA with Dunnett’s multiple comparison post hoc test to compare the differences among the groups. For comparisons between two groups, the significance of difference between means was determined by Student’s t-test. A *p* value of <0.05 was considered as statistical significance.

## 3 Results

### 3.1 Total saikosaponins treatment alleviates cognitive impairment in APP/PS1 mice

**FIGURE 2:**
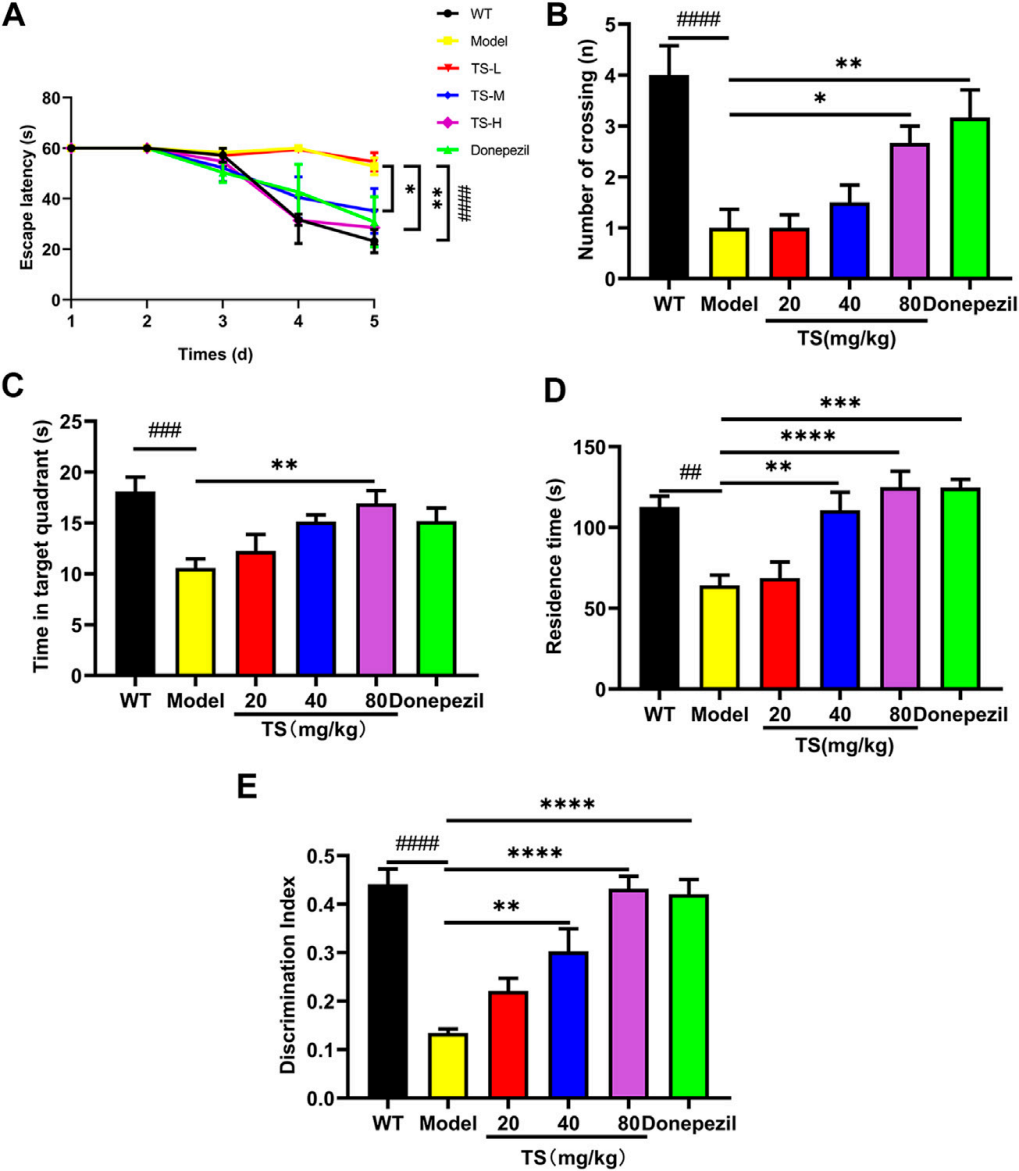
TS ameliorated cognitive impairment in APP/PS1 mice. **(A)** The effect of TS on the escape latency in APP/PS1 mice was investigated by Morris water maze (^####^ *p <* 0.0001, ^*^ *p =* 0.0277, ^**^ *p* = 0.0010). **(B)** The effect of TS on the number of crossing platform in APP/PS1 mice was investigated by Morris water maze (^####^ *p <* 0.0001, ^*^ *p =* 0.0353, ^**^ *p =* 0.0044). **(C)** The effect of TS on the time spent in the target quadrant in APP/PS1 mice by Morris water maze (^###^ *p =* 0.0007, ^**^ *p* = 0.0042). **(D)** The effect of TS on the residence time in APP/PS1 mice was evaluated by Y-maze test (^##^ *p* = 0.0015, ^**^ *p* = 0.0024, ^***^ *p =* 0.001, ^****^ *p <* 0.0001). **(E)** The effect of TS on the discrimination index in APP/PS1 mice was investigated by NOR test (^####^ *p <* 0.0001, ^**^ *p* = 0.0021, ^****^ *p <* 0.0001). ^#^ Compared with WT group; ^*^ Compared with model group. Data are presented as mean ± S.E.M. (*n* = 10).

The effect of TS on spatial memory ability impairment in APP/PS1 mice was evaluated by MWM test (Figures 2A–C). Compared with WT mice, the escape latency of APP/PS1 mice in the place navigation test was significantly increased (Figure 2A, *p <* 0.0001), and the number of crossing platform and the time spent in the target quadrant were significantly decreased (Figure 2B, *p <* 0.0001; Figure 2C, *p <* 0.001), indicating that the spatial memory ability of APP/PS1 mice was seriously impaired. Compared with APP/PS1 mice, the escape latency in the middle and high-dose TS treatment group was remarkably shortened (Figure 2A, *p* < 0.05 and *p <* 0.01 respectively), and the number of crossing platform and time spent in the target quadrant were significantly increased after high-dose TS treatment (Figure 2B, *p <* 0.05; Figure 2C, *p <* 0.01), indicating that 80 mg/kg TS treatment could significantly improve the spatial memory impairment of APP/PS1 mice.

The effect of TS on spatial working memory ability impairment in APP/PS1 mice was evaluated by Y-maze test. APP/PS1 mice showed less residence time in the new arm than WT mice (Figure 2D, *p <* 0.01), whereas middle and high-dose TS treatment significantly increased the residence time (Figure 2D, *p <* 0.01 and *p <* 0.0001, respectively), indicating that TS could effectively improve the spatial working memory ability of APP/PS1 mice.

The effect of TS on the NOR ability impairment in APP/PS1 mice was evaluated by NOR test. The results showed that compared with WT mice, the DI of APP/PS1 mice was significantly lower than that of WT mice (Figure 2E, *p <* 0.0001). After middle and high-dose TS treatment, the DI significantly increased, indicating that TS could improve NOR ability in APP/PS1 mice (Figure 2E, *p <* 0.01 and *p <* 0.0001 respectively). Collectively, these data indicated that TS treatment could significantly alleviate cognitive impairment in APP/PS1 mice.

### 3.2 Total saikosaponins treatment reduces Aβ level and senile plaque in APP/PS1 mice brain

**FIGURE 3:**
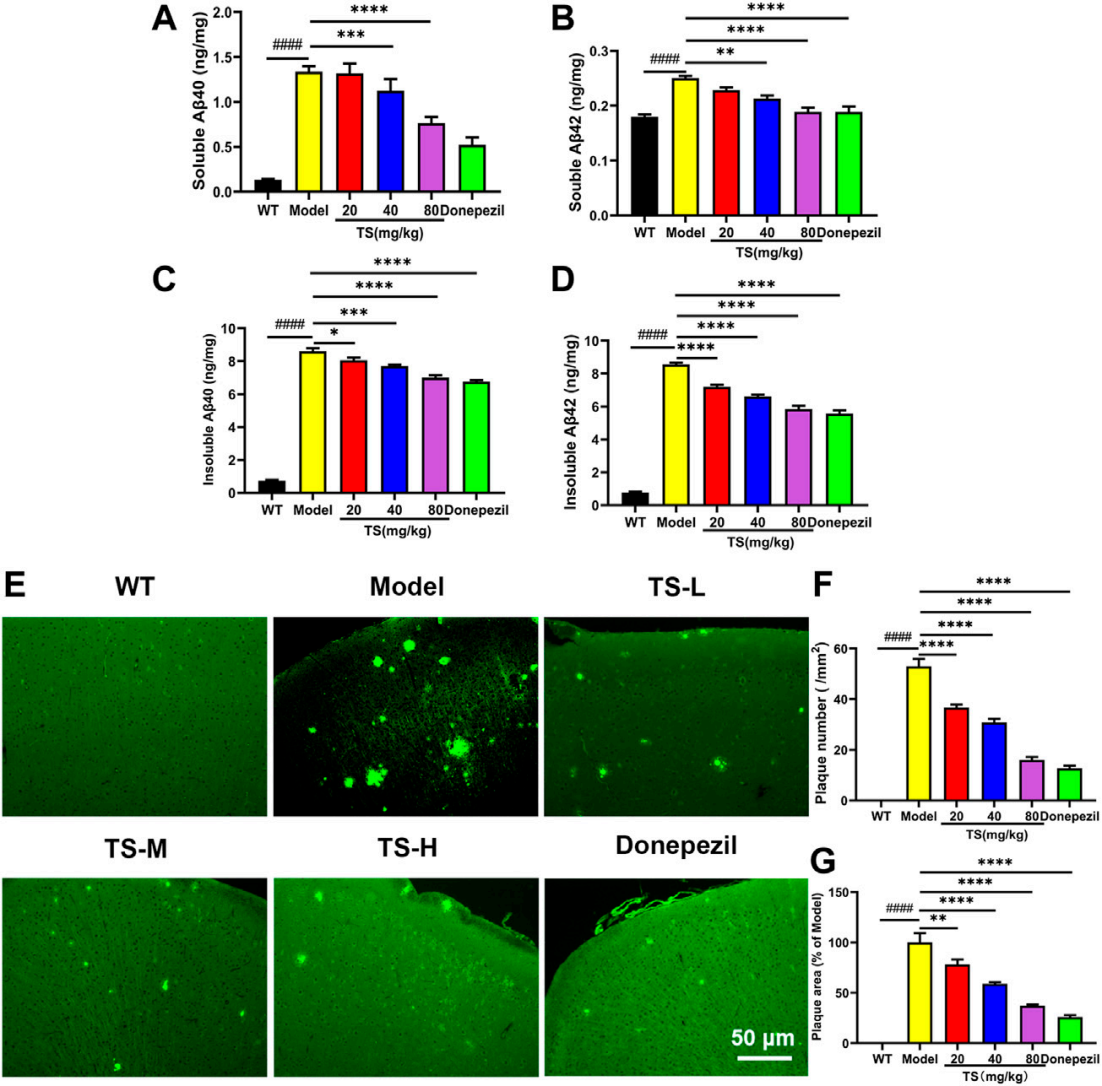
TS treatment decreased the levels of Aβ and senile plaque in the brain of APP/PS1 mice. **(A)** The effect of TS on the levels of soluble Aβ40 in the brain of APP/PS1 mice was measured by ELISA (^####^ *p <* 0.0001, ^***^ *p* = 0.0003, ^****^ *p <* 0.0001). **(B)** The effect of TS on the levels of soluble Aβ42 in the brain of APP/PS1 mice was measured by ELISA (^####^ *p <* 0.0001, ^**^ *p* = 0.0015, ^****^ *p <* 0.0001). **(C)** The effect of TS on the levels of insoluble Aβ40 in the brain of APP/PS1 mice was measured by ELISA (^####^ *p <* 0.0001, ^*^ *p* = 0.0220, ^***^ *p* = 0.0002, ^****^ *p <* 0.0001). **(D)** The effect of TS on the levels of insoluble Aβ42 in the brain of APP/PS1 mice was measured by ELISA (^####^ *p <* 0.0001, ^****^ *p <* 0.0001). **(E)** The effect of TS on the senile plaque in the brain of APP/PS1 mice was investigated by ThS fluorescence. **(F)** Quantitative analysis of the number of senile plaques in the brain of APP/PS1 mice (^####^ *p <* 0.0001, ^****^ *p <* 0.0001). **(G)** Quantitative analysis of the positive area of senile plaques in the brain of APP/PS1 mice (^####^ *p <* 0.0001, ^**^ *p* = 0.0069, ^****^ *p <* 0.0001). ^#^Compared with WT group; ^*^Compared with model group. Data are presented as mean ± SEM (*n* = 6).

The levels of soluble and insoluble Aβ40 and Aβ42 in brain tissue were measured by ELISA. Compared with WT mice, the levels of soluble and insoluble Aβ40 and Aβ42 in the brain of APP/PS1 mice significantly increased (Figures 3A–D, *p <* 0.0001), which were remarkably decreased after high-dose TS treatment (Figures 3A–D, *p <* 0.0001). Medium-dose TS treatment reduced the soluble Aβ42 level (Figure 3B, *p <* 0.01) and insoluble Aβ40 and Aβ42 levels (Figure 3C, *p <* 0.001; Figure 3D, *p <* 0.0001). Low-dose TS treatment only reduced the levels of insoluble Aβ40 and Aβ42 (Figure 3C, *p <* 0.05; Figure 3D, *p <* 0.0001). These results indicated that TS significantly reduced the level of Aβ in the brain of AD mice.

Thioflavine S (ThS) is a fluorescent dye with β-folding binding properties, which is widely used to observe the aggregation of amyloid plaques (Shin et al., 2021; Zhao et al., 2021). The ThS staining results showed no senile plaque in the brain of WT mice, and the number and area of senile plaques in the brain of APP/PS1 mice were significantly increased (Figures 3E,F, *p <* 0.0001), which were remarkably declined after TS treatment (Figures 3E,F). Collectively, these data showed that TS treatment was effective in reducing the production of Aβ and formation of senile plaques in the brain of APP/PS1 mice.

### 3.3 Total saikosaponins inhibits the expression of BACE1 via promoting the Nrf2 pathway in APP/PS1 mice to reduce Aβ deposition

**FIGURE 4:**
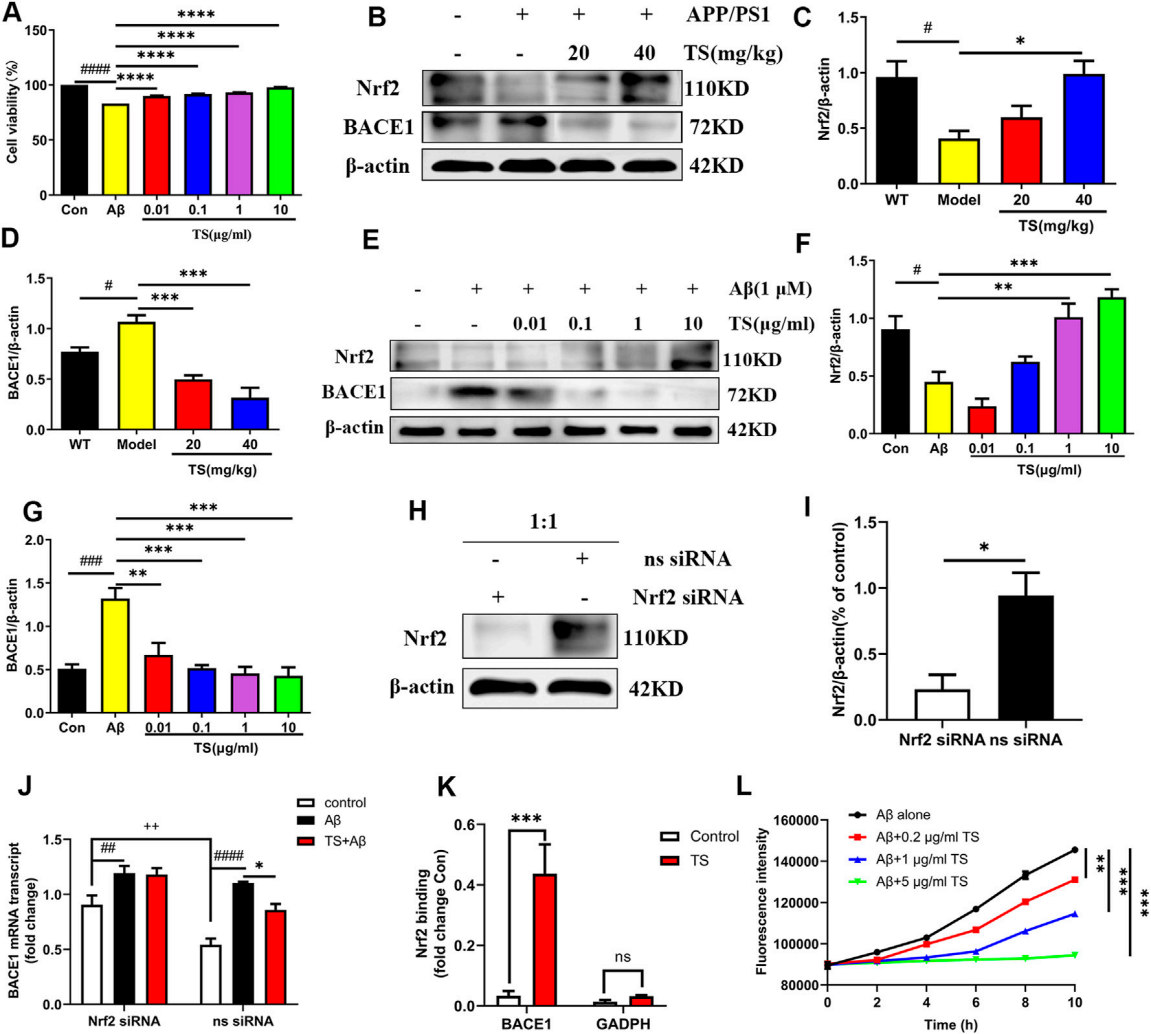
TS treatment promoted Nrf2 to inhibit the expression of BACE1 in the brain of APP/PS1 mice. **(A)** The effect of TS on the survival rate of Aβ-induced PC12 cells for 24 h was evaluated by MTT assay (^####^ *p <* 0.0001, ^****^ *p <* 0.0001). **(B)** The effect of TS on Nrf2 and BACE1 proteins in the brain of APP/PS1 mice were detected by Western blot. **(C)** Quantitative analysis of the relative expression level of Nrf2 protein in each group (^#^ *p* = 0.0195, ^*^ *p* = 0.0151). **(D)** Quantitative analysis of the relative expression level of BACE1 protein in each group (^#^ *p* = 0.0343, ^***^ *p* = 0.0008, ^***^ *p* = 0.001). **(E)** The effect of TS on Nrf2 and BACE1 protein in Aβ-induced PC12 cells were detected by Western blot. **(F)** Quantitative analysis of the relative expression concertation of Nrf2 protein in each group (^#^ *p* = 0.0119, ^**^ *p* = 0.0027, ^***^ *p* = 0.0003). **(G)** Quantitative analysis of the relative expression concertation of BACE1 protein in each group (^###^ *p* = 0.0003, ^**^ *p* = 0.0016, ^***^ *p* = 0.0003, ^***^ *p* = 0.0002, ^***^ *p* = 0.0001). **(H)** PC12 cells were transfected with Nrf2-siRNA and transfection reagent at 1:1 for 36h, and the expression of Nrf2 was detected by Western blotting. **(I)** Quantitative analysis of the relative expression level of Nrf2 protein in Nrf2-siRNA and ns-siRNA groups (^*^ *p* = 0.0199). **(J)** The effect of TS on transcription the level of BACE1 in Nrf2^−/−^ PC12 cells was investigated by qPCR (^##^ *p* = 0.0093, ^####^ *p* < 0.0001, ^*^ *p* = 0.0234, ^++^ *p* = 0.0030). **(K)** The effect of TS on the level of Nrf2 and BACE1 promoter binding was investigated by CHIP experiment (^***^ *p* = 0.0008). **(L)** The effect of TS on the level of Aβ aggregation was detected by ThT fluorescence method (^**^ *p* = 0.0058, ^***^ *p* = 0.0003, ^***^ *p* = 0.0002). ^#^: Comparing with WT group; ^*^: Compared with model group; ^+^: Compared with ns siRNA group. Data are presented as mean ± SEM (*n* = 3).

Aβ peptide is produced by sequential cleavage of APP mediated by BACE1 and γ-secretase, and the increase of BACE1 will lead to a sharp increase in Aβ production (Osama et al., 2020). Therefore, the effects of TS on the expression of BACE1 and Nrf2 were examined to explore the mechanism of TS-mediated reduction of Aβ production. First, MTT experiments were performed, and results revealed that TS could significantly inhibit the cytotoxicity induced by Aβ in PC12 cells (Figure 4A, *p <* 0.0001). Subsequently, the effects of TS on Nrf2 and BACE1 protein expression *in vivo* and *in vitro* were investigated using the APP/PS1 mouse model and Aβ-induced PC12 cell model. The results showed that compared with WT mice and blank PC12 cells, the expression level of Nrf2 protein in the model group decreased *in vivo* (Figures 4B,C, *p <* 0.05) and *in vitro* (Figures 4E,F, *p <* 0.05), and the expression of BACE1 protein increased *in vivo* (Figures 4B,D, *p <* 0.05) and *in vitro* (Figures 4E,G, *p <* 0.001). After TS treatment, the expression of Nrf2 protein increased *in vivo* (Figures 4B,C) and *in vitro* (Figures 4E,F). Similarly, the expression of BACE1 protein decreased *in vivo* (Figures 4B,D) and *in vitro* after TS treatment (Figures 4E,G).

PC12 cells were transfected with Nrf2-siRNA to silence the Nrf2 gene to explore the role of Nrf2 in the anti-AD effect of TS. The transfection efficiency reached more than 50% based on the results of Western blot analysis (Figures 4H,I, *p <* 0.05). QPCR was used to investigate the effect of TS on BACE1 transcription after Nrf2 silencing (Figure 4J). The result showed that the transcription level of BACE1 in the model group increased significantly (Figure 4J, *p <* 0.0001) compared with the blank group, which was decreased after TS treatment (Figure 4J, *p <* 0.05). After Nrf2 gene silencing, the inhibitory effect of TS on BACE1 transcription was remarkably attenuated, indicating that TS inhibits the transcription of BACE1 through Nrf2.

Next, ChIP assay was performed to study whether TS can promote the binding of Nrf2 to BACE1 promoter. Nrf2 binding to ARE1 in rat BACE1 ARE1 promoter was increased in TS-treated PC12 cells compared with controls (Figure 4K, *p <* 0.001), but the negative primers on rat GAPDH promoter had no significant change. Therefore, Nrf2 can directly bind to the ARE1 region of BACE1 promoter, and TS can promote the binding of Nrf2 and BACE1 promoter.

ThT fluorescence assay was used to evaluate the effect of TS on Aβ aggregation. The results showed that Aβ gradually accumulates from monomer to fibril formation with time, which was indicated by the increasing fluorescence density of ThT. TS incubation could inhibit Aβ aggregation in a concentration-dependent manner (Figure 4L). Co-incubation of 5 μg/ml of TS with 10 μM of Aβ could inhibit the aggregation of Aβ (Figure 4L, *p <* 0.001). Collectively, these results showed that TS could reduce the level of BACE1 by activating the expression of Nrf2, thereby reducing the formation and aggregation of Aβ.

### 3.4 Total saikosaponins treatment reduces *p-*tau protein level via inducing autophagy

**FIGURE 5:**
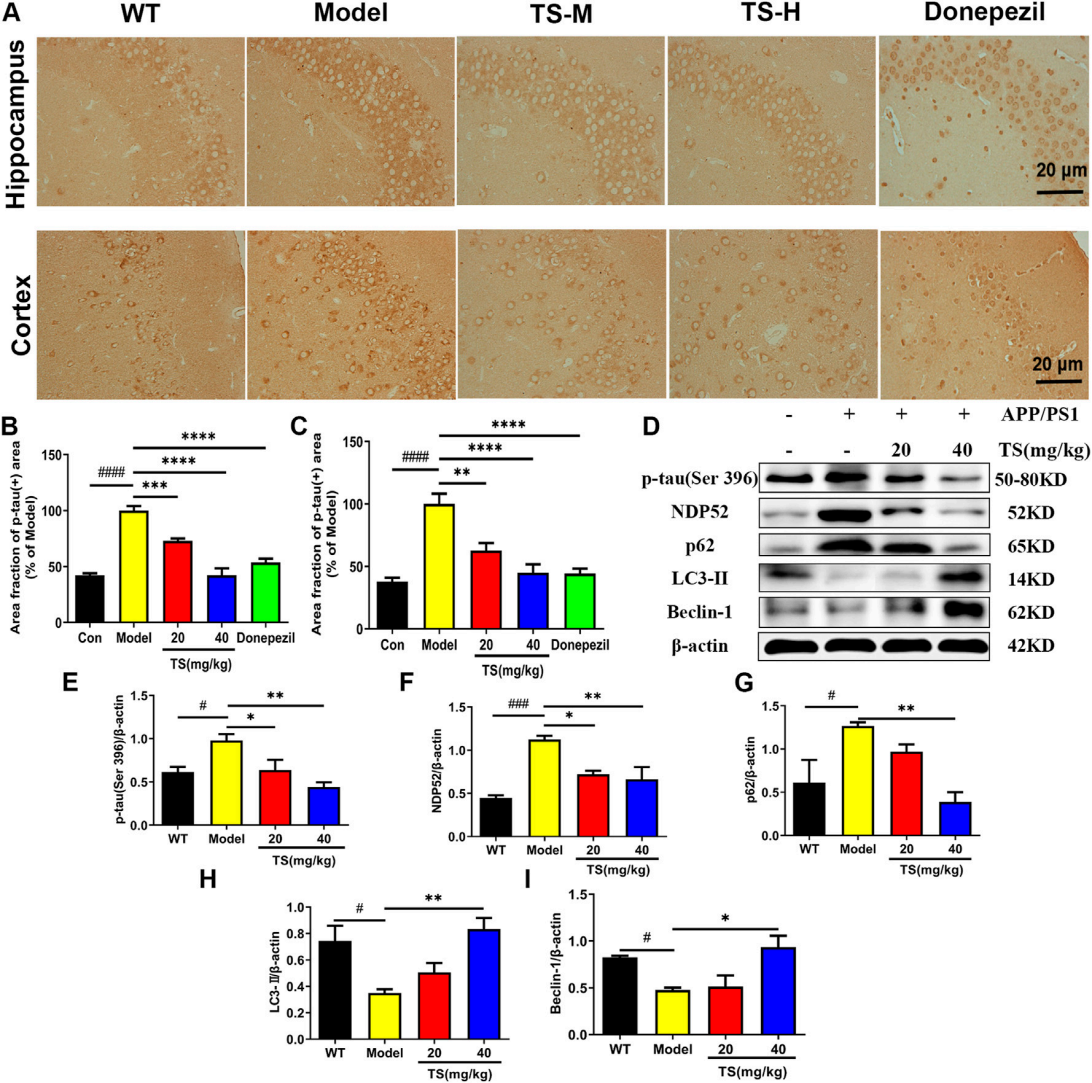
Effects of TS on expressions of *p-*tau, NDP52, p62 and LC3-II proteins in APP/PS1 mice. **(A)** The effect of TS on *p-*tau (ser396) in hippocampus and cortex of APP/PS1 mice was investigated by immunohistochemistry. **(B)** Quantitative analysis of the percentage of *p-*tau (Ser396) positive area in the hippocampus of APP/PS1 mice (^####^ *p <* 0.0001, ***p = 0.0007, ****p < 0.0001). **(C)** Quantitative analysis of the percentage of *p-*tau (Ser396) positive area in the cortex of APP/PS1 mice (^####^ *p <* 0.0001, **p = 0.0016, ****p < 0.0001). **(D)** The effects of TS on expressions of *p-*tau (Ser 396), NDP52, p62 and LC3-II proteins in the brain of APP/PS1 mice were detected by Western blot. **(E)** Quantitative analysis of the relative expression level of *p-*tau (Ser 396) protein in each group (^#^ *p* = 0.0332, ^*^ *p* = 0.0436, ^**^ *p* = 0.0042). **(F)** Quantitative analysis of the relative expression level of NDP52 protein in each group (^###^ *p* = 0.0008, *p = 0.0177, **p = 0.0086). **(G)** Quantitative analysis of the relative expression level of p62 protein in each group (^#^ *p* = 0.0379, **p = 0.0086). **(H)** Quantitative analysis of the relative expression level of LC3-II protein in each group (^#^ *p* = 0.0216, ^**^ *p* = 0.0073). **(I)** Quantitative analysis of the relative expression level of Beclin-1 protein in each group (^#^ *p =* 0.0496, *p = 0.0136). ^#^Compared with WT group; *Compared with model group. Data are presented as mean ± SEM (*n* = 3).

Hyperphosphorylation of tau protein leads to formation of neurofibrillary tangles and cognitive impairment. The effect of TS on the level of *p-*tau (Ser396) in the hippocampus and cortex of APP/PS1 mice was evaluated by immunohistochemical staining. Compared with WT mice, the levels of *p-*tau in the hippocampus and cerebral cortex of the model group mice were significantly higher (Figures 5A,B, *p <* 0.0001; Figures 5A,C, *p <* 0.0001), whereas the levels of *p-*tau in the low and middle-dose TS treatment groups were significantly lower than those in the model group (hippocampus: Figures 5A,B, *p <* 0.001 and *p <* 0.0001, respectively; cerebral cortex: Figures 5A,C, *p <* 0.01 and *p <* 0.0001, respectively). The effect of TS on the level of *p-*tau protein *in vivo* and *in vitro* was further investigated by Western blot. The level of *p-*tau in the brain of APP/PS1 mice was higher than that of WT mice (Figures 5D,E, *p <* 0.05), whereas low and middle-dose TS treatment significantly decreased the level of *p-*tau in the brain of APP/PS1 mice (Figures 5D,E, *p <* 0.05 and *p <* 0.01 respectively). These data indicated that TS treatment could reduce *p-*tau level in the brain of APP/PS1 mice.

In exploring the mechanism of TS reducing *p-*tau protein level, the effects of TS on autophagy were examined. The changes in the expression of autophagic proteins were assessed by Western blot assay. The results showed that compared with WT mice, the expression of LC3-II and Beclin-1 in the model group decreased (Figures 5D,H, *p <* 0.05; Figures 5D,I, *p <* 0.05), whereas the expression of NDP52 and p62 increased (Figures 5D,F, *p <* 0.001; Figures 5D,G, *p <* 0.05), indicating that autophagy was inhibited. After treatment with TS, the expression of LC3-II and Beclin-1 increased significantly (Figures 5D,H,I), whereas the expression of NDP52 and p62 decreased in a dose-dependent manner (Figures 5D,F,G).

**FIGURE 6:**
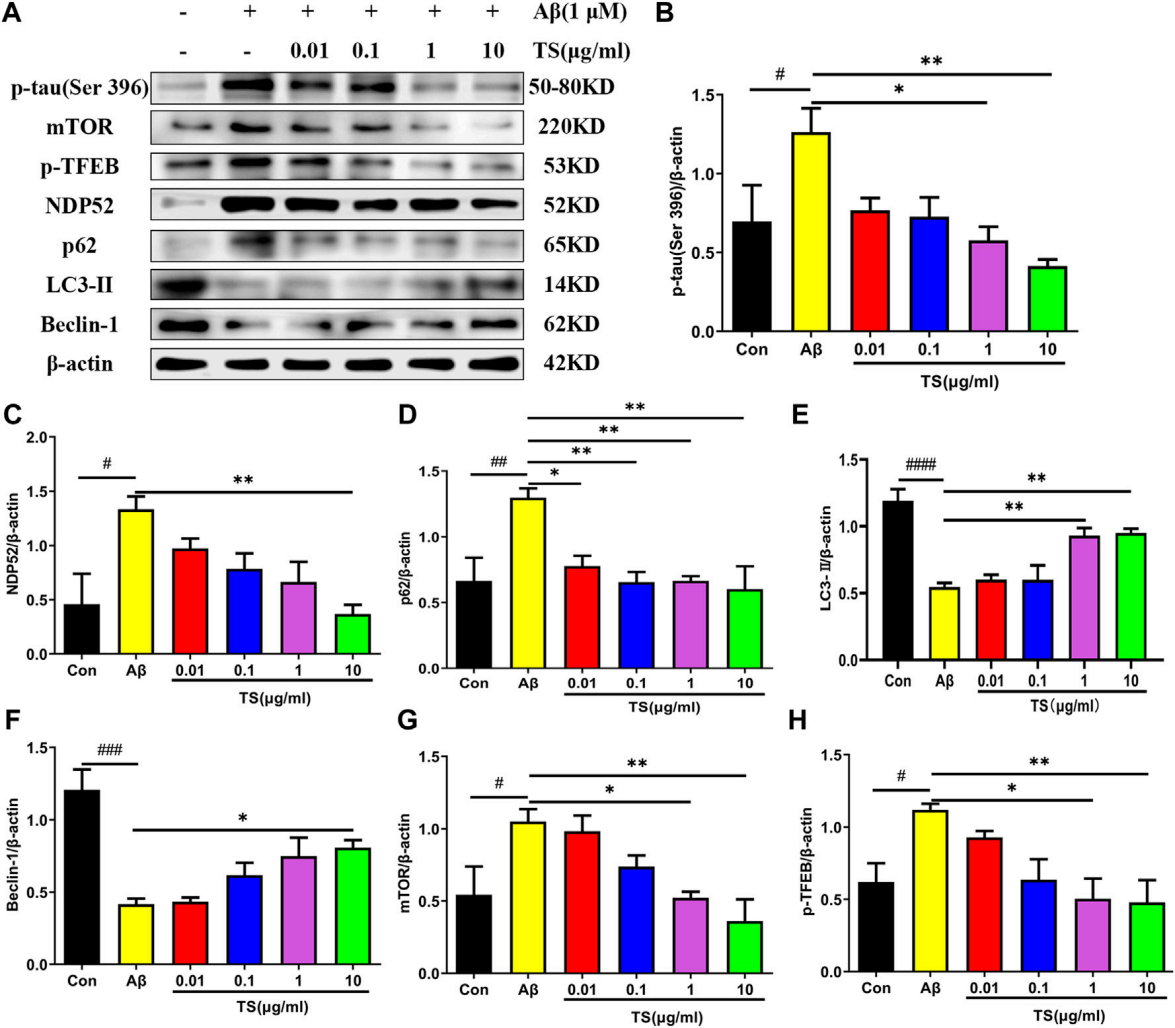
TS treatment promoted autophagy and clear *p-*tau. **(A)** The effect of TS on *p-*tau (Ser 396), NDP52, p62 and LC3-II and Beclin-1 proteins in the Aβ-induced PC12 cells were detected by Western blot. **(B)** Quantitative analysis of the relative expression concertation of *p-*tau (Ser 396) protein in each group (^#^ *p* = 0.0409, *p = 0.0130, **p = 0.0029). **(C)** Quantitative analysis of the relative expression concertation of NDP52 protein in each group (^#^ *p* = 0.0113, **p = 0.0057). **(D)** Quantitative analysis of the gray value of p62 protein bands in each group (^##^ *p* = 0.0093, *p = 0.0312, **p = 0.0084, **p = 0.0093, **p = 0.0047). **(E)** Quantitative analysis of the relative expression concertation of LC3-II protein in each group (^####^ *p <* 0.0001, **p = 0.0053, **p = 0.0037). **(F)** Quantitative analysis of the relative expression concertation of Beclin-1 protein in each group (^###^ *p* = 0.0002, *p = 0.0356). **(G)** Quantitative analysis of the relative expression level of mTOR protein in each group (^#^ *p* = 0.0450, *p = 0.0359, **p = 0.0068). **(H)** Quantitative analysis of the relative expression level of *p-*TFEB protein in each group (^#^ *p* = 0.0435, *p = 0.0129, **p = 0.0099). ^#^Compared with WT group; *Compared with model group. Data are presented as mean ± SEM (*n* = 3).

Consistent results were obtained in *in vivo* experiments using Aβ-induced PC12 cells. As shown in Figure 6, the level of *p-*tau in the model group was increased compared with the blank group (Figures 6A,B, *p <* 0.05), whereas TS treatment could reduce the level of *p-*tau in Aβ-induced PC12 cells (Figures 6A,B, *p <* 0.05 and *p <* 0.01, respectively). Meanwhile, levels of autophagy-related proteins also changed in Aβ-induced PC12 cells. Compared with the blank group, the expression of LC3-II and Beclin-1 in the model group was decreased (Figures 6A,E, *p <* 0.0001; Figures 6A,F, *p <* 0.001), whereas the expression of NDP52, P62, mTOR and *p*-TFEB was increased (Figures 6A,C, *p <* 0.05; Figures 6A,D, *p <* 0.01; Figures 6A,G, *p <* 0.05; Figures 6A,H, *p <* 0.05), which indicated that autophagy was suppressed. TS treatment could significantly increase the expression of LC3-II and Beclin-1 (Figures 6A,E,F) and reduce the expression of NDP52, P62, mTOR and *p*-TFEB (Figures 6A,C,D,G,H), indicating that TS may reduce the *p-*tau protein level by promoting autophagy.

**FIGURE 7:**
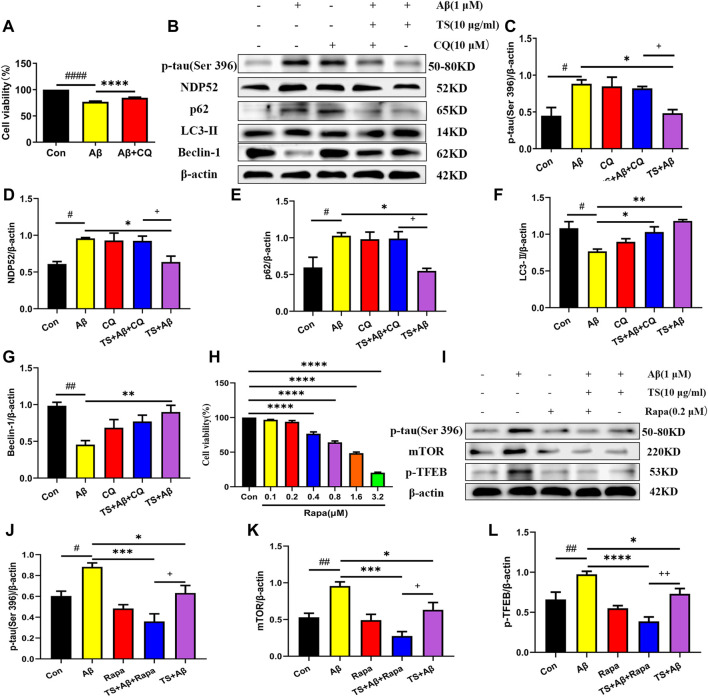
TS promoted autophagy in Aβ-induced PC12 cells to clear *p-*tau. **(A)** The effect of CQ on the survival rate of Aβ-induced PC12 cells for 24 h was evaluated by MTT assay (^####^ *p <* 0.0001, ^****^ *p <* 0.0001). **(B)** The effects of CQ on expression of *p-*tau (Ser 396), NDP52, p62, LC3-II and Beclin-1 proteins in the Aβ-induced PC12 cells after TS treatment were detected by Western blot. **(C)** Quantitative analysis of the relative expression level of *p-*tau (Ser 396) protein in each group (^#^ *p* = 0.0128, *p = 0.0205, ^+^ *p* = 0.0495). **(D)** Quantitative analysis of the relative expression level of NDP52 protein in each group (^#^ *p* = 0.0134, *p = 0.0217, ^+^ *p* = 0.0386). **(E)** Quantitative analysis of the relative expression level of p62 protein bands in each group (^#^ *p* = 0.0226, *p = 0.0124, ^+^ *p* = 0.0200). **(F)** Quantitative analysis of the relative expression level of LC3-II protein in each group (^#^ *p* = 0.0101, *p = 0.0277, **p = 0.0016). **(G)** Quantitative analysis of the relative expression level of Beclin-1 protein in each group (^##^ *p* = 0.0013, **p = 0.0056). **(H)** MTT assay was used to test the effect of Rapa on PC12 cells for 24 h (^****^ *p <* 0.0001). **(I)** The effects of Rapa on expression of *p-*tau (Ser 396), mTOR and *p*-TFEB proteins in the Aβ-induced PC12 cells after TS treatment were detected by Western blot. **(J)** Quantitative analysis of the relative expression level of *p-*tau (Ser 396) protein in each group (^#^ *p* = 0.00175, ***p = 0.0002, *p = 0.0315, ^+^ *p* = 0.0442). **(K)** Quantitative analysis of the relative expression level of mTOR protein in each group (^##^ *p* = 0.0062, ***p = 0.0002, *p = 0.0305, ^+^ *p* = 0.0250). **(L)** Quantitative analysis of the relative expression level of *p-*TFEB protein in each group (^##^ *p* = 0.0041, ****p < 0.0001, *p = 0.0288, ^++^ *p* = 0.0081). ^#^: Compared with WT group; *Compared with model group; ^+^Comparing with TS+Aβ group. Data are presented as mean ± SEM (*n* = 3).

Subsequently, CQ, an inhibitor of autophagic degradation, was employed to further clarify the mechanism of TS on autophagy and the relationship between autophagy inhibiting and *p-*tau reducing effects of TS. The MTT experiment results showed that CQ treatment could protect PC12 cells from Aβ-induced reduction in cell viability (Figure 7A). The levels of *p-*tau and autophagic proteins were detected using western blot analysis. It was found that compared with the TS group without CQ treatment, the *p-*tau level of the TS group treated with CQ was higher (Figures 7B,C, *p <* 0.05), indicating that CQ could inhibit *p-*tau reducing effect of TS. Western blotting results of autophagic proteins showed that compared with the TS dose group without CQ treatment, the level of NDP52 in the TS group treated with CQ increased (Figures 7B,D, *p <* 0.05), and the expression of Beclin-1 decreased (Figures 7B,E, *p <* 0.05), indicating that CQ inhibited the degradation of autophagosomes. No significant difference in the expression of p62 and LC3-II was observed. Furthermore, Rapamycin (Rapa), an mTOR inhibitor and autophagy inducer, was employed to further clarify the mechanism of TS on *p-*tau clearance through lysosome and autophagy. MTT results showed that Rapa treatment had no significant effect on cell viability at 0.1 or 0.2 μM (Figure 7H). We then examined the levels of *p-*tau and lysosome-associated proteins via western bolt analysis. It was found that compared with the TS group without Rapa treatment, the level of *p-*tau, mTOR and *p*-TFEB in the TS group treated with Rapa was decreased (Figures 7I–K, *p <* 0.05; Figures 7I,L, *p <* 0.01), indicating that Rapa could prompt *p-*tau clearance effect of TS through increasing the number of lysosomes and promoting the degradation of autophagosomes. Taken together, TS could reduce *p-*tau protein level by promoting autophagy, and the increase of autophagy flux is positively correlated with the clearance of *p-*tau.

### 3.5 Total saikosaponins treatment attenuates oxidative stress and inflammation in brain tissue of APP/PS1 mice

**FIGURE 8:**
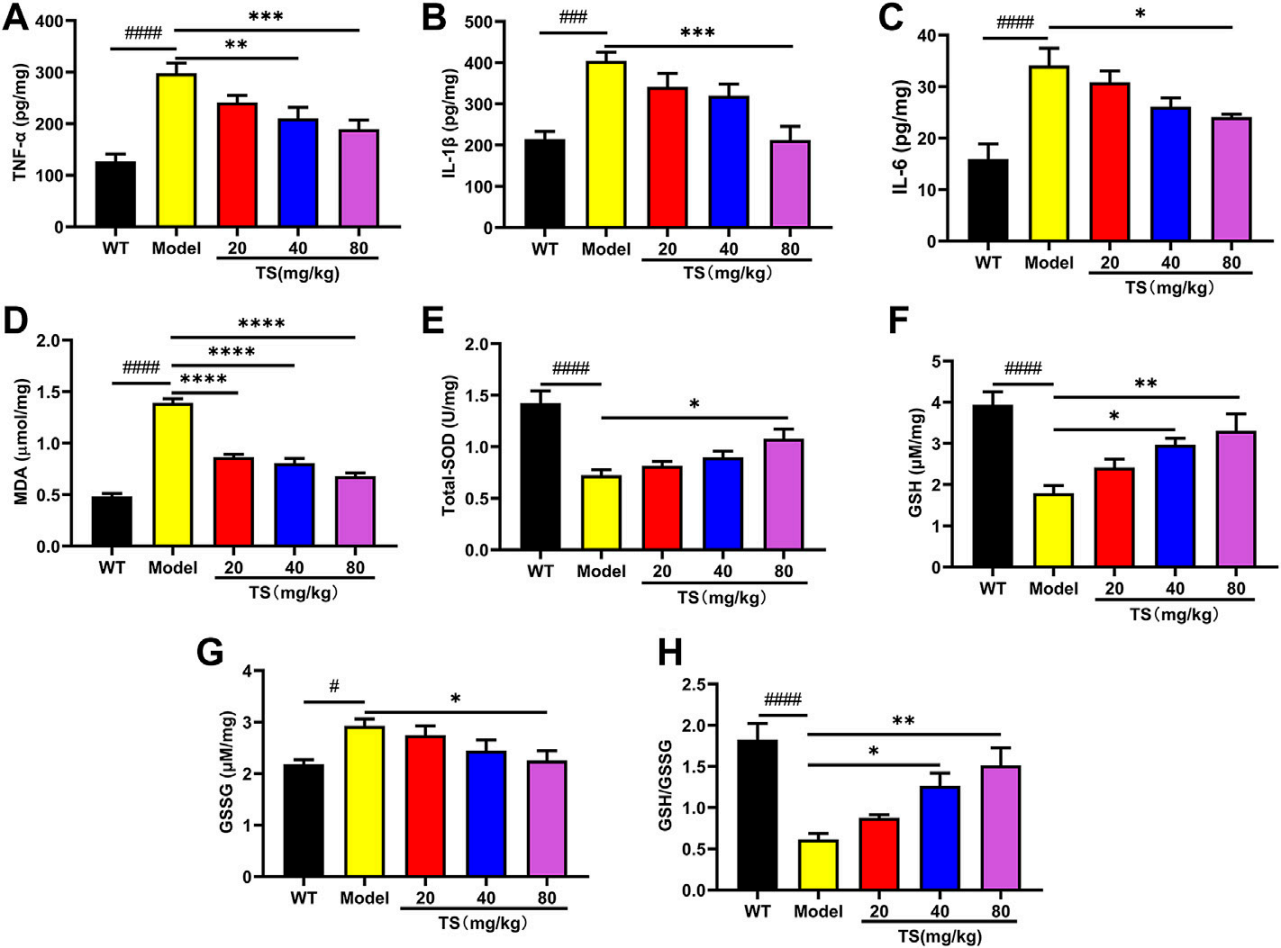
TS treatment inhibited cerebral inflammation and oxidative stress in APP/PS1 mice. **(A)** The effect of TS on the levels of TNF-α in the brain of APP/PS1 mice was measured by ELISA (^####^ *p <* 0.0001, **p = 0.0065, ***p = 0.0008). **(B)** The effect of TS on the levels of IL-1β in the brain of APP/PS1 mice was measured by ELISA (^###^ *p* 0.0002, ***p = 0.0002). **(C)** The effect of TS on the levels of IL-6 in the brain of APP/PS1 mice was measured by ELISA (^####^ *p <* 0.0001, *p = 0.0209). **(D)** The effect of TS on the levels of MDA in the brain of APP/PS1 mice was measured by ELISA (^####^ *p <* 0.0001, ****p < 0.0001). **(E)** The effect of TS on the levels of SOD in the brain of APP/PS1 mice was measured by ELISA (^####^ *p <* 0.0001, *p = 0.0129). **(F)** The effect of TS on the levels of GSH in the brain of APP/PS1 mice was measured by ELISA (^####^ *p <* 0.0001, *p = 0.0177, **p = 0.0020). **(G)** The effect of TS on the levels of GSG in the brain of APP/PS1 mice was measured by ELISA (^#^ *p* = 0.0123, *p = 0.0248). **(H)** Quantitative analysis of the ratio of GSH/GSSG (^####^ *p <* 0.0001, *p = 0.0178, **p = 0.0010). ^#^Compared with WT group; *Compared with model group. Data are presented as mean ± SEM (*n* = 6).

Considering the key role of oxidative stress and inflammation in the etiology of AD, the levels of key biomarkers of oxidative stress and inflammation in mice brain were evaluated using ELISA. Firstly, the levels of inflammatory factors were detected. The results showed that compared with the WT mice, the levels of TNF-α, IL-1βand IL-6 in the brain of the model group mice were significantly increased (Figures 8A–C, *p <* 0.0001). While high dose of TS could significantly reduce the levels of TNF-α, IL-1β and IL-6 (Figures 8A–C, *p <* 0.01, *p <* 0.0001, *p <* 0.05, respectively) in the brain of AD mice. Middle dose of TS only decreased the level of TNF-α (Figure 8A, *p <* 0.05).

Then the levels of oxidative stress markers in mice brain were measured, including the levels of MDA, SOD and GSH. The results showed that compared with WT mice, the levels of MDA and GSSG in the brain of APP/PS1 mice were increased (Figures 8D,G, *p <* 0.0001, *p <* 0.05, respectively), while the levels of SOD, GSH and the ratio of GSH/GSSG were significantly decreased (Figures 8E,F,H, all *p <* 0.0001). Compared with the model group, different dose of TS treatment significantly reduced the content of MDA in the brain of APP/PS1 mice (Figure 8D, *p <* 0.0001). High-dose TS treatment could significantly increase the content of antioxidant SOD, GSH and the ratio of GSH/GSSG (Figures 8E,F,H, *p <* 0.05, *p <* 0.01, *p <* 0.01, respectively), and decrease the content of GSSG (Figure 8G, *p <* 0.05) in the brain of AD mice. Medium dose of TS can increase the level of GSH and the ratio of GSH/GSSG (Figures 8F,H, *p <* 0.05). These results suggested that TS treatment could inhibit oxidative stress and neuroinflammation in the brain of AD mice.

### 3.6 Total saikosaponins inhibits neuroinflammation through Nrf2/NF-κB pathway

**FIGURE 9:**
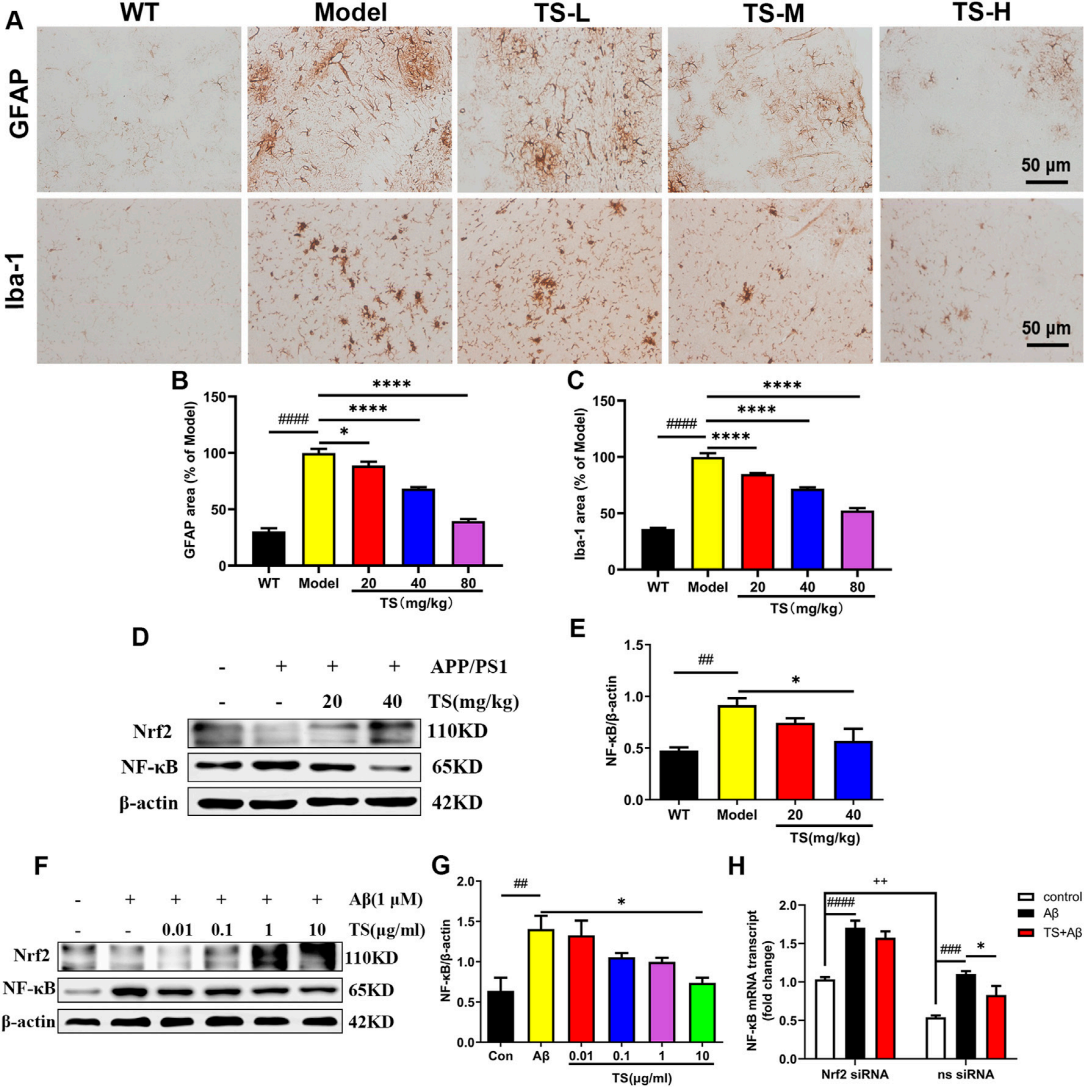
TS treatment reduced astrocytes and microglia activation in APP/PS1 mice. **(A)** The effects of TS on the activation of astrocytes and microglia in the brain of APP/PS1 mice were investigated by immunohistochemical staining. **(B)** Quantitative analysis of the percentage of GFAP positive area in the brain of APP/PS1 mice (^####^ *p <* 0.0001, *p = 0.0312, ****p < 0.0001). **(C)** Quantitative analysis of the percentage of Iba-1 positive area in the brain of APP/PS1 mice (^####^ *p <* 0.0001, ****p < 0.0001). **(D)** The effects of TS on expressions of Nrf2 and NF-κB proteins in the brain of APP/PS1 mice were detected by Western blot. **(E)** Quantitative analysis of the relative expression level of NF-κB protein in each group (^##^ *p* = 0.0068, *p = 0.0243). **(F)** The effects of TS on expressions of Nrf2 and NF-κB proteins in the Aβ-induced PC12 cells were detected by Western blot. **(G)** Quantitative analysis of the relative expression level of NF-κB protein in each group (^##^ *p* = 0.0048, *p = 0.0126). **(H)** The effect of TS on the transcription level of NF-κB in Nrf2^−/−^ PC12 cells was detected by qPCR. (^###^ *p* = 0.0003, ^####^ *p <* 0.0001, ^*^ *p* = 0.0382, ^++^ *p* = 0.0013). ^#^Compared with WT group; *Compared with model group; ^+^Compared with ns siRNA group. Data are presented as mean ± SEM (*n* = 3).

The activation of astrocytes and microglia is considered as key event in the progression of AD, which was evaluated using Iba-1 and GFAP immunohistochemical staining. The results of GFAP immunostaining showed that compared with WT mice, the activation of astrocytes in the brain of model group mice was significantly increased (Figures 9A,B, *p <* 0.0001), while TS could significantly reduce the positive staining area of GFAP (Figures 9A,B), suggesting that TS could inhibit the excessive activation of astrocytes in the brain of AD mice. The results of Iba-1 immunostaining showed that the activation of microglia in the brain of model mice was significantly increased than that of WT mice (Figures 9A,C, *p <* 0.0001), and TS treatment could significantly reduce the activation of microglia (Figures 9A,C, *p <* 0.0001). The above results suggested that TS treatment could significantly inhibit the activation of glial cells including astrocytes and microglia in the brain of APP/PS1 mice.

In order to investigate the mechanism of TS in inhibiting neuroinflammation, the expression of Nrf2 and NF-κB protein in the brain of APP/PS1 mice and Aβ-induced PC12 cells were examined. The results showed that compared with blank group, the expression level of NF-κB protein in the model group increased both *in vivo* (Figures 9D,E, *p <* 0.01) and *in vitro* (Figures 9F,G, *p <* 0.01). After treatment with TS, the expression of NF-κB protein decreased in high dose of TS *in vivo* (Figures 9D,E, *p <* 0.05) and in 10 μg/ml of TS *in vitro* (Figures 9F,G, *p <* 0.05), suggesting that TS could inhibit the expression of NF-κB in the brain of APP/PS1 mice and Aβ-induced PC12 cells.

Nrf2-siRNA transfected PC12 cells were employed to further explore the role of Nrf2 in the down-regulation of NF-κB expression induced by TS. It was found that compared with the blank group, the transcription level of NF-κB in the model group increased significantly (Figure 9H, *p <* 0.001), which was decreased after treatment with TS (Figure 9H, *p <* 0.05). After Nrf2 gene silencing, the inhibitory effect of TS on NF-κB transcription was remarkably attenuated, suggesting that TS inhibits the transcription of NF-κB through Nrf2.

### 3.7 Total saikosaponins treatment ameliorate synaptic loss in APP/PS1 mice

**FIGURE 10:**
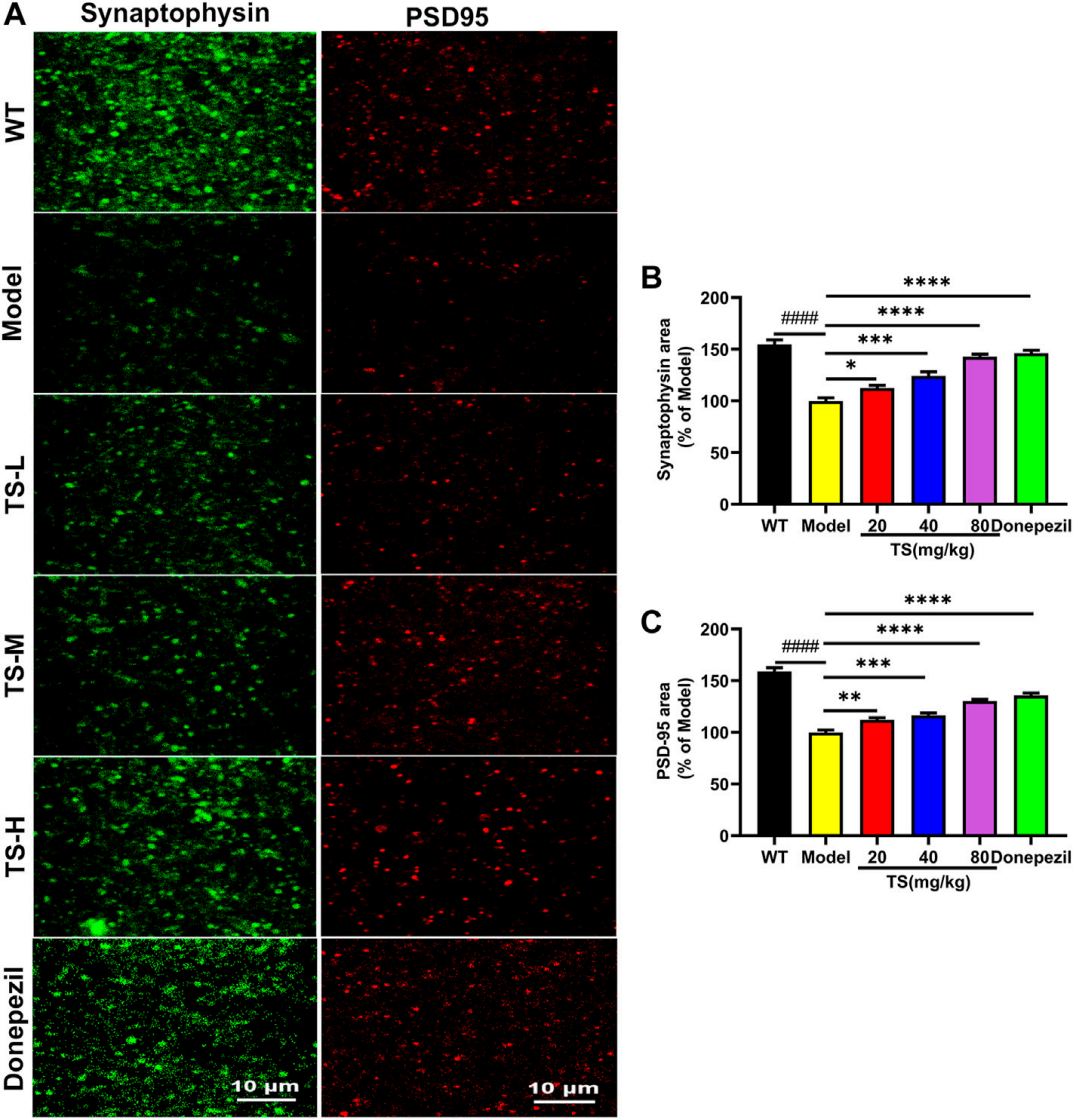
TS treatment improved synaptic dysfunction in APP/PS1 mice. **(A)** The effect of TS on the level of synaptophysin and PSD-95 in the brain of APP/PS1 mice was investigated by immunofluorescence assay. **(B)** Quantitative analysis of the percentage of synaptophysin positive area in the brain of APP/PS1 mice (^####^ *p <* 0.0001, *p = 0.0496, ****p < 0.0001). **(C)** Quantitative analysis of the percentage of PSD-95 positive area in the brain of APP/PS1 mice (^####^ *p <* 0.0001, **p = 0.0059, ***p = 0.0002, ****p < 0.0001). ^#^Compared with WT group; *Compared with model group. Data are presented as mean ± SEM (*n* = 6).

Synaptic loss is an important pathological feature of AD, which is highly correlated with cognitive function impairment in AD patients or animal models (John & Reddy, 2021). Presynaptic protein synaptophysin and postsynaptic protein PSD-95 are two synaptic marker proteins (John & Reddy, 2021). Postsynaptic density protein PSD-95 is the main scaffold protein of dendritic spine coordinating the relationship between neurotransmitters and receptors, and ultimately determining the synaptic response (Savioz et al., 2014; John & Reddy, 2021). Synaptophysin and PSD-95 immunofluorescence assay was performed to verify the effect of TS on synaptic loss. The results of immunofluorescence staining in mice brain showed that the levels of synaptophysin (Figures 10A,B, *p <* 0.0001) and PSD-95 (Figures 10A,C, *p <* 0.0001) in the brain of AD mice were significantly lower than those of WT mice. While TS treatment could significantly increase the levels of synaptophysin (Figures 10A,B) and PSD-95 (Figures 10A,C), suggesting that TS treatment could effectively prevent synaptic loss in the brain of AD mice.

### 3.8 Total saikosaponins treatment reverses the microbiota disorder in APP/PS1 mice

**FIGURE 11:**
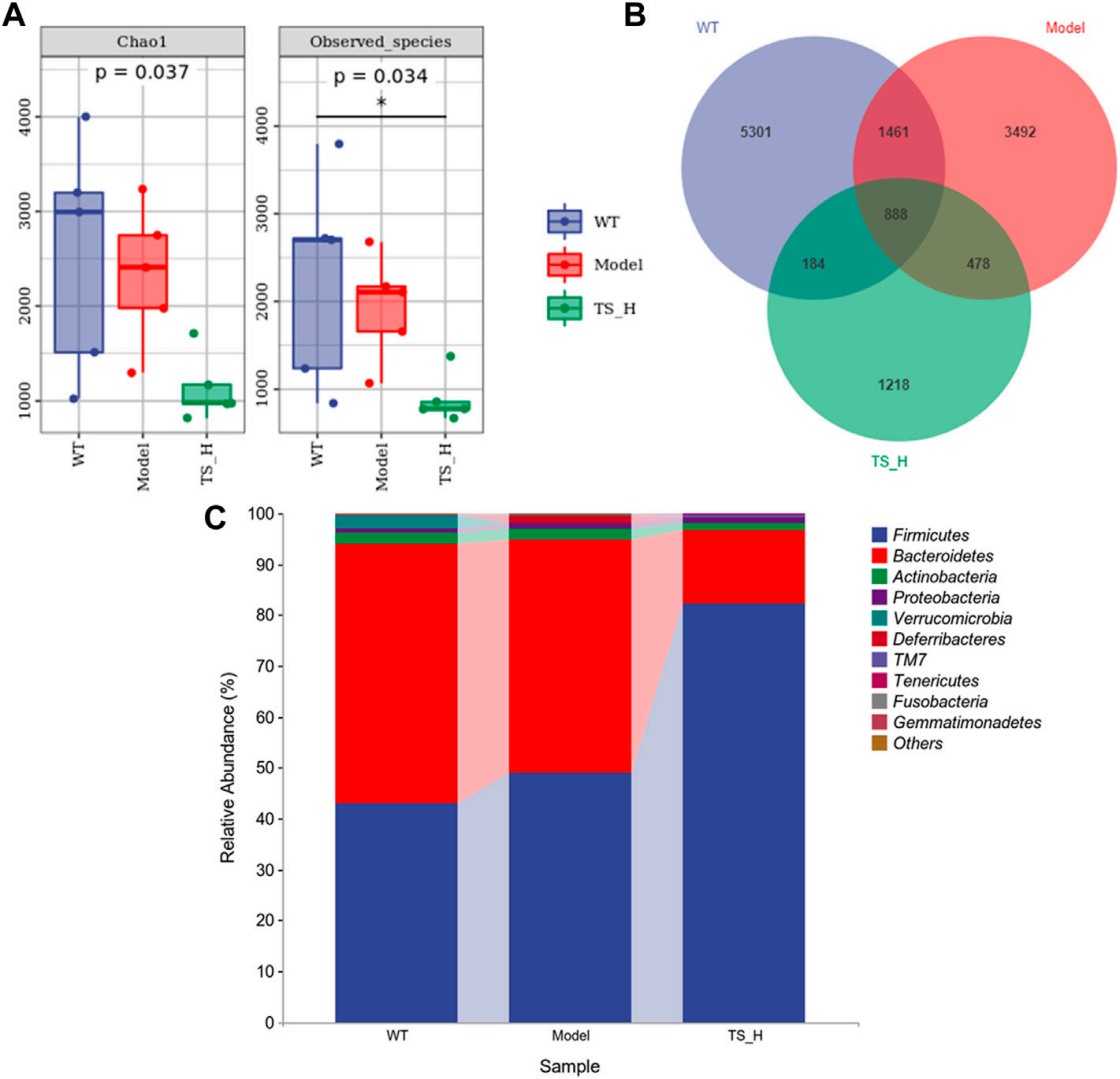
Differences in the diversity and composition of gut microbial genes among WT mice, APP/PS1 mice and TS treatment APP/PS1 mice. **(A)** Alpha diversity analysis-Chao1 index and Observed species index box chart. **(B)** Venn diagram. **(C)** Column pictures of relative abundance and composition of gut microbiota at phylum level in each group. *p < 0.05 versus WT group (*n* = 5).

**FIGURE 12:**
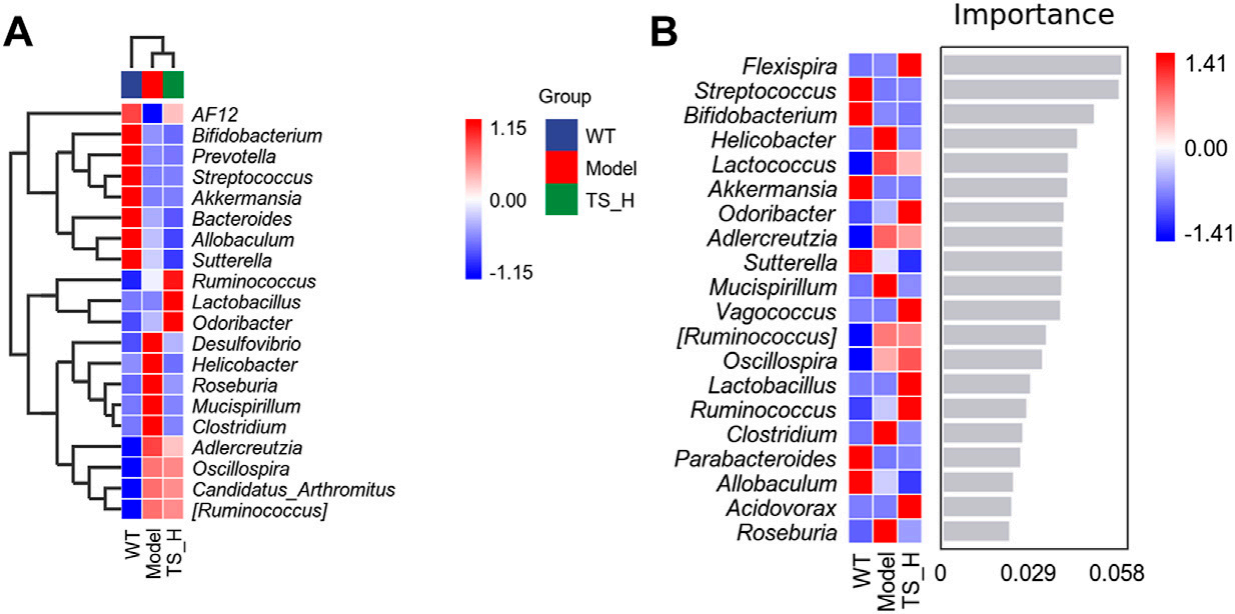
Heatmaps of most differentially abundant taxa in WT mice, APP/PS1 mice and TS treatment APP/PS1 mice. **(A)** Heat-map of microbial community composition. **(B)** Random forest analysis map. *n* = 5 for each treatment.

**FIGURE 13:**
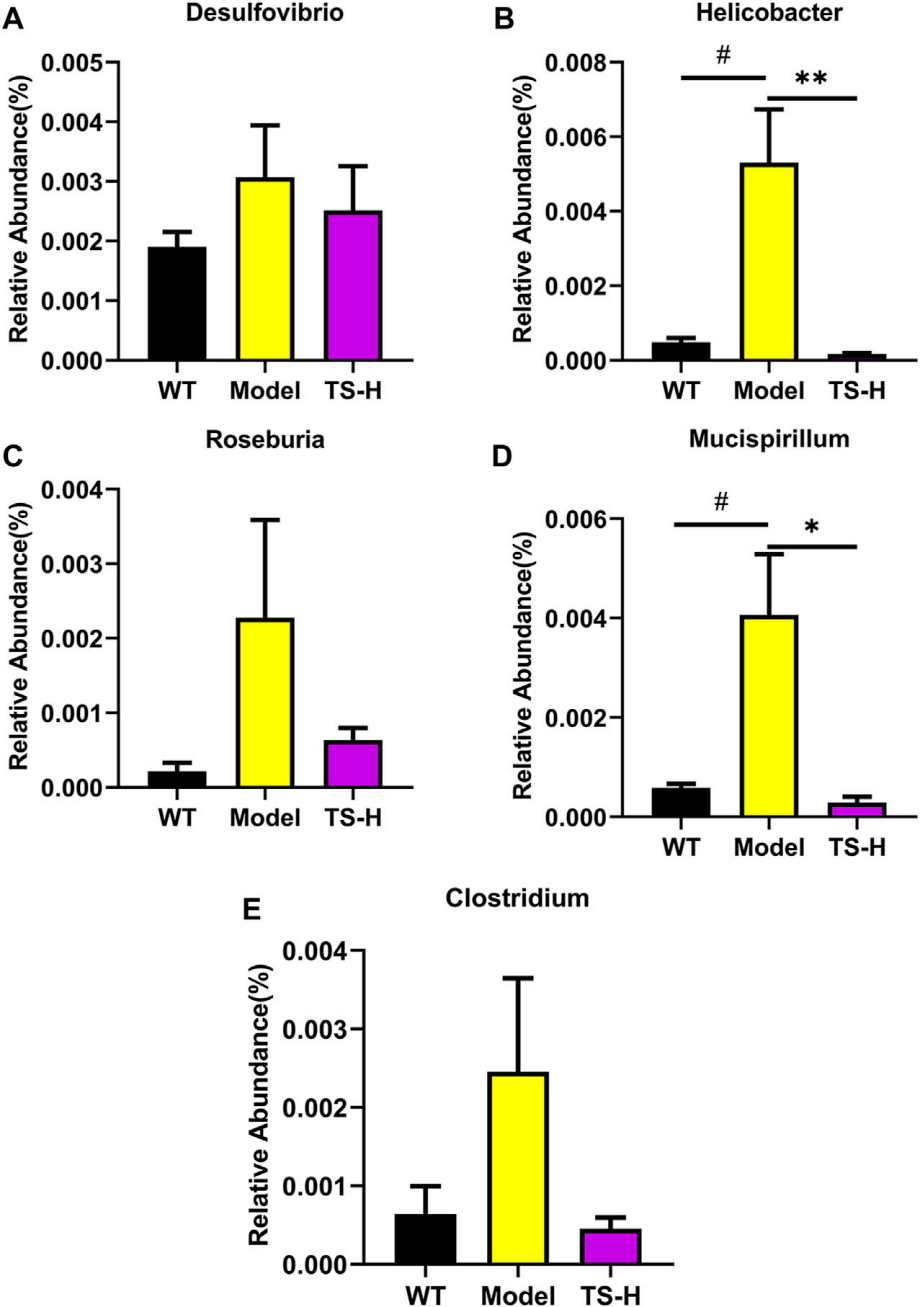
Relative abundance of five representative microbial species at the genus levels in WT mice, APP/PS1 mice and TS treatment APP/PS1 mice. **(A)** *Desulfovibrio*. **(B)** *Helicobacter* (^#^ *p* = 0.0113, **p = 0.0084). **(C)** *Roseburia*. **(D)** *Mucispirillum* (^#^ *p* = 0.0239, *p = 0.0169). **(E)** *Clostridium*. Data were expressed as mean ± SEM. ^#^Compared with WT group; *Compared with model group (*n* = 3).

16S rRNA gene sequencing was used to analyze and evaluate the effect of TS on GM in APP/PS1 mice. The Chao1 index and observed index show the significant change of bacterial communities in alpha diversity among different groups (Figure 11A), indicating the exits of microbiota dysbiosis in APP/PS1 mice, and TS reverses this pathological change. The Venn diagram showed the difference of ASV numbers detected in three groups of mice (Figure 11B), indicating that the composition of flora changed among the three groups. Then, the gut bacterial composition at the phylum level was profiled. As shown in Figure 11C, the main bacteria in three groups are identified. The result revealed that the microbiome at the phylum level underwent a disorder in APP/PS1 mice, and TS treatment remarkably affected the microbiome composition, indicating a potential mechanism and a therapy target of AD though the gut–brain axis. Heatmaps of most differentially abundant taxa revealed the change trend in three groups. Based on heatmap analysis (Figure 12), the abundance of *Desuflovibrio*, *Helicobacter*, *Mucispirillum*, *Roseburia*, and *Clostridium* increased in APP/PS1 mice, whereas it was reduced after TS treatment. Random forest analysis suggests that *Helicobacter* and *Mucispirillum* show a significant difference among the groups (Figure 13), indicating that these bacteria were involved in the pathogenesis of AD, and TS prevents cognitive impairment in APP/PS1 mice by remodeling the GM.

## 4 Discussion

**FIGURE 14:**
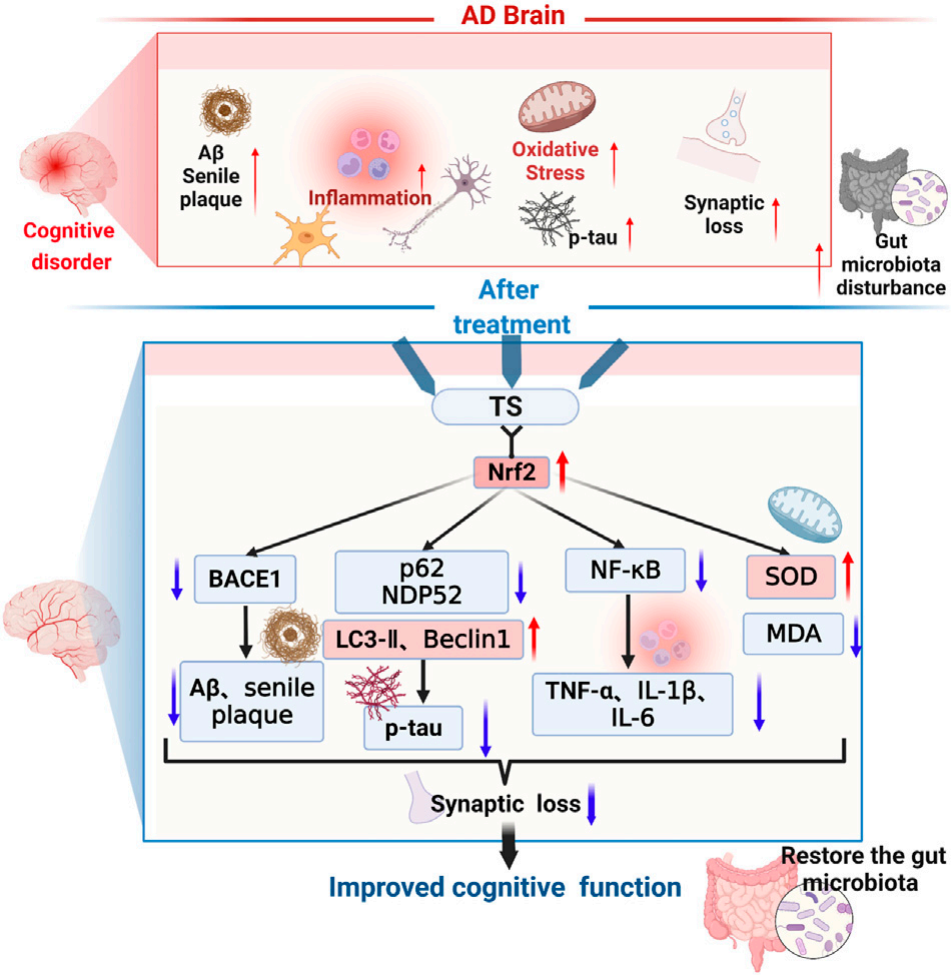
The therapeutic effects and related mechanisms of TS against AD. The following pathological phenomena occur in the AD brain: (1) increased Aβ generation, aggregation and increased deposition of senile plaques; (2) enhanced activation of microglia and astrocytes, elevated inflammatory cytokines levels; (3) increased oxidative stress and decreased secretion of antioxidant stress factors; (4) increased synaptic loss; (5) aggravated gut mictobiota dysbiosis and increased the pathogenic bacteria. Finally, these pathological changes led to the cognitive dysfunction of the AD patients. TS treatment could improve cognitive function through multiple mechanisms. Firstly, TS regulated the transcription and expression of BACE1 via Nrf2, reduced the generation and aggregation of Aβ, thus inhibited the deposition of senile plaque. Secondly, TS downregulated the expression of p62 and NDP52 by promoting autophagy, upregulated the expression of LC3-II and Beclin-1, thus promoting the clearance of *p-*tau. Thirdly, TS downregulated the transcription and expression of NF-κB via Nrf2, reduced the levels of inflammatory cytokines, thus ameliorated the neuroinflammation. Fourthly, TS increased the level of SOD and reduced the level of MDA, alleviating oxidative stress. Eventually, TS ameliorated synapse loss and alleviated the gut microbiota dysbiosis. Red arrows represent upregulation and blue arrows represent downregulation. AD, Alzheimer’s disease; TS, Total saikosaponins; Nrf2, nuclear factor 2; BACE1, β-secretase 1; Aβ, amyloid-beta; *p-*tau, phosphorylated tau; NF-κB, nuclear factor-κB; TNF-α, tumour necrosis factor α; IL-1β, interleukin-1β; IL-6, interleukin-6; SOD, superoxide dismutase; MDA, malondialdehyde.

In this study, it was demonstrated that TS has protective effects on cognitive function and neuropathological impairments in APP/PS1 transgenic mice, reducing Aβ production, senile plaque deposition, *p-*tau level, oxidative stress, and inflammatory response; inhibiting glial cell activation; and improving synaptic function. As shown in Figure 14, the underlying mechanism was found to involve reducing Aβ deposition by activating the Nrf2/BACE1 signaling pathway and promoting autophagy by regulating the expression of NDP52 and other autophagy-related proteins, thereby reducing the level of *p-*tau, which has been rarely reported in previous literature. With regard to neuroinflammation, TS can downregulate NF-κB expression through Nrf2, which inhibited the activation of glial cells and reduced the production of inflammatory factors. Moreover, TS could upregulate the expression of anti-oxidative stress factors such as SOD and downregulate the expression of oxidative stress factors such as MDA through Nrf2, thereby exerting an anti-oxidative stress effect. Furthermore, it was found that TS could significantly affect the diversity, composition, and abundance of intestinal flora and GM. The results showed that TS could significantly downregulate the abnormally increased abundance of *Helicobacter*, *Mucispirillum*, *Roseburia*, and *Clostridium* in APP/PS1 transgenic mice, indicating that TS may have a protective effect on AD by regulating GM.

Three behavioral experiments were used to investigate the effects of TS on the cognitive function of APP/PS1 mice. The results indicated that TS treatment could significantly improve the cognitive function of APP/PS1 mice. APP/PS1 transgenic mice are an AD mouse model expressing mutations in APP and presenilin genes transcribed under the control of a neuron-specific Thy1 promoter. These two genes can lead to early onset of familial AD by jointly exacerbating Aβ or tau pathology (Dodiya et al., 2019; Soto-Faguas et al., 2021). Based on previous studies, APP/PS1 mice develop brain amyloid lesions at 6–8 weeks of age; Aβ accumulates in the cerebral cortex and hippocampus at 8 months, and hyperphosphorylated tau near Aβ plaques and reduced synaptic density are observed (Kosel et al., 2020). Senile plaques are secreted proteins released by stepwise proteolysis of APP, whereas hyperphosphorylation of tau leads to the formation of NFTs in AD (Lee et al., 2016). Therefore, the APP/PS1 mouse model has been widely used in the study of AD, which is a pathological model with Aβ aggregation and senile plaque deposition, tau hyperphosphorylation, and synaptic dysfunction, which is also consistent with our experimental results. In addition, after antibiotic or bacterial treatment, APP/PS1 mice have reduced Aβ deposition, improved cognitive memory function, and reduced inflammatory levels (Cryan et al., 2019). Jing Sun et al. also used this model to study the reduction of microglia-mediated neuroinflammation by probiotic CB in relation to its metabolite butyric-mediated regulation of the brain–gut axis (Sun et al., 2020). These results indicate that the APP/PS1 mouse model achieves the conditions for GM experiments.

Nrf2 is a transcription factor that primarily exists in the cytosol of hippocampal neurons, regulates many antioxidant enzymes, such as heme oxygenase-1 (HO-1) and quinine oxidoreductase 1 (NQO1), and upregulates proinflammatory cytokines through the ARE pathway (Kobayashi et al., 2016; Bahn et al., 2019; Ren et al., 2020). After oxidative stress, Nrf2 translocates into the nucleus in response to this pathway, Keap1/Nrf2/ARE, initiating the transcription of anti-oxidative stress genes such as SOD, upregulating GSH levels, and downregulating peroxidase MDA levels (Tu et al., 2019; Osama et al., 2020; Toma et al., 2021). Therefore, Nrf2 activation can protect our body from harmful stress by upregulating antioxidant defense, inhibiting inflammation, and maintaining protein homeostasis and play a protective role in AD brain (Ren et al., 2020). Nrf2 levels could decrease with age in postmortem human brain and animal models of AD (Ren et al., 2020; Wei et al., 2020). Significantly reduced levels of Nrf2 were also detected in APP/PS1 mice and Aβ-induced cell models. Recent studies have shown a correlation between Nrf2 deficiency and AD because Nrf2 not only reduces oxidative stress and inflammation but also directly or indirectly regulates autophagy *in vivo* and *in vitro* (Pajares et al., 2016; Branca et al., 2017; Jan et al., 2017; Rojo et al., 2017; Ren et al., 2020). Transcriptional analysis suggests that the brain of Nrf2 knockout mice replicates the least normal pathway in the human AD brain (Rojo et al., 2017; Wei et al., 2020). Subsequently, some studies have found that Nrf2 knockout exacerbates cognitive deficits in mouse models of AD, induces multiple stress responses, and aggravates AD pathologies (Branca et al., 2017; Rojo et al., 2017; Ren et al., 2020). This result indicates that Nrf2 can be activated through the genetic or pharmacological pathways to play a neuroprotective effect.

APP is a single-pass transmembrane protein expressed in high levels in the brain, and three enzymes are responsible for cleaving APP: α-secretase, BACE1, and γ-secretase (Lee et al., 2016). Aβ, a derivative of APP, is primarily produced by sequential cleavage of BACE1 and γ-secretase, and deposited in the brains of AD patients in the form of plaques (Lee et al., 2016; Jiang et al., 2020; Han et al., 2021; Ledo et al., 2021). Increased concentration and activity of BACE1 was also observed in AD brain and body fluids (Lee et al., 2016; Hampel et al., 2021). Some ingredients of traditional Chinese medicine have been found to show effects on BACE1. Ginsenosides can reduce the activity and expression of BACE1, but have no effect on the levels of total APP and SAPPα (Cao et al., 2016; Singh et al., 2021). Baicalein shows potent anti-BACE1 activity and can inhibit Aβ-induced PC12 cytotoxicity (Singh et al., 2021). Thus, the effect of TS on BACE1 expression was investigated. Aβ is a polypeptide composed of 39–42 amino acids, and the most common Aβ fragments contain 40 or 42 amino acids. The levels of soluble and insoluble Aβ40 and Aβ42 in brain tissue were examined (Lee et al., 2016; Fao et al., 2019; Kwak et al., 2020). It was found that high doses of TS significantly reduced the levels of soluble and insoluble Aβ40 and Aβ42 in the brain, whereas different doses of TS significantly reduced the deposition of senile plaques in APP/PS1 mice. It was also found that TS could effectively inhibit Aβ aggregation and diminish cytotoxicity in PC12 cells after Aβ-induced *in vitro* studies by ThT and MTT assays. The abovementioned results indicated that TS treatment significantly reduced Aβ generation and aggregation in AD mice and cells. In the mechanism study, it was found that TS reduced BACE1 expression in APP/PS1 mice *in vivo* and in Aβ-induced PC12 cells *in vitro*, indicating that TS inhibits Aβ formation by reducing BACE1 expression. The results were consistent with previous report, which found that treatment with therapeutic drugs significantly inhibited the expression of BACE1 and reduced the release of SAPPβ in APPswe/PS1ΔE9 mice, but it did not change the expression of full-length APP, PS1, a disintegrin, metalloproteinase 10 (ADAM10), and other cleavage products, including SAPPα, neprilysin, and insulin-degrading enzyme in APPswe/PS1dE9 mice (Wei et al., 2020). Therefore, Aβ was primarily reduced by inhibiting β-secretase-induced amyloid generation but not by increasing non-amyloid production.

Recently, Nrf2 was found to negatively regulate BACE1 expression and improve cognitive deficits in mouse models of AD (Bahn et al., 2019; Ren et al., 2020). Bahn et al. revealed a previously unknown molecular mechanism, in which Nrf2-deficient AD mice had significantly increased BACE1 and BACE1-AS expression and Aβ deposition, and more severe cognitive dysfunction compared with 5xFAD and Nrf2 knockout mice, whereas the activation of Nrf2 inhibited BACE1 and BACE1-AS expression and Aβ production and improved cognitive dysfunction and AD-related pathological characteristics (Bahn et al., 2019). Thus, it was hypothesized whether TS could inhibit BACE1 expression by activating Nrf2, thereby reducing Aβ formation. Subsequently, the changes of Nrf2 and BACE1 and the relationship between them after TS treatment were investigated. The results showed that TS could reduce the expression of BACE1 by activating Nrf2, and when Nrf2 was knocked down, the effect of TS on inhibiting BACE1 expression was attenuated, which indicated that TS negatively regulated the expression of BACE1 by activating Nrf2, thereby inhibiting the production and deposition of Aβ. Moreover, using CHIP assay, it was found that Nrf2 could directly bind to the ARE1 site in the BACE1 promoter, and TS could promote the binding of Nrf2 and BACE1 promoters to inhibit the transcription of BACE1.

Tau primarily accumulates in the medial temporal lobe, and its main function is to bind and stabilize microtubules, which are a major component of the neuronal cytoskeleton, providing structural support to neurons, and binding is regulated by its phosphorylation state (Lee et al., 2016; Maass et al., 2018; Lowe et al., 2019; Kosel et al., 2020; Kwak et al., 2020; Limorenko & Lashuel, 2022). In AD, abnormally phosphorylated tau detaches from microtubules and begins to accumulate with other tau filaments (Lee et al., 2016). This process has two stages: paired helical filament (PHF) stage, in which these aggregated tau filaments form PHF, and neurofibrillary tangle (NFT) stage, in which the formed PHF winds around each other to form insoluble double-fiber NFT in the cell and eventually accumulates in the form of NFT (Jo et al., 2014; Lee et al., 2016; Limorenko & Lashuel, 2022). In this process, microtubule disassembly can cause the collapse of neurons, impairing the ability of neurons to communicate with one another (Jo et al., 2014; Lee et al., 2016). Among the multiple phosphorylation sites associated with AD, tau is phosphorylated at Thr231 and Ser396 during the PHF stage, whereas it is primarily phosphorylated at Ser396 during the NFT stage. In addition, Aβ can induce tau phosphorylation at Thr231 and Ser396 (Lee et al., 2016). Collectively, tau is primarily phosphorylated at Ser396, and it belongs to the C-terminal domain, which plays a key role in regulating tau aggregation (Jo et al., 2014). Thus, the effect of TS on *p-*tau (Ser396) level were examined. The results showed that TS could reduce the accumulation of *p-*tau (Ser396) and reduce the level of *p-*tau (Ser396) in the hippocampus and cerebral cortex of APP/PS1 mice. In the study of Saikosaponin C (SSc) by Lee et al., they found that SSc had an inhibitory effect on *p-*tau but not on the expression of total Tau (Tau5) (Lee et al., 2016). Combined with their study, TS and its monomeric saponin SSc can reduce *p-*tau level in AD brain.

At present, increasing evidence shows that impaired autophagy is closely related to the pathogenesis of AD. The researchers found that autophagosomes and lysosome accumulated in the brains of AD patients by electron microscopy and that autophagic flux was inhibited (Zhang et al., 2021). In addition, autophagy plays an important role in tau pathology. Data have shown that once autophagic flux is blocked, the clearance of Tau is affected, and insoluble tau aggregates accumulate remarkably (Zhang et al., 2021). In the brains of AD patients, hyperphosphorylated tau colocalized with the autophagic marker LC3 and autophagic receptor p62, which was not observed in controls (Piras et al., 2016; Zhang et al., 2021). The turnover of LC3 is an essential process during autophagic activation, in which LC3-I is modified by phosphatidyl ethanolamine and converted to LC3-II (Jiang et al., 2018). Autophagic receptors interact with LC3, including p62, NBR1, and NDP52, of which p62 and NDP52 are the most studied. They can direct autophagic targets to autophagosomes, which then fuse cargo-containing autophagosomes with lysosomal vesicles and promote the degradation of autophagosomes (Jo et al., 2014; Menzies et al., 2017; Viret et al., 2018). Based on previous results, the downregulation of p62 expression is induced by Nrf2 activation, and the accumulation of hyperphosphorylated tau is observed in p62 knockout mice, and few studies have investigated the effect of NDP52 on tau (Jo et al., 2014). Jo et al. showed that compared with p62, their data strongly indicated that NDP52 may be the main autophagy adaptor that promotes phosphorylated tau degradation (Jo et al., 2014). Based on the abovementioned findings, whether the reduction of *p-*tau level by TS was through the autophagy of p62 and NDP52 was investigated. The changes in the expression levels of autophagic proteins such as p62 and NDP52 and the changes in *p-*tau in APP/PS1 mice and Aβ-induced PC12 cells after TS treatment were examined. The results showed that after TS treatment, the expression of LC3-II and Beclin-1 increased, and the expression of NDP52 and p62 decreased in AD mice and cells, indicating that the autophagic flux increased, whereas the level of *p-*tau significantly decreased; after subsequent administration of the autophagic degradation inhibitor CQ, the levels of NDP52 and p62 increased, and the clearance of *p-*tau decreased, indicating that TS upregulated the expression of autophagy-related proteins such as NDP52 and p62 by promoting autophagy and promoted the formation and degradation of autophagosomes, thereby promoting the clearance of *p-*tau.

Transcription factor EB (TFEB) is also an important autophagic factor, which is highly expressed in the central nervous system and active in neurons and astrocytes (Bao et al., 2016; Sachchida Nand Rai, 2021). TFEB can regulate the expression of genes involved in the autophagy-lysosome pathway (Puertollano et al., 2018; La Spina et al., 2020), and regulate autophagy flux by promoting the occurrence of lysosomes and autophagosomes and regulating autophagy body-lysosome fusion, thus promoting autophagic clearance (Sachchida Nand Rai, 2021). Evidences revealed that TFEB could effectively reduce the nerve fiber entanglement and the level of *p-*tau, thus ameliorating cognitive dysfunction (Wang H. et al, 2016; Sardiello, 2016; Sachchida Nand Rai, 2021). Therefore, TFEB has become a potential therapeutic target for the development of effective drugs. MTOR complex 1 (mTORC1) can negatively regulate TFEB, promote the production of *p-*TFEB and inhibit its nuclear translocation, thus hinder the process of autophagy degradation (Saftig & Haas, 2016; Napolitano et al., 2020). In this study, after treatment with TS, we observed a decrease in the levels of *p-*TFEB and mTOR, indicating that TS can inhibit the expression of mTOR, reduce the production of *p-*TFEB, and promote the nuclear translocation of TFEB to regulate autophagosome-lysosome fusion. After administration of Rapa, the levels of *p-*TFEB, mTOR and *p-*tau decreased, indicating that TS down-regulated the expression of *p-*TFEB and mTOR, accelerated autophagy degradation by promoting autophagosome-lysosome fusion, thus boosting the clearance of *p-*tau.

Oxidative stress can lead to a severe imbalance between the production of ROS and reactive nitrogen species and antioxidant defenses (Butterfield & Halliwell, 2019). Extensive studies have shown that oxidative stress has a great impact on the pathogenesis and progression of AD, and the relationship between oxidative stress and AD neurodegeneration has been widely reported (Cheignon et al., 2018; Butterfield & Halliwell, 2019; Wei et al., 2020). The indicators which were examined to represent changes in oxidative stress included MDA, SOD, GSH, GSSG, and the ratio of GSH/GSSG. MDA is a major aldehyde produced by the peroxidation decomposition of unsaturated fatty acids, a studied indicator of the degrees of lipid peroxidation, reflecting the overproduction of ROS and the ability to surpass the endogenous antioxidant defense system (Liu et al., 2020; Pau et al., 2021). In addition, the antioxidant enzymes SOD and GSH and GSSG play an important role in regulating the balance of the oxidative stress system. SOD, an important free radical scavenging enzyme in organisms, is the first line of defense against oxidative stress, which can catalyze O_2_ disproportionation reaction to eliminate oxygen free radicals produced in the body, thereby protecting cells from oxygen free radicals (Balamurugan et al., 2018; Liu et al., 2020). In addition, SOD and MDA can verify the oxidative stress status of cells, and the imbalance between them often leads to pathological factors of neurodegenerative diseases (Liu et al., 2020). Moreover, glutathione peroxidase (GSH) is widely present in the human body, and it specifically catalyzes the reduction of hydrogen peroxide by reducing glutathione to protect the structure and function of cell membrane (Liu et al., 2020). In addition, the ratio of GSSG/GSH is a good marker of oxidative stress, depletion of GSH content, increase of GSSG content, and imbalance of GSH/GSSG ratio in AD patients and animal models (Vida et al., 2017). Our experimental results demonstrated that TS could decrease the content of MDA and GSSG, increase the content of SOD and GSH, and the ratio of GSH/GSSG, indicating that TS could inhibit the oxidative stress state in APP/PS1 mice. The regulation of oxidative stress is closely related to the function of mitochondria. Mitochondrial dysfunction plays an important role in the AD pathogenesis (Rai et al., 2020). Evidence shows that in the pathogenesis of AD, excessive production of ROS leads to oxidative stress, which seriously impairs the function of mitochondria and leads to neuronal damage (Rai et al., 2020). The effect of TS on mitochondrial function was not investigated in this study, which is needed to be further elucidated.

Neuroinflammation processes can produce proinflammatory cytokines (including IL-1β, IL-6, IL-1β, and TNF-α), and the innate immune cells involved in the process primarily include microglia and astrocytes (Leng & Edison, 2021). The release of proinflammatory molecules may lead to synaptic dysfunction, neuronal death, and inhibition of neurogenesis. For example, IL-1β leads to synaptic loss by increasing prostaglandin E2 production, when the NF-κB pathway is inhibited, TNF-α leads to neuronal death by activating tumor necrosis factor receptor 1 (TNFR 1) (Leng & Edison, 2021). Therefore, the effects of TS on the activation of microglia and astrocytes and the release of proinflammatory cytokines were examined. It was found that TS could reduce the levels of Iba-1 and GFAP and the activation of microglia and astrocytes, thereby reducing the levels of TNF-α, IL-1β, IL-1, and the release of proinflammatory factors in APP/PS1 mice to inhibit neuroinflammation.

The abovementioned results illustrate that TS can inhibit glial activation and neuroinflammation in APP/PS1 transgenic mice, and the underlying mechanism was further investigated. It has been shown that Aβ can activate the NF-κB pathway in astrocytes, which can act on receptors on neurons and microglia, leading to neuronal dysfunction and microglial activation (Leng & Edison, 2021). Numerous studies have shown an interaction between Nrf2 and NF-κB signaling pathways, which regulate the main pathway of inflammatory response (Wardyn et al., 2015; Tom et al., 2019; Sorrenti et al., 2020). The effect of Nrf2 is related to its ability to antagonize NF-κB, and the activation of Nrf2 can inhibit the NF-κB pathway. For example, the Nrf2 agonist SFN can reduce the activity of NF-κB (Sorrenti et al., 2020). Thus, the NF-κB signaling pathway was selected to investigate the effects of TS on inflammatory factors and glial activation to improve the mechanism of TS. It was found that TS treatment significantly inhibited the expression of NF-κB in APP/PS1 mice and Aβ-induced PC12 cells by *in vivo* and *in vitro* experiments. However, in Nrf2 knockout PC12 cells, the transcriptional effect of TS inhibition of NF-κB was weakened, demonstrating that TS improves neuroinflammation by acting on Nrf2 to downregulate the expression of NF-κB.

Synapses, the junctions between two neurons, are important structures for maintaining the normal functional network of neurons, and they are considered as pathologically relevant factors for cognitive decline (Lee et al., 2016; Wei et al., 2020). The presynaptic vesicle protein synaptophysin and postsynaptic protein PSD-95 play crucial roles in synaptic transmission, synaptic maturation, and synaptic plasticity, and if the molecular network between synapses controls the transmission of synaptic signals and synaptic plasticity, then disturbances in synaptic function may lead to long-term neuronal damage and cognitive decline (Lee et al., 2016). In our results, TS increased the expression of synaptophysin and PSD-95 in APP/PS1 mice and alleviated synaptic dysfunction. Similarly, Chao Wei and Lee et al. found that SSc treatment could increase the protein levels of synaptophysin and PSD-95, thus enhancing synaptic integrity (Lee et al., 2016; Wei et al., 2020). Collectively, TS and its monomeric saponin SSc could protect synaptic function via upregulating the expression of synaptophysin and PSD-95.

Considerable evidence shows that AD is closely related to GM. Clinical data and animal experimental studies show intestinal flora imbalance in AD patients and models, which can promote neuroinflammation and amyloidosis, thereby playing a role in the occurrence and development of AD. Moreover, various drugs have been proved to achieve therapeutic effects through flora, such as probiotics, antibiotics and GV-971. In order to comprehensively evaluate the protective effect of TS, we applied 16s rRNA high-throughput sequencing to analyze the structural diversity, composition, and abundance of GM in WT mice, APP/PS1 transgenic mice, and TS intervention mice. Our results showed that the abundance of *Desuflovibrio*, *Helicobacter*, *Mucispirillum*, *Roseburia*, and *Clostridium* in APP/PS1 mice was significantly higher than that in WT mice, which was partly consistent with previous research. Furthermore, we found that TS treatment reversed the abnormal upregulated abundance of these flora, indicating that TS may play a protective role in AD by regulating GM. Further investigation are needed to verify how TS exerts therapeutic effect against AD through GM. Existing studies have shown a close relationship between GM and inflammation (Giau et al., 2018; Garcez et al., 2019; Wang et al., 2019; Sun et al., 2020). Therefore, whether TS can reduce the inflammatory response by regulating GM is a reasonable research direction.

In conclusion, TS plays a comprehensive role in the treatment of AD through the abovementioned ways, which can effectively ameliorate the cognitive impairment in AD mice. How TS plays a protective role by regulating the GM need to be further investigated. Whether GM affect the release of inflammatory factors or the deposition of Aβ through their metabolites, such as short-chain fatty acids, may be the next research direction in the future.

## Data Availability

The original contributions presented in the study are publicly available. This data can be found here: https://www.ncbi.nlm.nih.gov/, PRJNA859675.
