# Supplementary figures and images for: Therapeutic effects of total saikosaponins from Radix bupleuri against Alzheimer’s disease (part 1 of 2)
Source: Front Pharmacol. 2022 Jul 21;13:940999. doi: 10.3389/fphar.2022.940999 (PMC9351603; doi:10.3389/fphar.2022.940999)

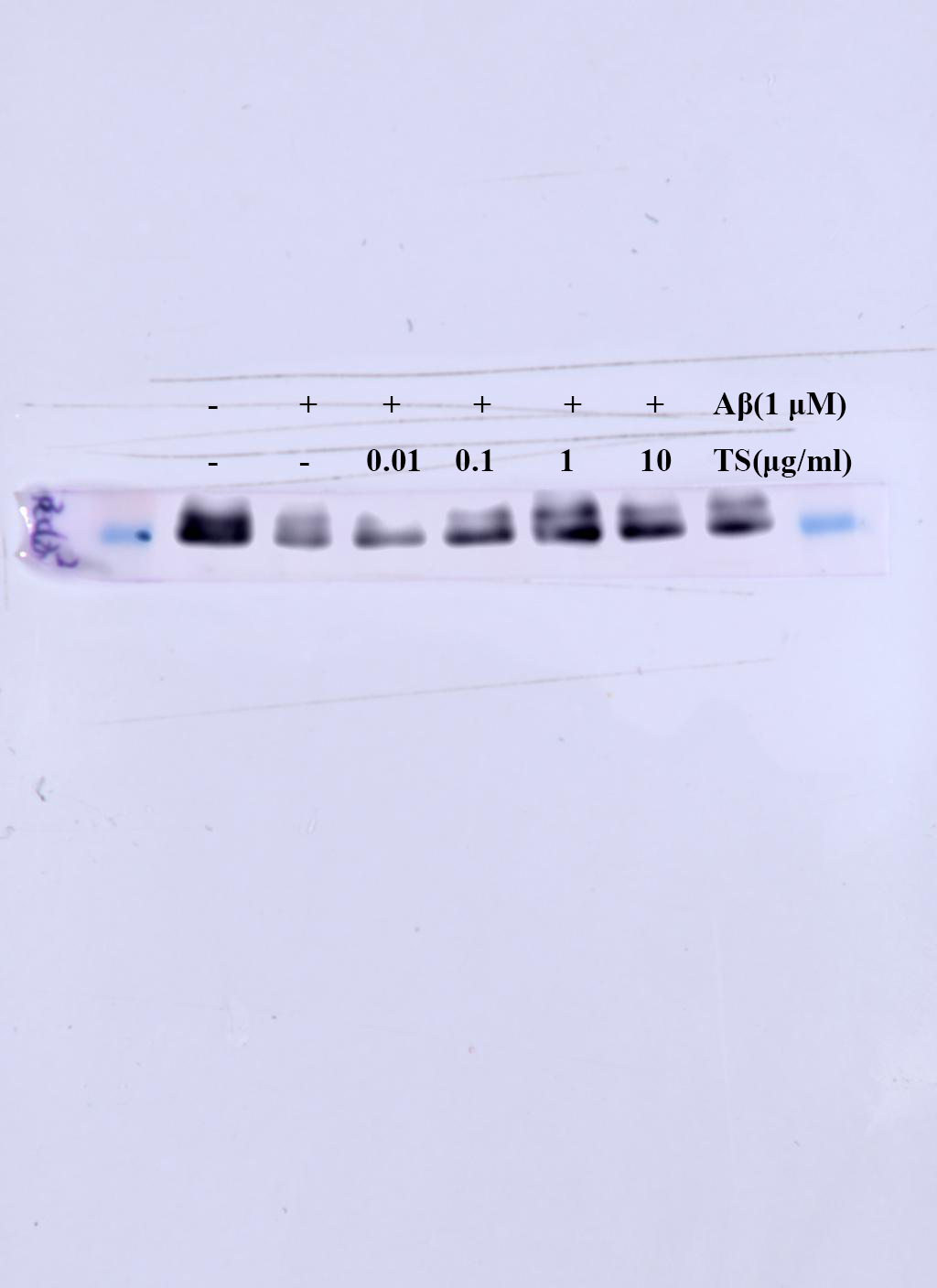

Supplement: Supplementary file 1 [file DataSheet3.ZIP › Fig.6A Beclin/Fig.8A orginal images for quantitative analysis-1.tif]

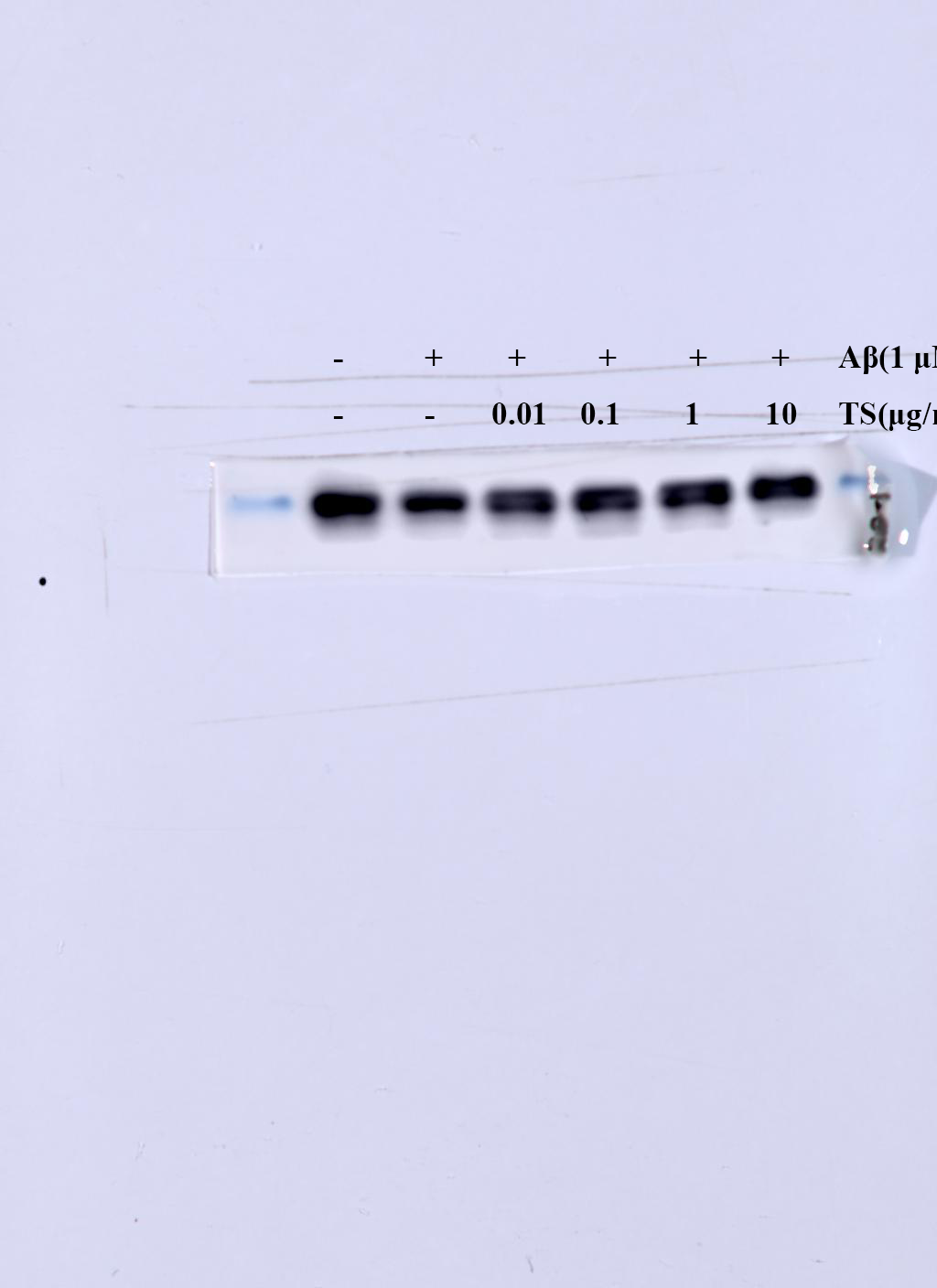

Supplement: Supplementary file 1 [file DataSheet3.ZIP › Fig.6A Beclin/Fig.8A orginal images for quantitative analysis-2.tif]

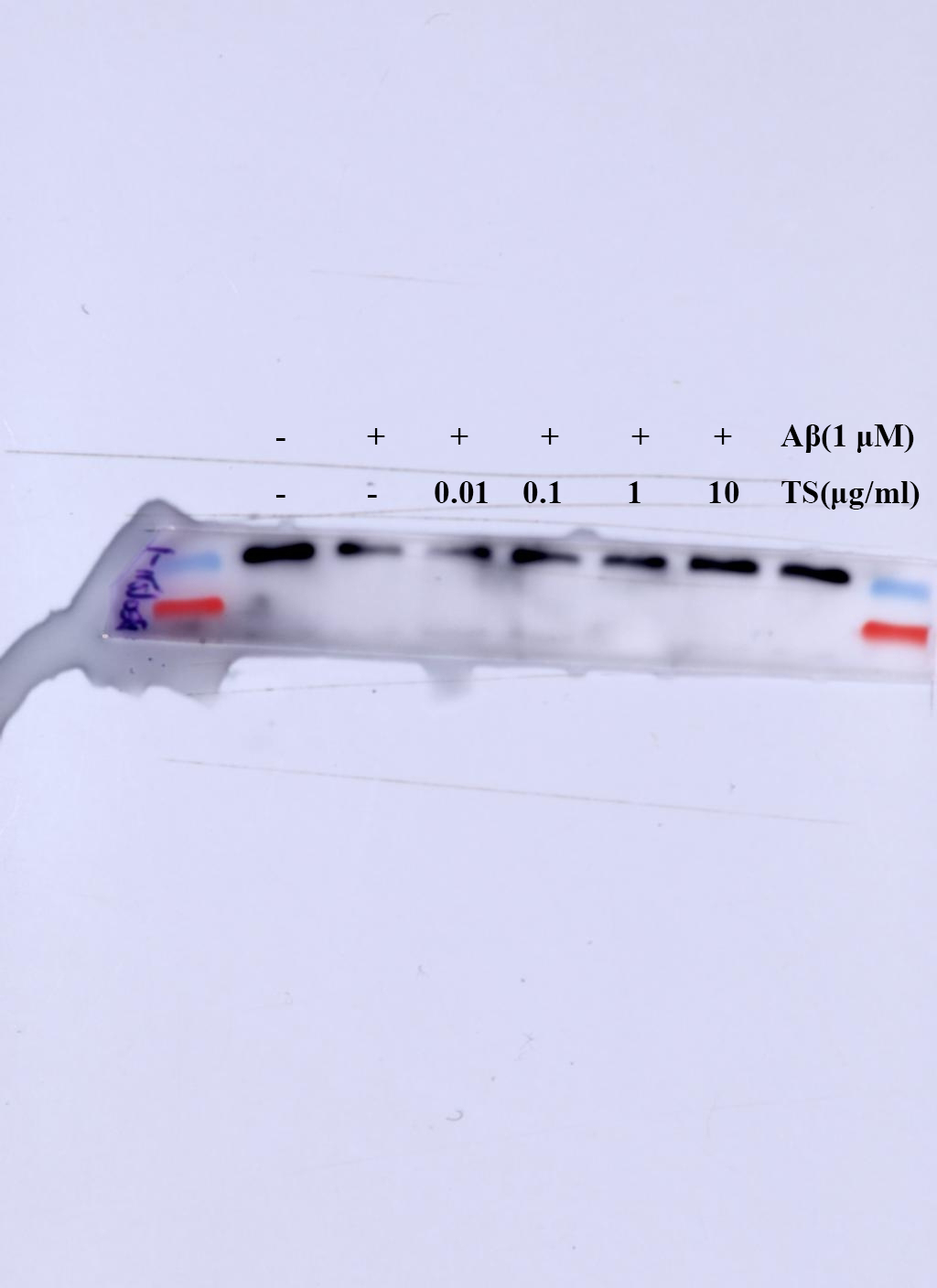

Supplement: Supplementary file 1 [file DataSheet3.ZIP › Fig.6A Beclin/Fig.8A orginal images.tif]

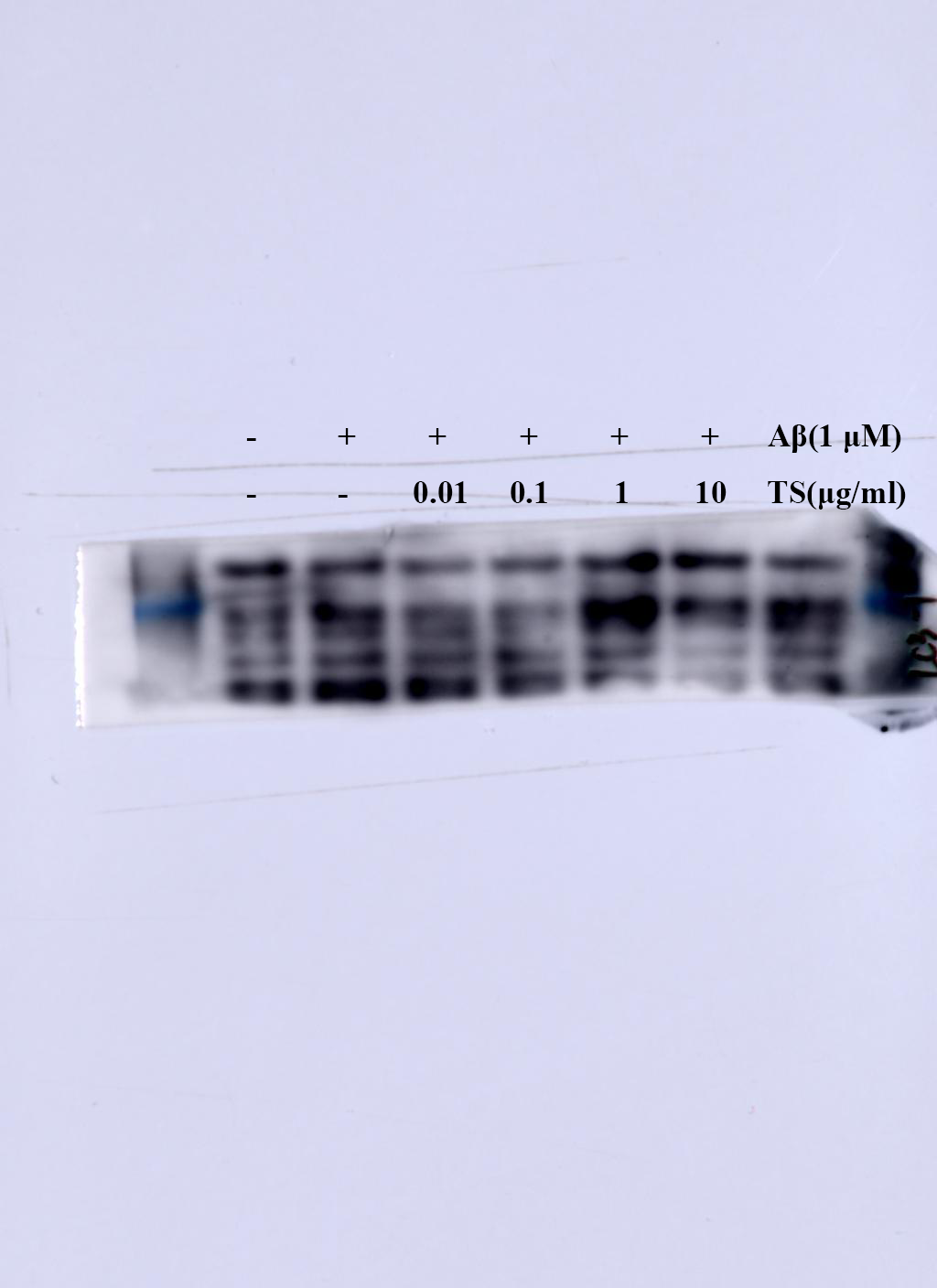

Supplement: Supplementary file 1 [file DataSheet3.ZIP › Fig.6A LC3/Fig.8A orginal image for quantitative analysis-1.tif]

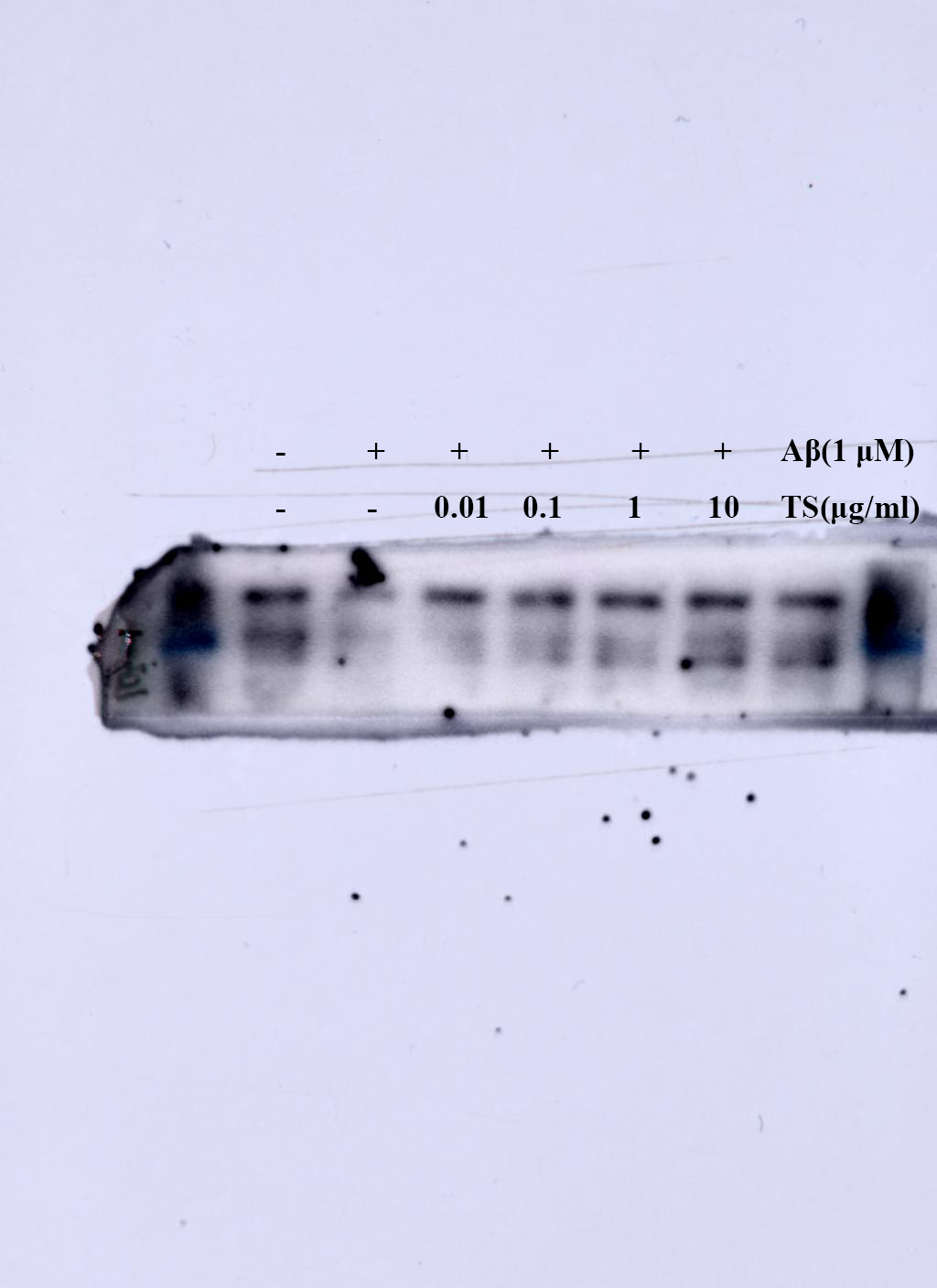

Supplement: Supplementary file 1 [file DataSheet3.ZIP › Fig.6A LC3/Fig.8A orginal image for quantitative analysis-2.tif]

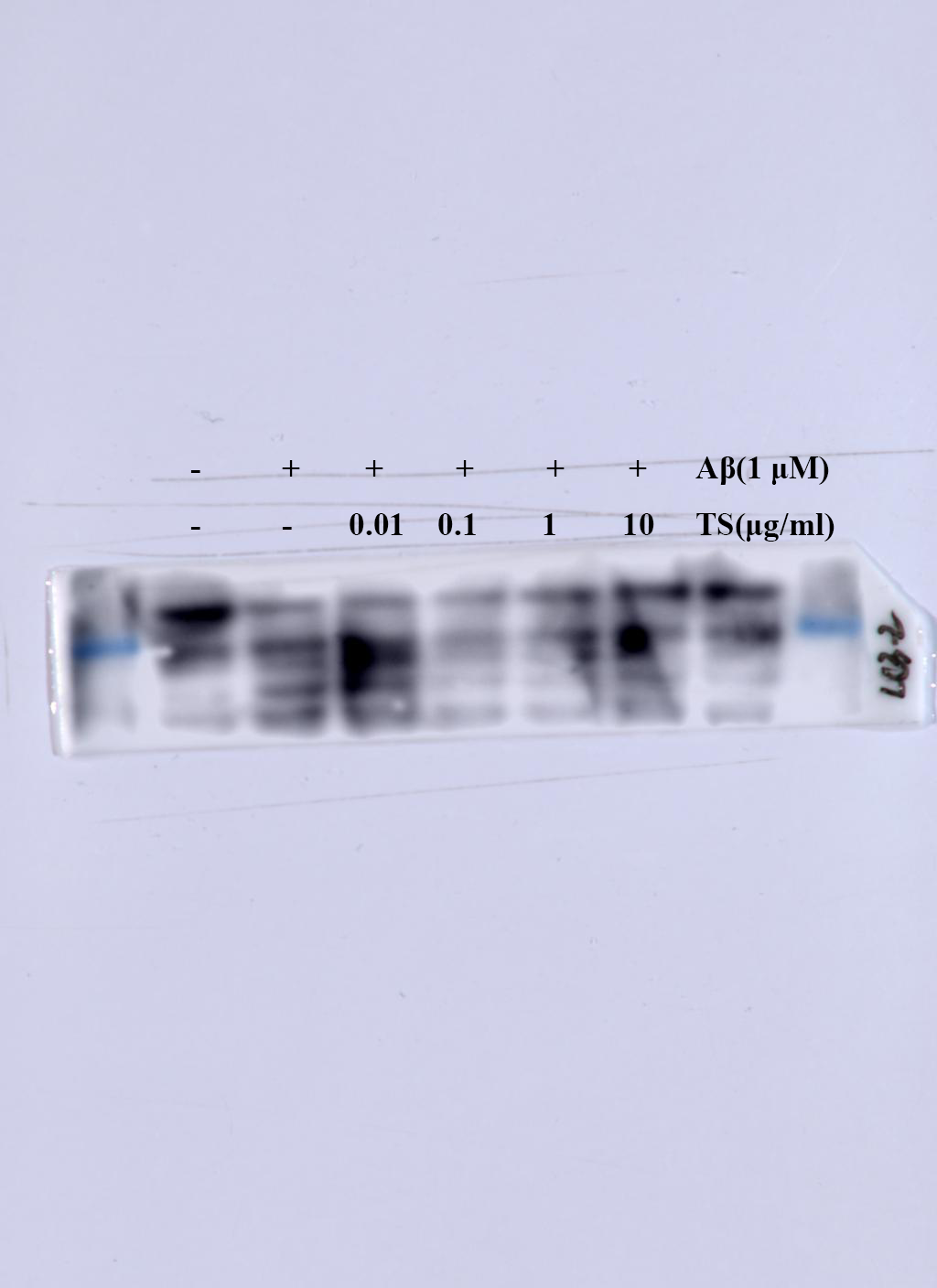

Supplement: Supplementary file 1 [file DataSheet3.ZIP › Fig.6A LC3/Fig.8A orginal image.tif]

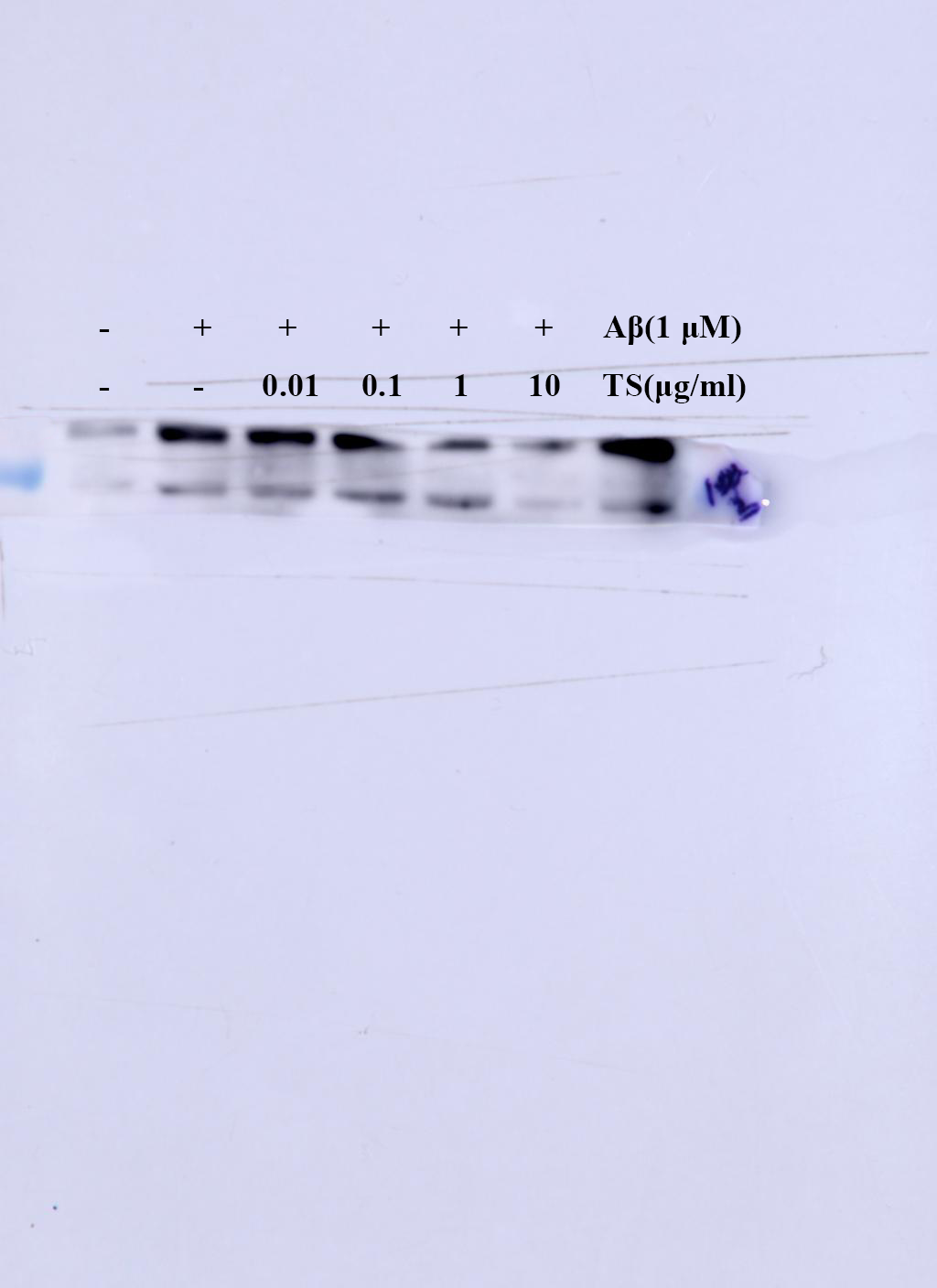

Supplement: Supplementary file 1 [file DataSheet3.ZIP › Fig.6A NDP52/Fig.8A orginal images for quantitative analysis-1.tif]

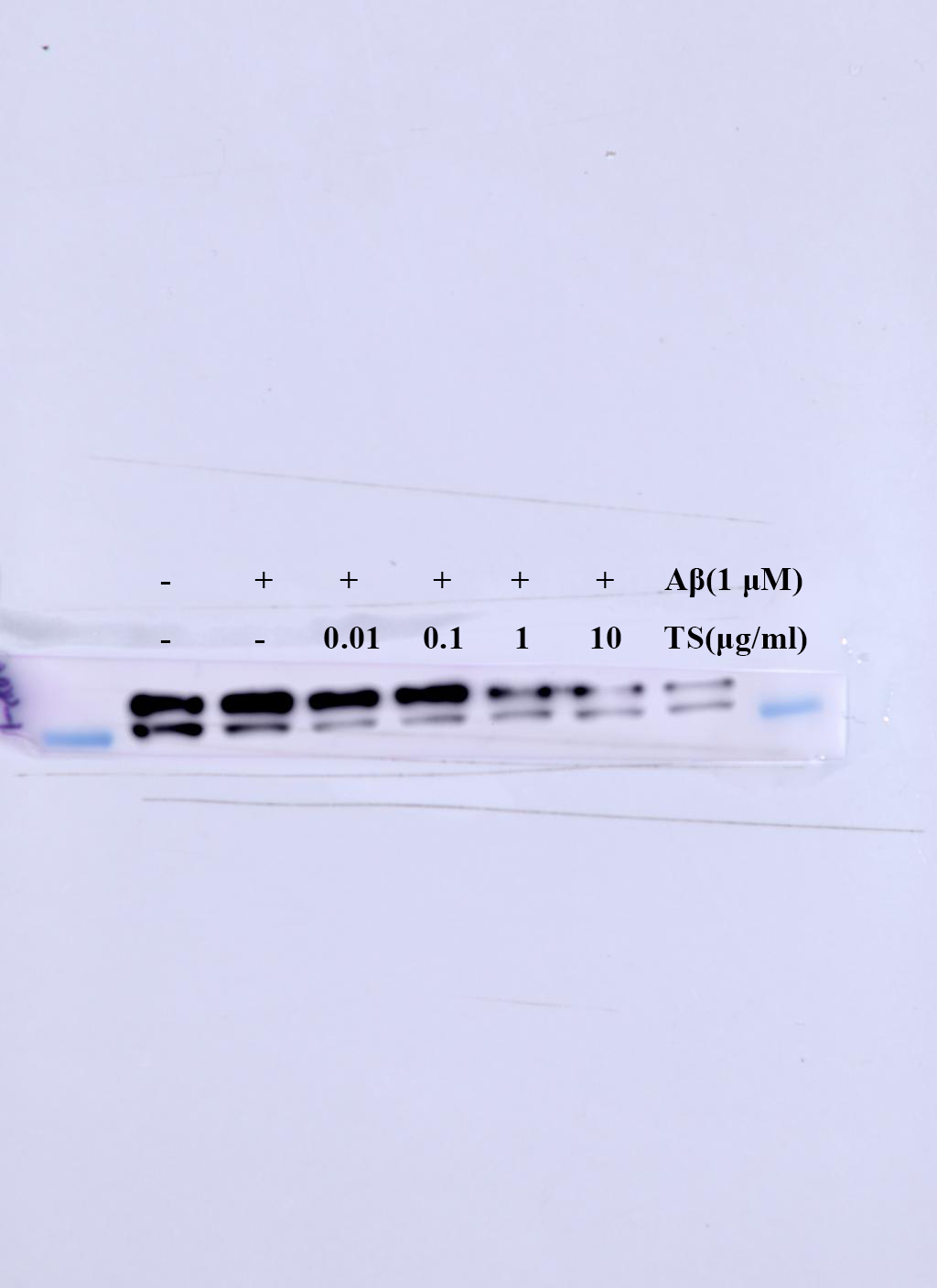

Supplement: Supplementary file 1 [file DataSheet3.ZIP › Fig.6A NDP52/Fig.8A orginal images for quantitative analysis-2.tif]

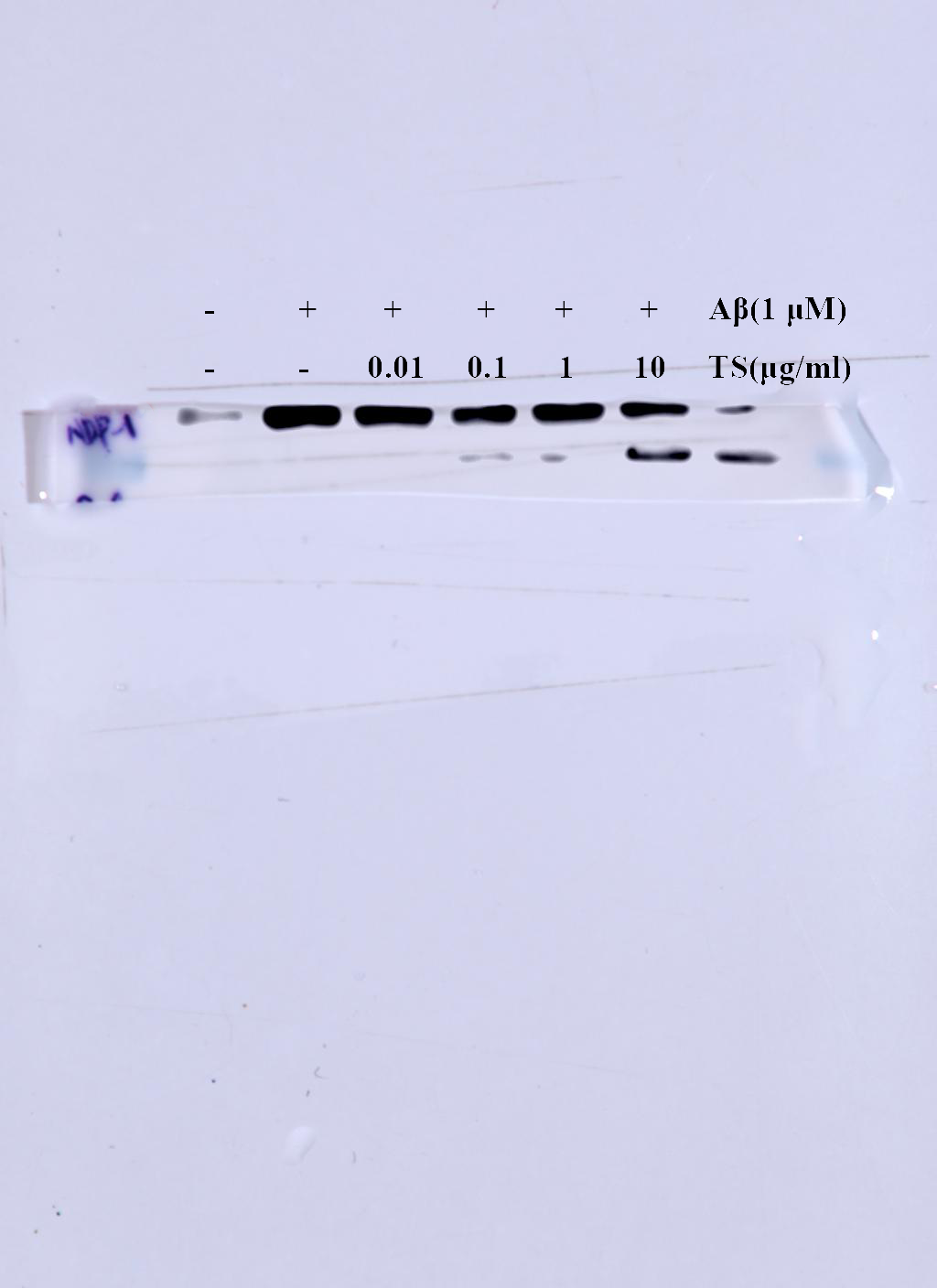

Supplement: Supplementary file 1 [file DataSheet3.ZIP › Fig.6A NDP52/Fig.8A orginal images.tif]

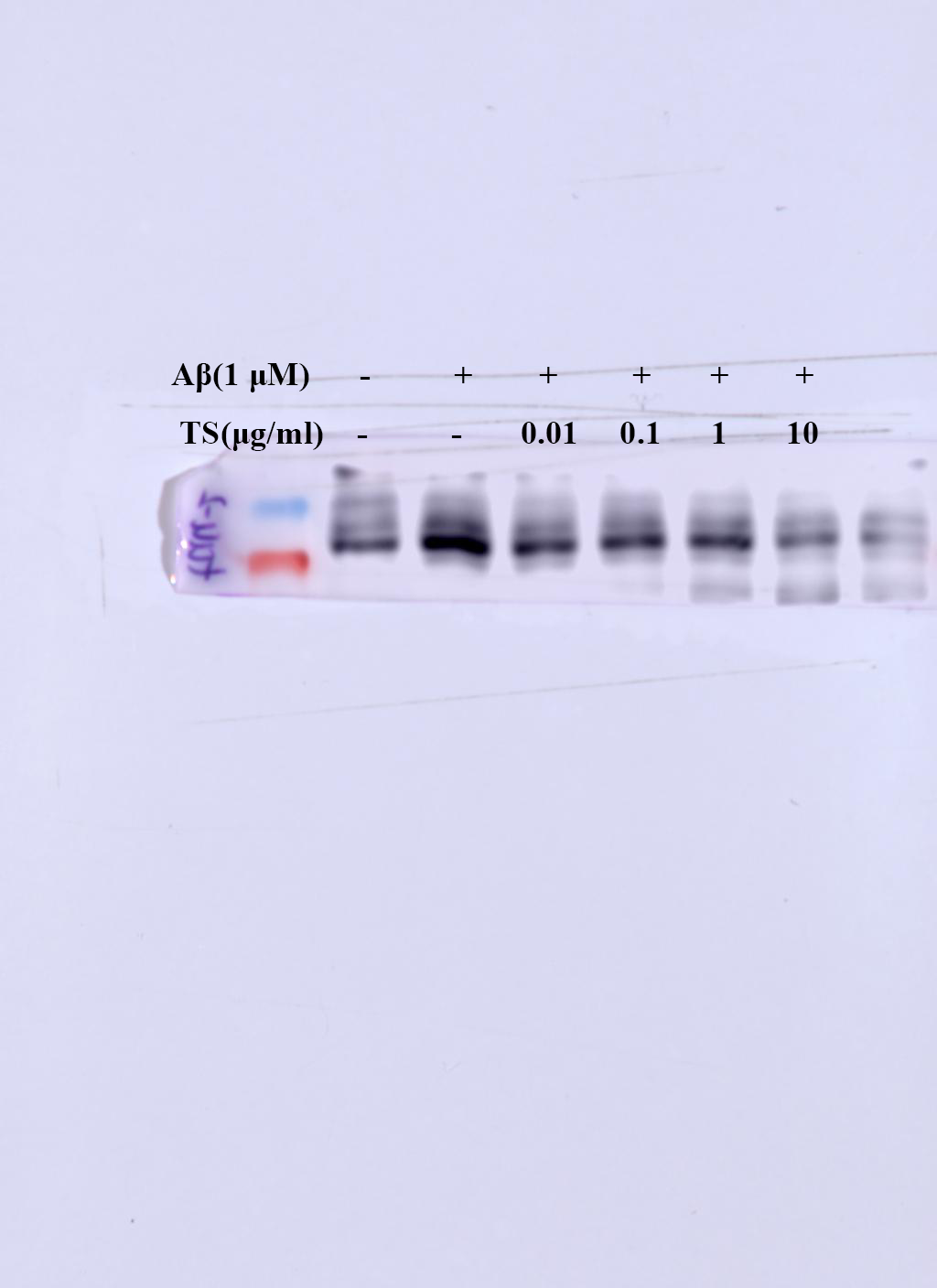

Supplement: Supplementary file 1 [file DataSheet3.ZIP › Fig.6A p-tau/Fig.6A orginal images for quantitative anaylsis-1.tif]

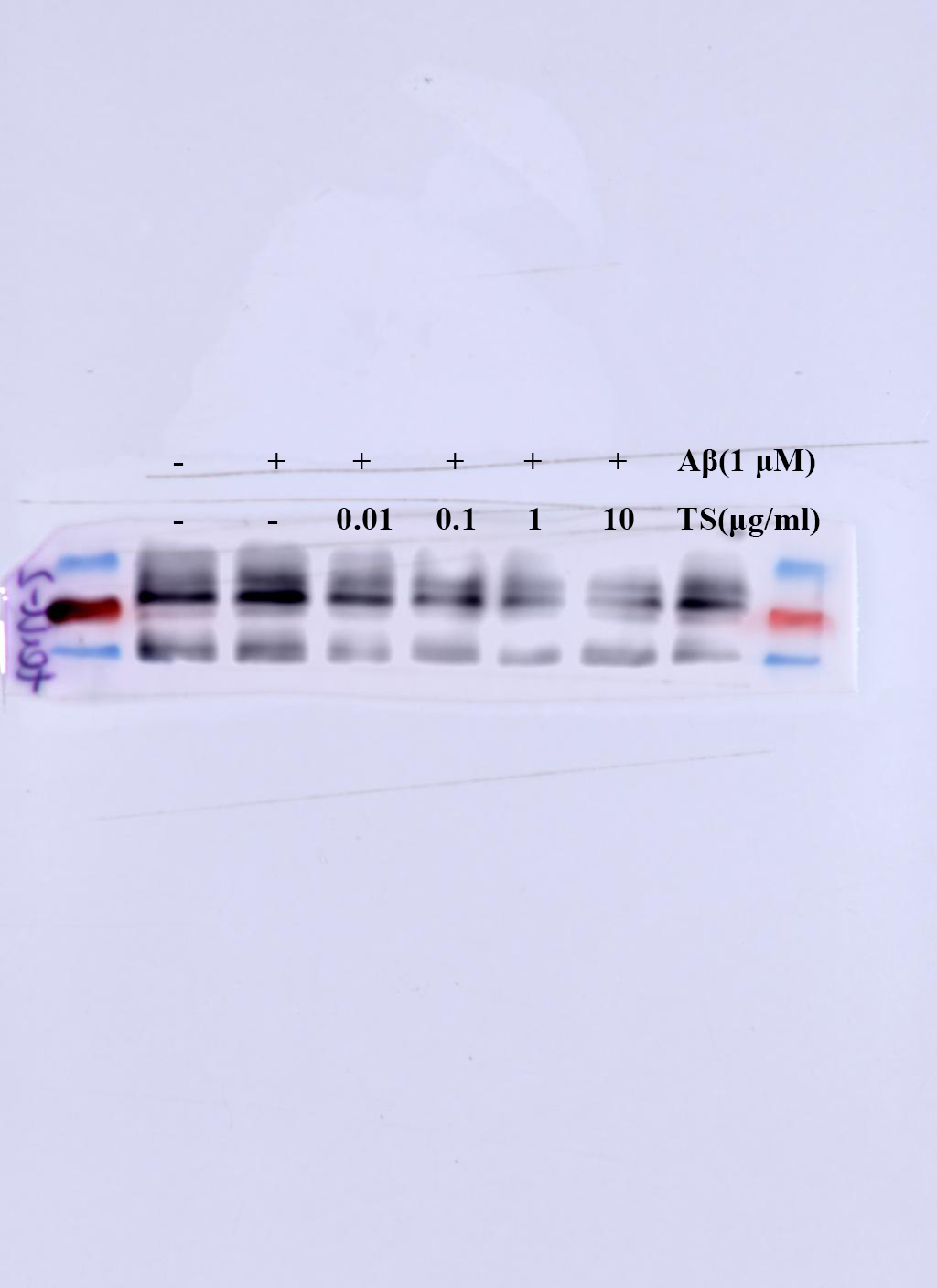

Supplement: Supplementary file 1 [file DataSheet3.ZIP › Fig.6A p-tau/Fig.6A orginal images for quantitative anaylsis-2.tif]

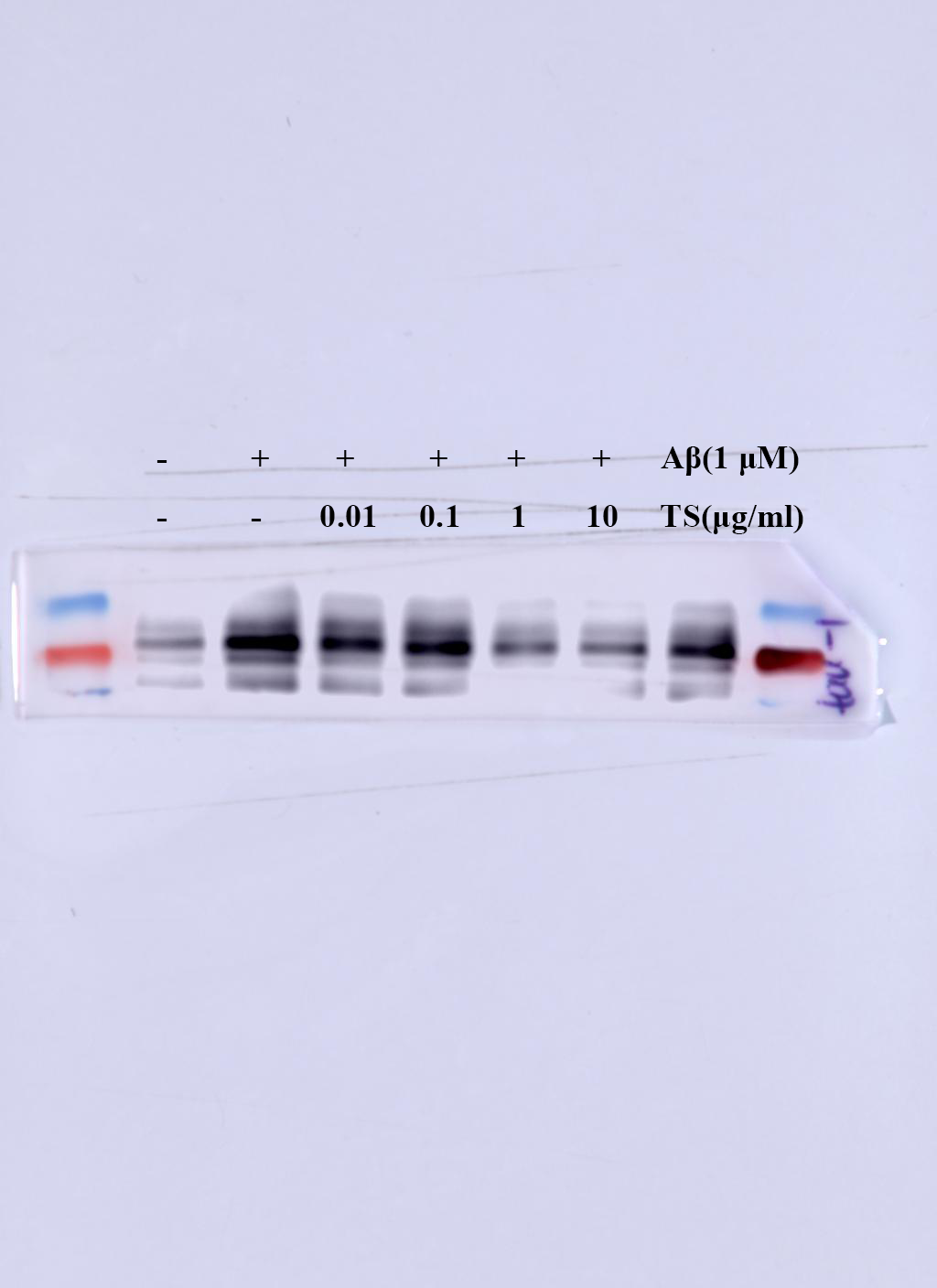

Supplement: Supplementary file 1 [file DataSheet3.ZIP › Fig.6A p-tau/Fig.6A orginal images.tif]

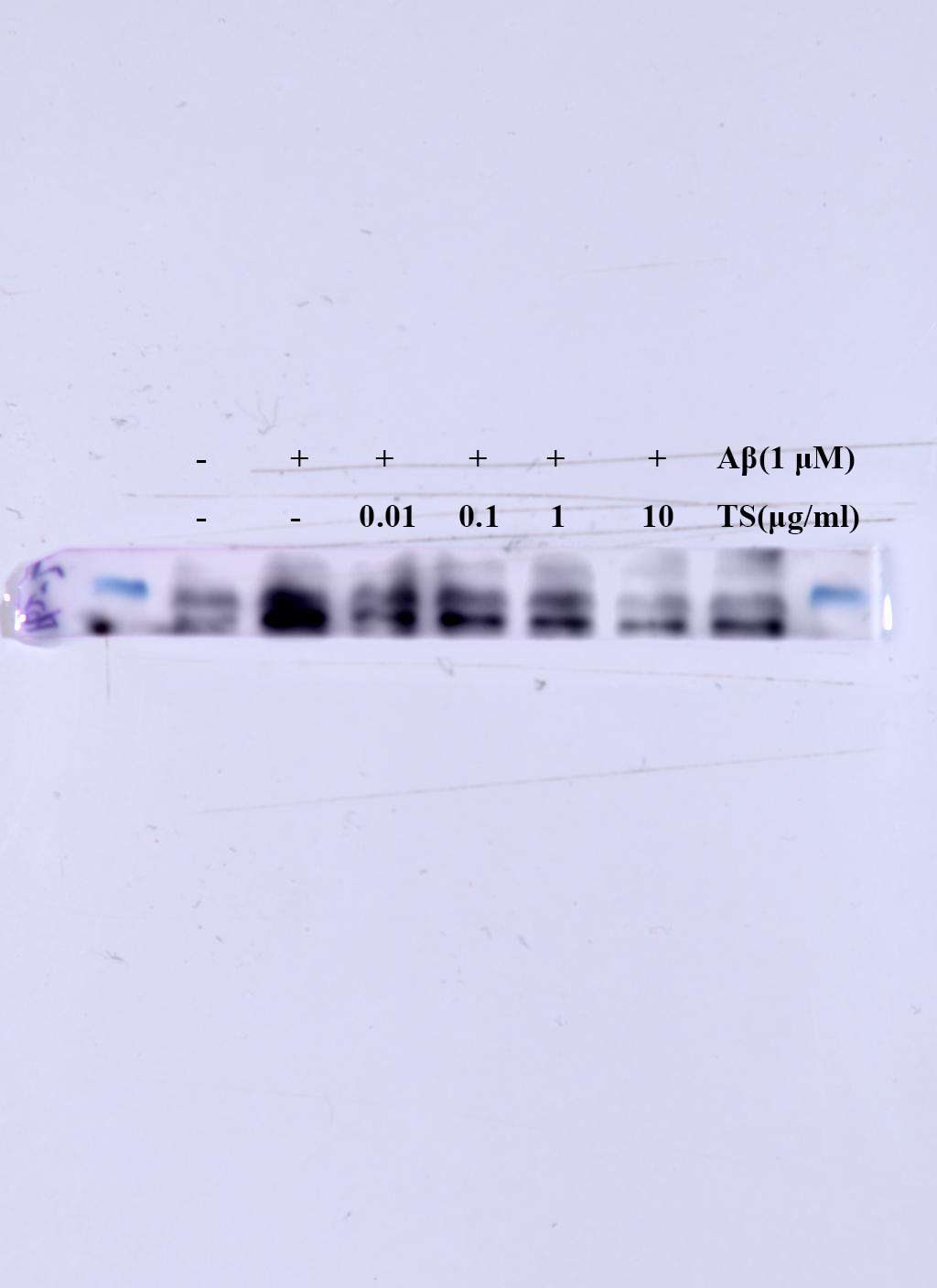

Supplement: Supplementary file 1 [file DataSheet3.ZIP › Fig.6A p62/Fig.8A orginal image for quantitative analysis-1.tif]

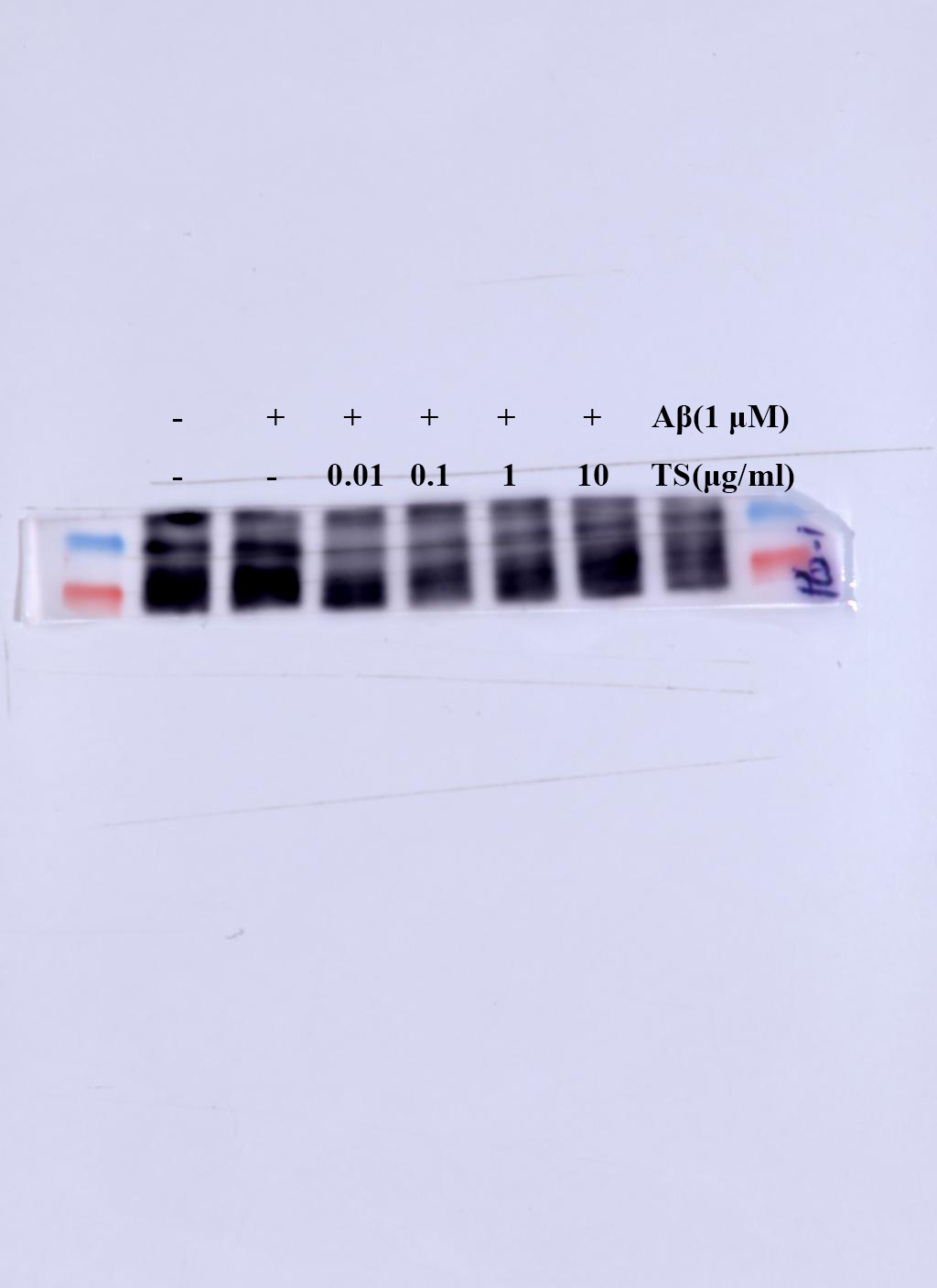

Supplement: Supplementary file 1 [file DataSheet3.ZIP › Fig.6A p62/Fig.8A orginal image for quantitative analysis-2.tif]

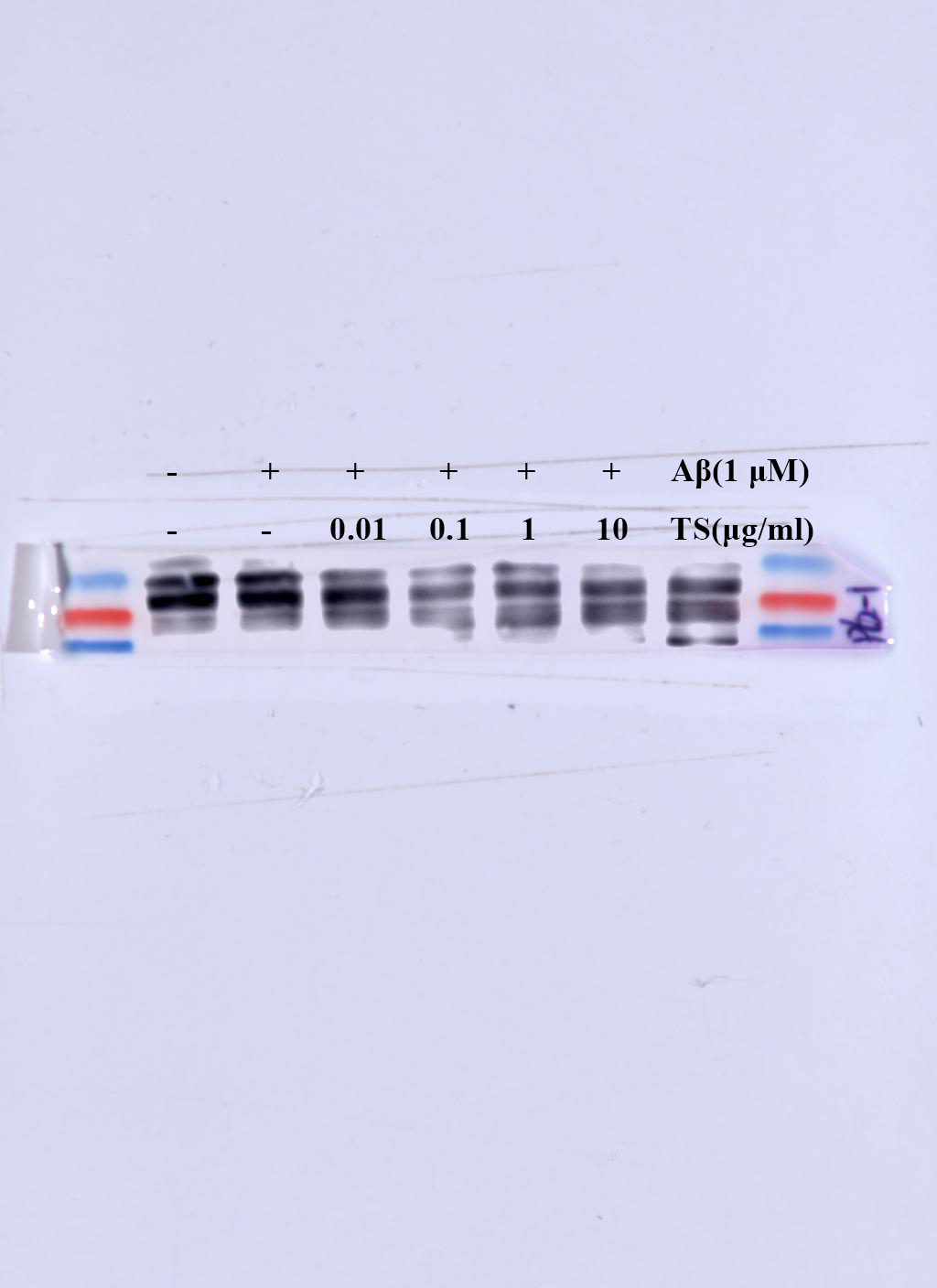

Supplement: Supplementary file 1 [file DataSheet3.ZIP › Fig.6A p62/Fig.8A orginal image for quantitative analysis-3.tif]

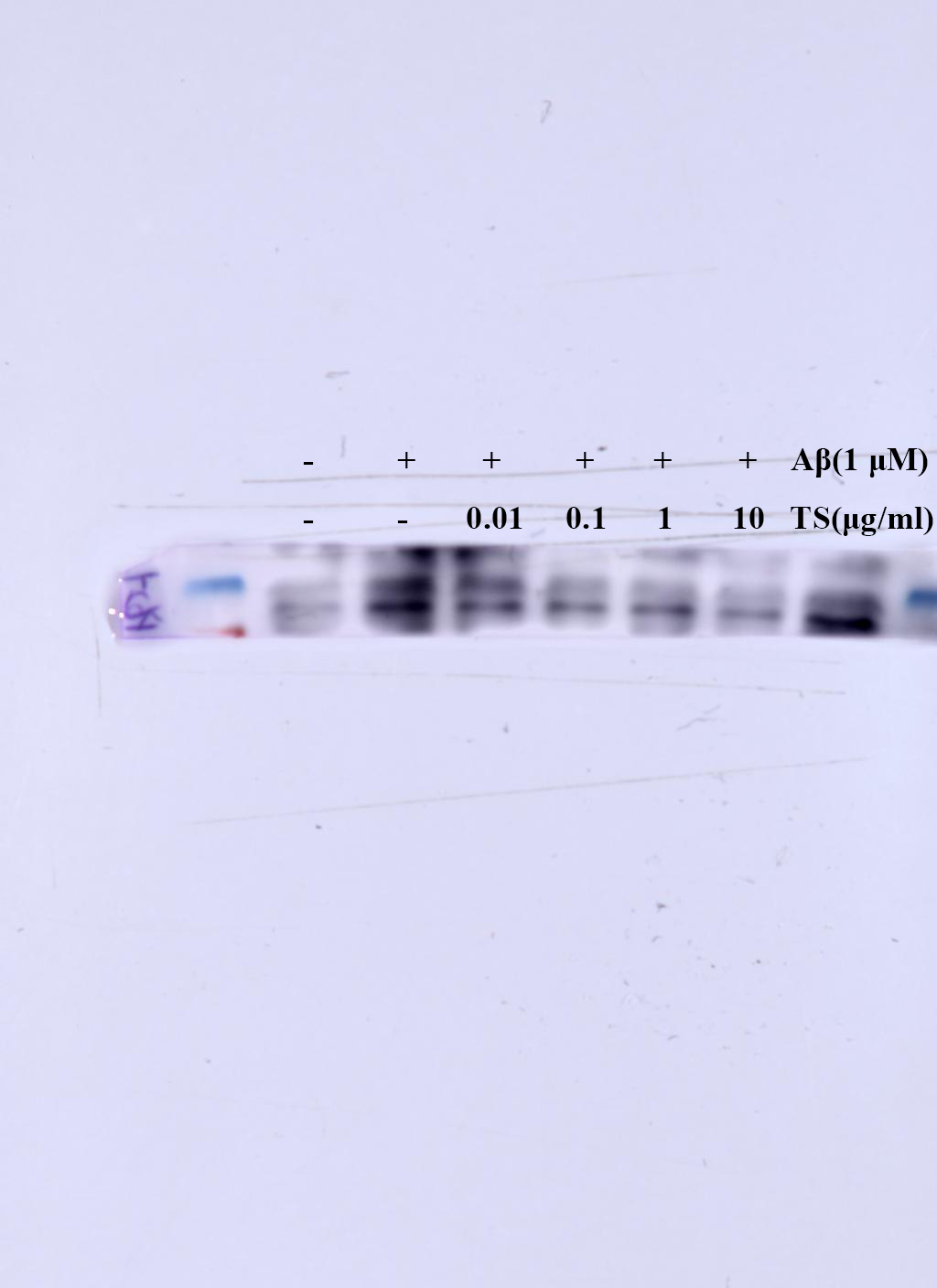

Supplement: Supplementary file 1 [file DataSheet3.ZIP › Fig.6A p62/Fig.8A orginal image.tif]

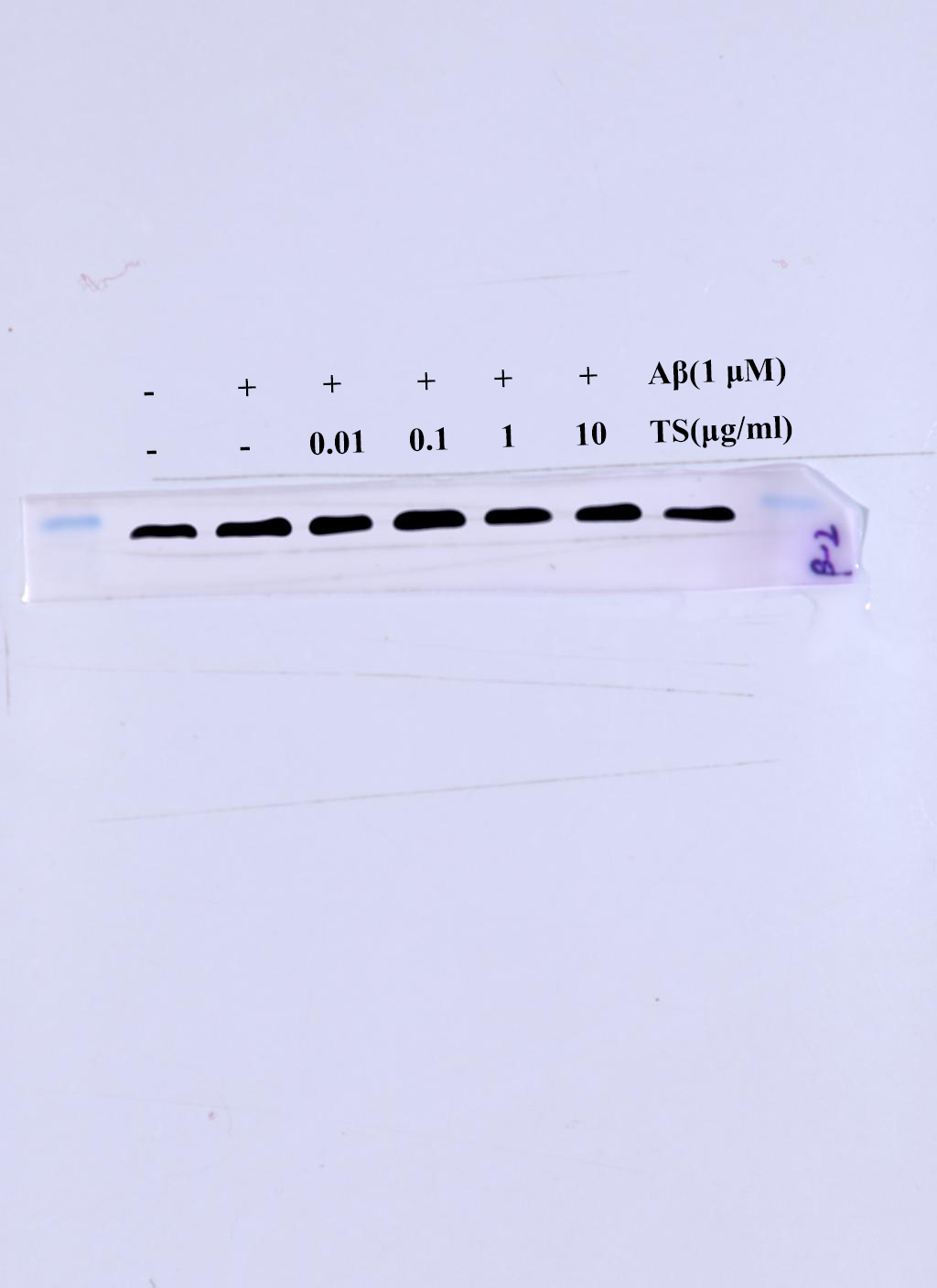

Supplement: Supplementary file 1 [file DataSheet3.ZIP › Fig.6A a┬-actin/Fig.6A orginal images for quantitative anaylsis-1.tif]

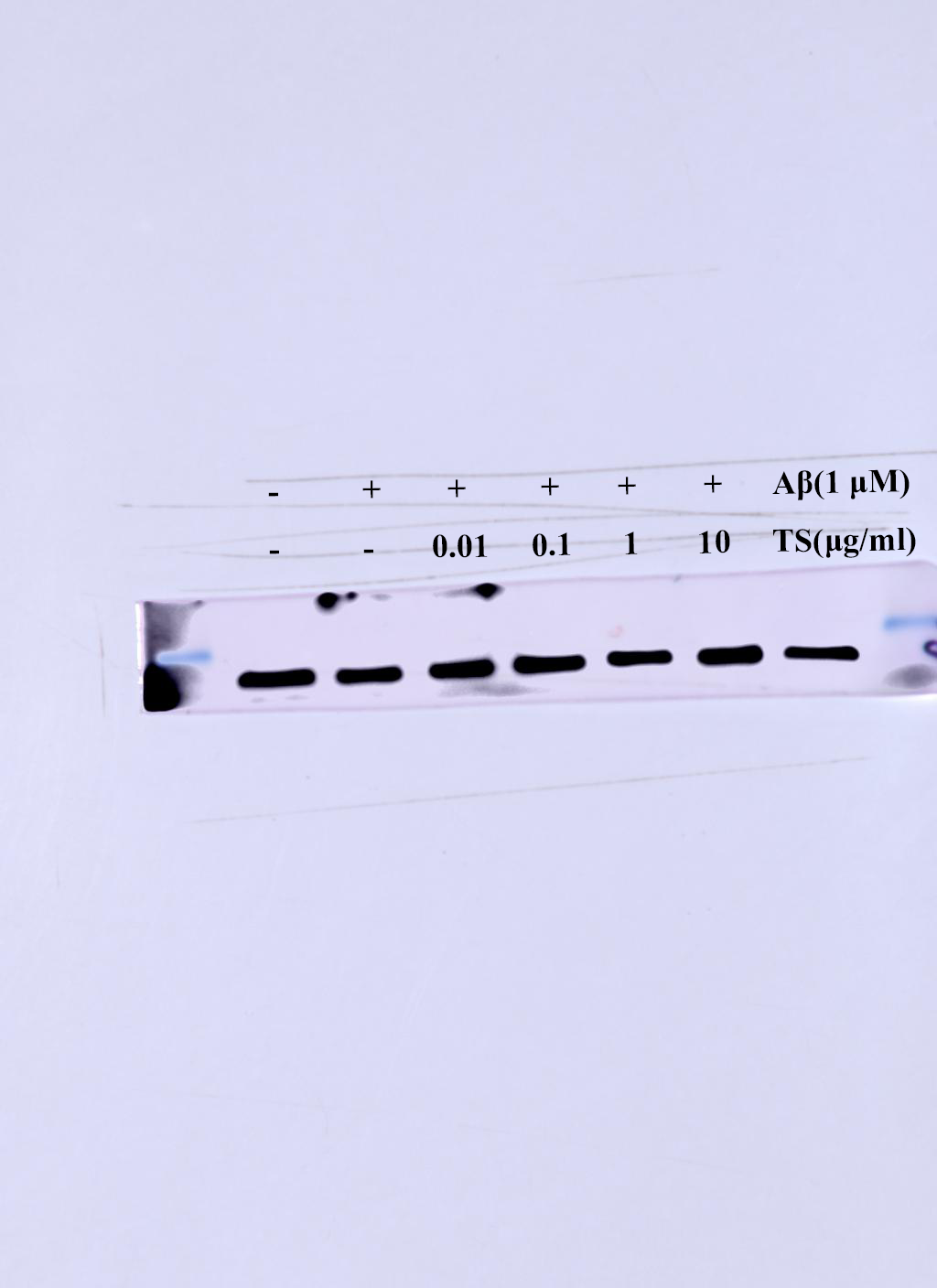

Supplement: Supplementary file 1 [file DataSheet3.ZIP › Fig.6A a┬-actin/Fig.6A orginal images for quantitative anaylsis-2.tif]

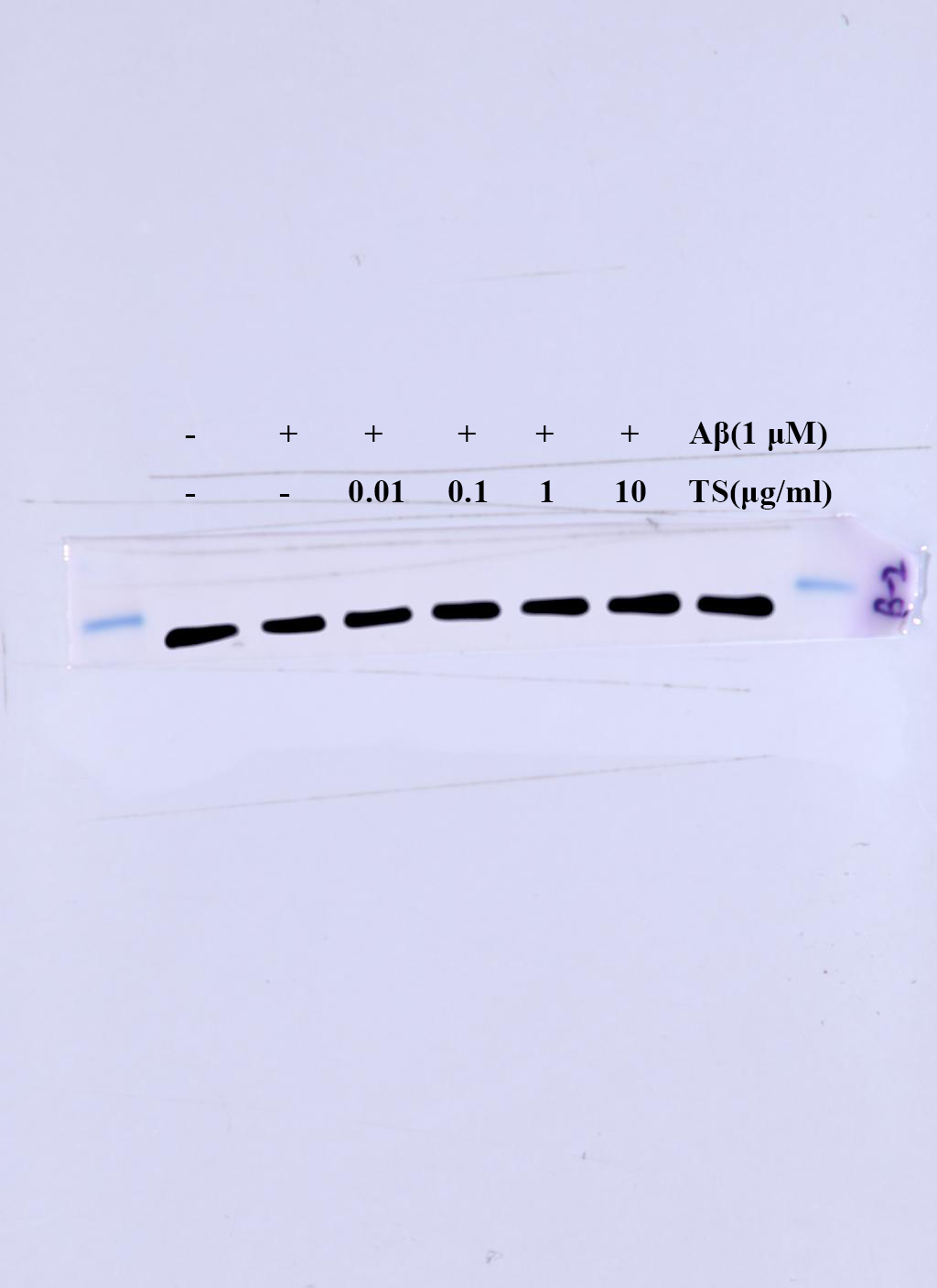

Supplement: Supplementary file 1 [file DataSheet3.ZIP › Fig.6A a┬-actin/Fig.6A orginal images.tif]

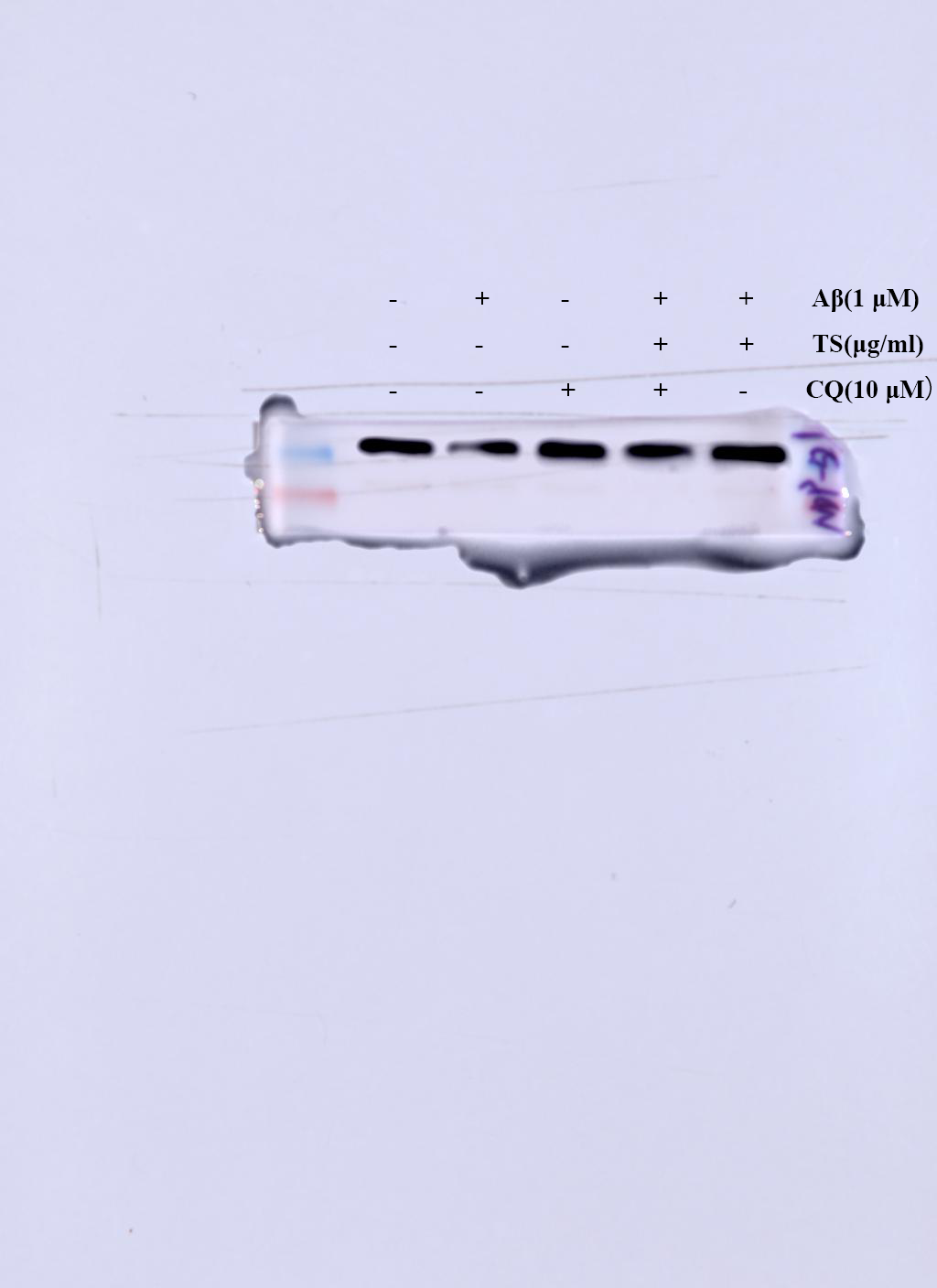

Supplement: Supplementary file 2 [file DataSheet4.ZIP › Fig.7B Beclin/Fig.7B orginal images for quantitative analysis-1.tif]

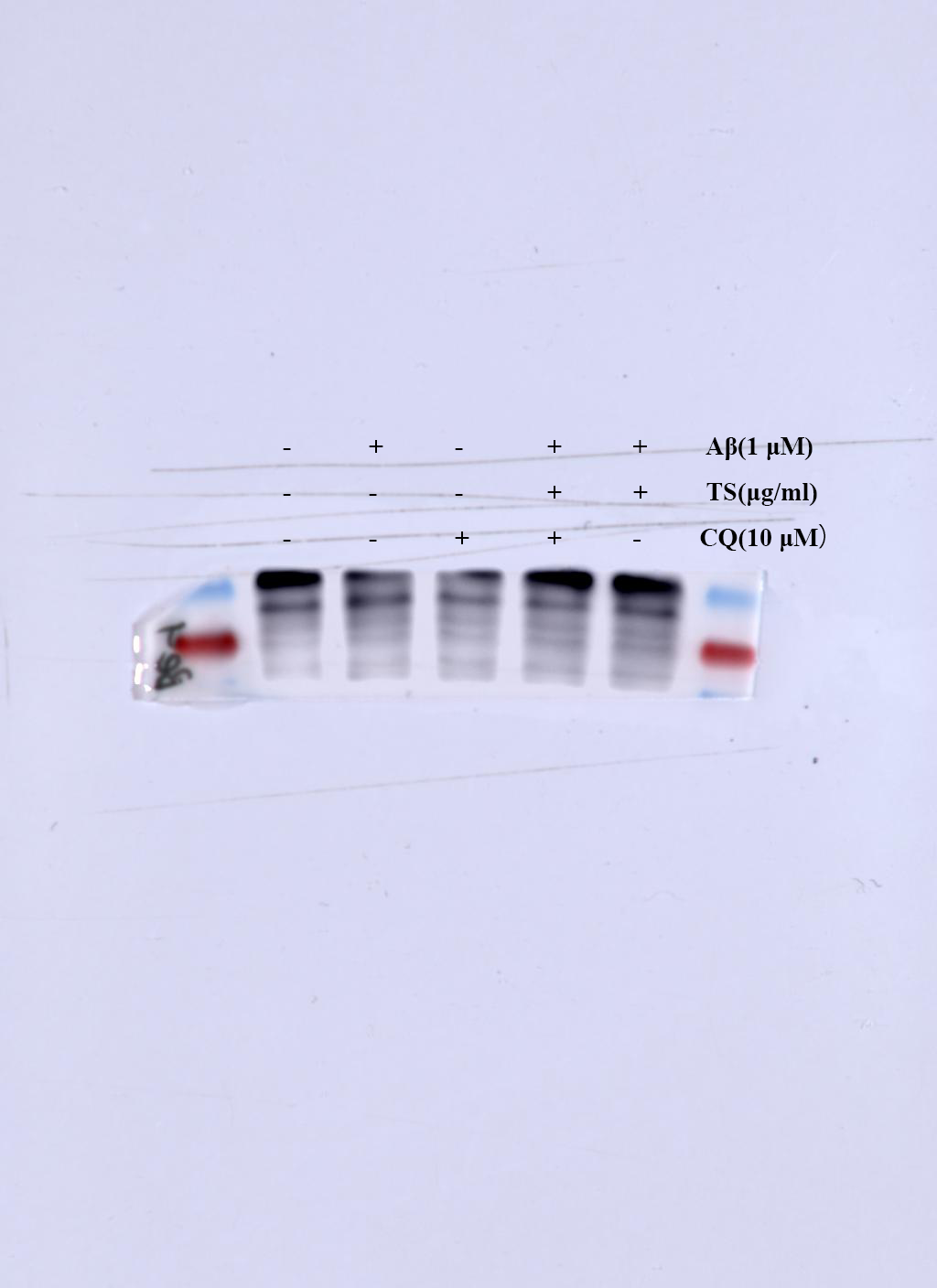

Supplement: Supplementary file 2 [file DataSheet4.ZIP › Fig.7B Beclin/Fig.7B orginal images for quantitative analysis-2.tif]

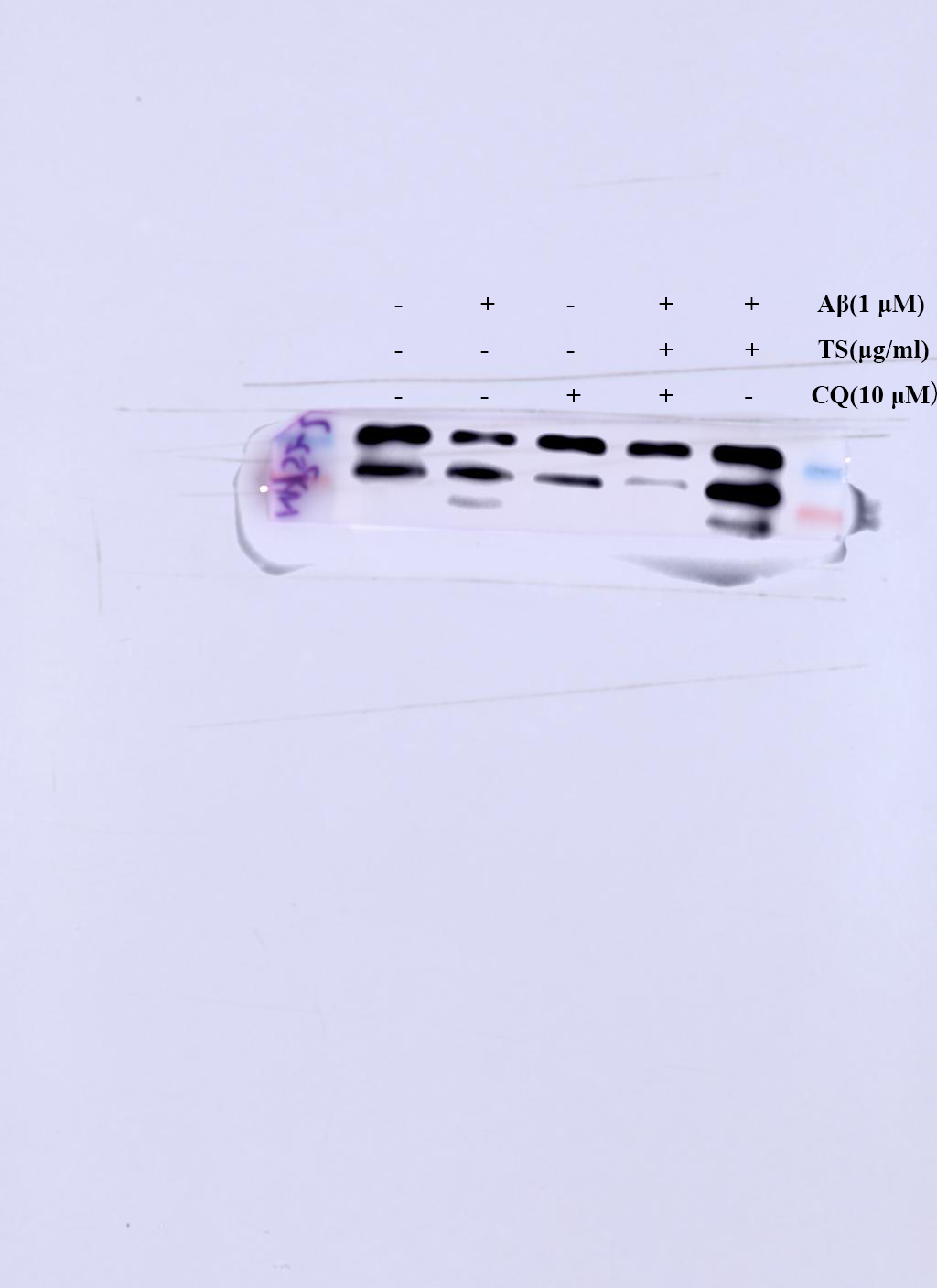

Supplement: Supplementary file 2 [file DataSheet4.ZIP › Fig.7B Beclin/Fig.7B orginal images for quantitative analysis-3.tif]

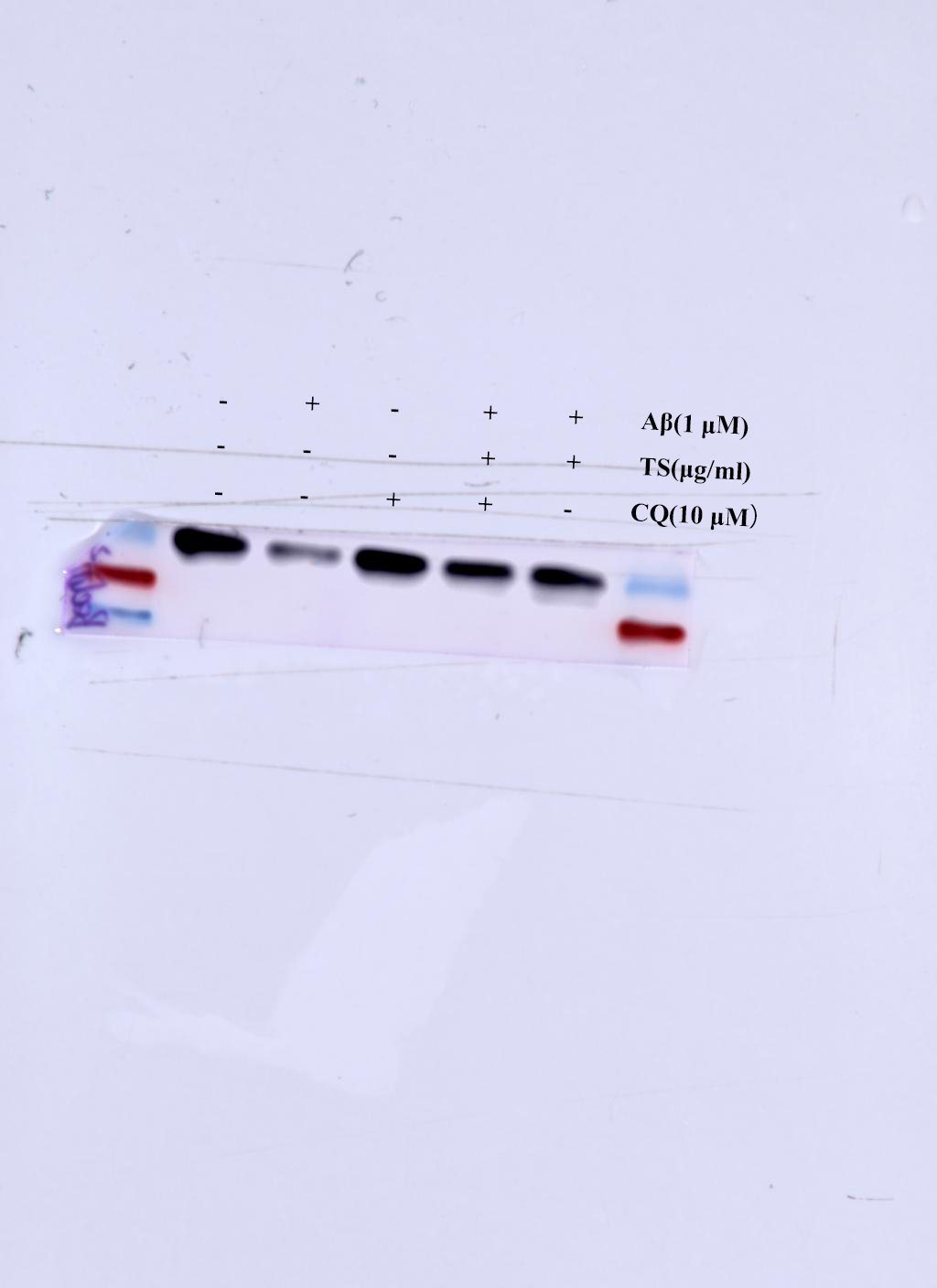

Supplement: Supplementary file 2 [file DataSheet4.ZIP › Fig.7B Beclin/Fig.7B orginal images.tif]

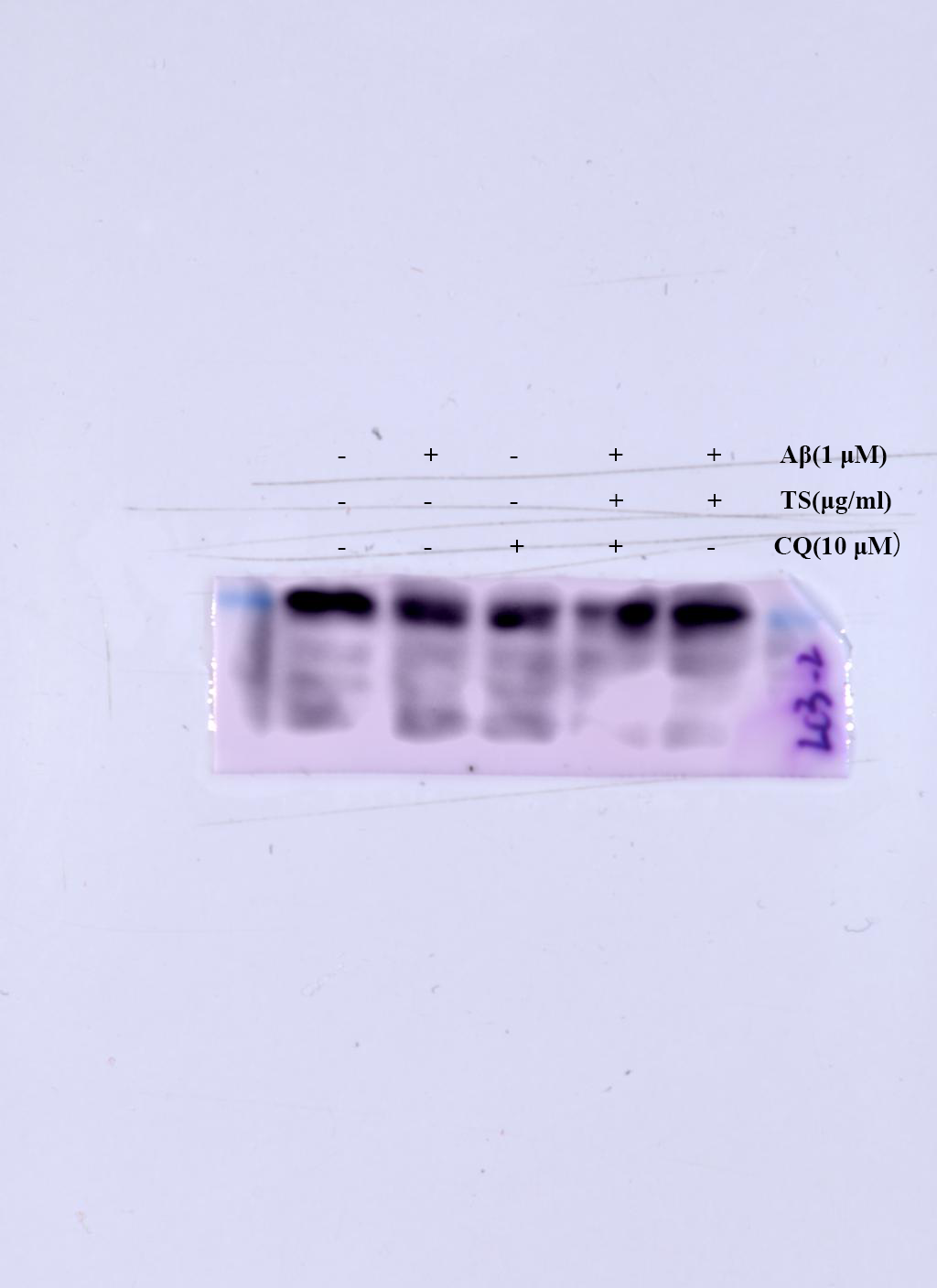

Supplement: Supplementary file 2 [file DataSheet4.ZIP › Fig.7B LC3/Fig.7B orginal images for quantitative anaylsis-1.tif]

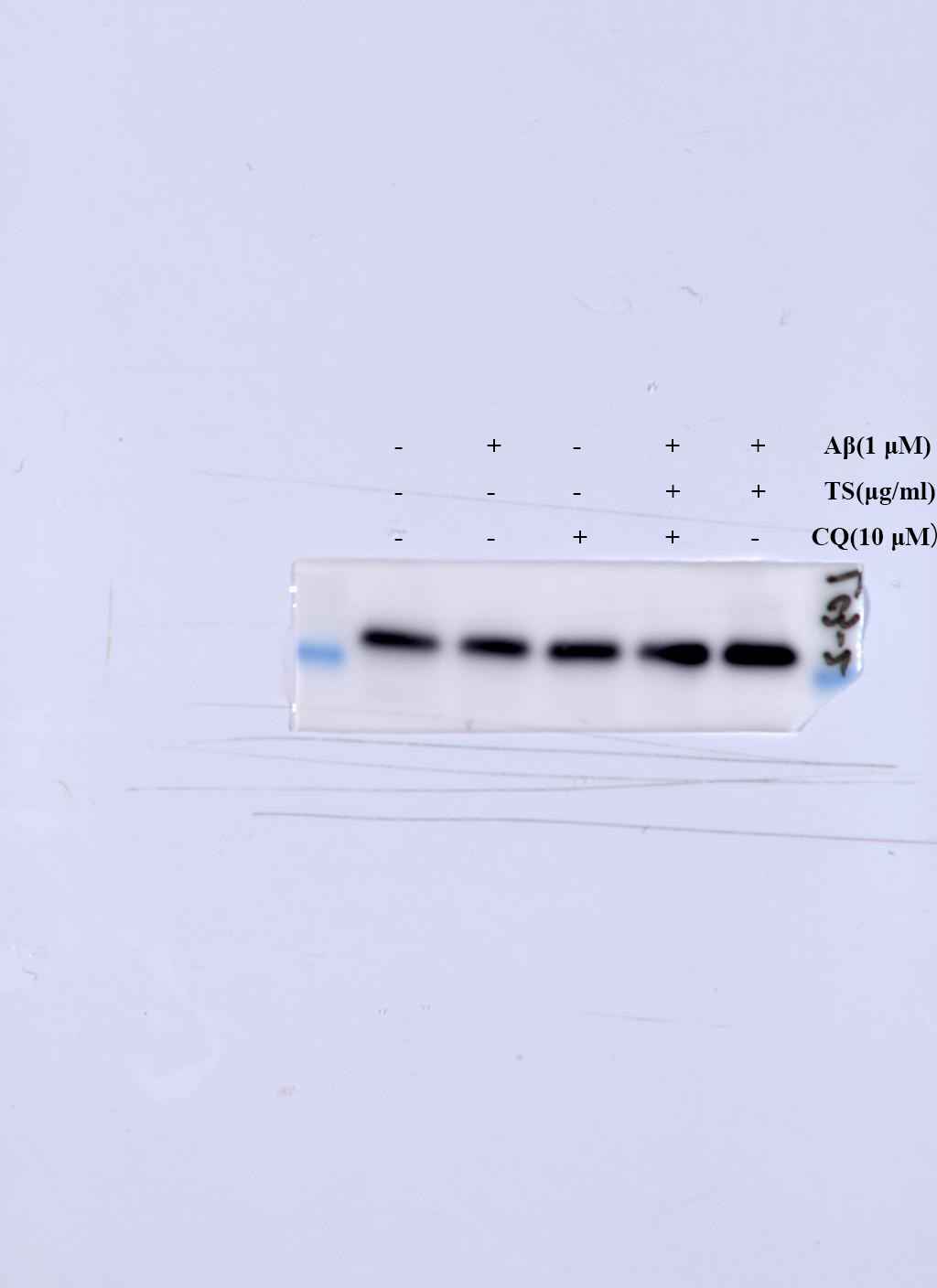

Supplement: Supplementary file 2 [file DataSheet4.ZIP › Fig.7B LC3/Fig.7B orginal images for quantitative anaylsis-2.tif]

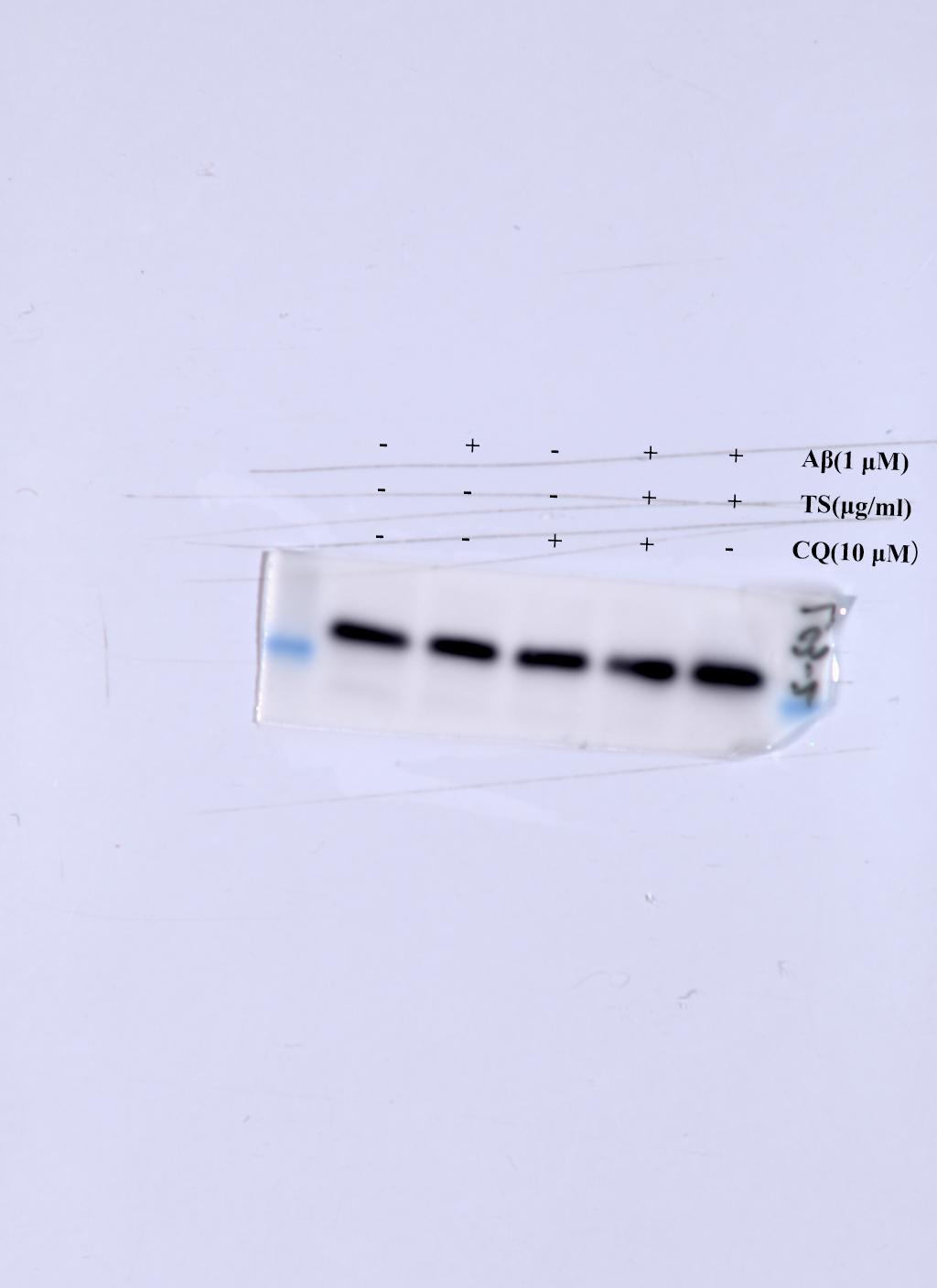

Supplement: Supplementary file 2 [file DataSheet4.ZIP › Fig.7B LC3/Fig.7B orginal images.tif]

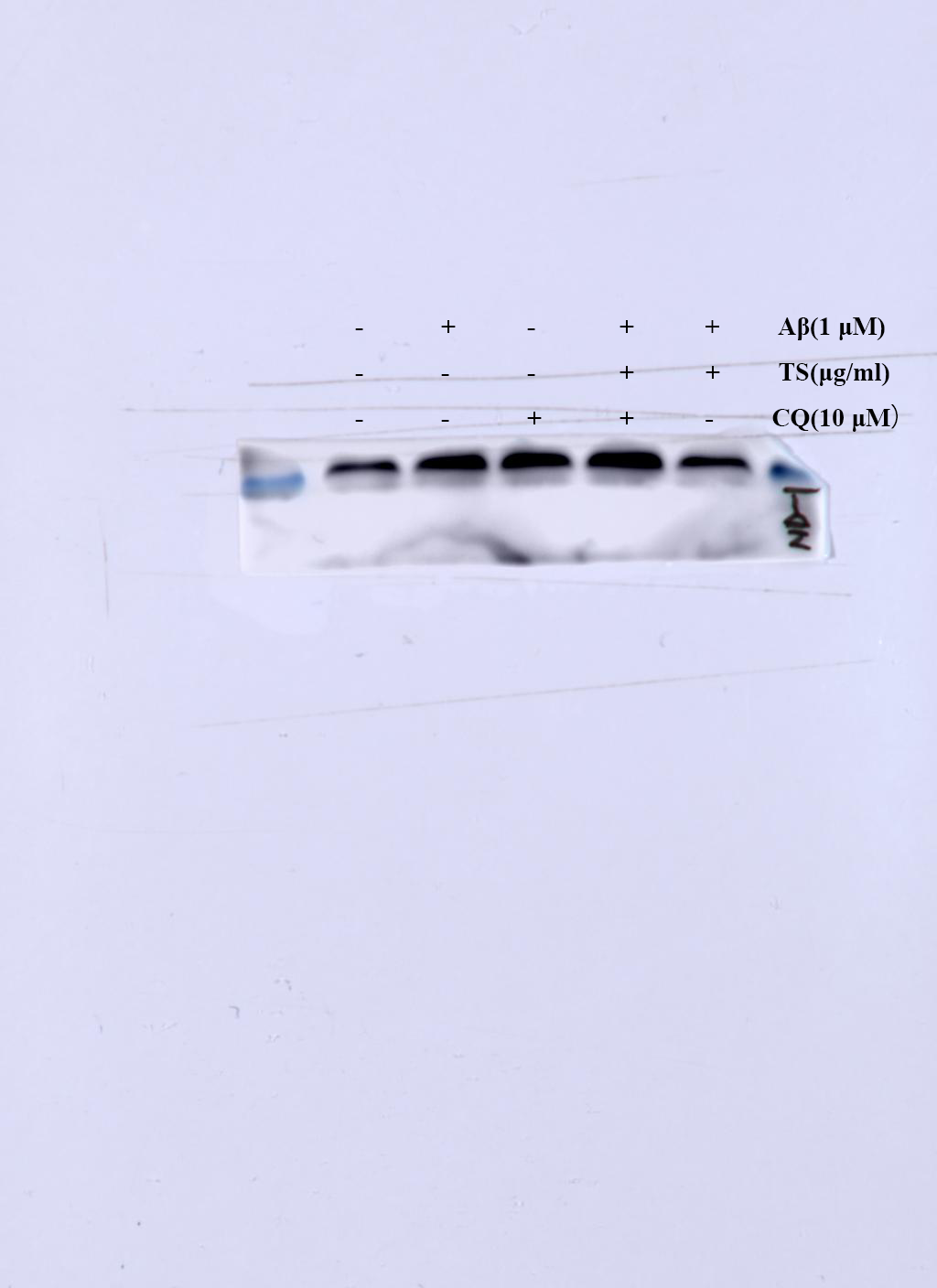

Supplement: Supplementary file 2 [file DataSheet4.ZIP › Fig.7B NDP52/Fig.7B orginal images for quantitative anaylsis-1.tif]

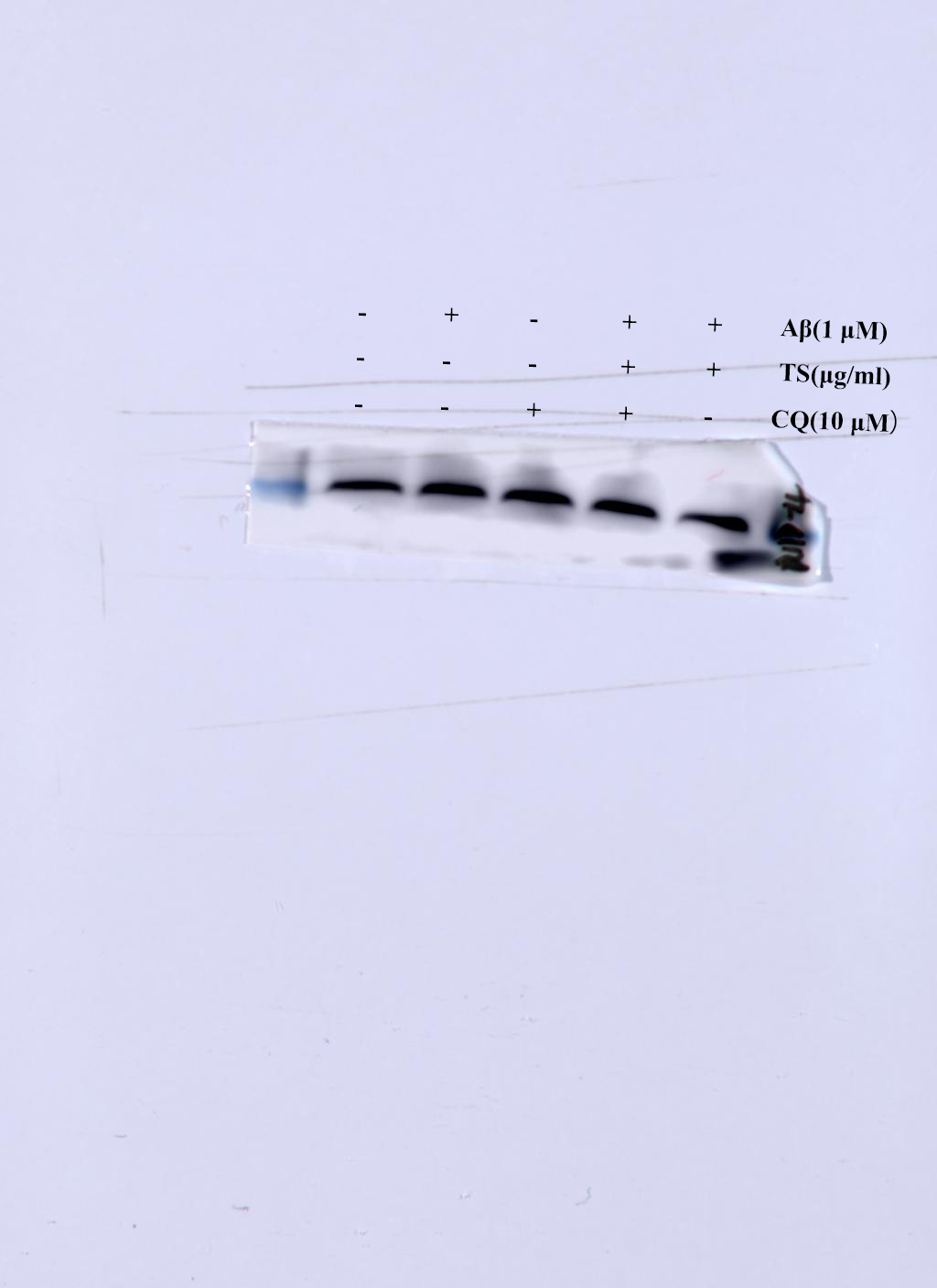

Supplement: Supplementary file 2 [file DataSheet4.ZIP › Fig.7B NDP52/Fig.7B orginal images for quantitative anaylsis-2.tif]

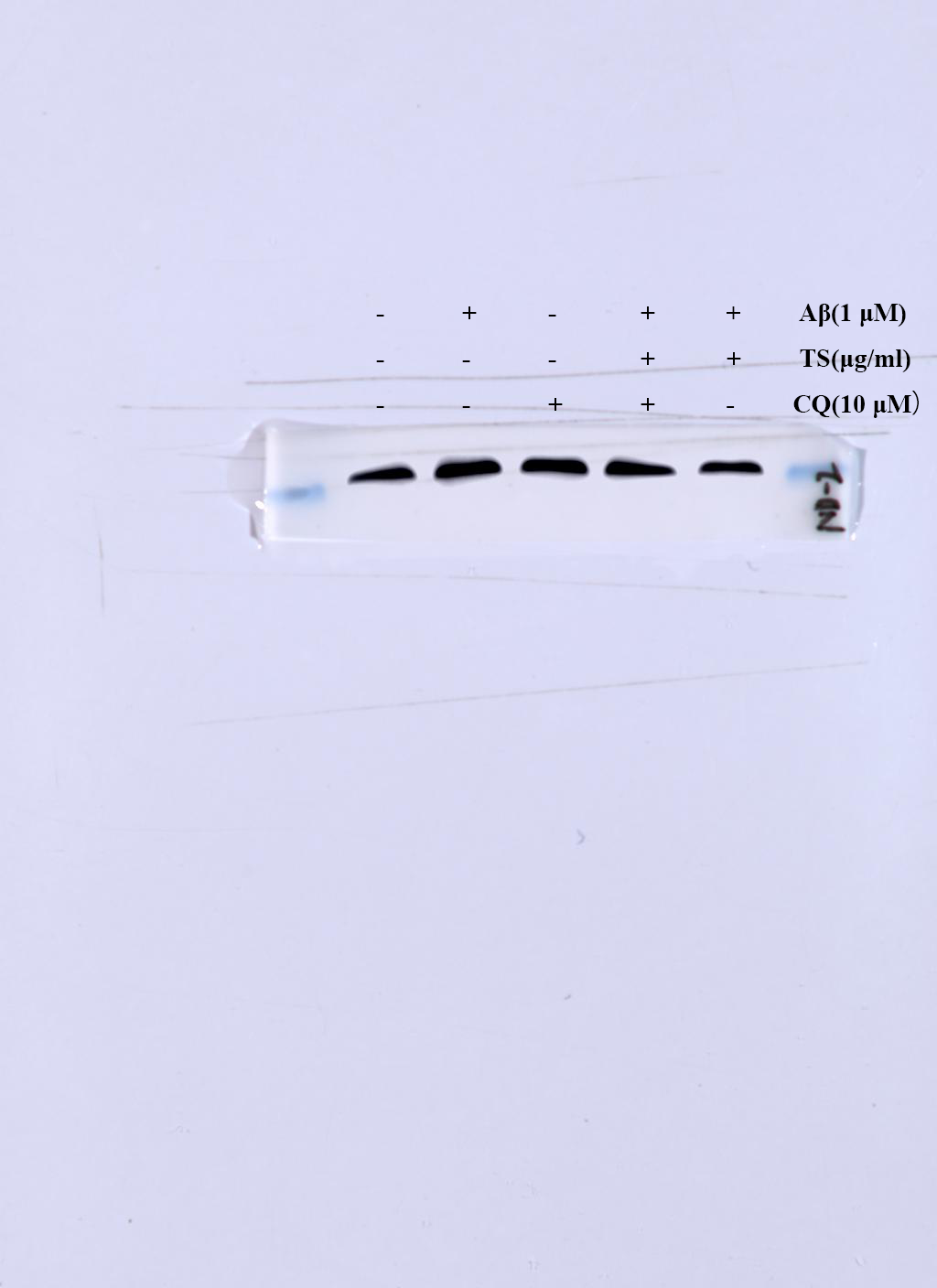

Supplement: Supplementary file 2 [file DataSheet4.ZIP › Fig.7B NDP52/Fig.7B orginal images.tif]

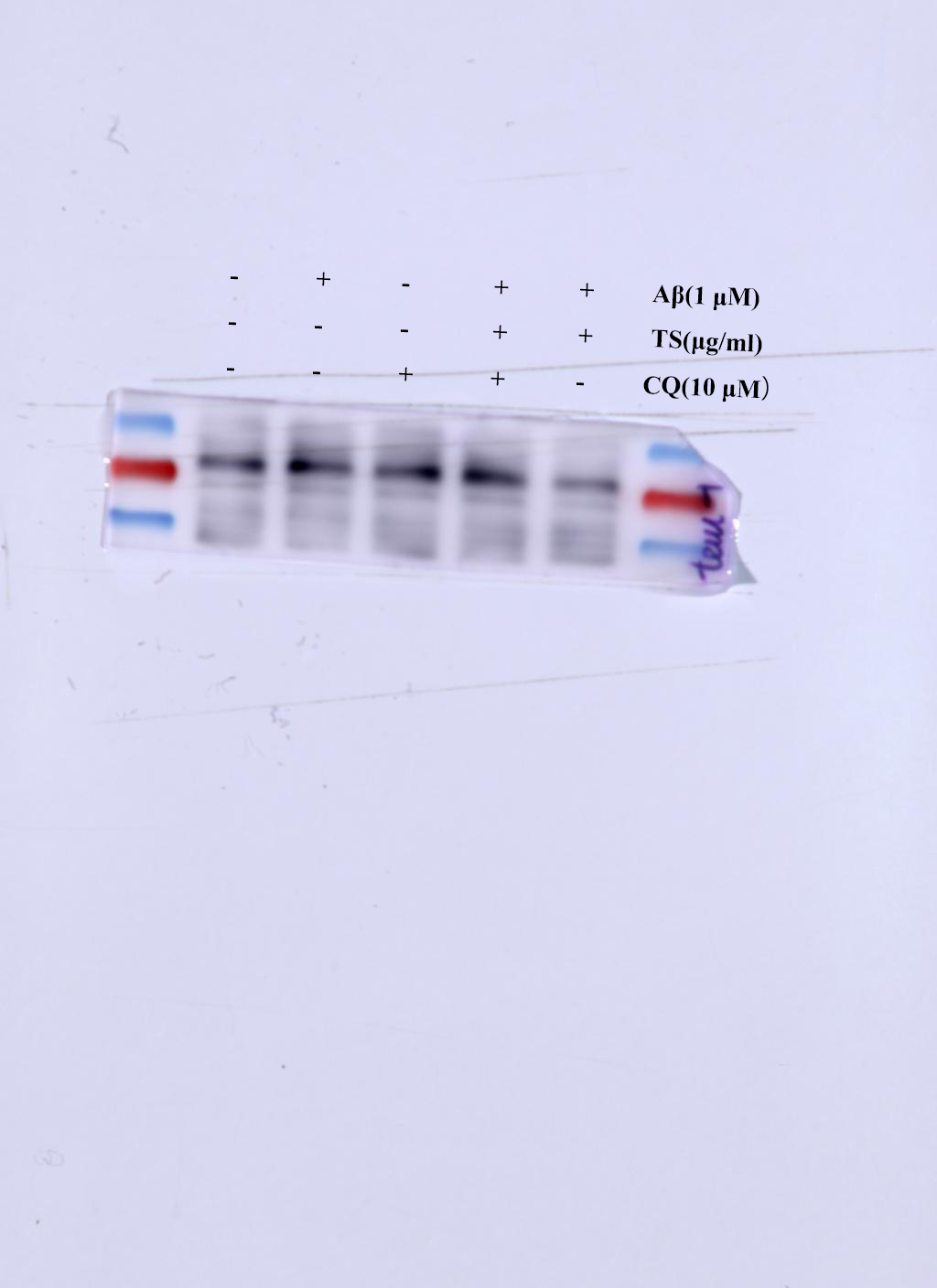

Supplement: Supplementary file 2 [file DataSheet4.ZIP › Fig.7B p-tau/Fig.7B orginal images for quantitative anaylsis-1.tif]

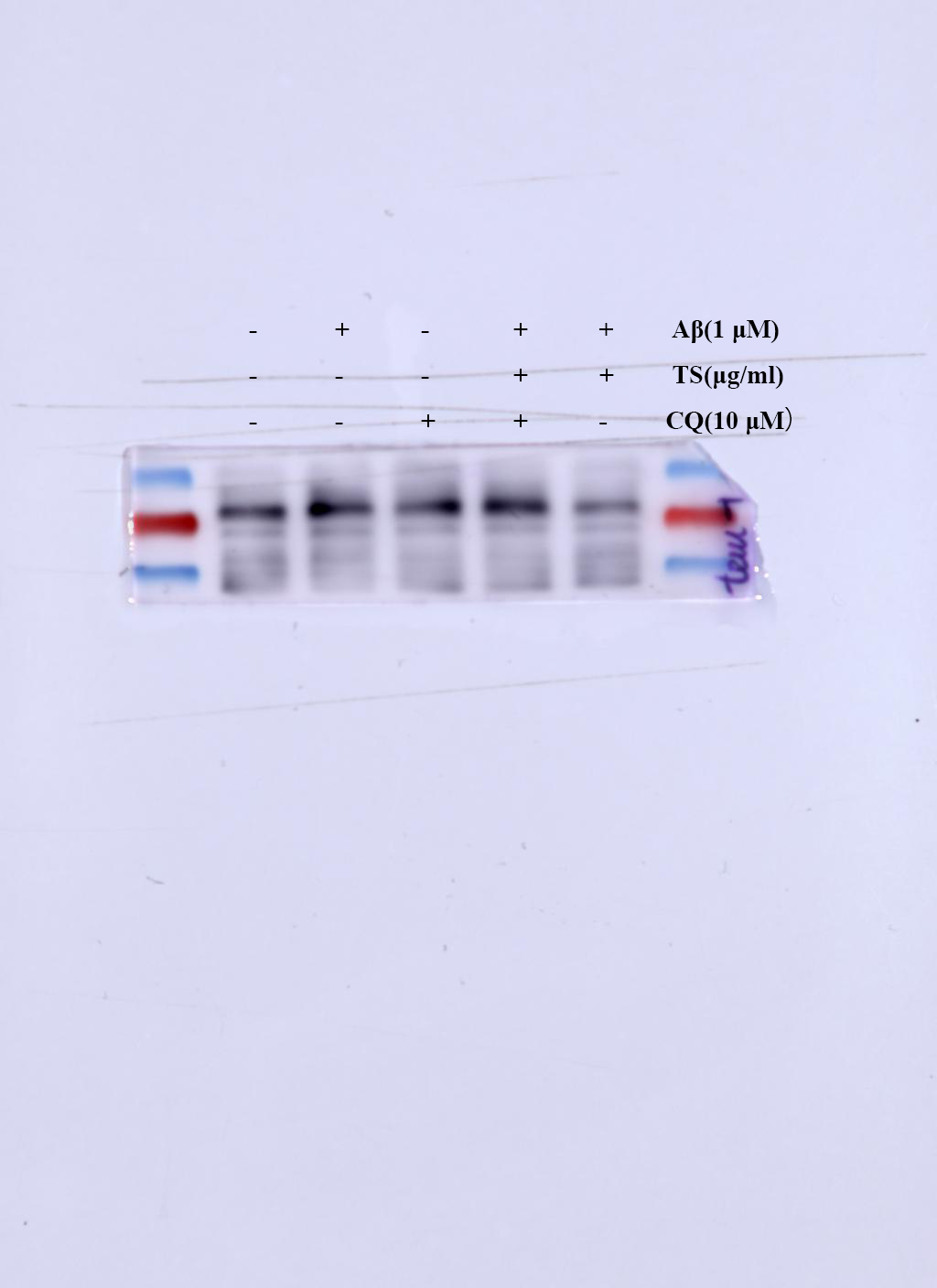

Supplement: Supplementary file 2 [file DataSheet4.ZIP › Fig.7B p-tau/Fig.7B orginal images for quantitative anaylsis-2.tif]

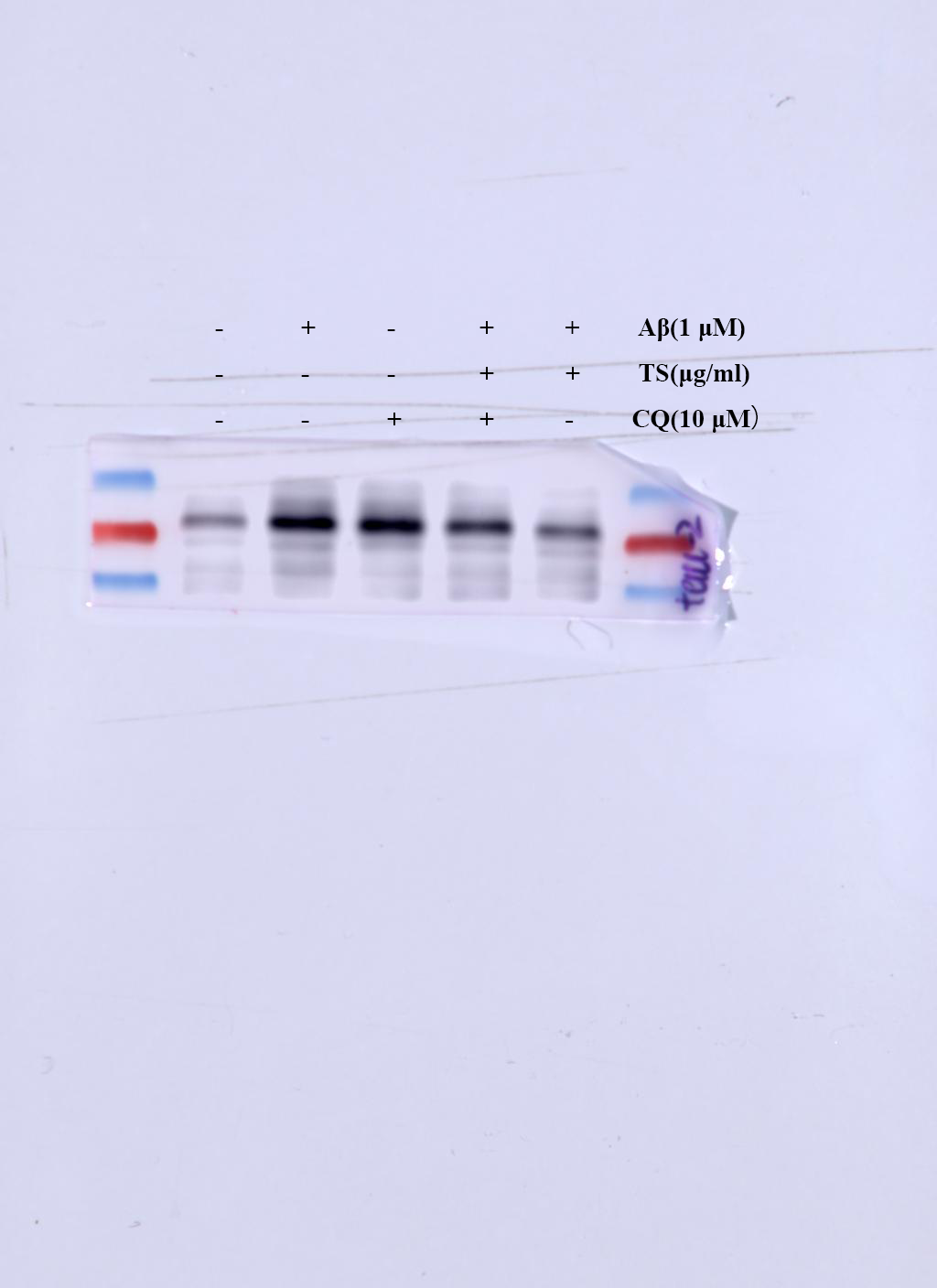

Supplement: Supplementary file 2 [file DataSheet4.ZIP › Fig.7B p-tau/Fig.7B orginal images.tif]

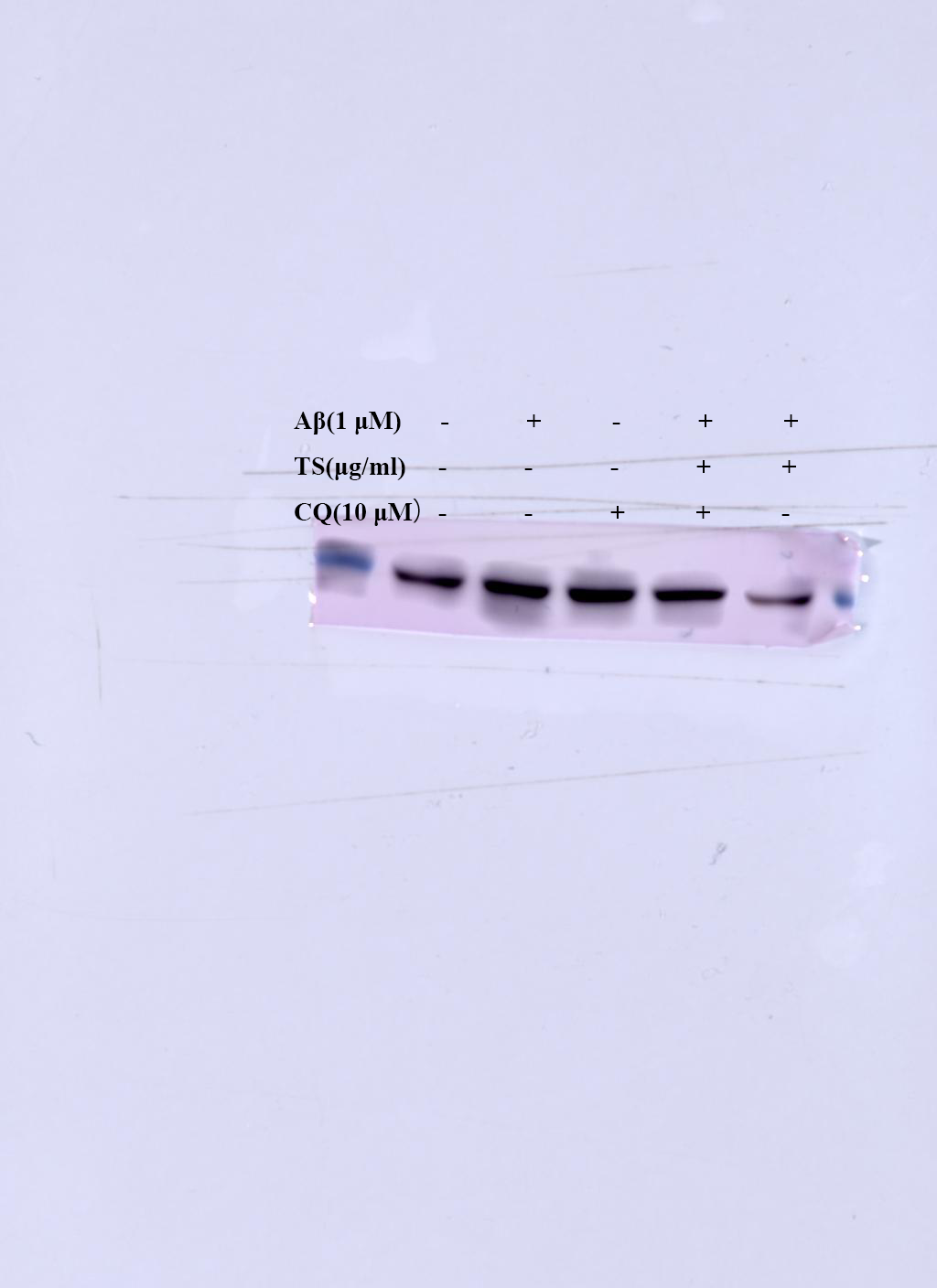

Supplement: Supplementary file 2 [file DataSheet4.ZIP › Fig.7B p62/Fig.4B orginal images for quantitative analysis-2.tif]

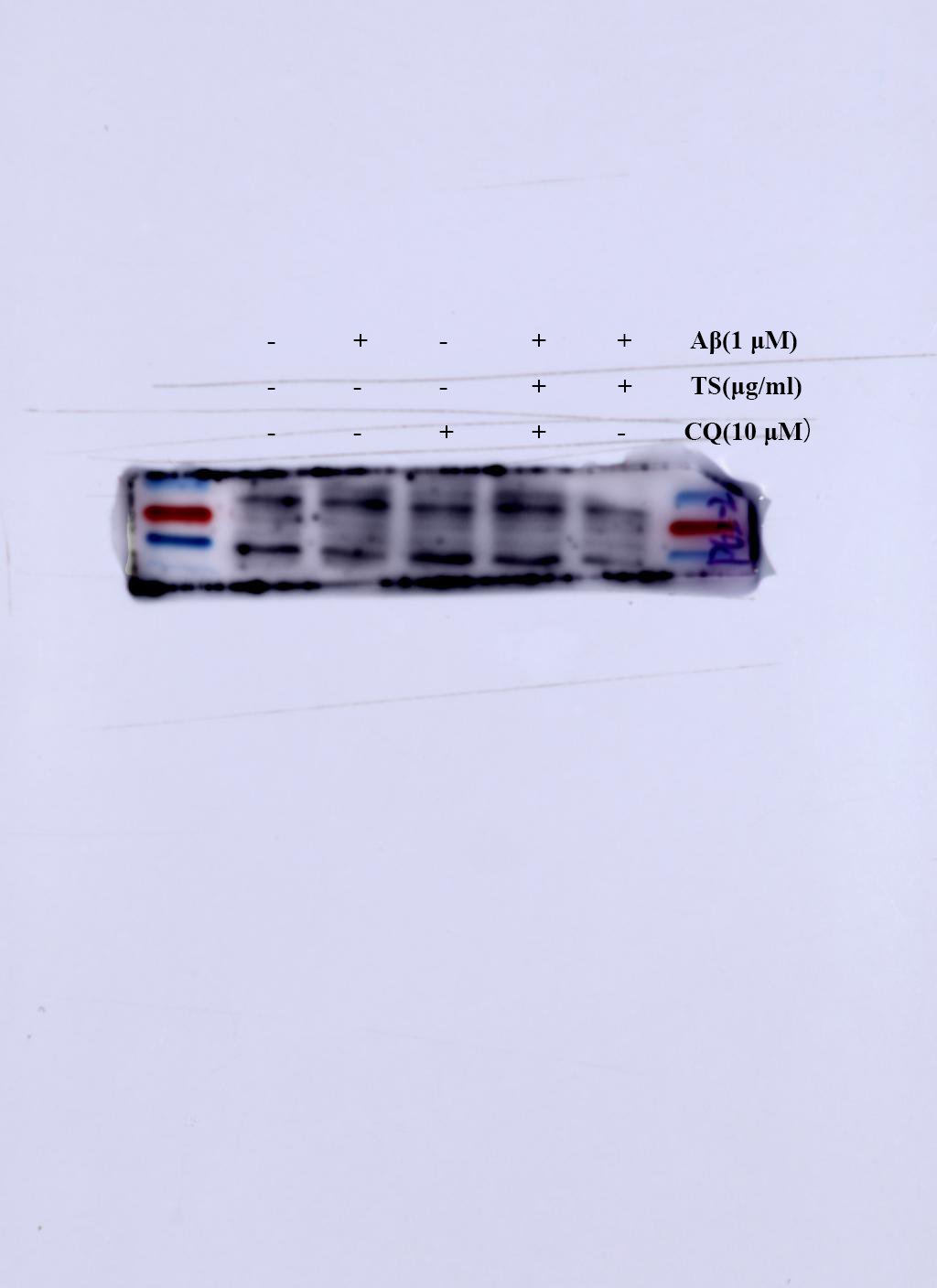

Supplement: Supplementary file 2 [file DataSheet4.ZIP › Fig.7B p62/Fig.7B orginal images for quantitative anaylsis-1.tif]

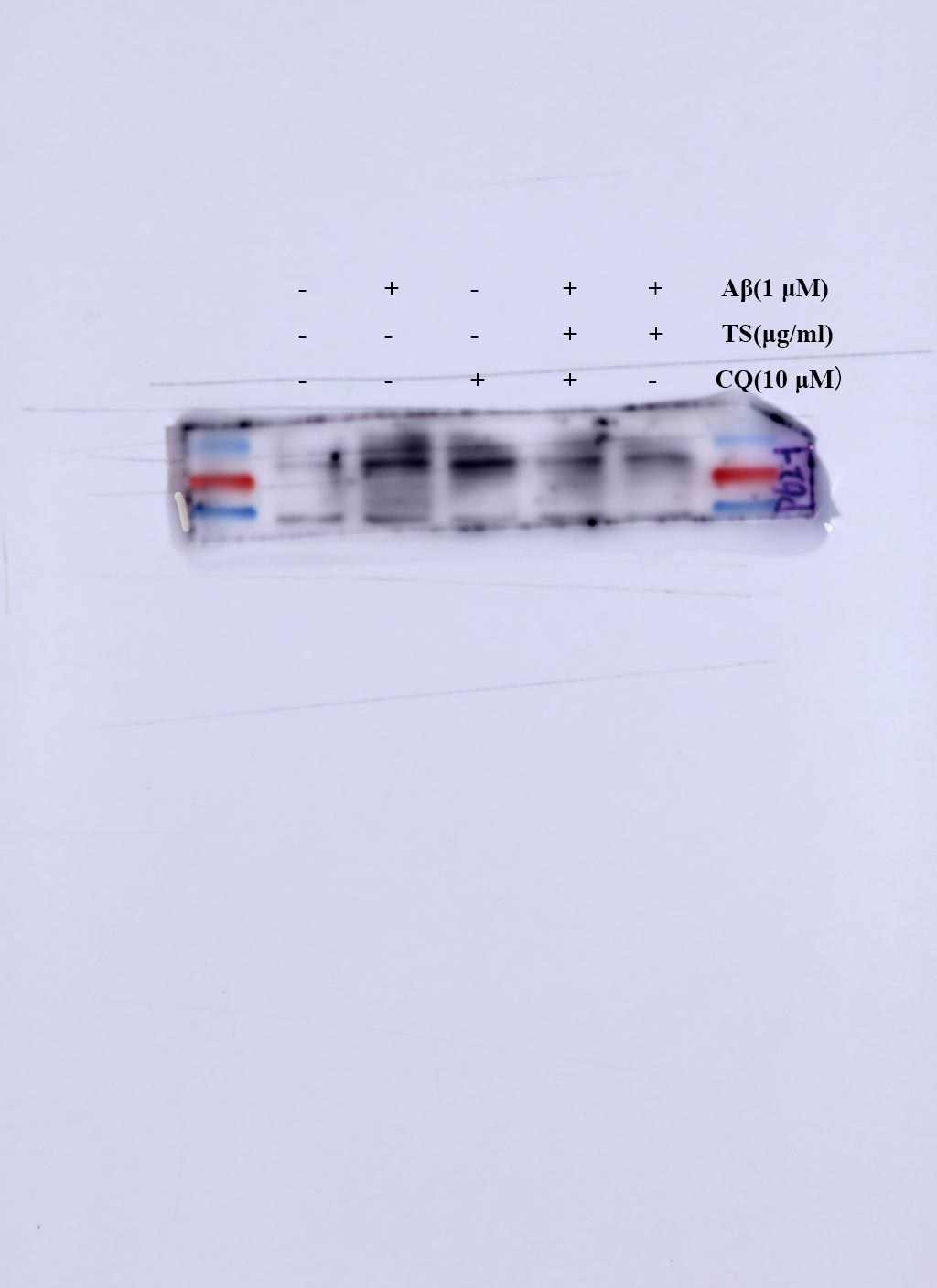

Supplement: Supplementary file 2 [file DataSheet4.ZIP › Fig.7B p62/Fig.7B orginal images.tif]

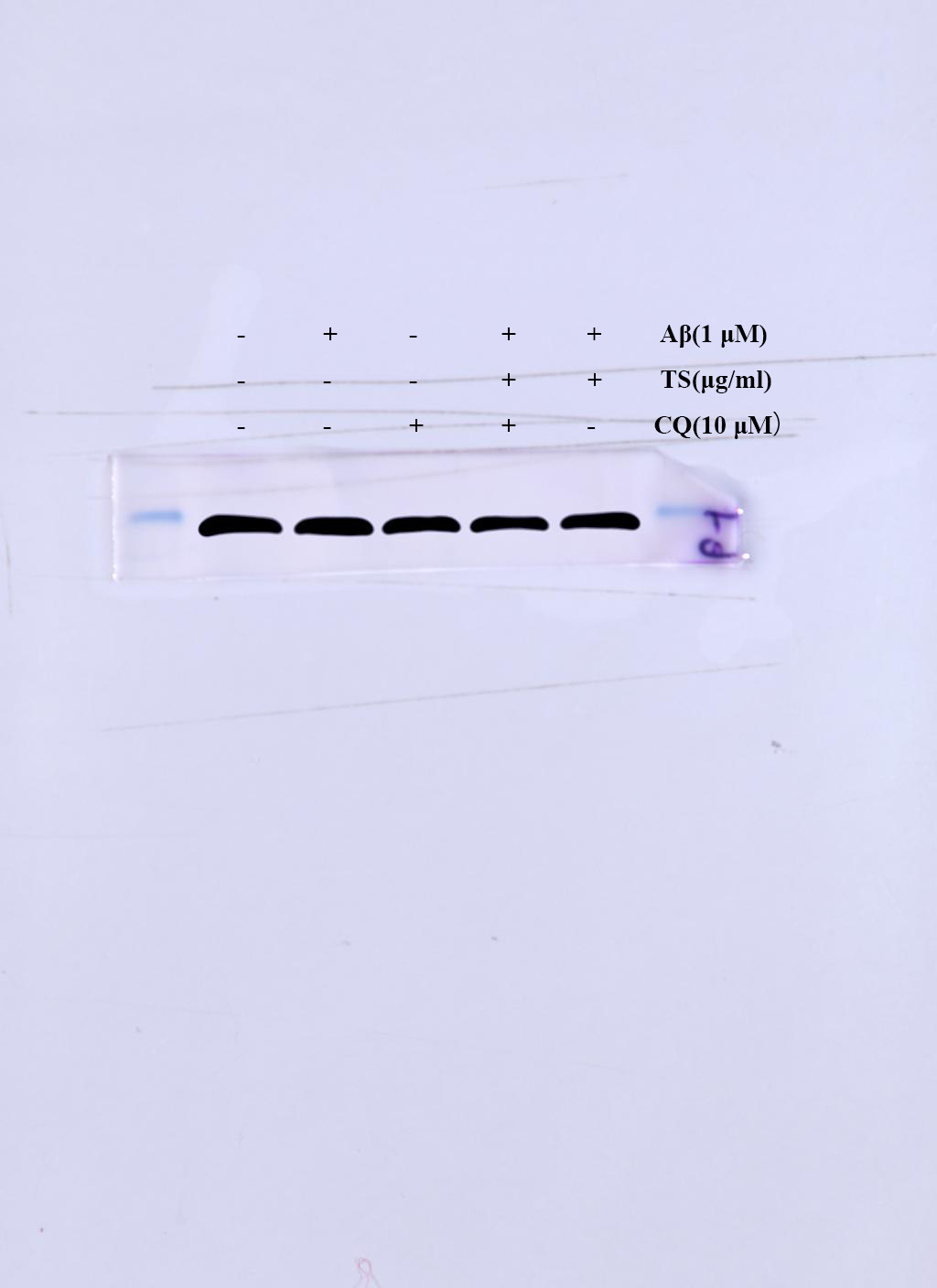

Supplement: Supplementary file 2 [file DataSheet4.ZIP › Fig.7B a┬-actin/Fig.7B orginal images for quantitative analysis-1.tif]

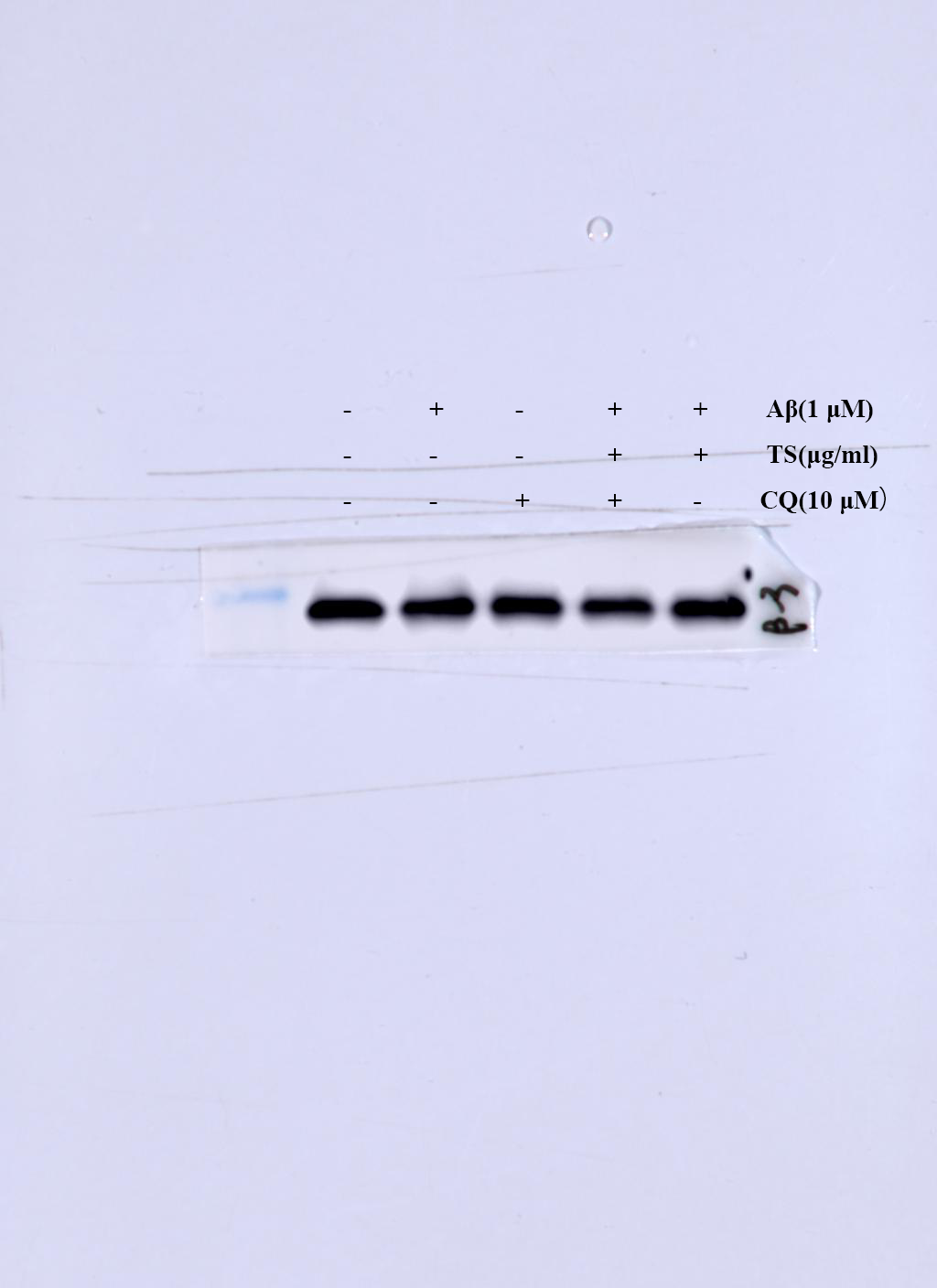

Supplement: Supplementary file 2 [file DataSheet4.ZIP › Fig.7B a┬-actin/Fig.7B orginal images for quantitative analysis-2.tif]

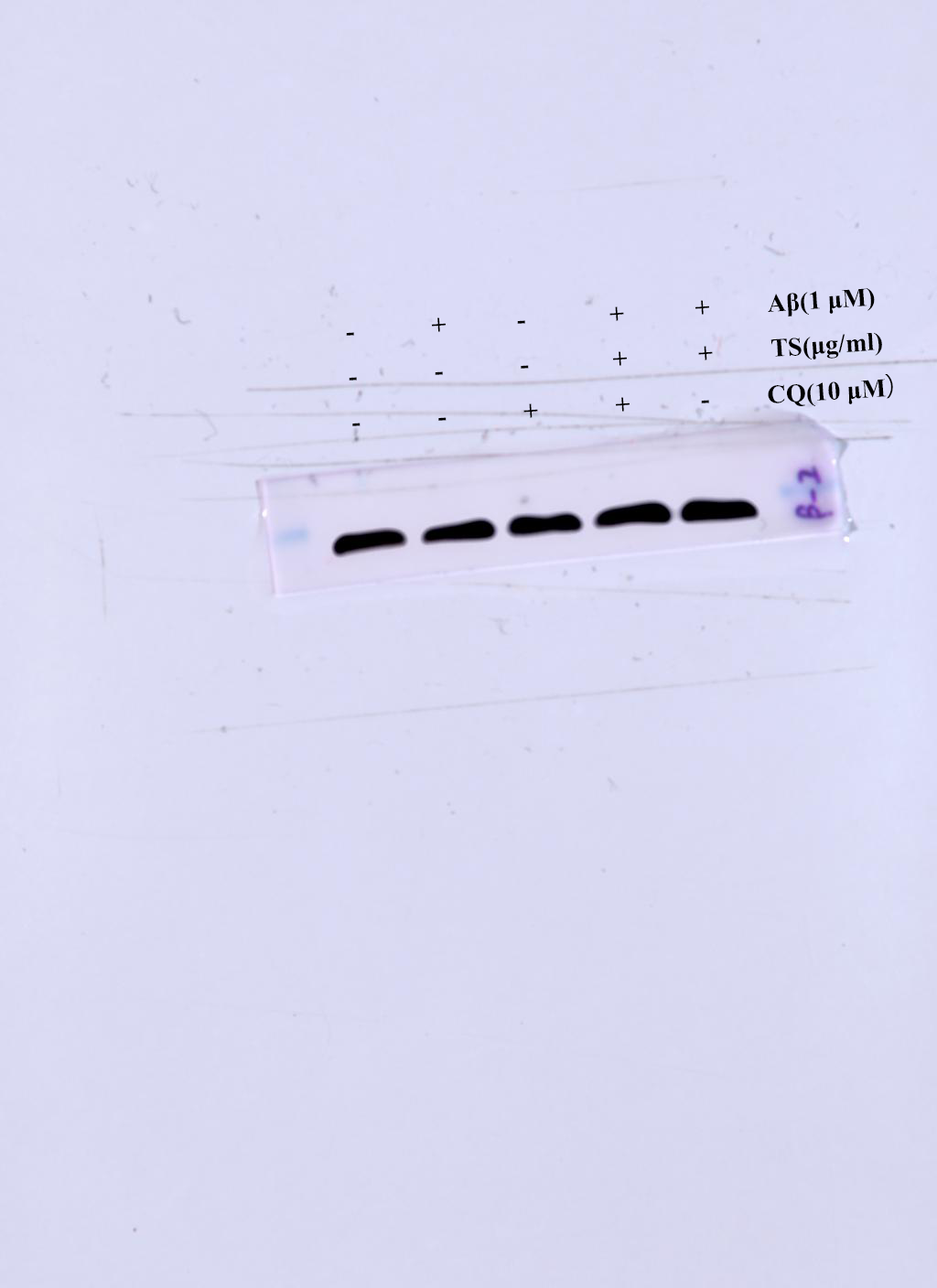

Supplement: Supplementary file 2 [file DataSheet4.ZIP › Fig.7B a┬-actin/Fig.7B orginal images.tif]

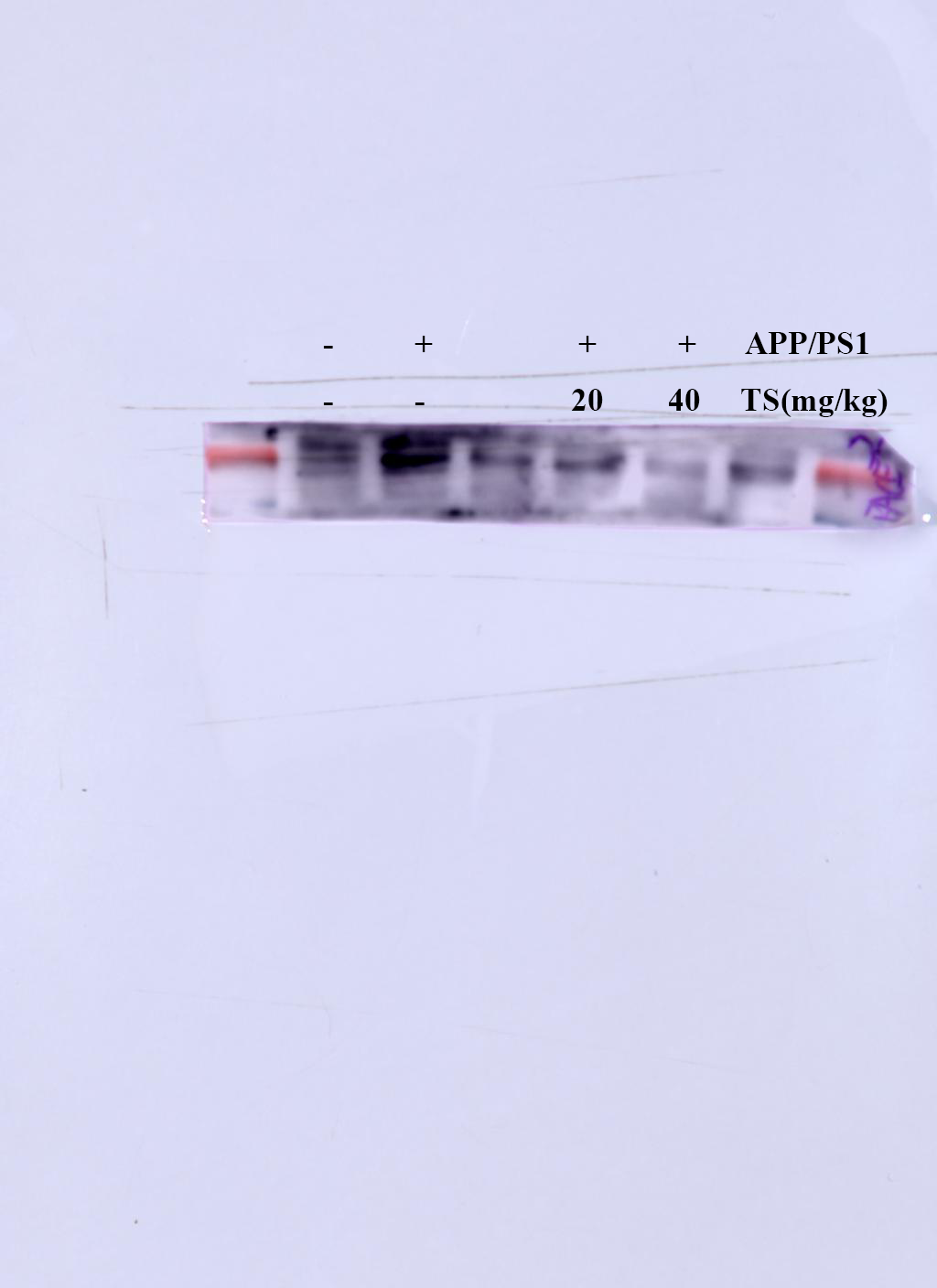

Supplement: Supplementary file 3 [file DataSheet1.ZIP › Fig.4B BACE1/Fig.4B orgianl images for quantitative analysis-1.tif]

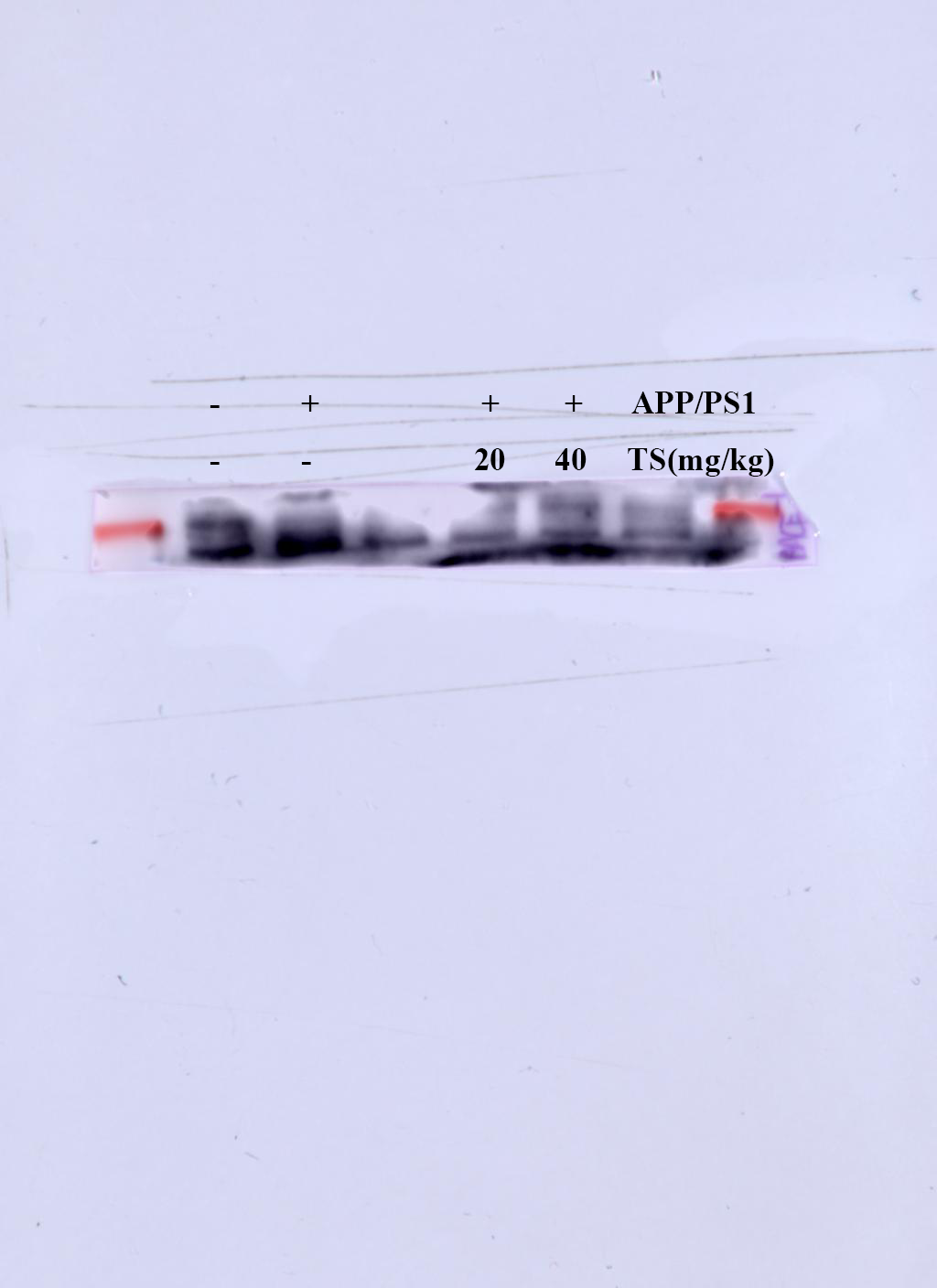

Supplement: Supplementary file 3 [file DataSheet1.ZIP › Fig.4B BACE1/Fig.4B orgianl images for quantitative analysis-2.tif]

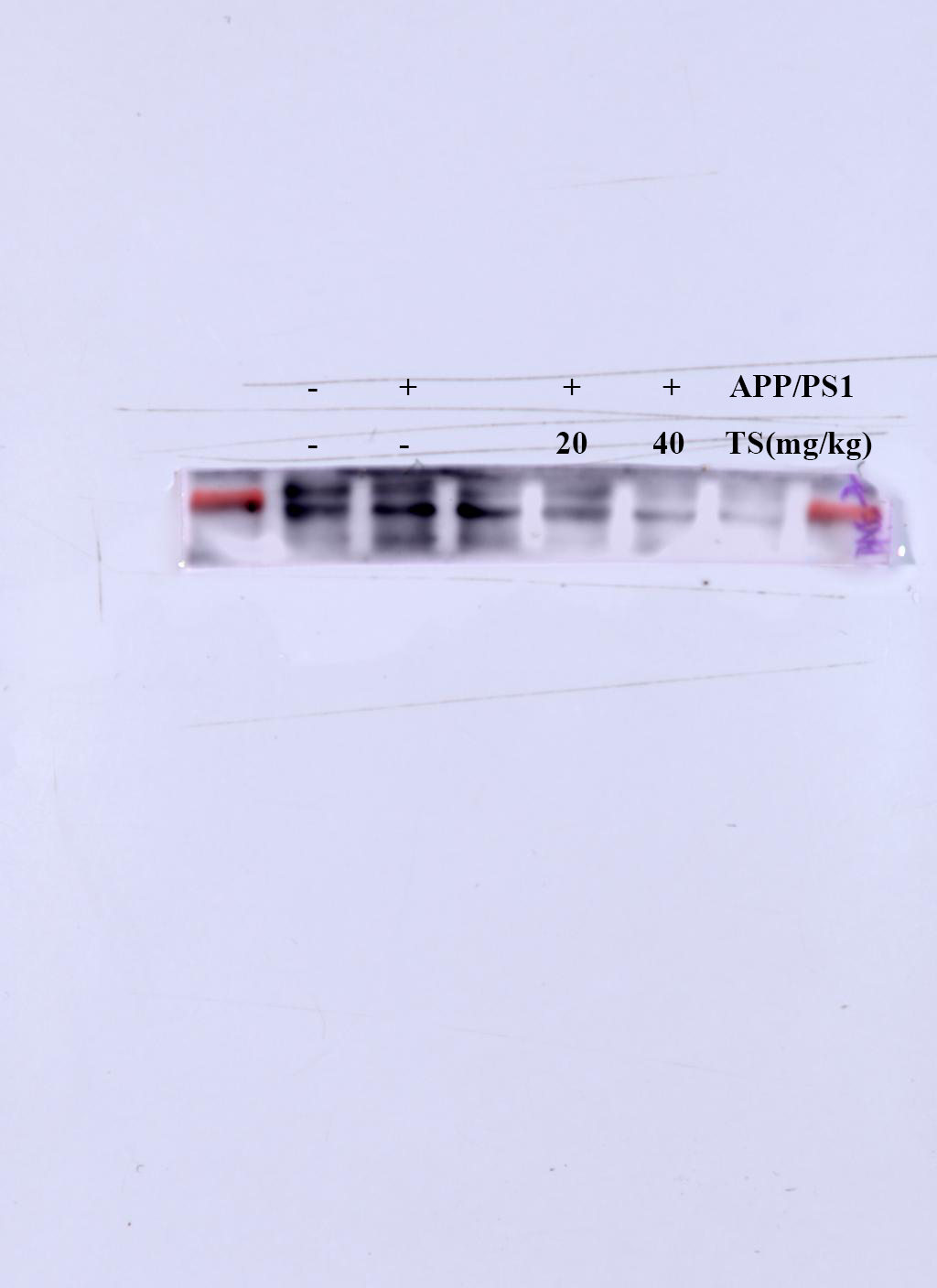

Supplement: Supplementary file 3 [file DataSheet1.ZIP › Fig.4B BACE1/Fig.4B orgianl images.tif]

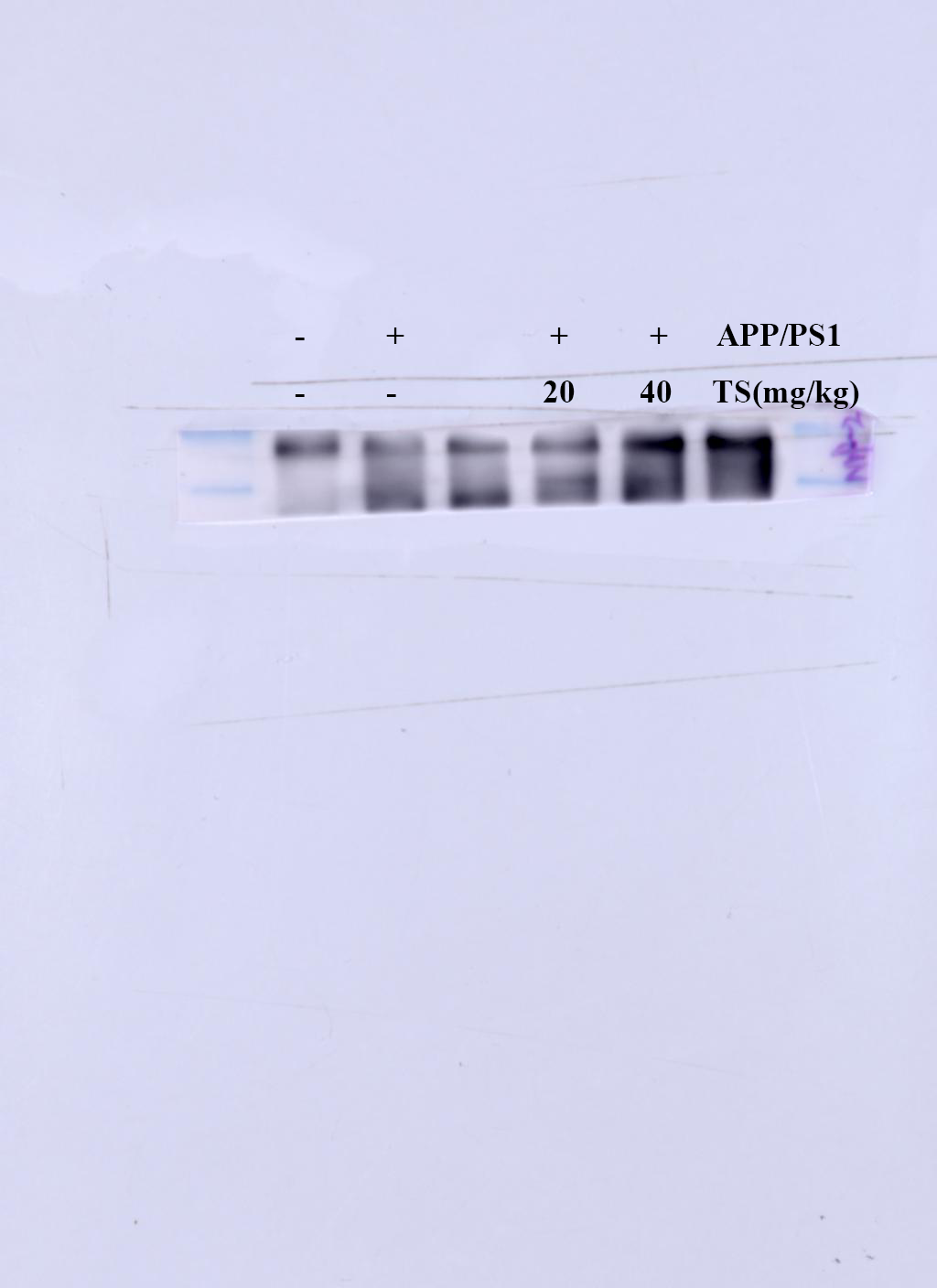

Supplement: Supplementary file 3 [file DataSheet1.ZIP › Fig.4B Nrf2/Fig.4B orgianl images for quantitative analysis-1.tif]

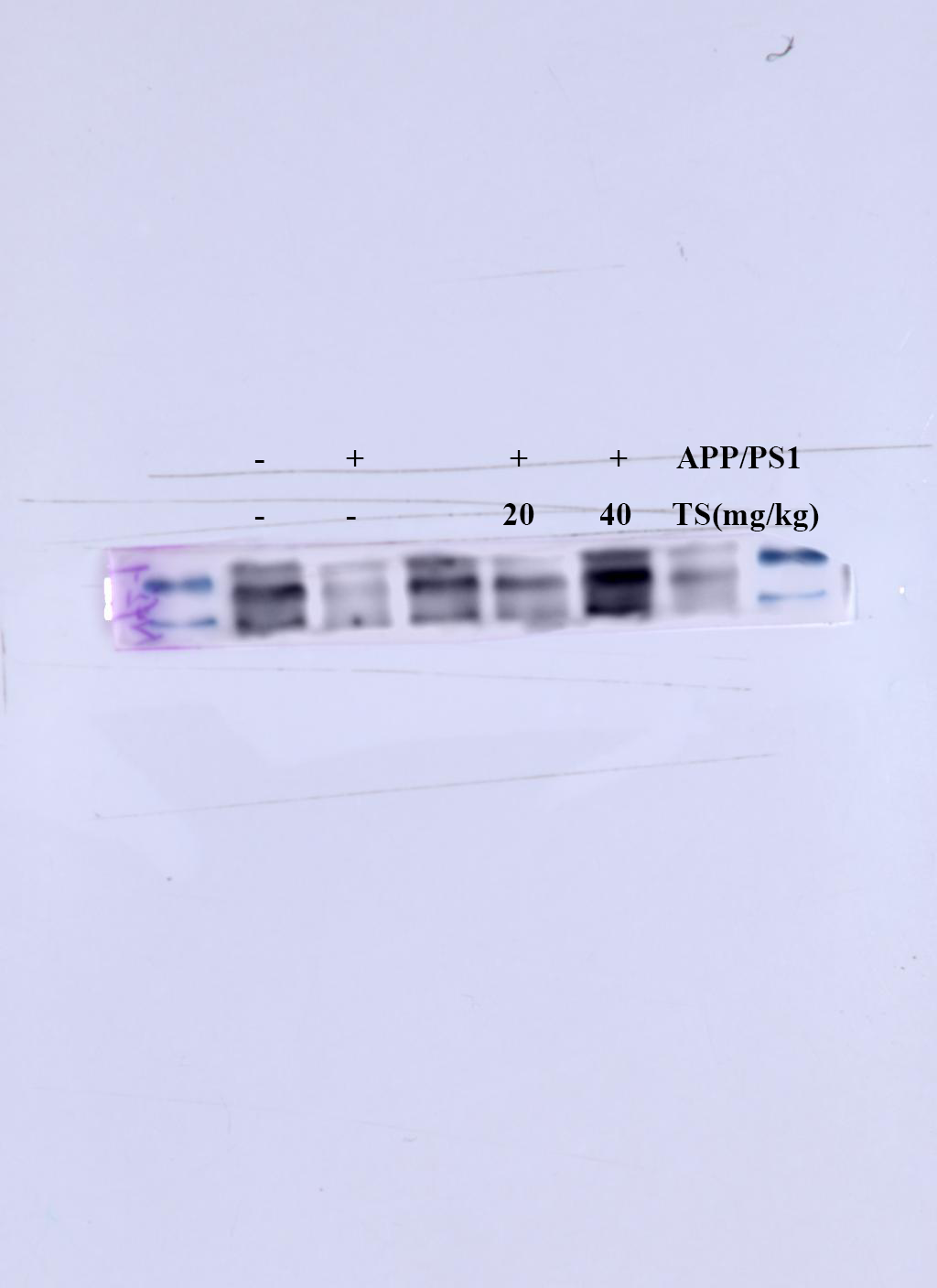

Supplement: Supplementary file 3 [file DataSheet1.ZIP › Fig.4B Nrf2/Fig.4B orgianl images for quantitative analysis-2.tif]

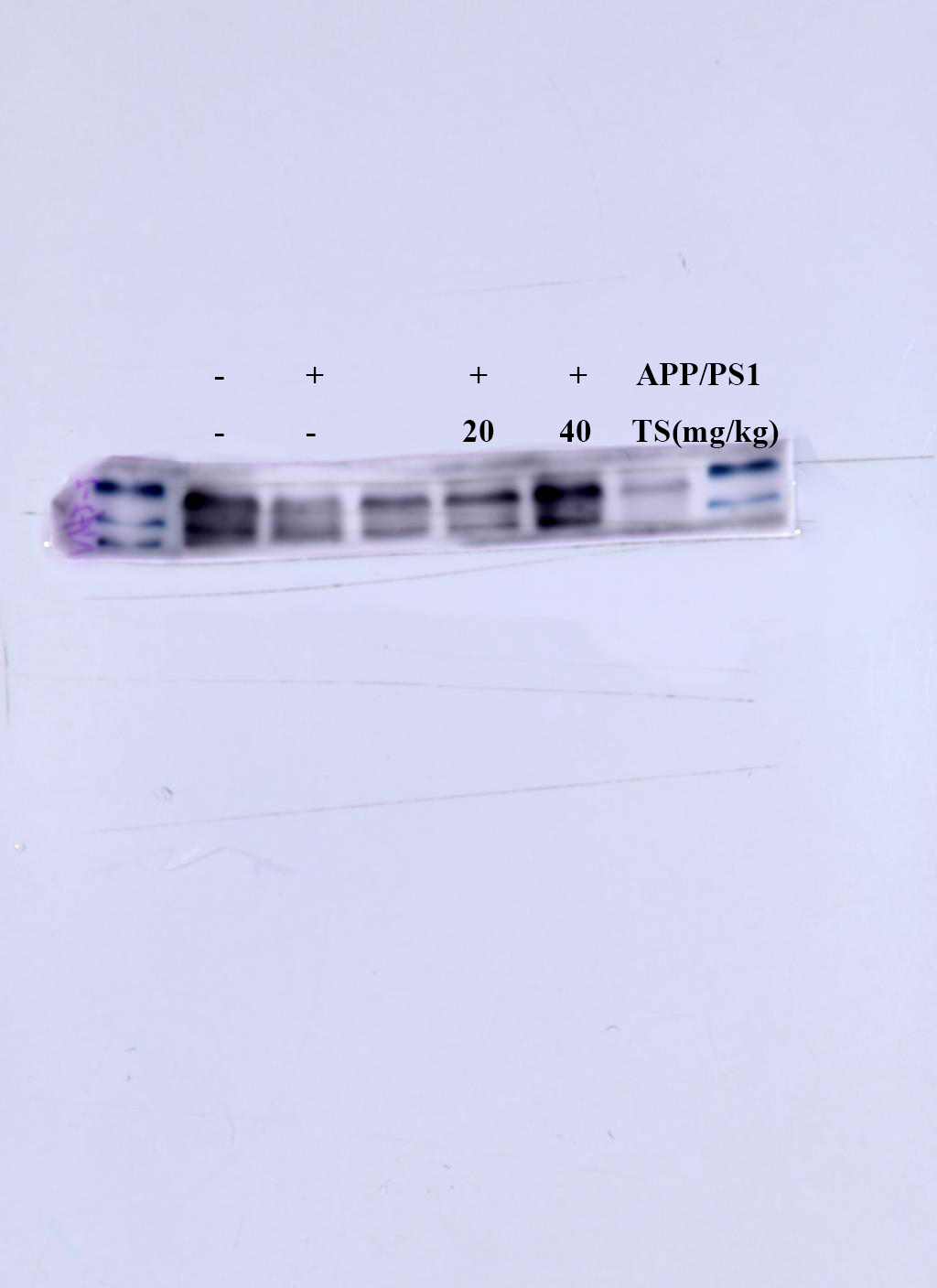

Supplement: Supplementary file 3 [file DataSheet1.ZIP › Fig.4B Nrf2/Fig.4B orginal images.tif]

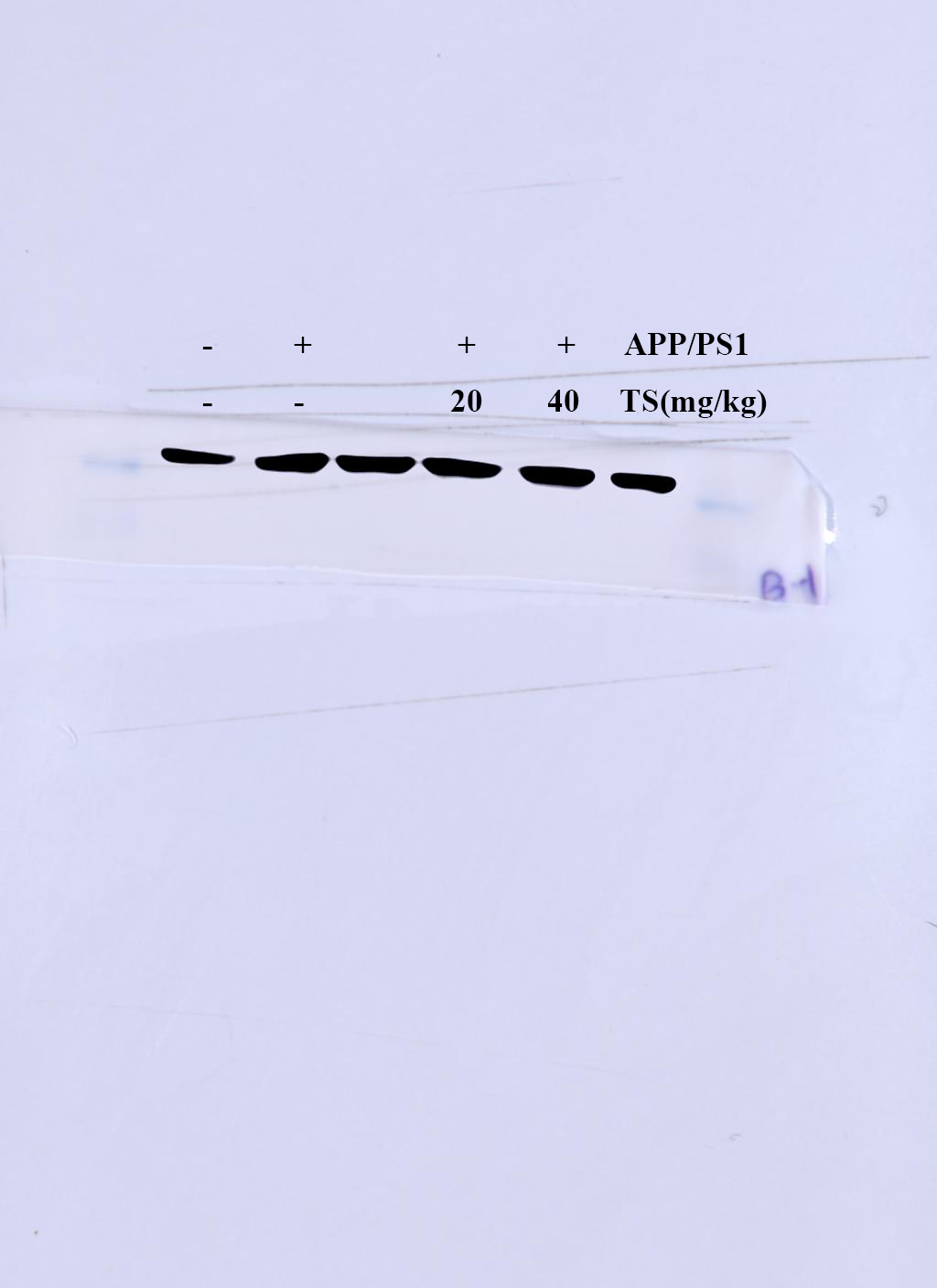

Supplement: Supplementary file 3 [file DataSheet1.ZIP › Fig.4B a┬-actin/Fig.4B ofginal for quantitative analysis-2.tif]

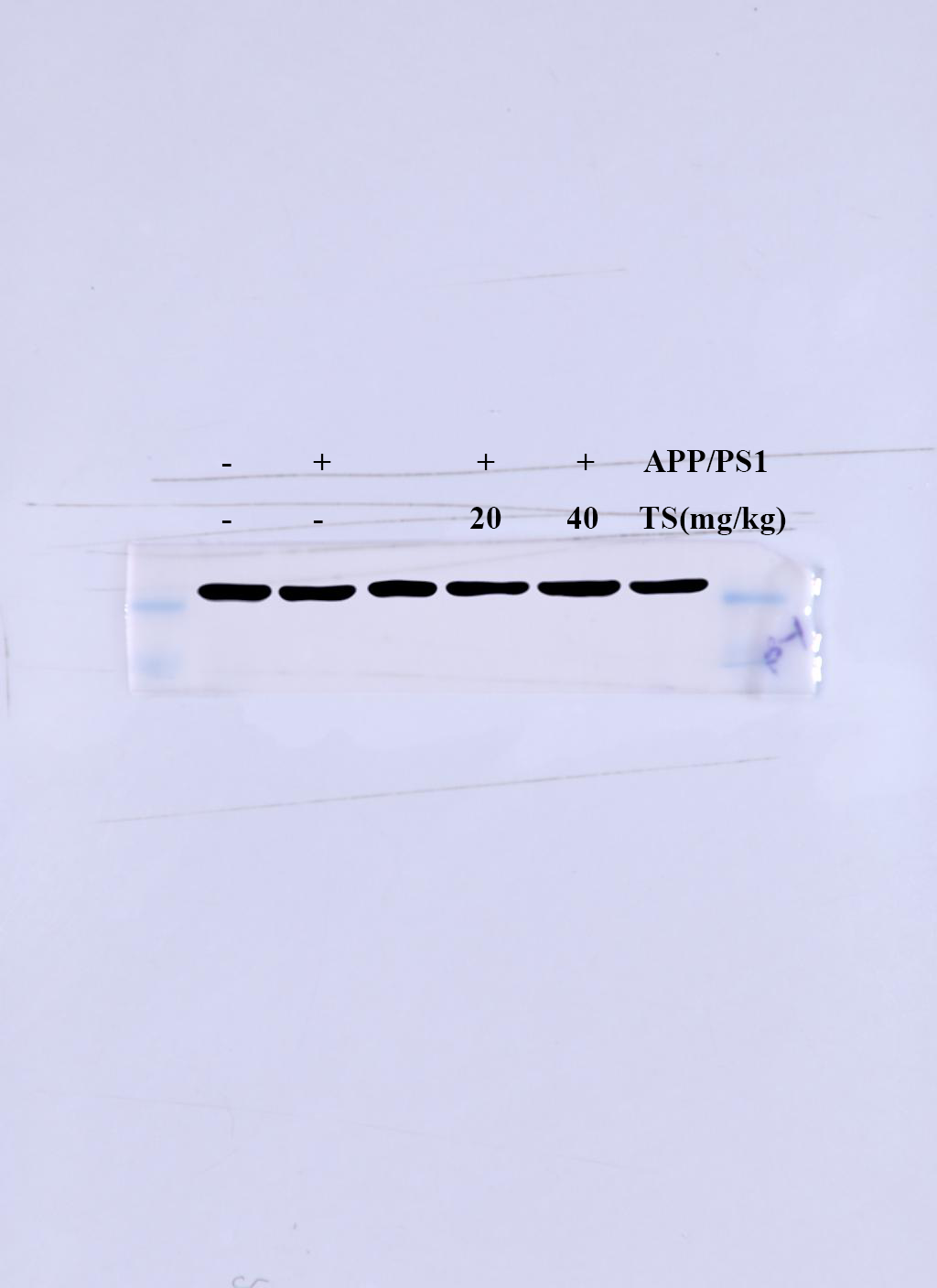

Supplement: Supplementary file 3 [file DataSheet1.ZIP › Fig.4B a┬-actin/Fig.4B orginal for quantitative analysis-1.tif]

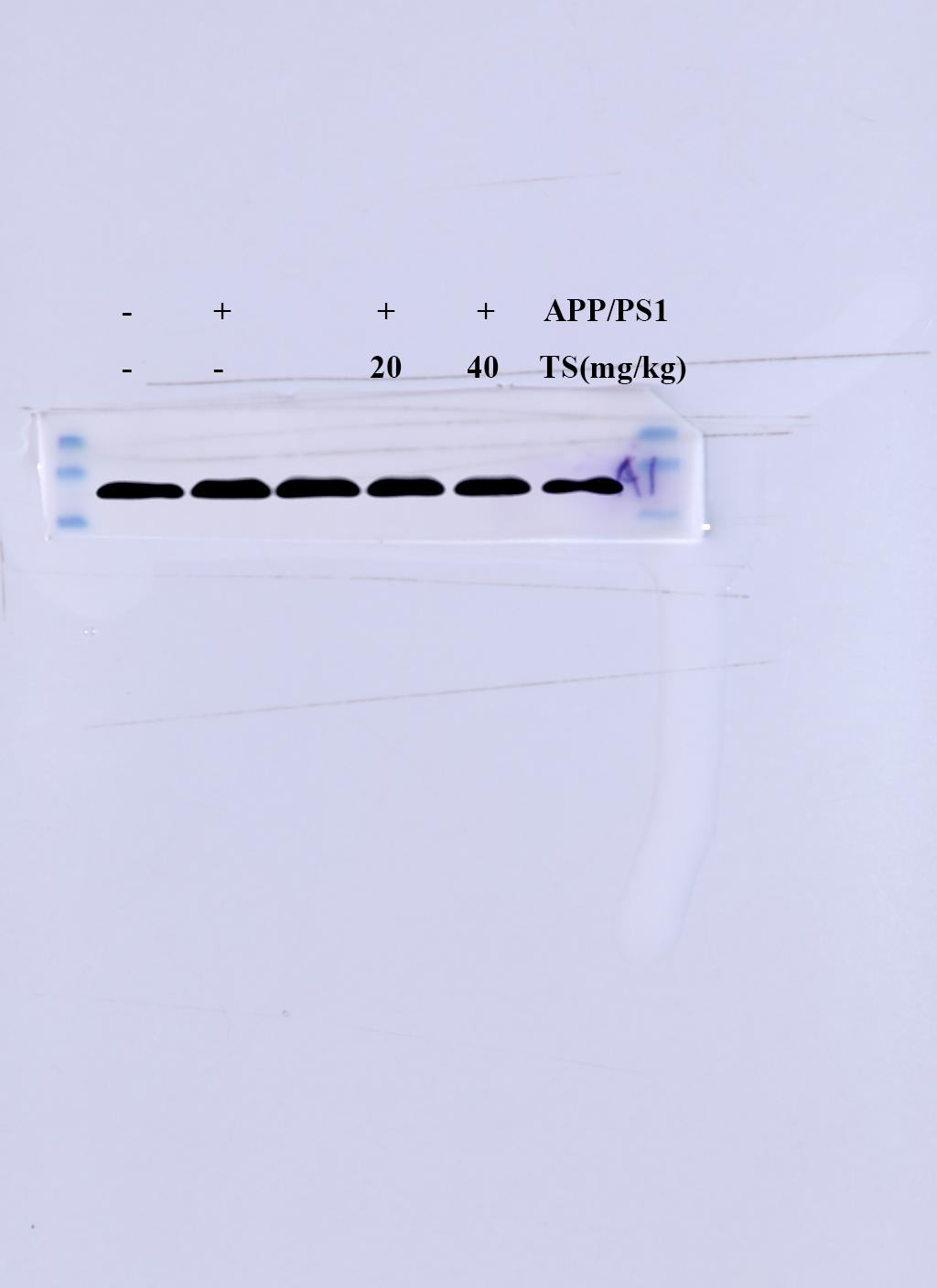

Supplement: Supplementary file 3 [file DataSheet1.ZIP › Fig.4B a┬-actin/Fig.4B orginal images.tif]

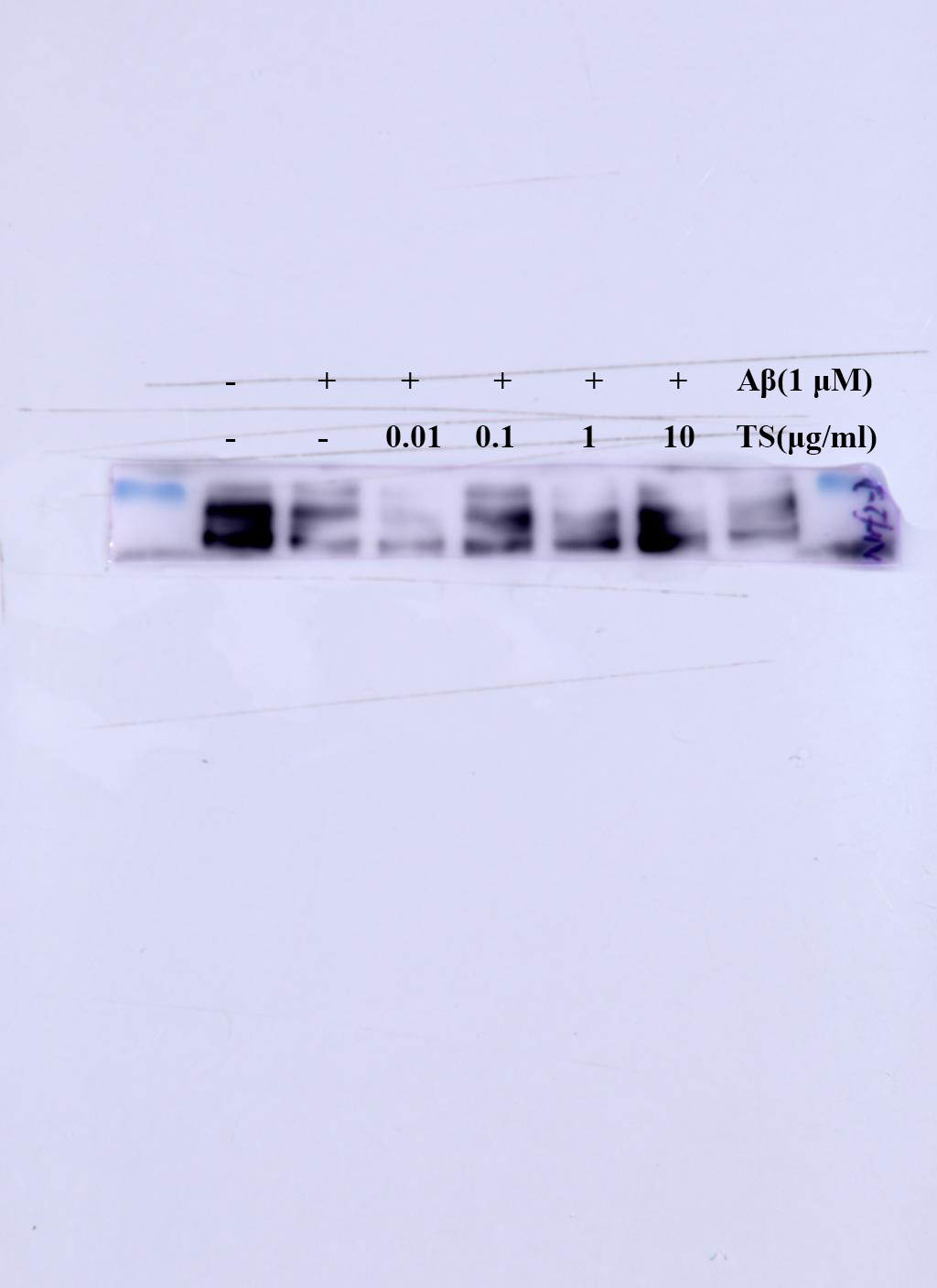

Supplement: Supplementary file 3 [file DataSheet1.ZIP › Fig.4E Nrf2/Fig.4E orginal images for quantitative analysis-1.tif]

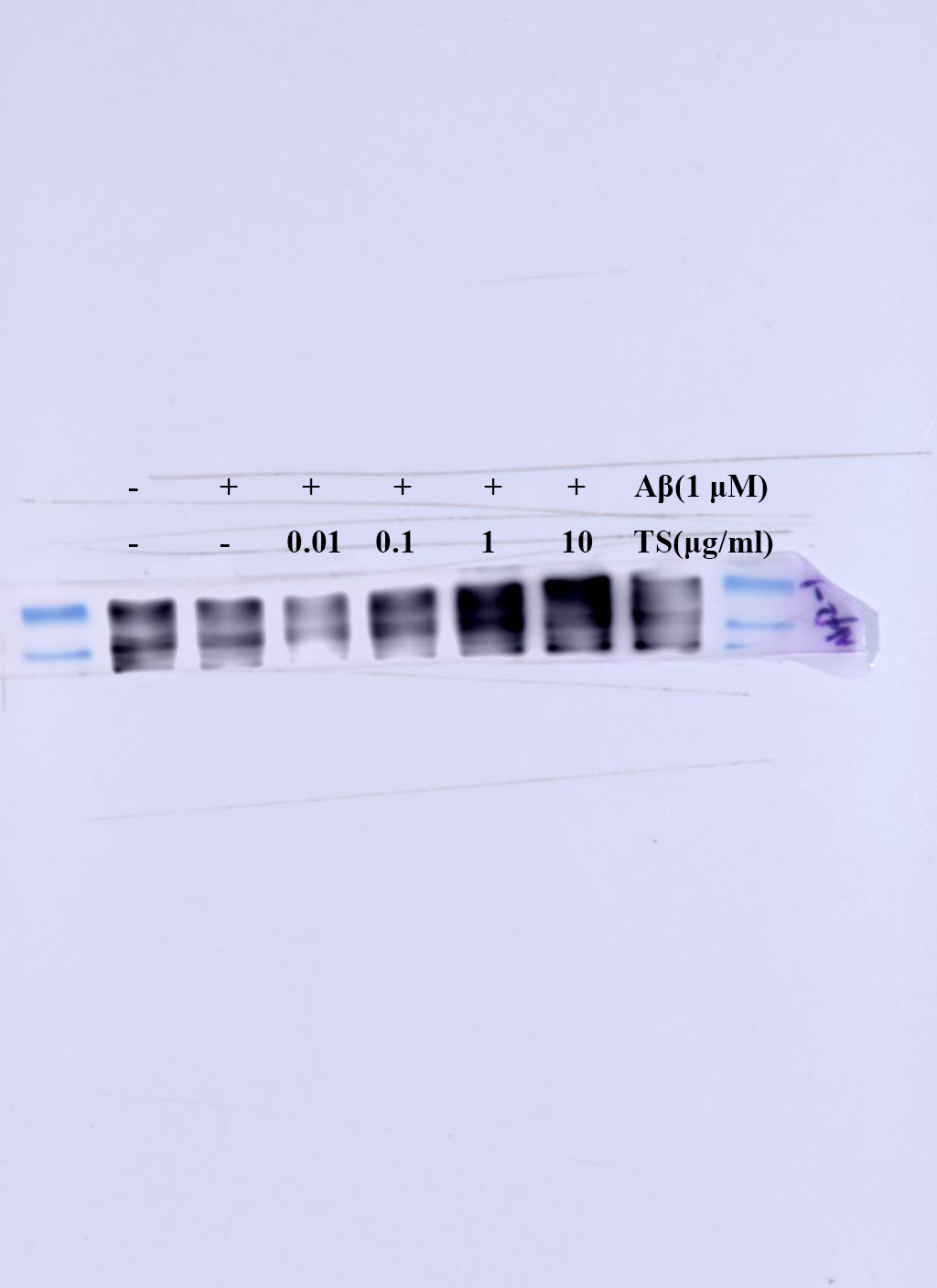

Supplement: Supplementary file 3 [file DataSheet1.ZIP › Fig.4E Nrf2/Fig.4E orginal images for quantitative analysis-2.tif]

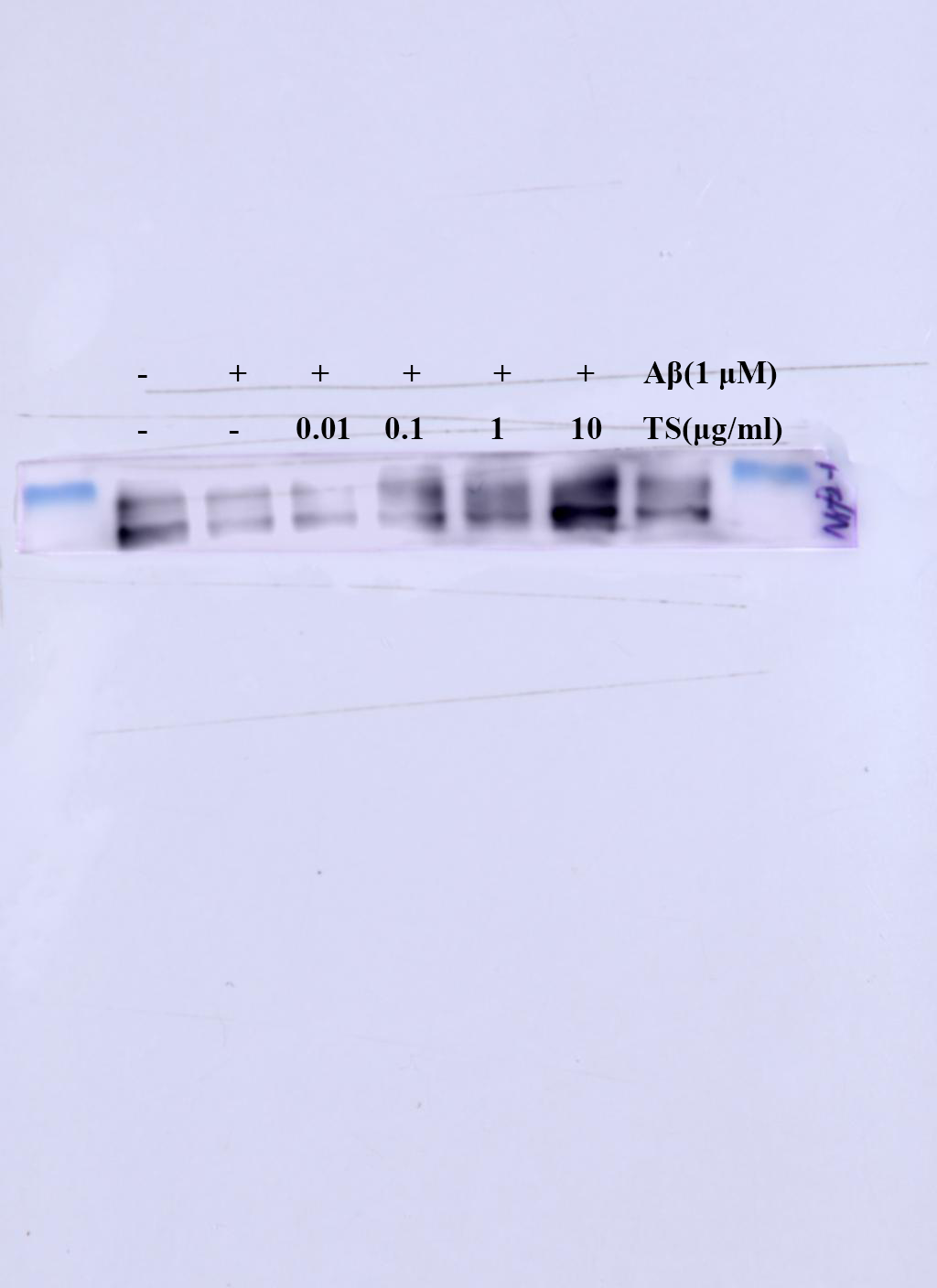

Supplement: Supplementary file 3 [file DataSheet1.ZIP › Fig.4E Nrf2/Fig.4E orginal images.tif]

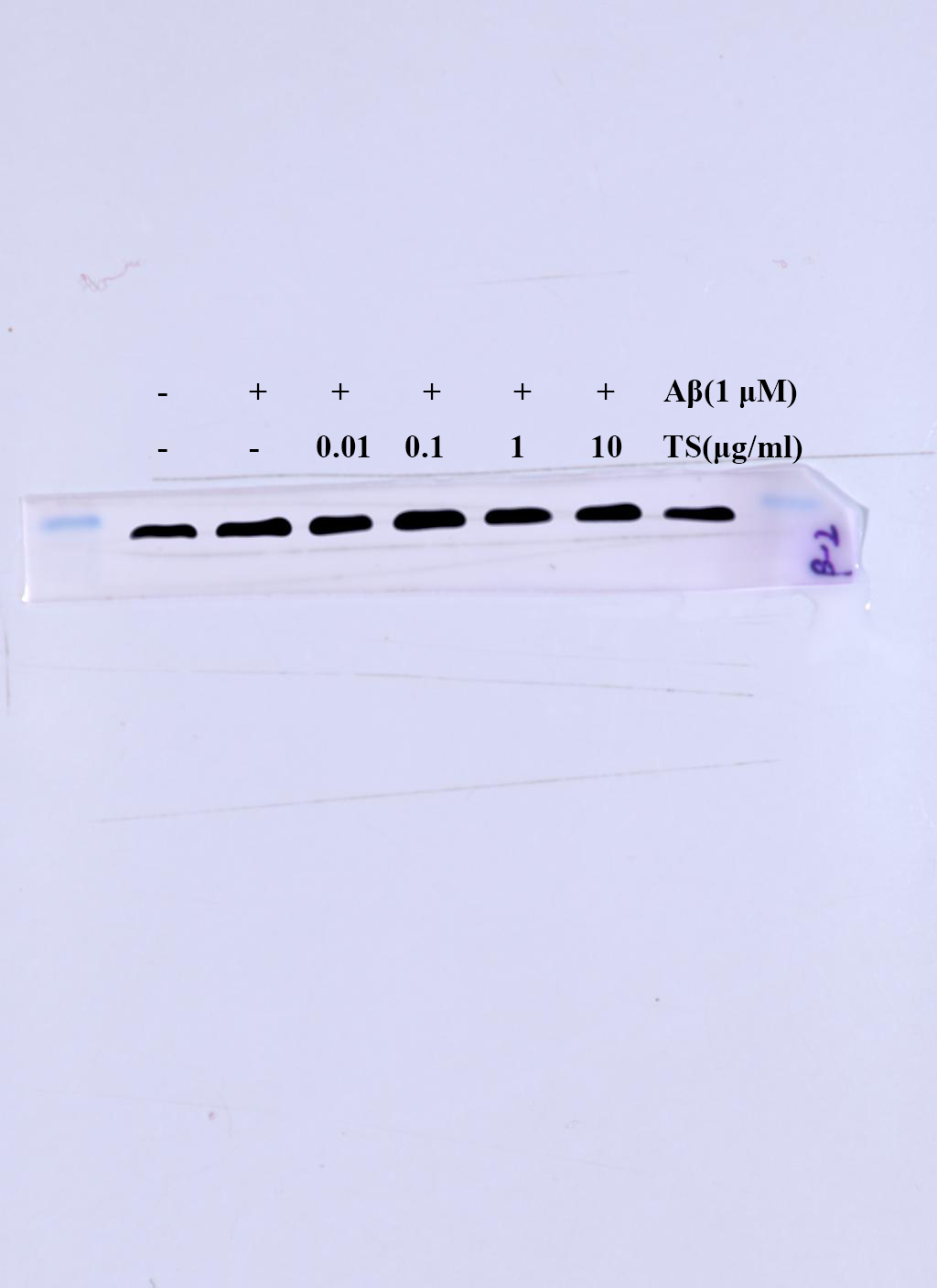

Supplement: Supplementary file 3 [file DataSheet1.ZIP › Fig.4E a┬-actin/Fig.4E orginal images for quantitative analysis-1.tif]

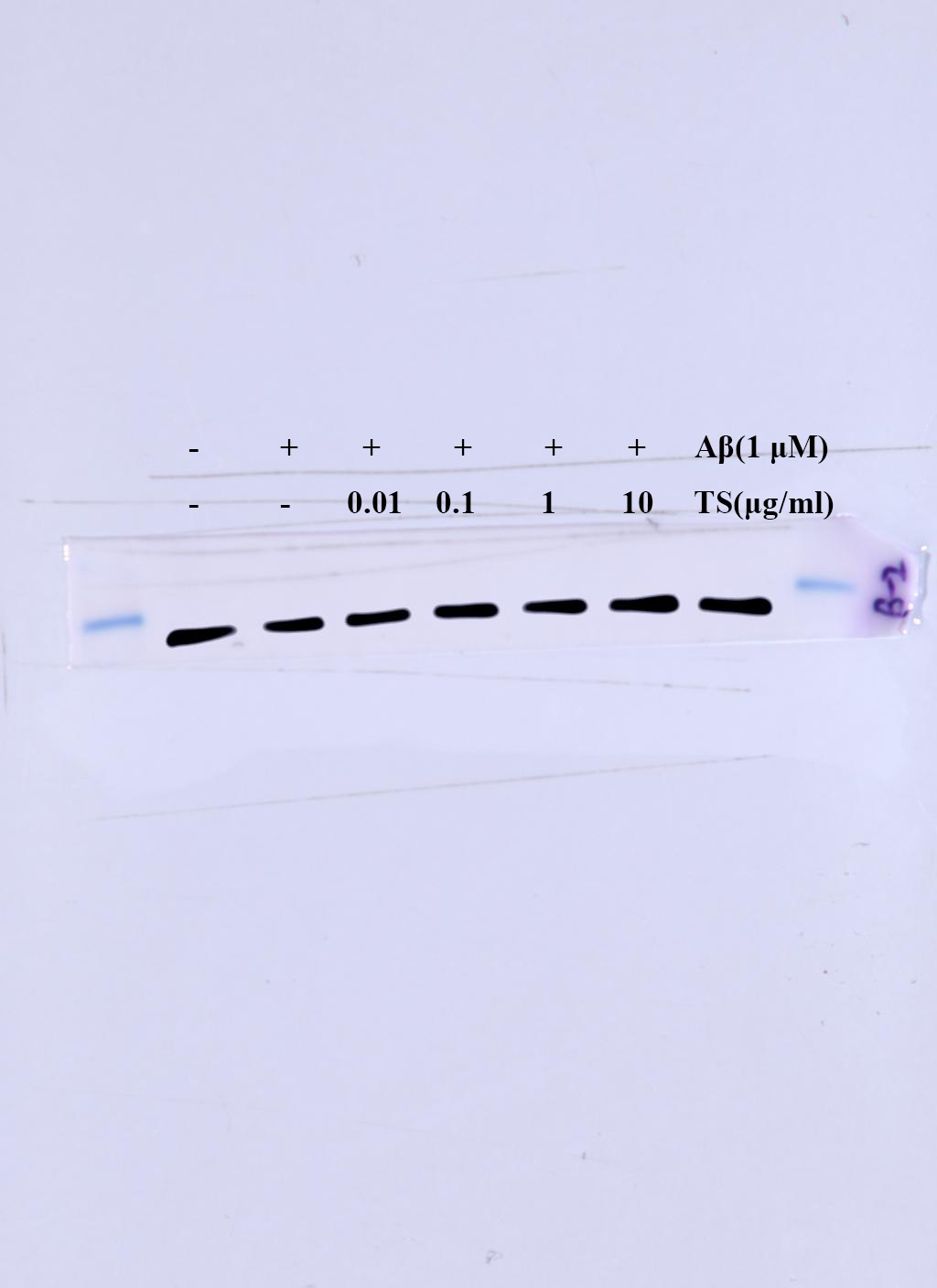

Supplement: Supplementary file 3 [file DataSheet1.ZIP › Fig.4E a┬-actin/Fig.4E orginal images for quantitative analysis-2.tif]

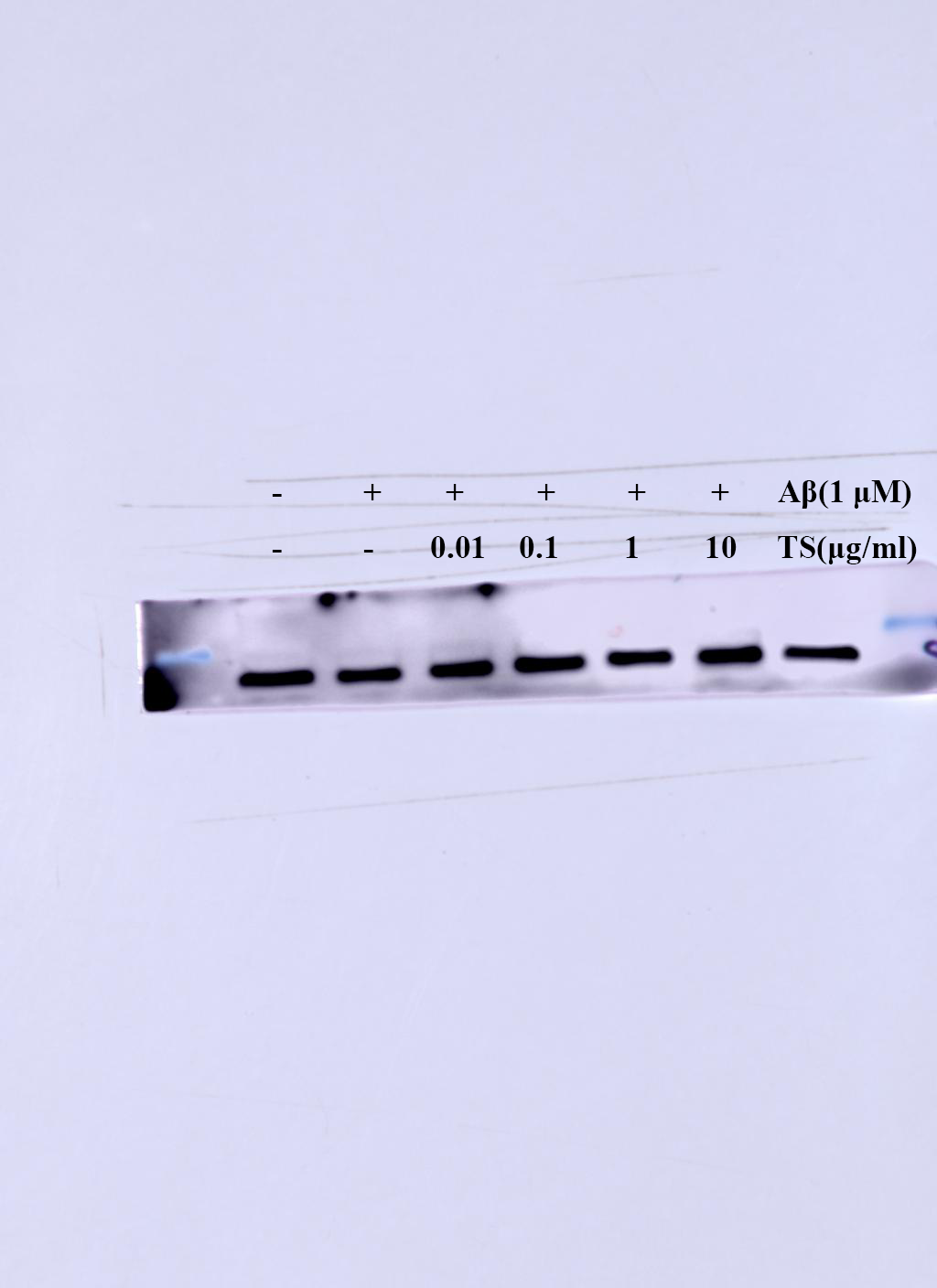

Supplement: Supplementary file 3 [file DataSheet1.ZIP › Fig.4E a┬-actin/Fig.4E orginal images.tif]

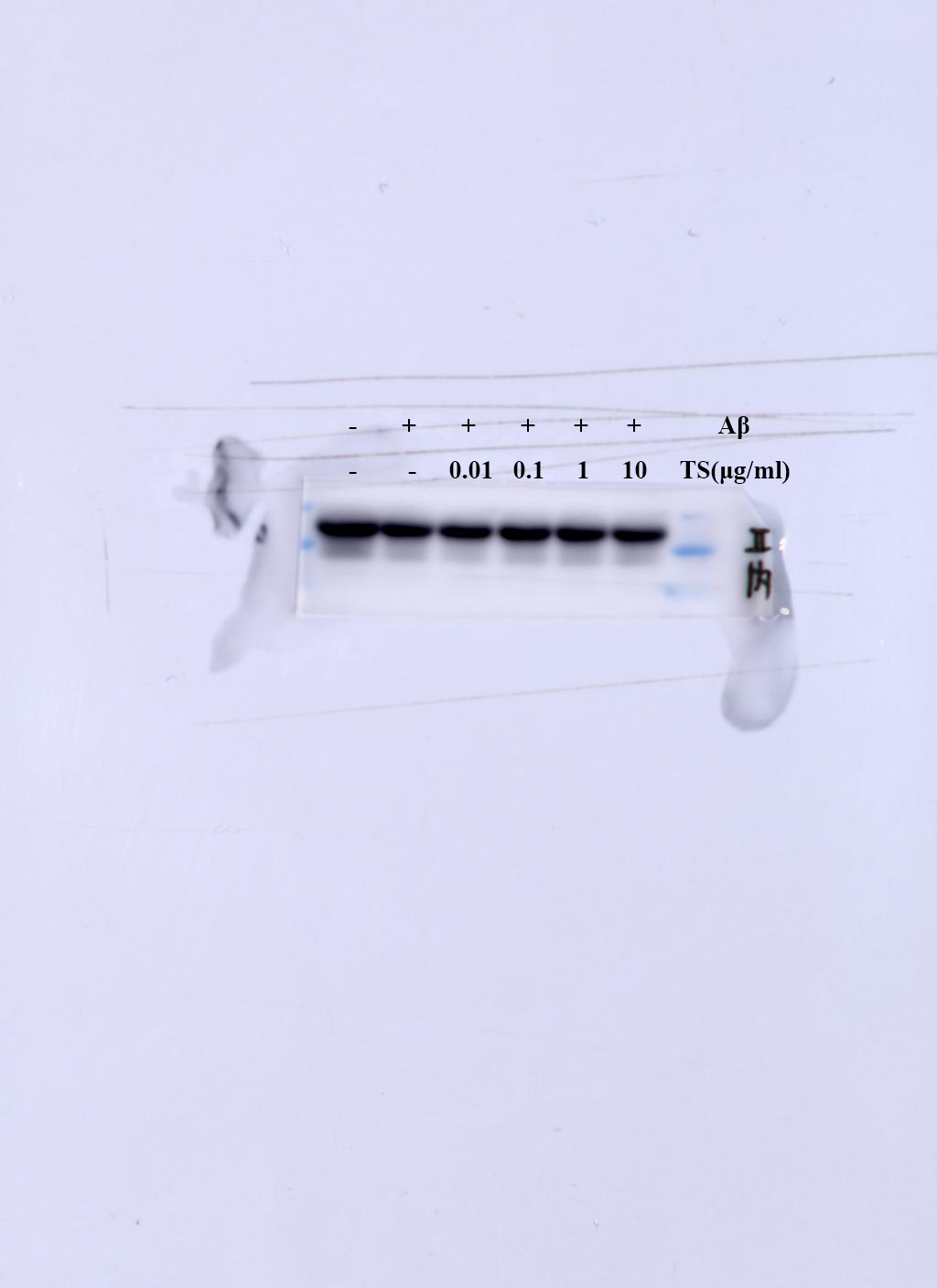

Supplement: Supplementary file 3 [file DataSheet1.ZIP › Fig.4H BACE1/Fig.4H orginal images for quantitative analysis-1-a┬ actin.tif]

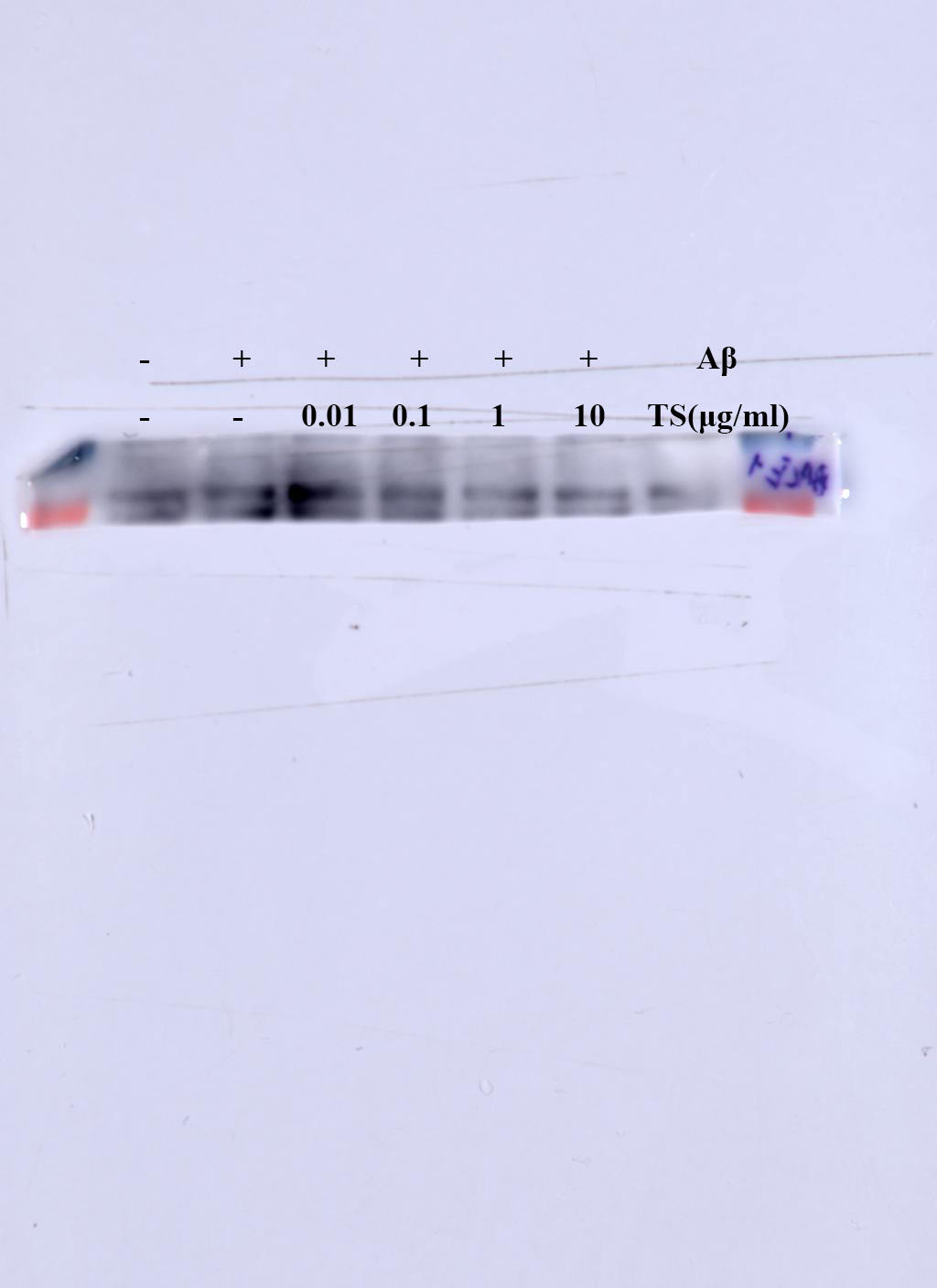

Supplement: Supplementary file 3 [file DataSheet1.ZIP › Fig.4H BACE1/Fig.4H orginal images for quantitative analysis-1.tif]

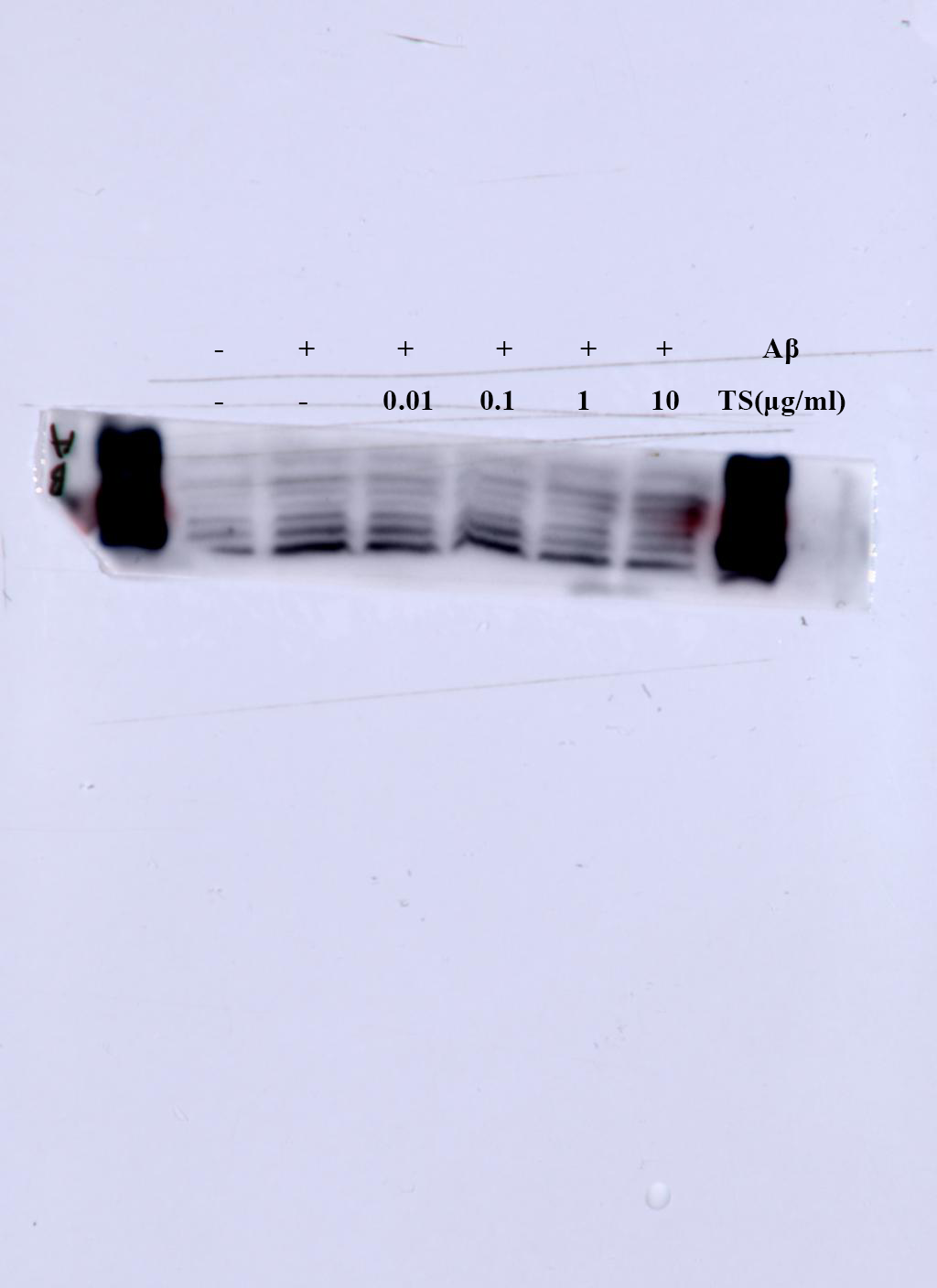

Supplement: Supplementary file 3 [file DataSheet1.ZIP › Fig.4H BACE1/Fig.4H orginal images for quantitative analysis-2.tif]

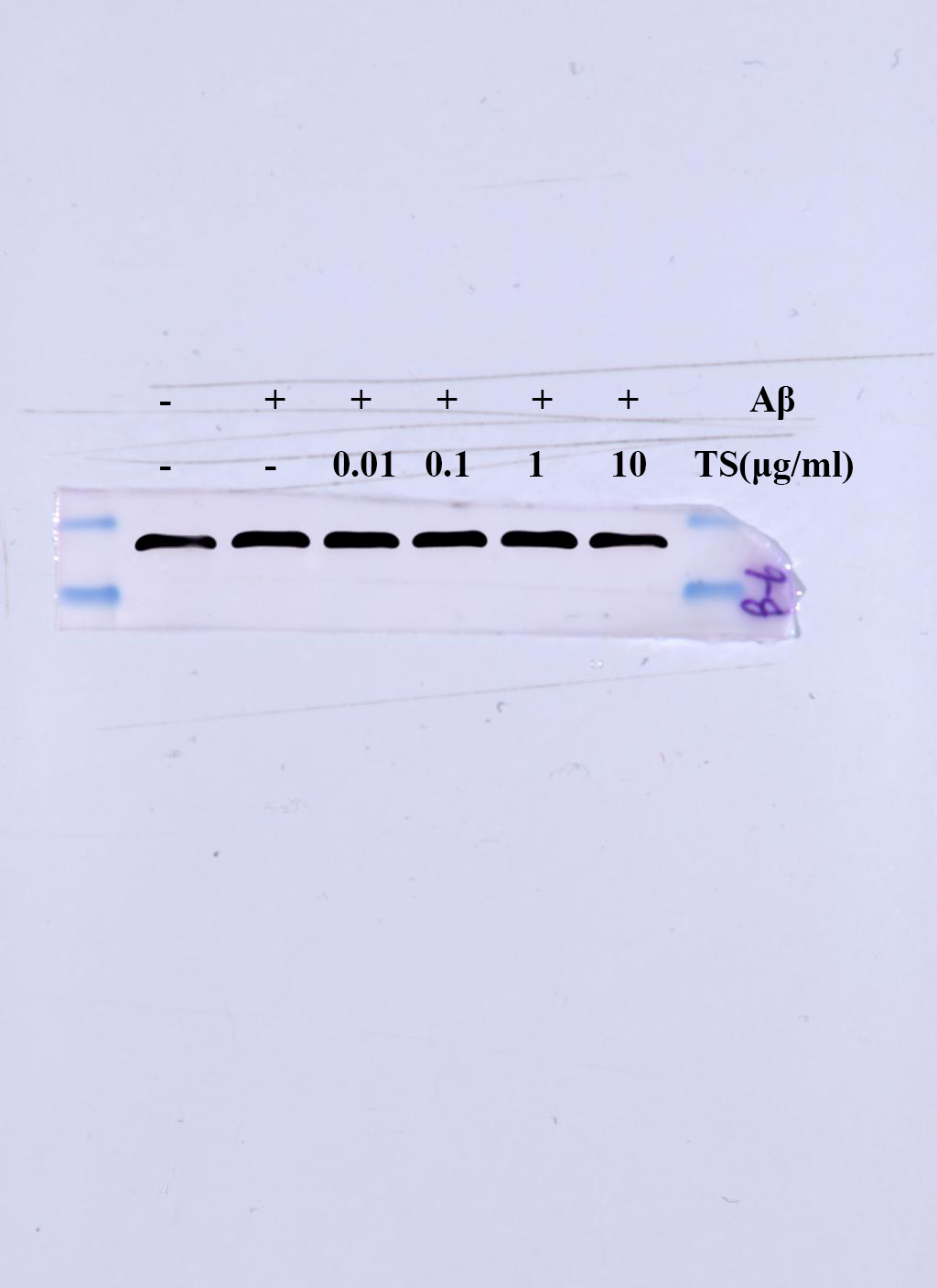

Supplement: Supplementary file 3 [file DataSheet1.ZIP › Fig.4H BACE1/Fig.4H orginal images for quantitative-2-a┬ actin.tif]

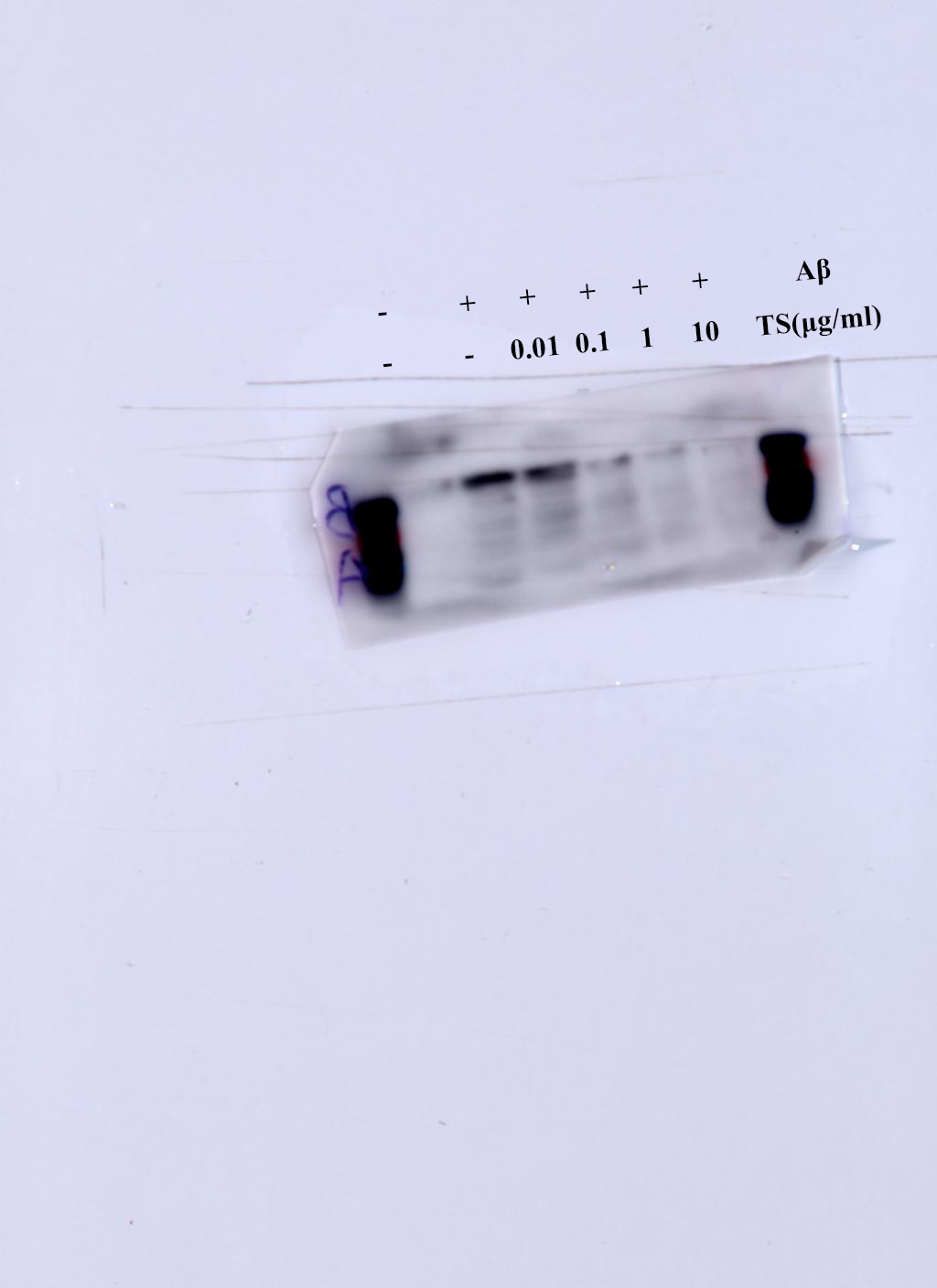

Supplement: Supplementary file 3 [file DataSheet1.ZIP › Fig.4H BACE1/Fig.4H orginal images.tif]

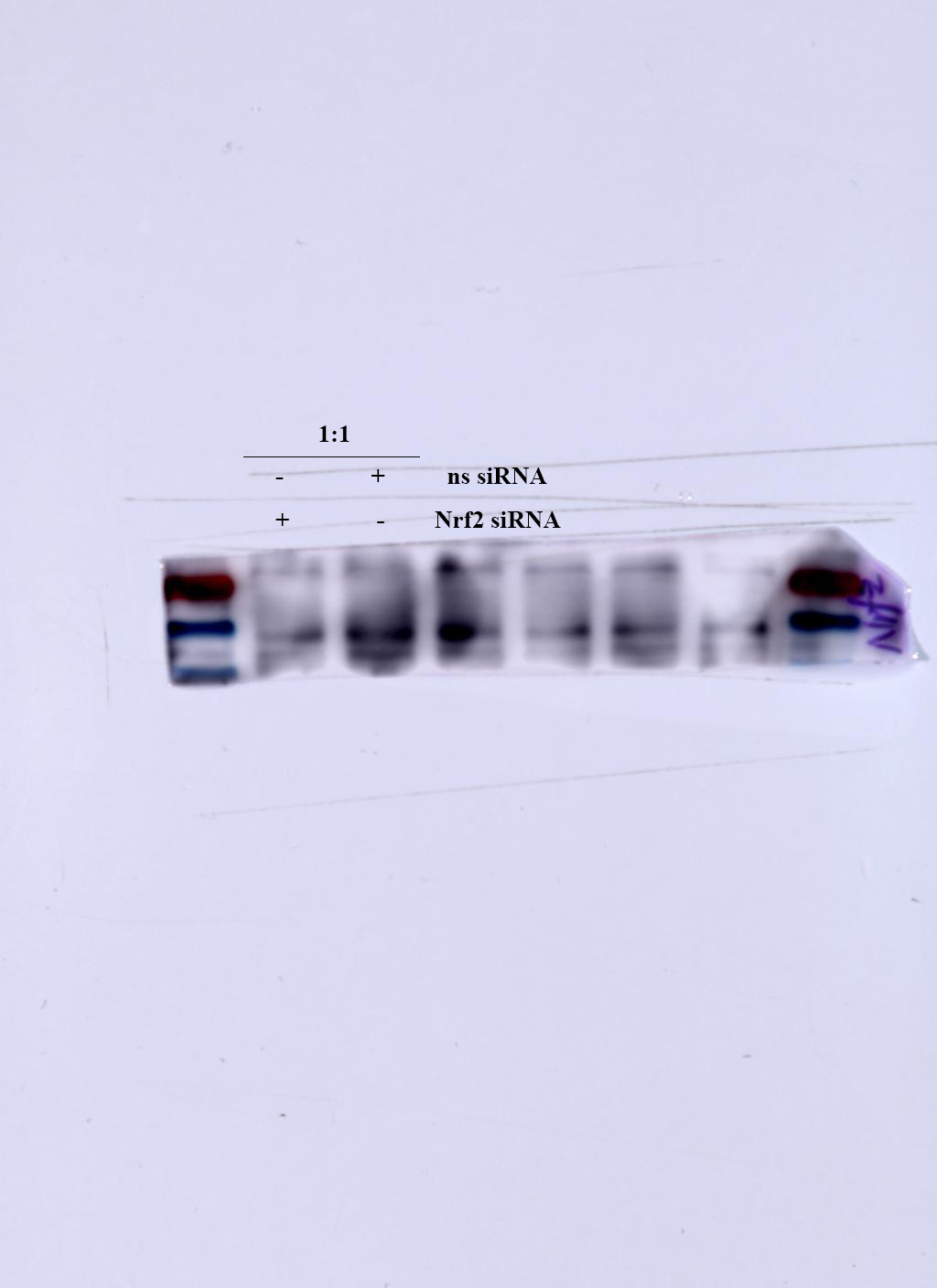

Supplement: Supplementary file 3 [file DataSheet1.ZIP › Fig.4H Nrf2/Fig.4H orginal images for quantitative analysis-1.tif]

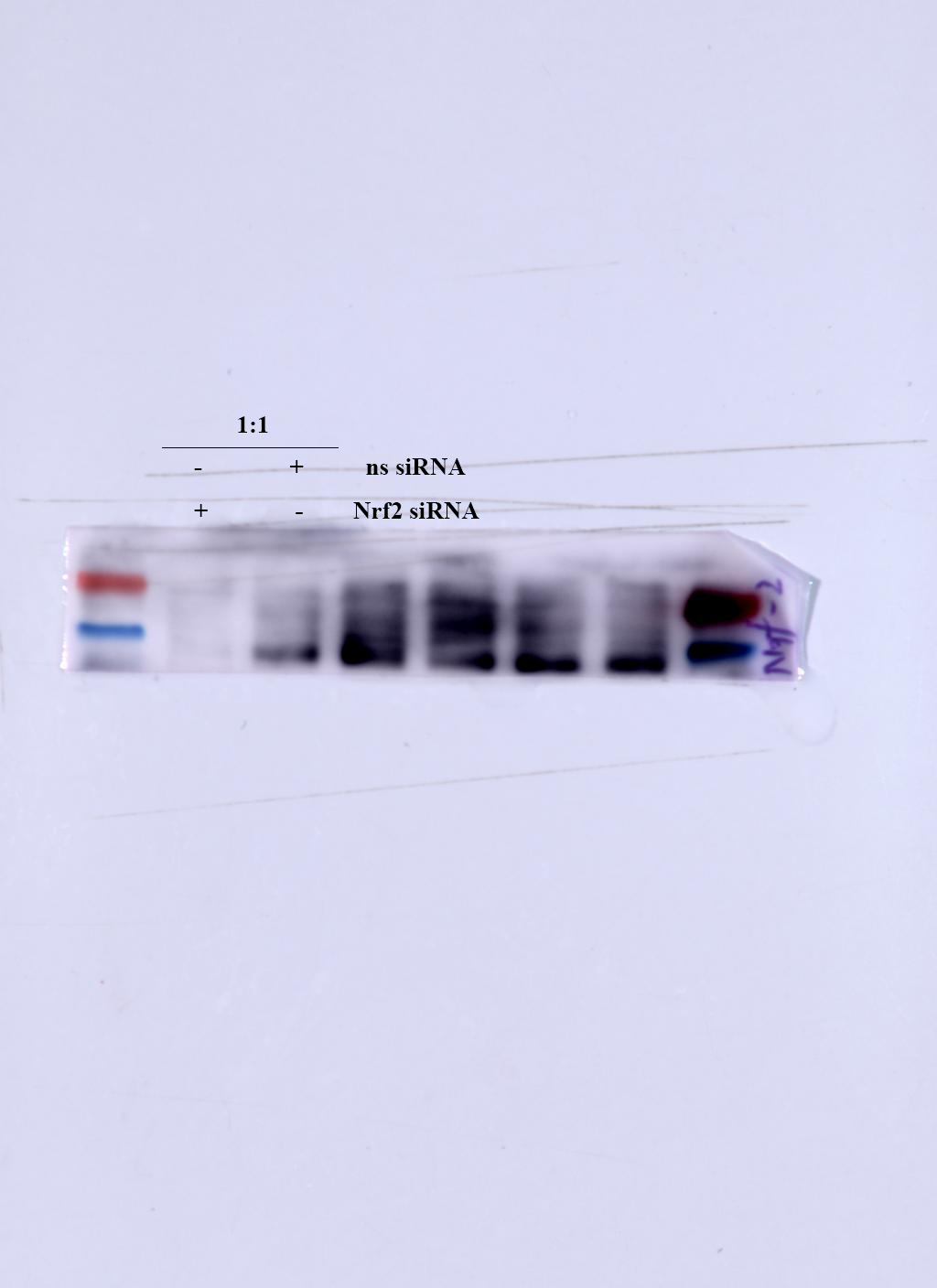

Supplement: Supplementary file 3 [file DataSheet1.ZIP › Fig.4H Nrf2/Fig.4H orginal images for quantitative analysis-2.tif]

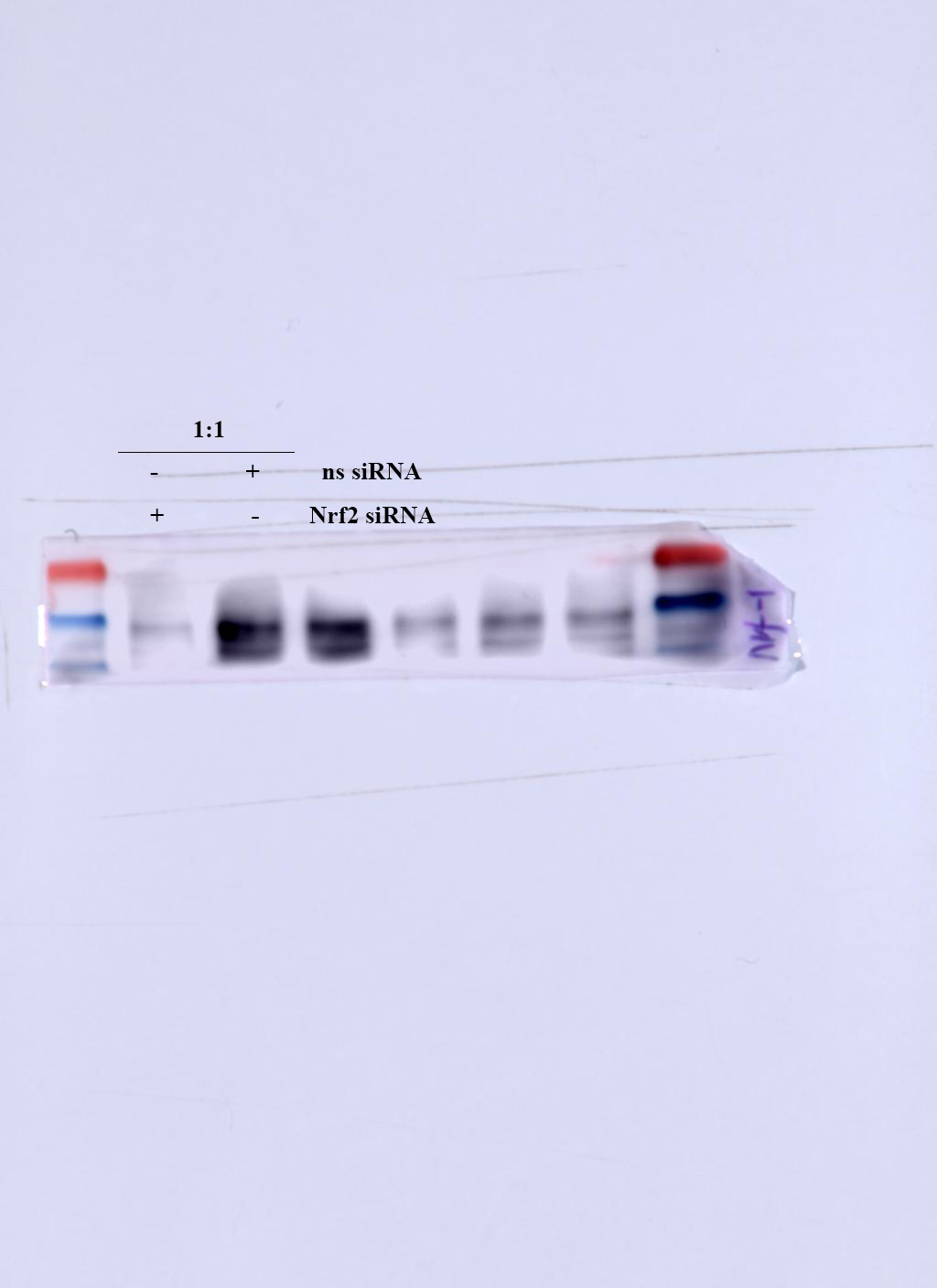

Supplement: Supplementary file 3 [file DataSheet1.ZIP › Fig.4H Nrf2/Fig.4H orginal images.tif]

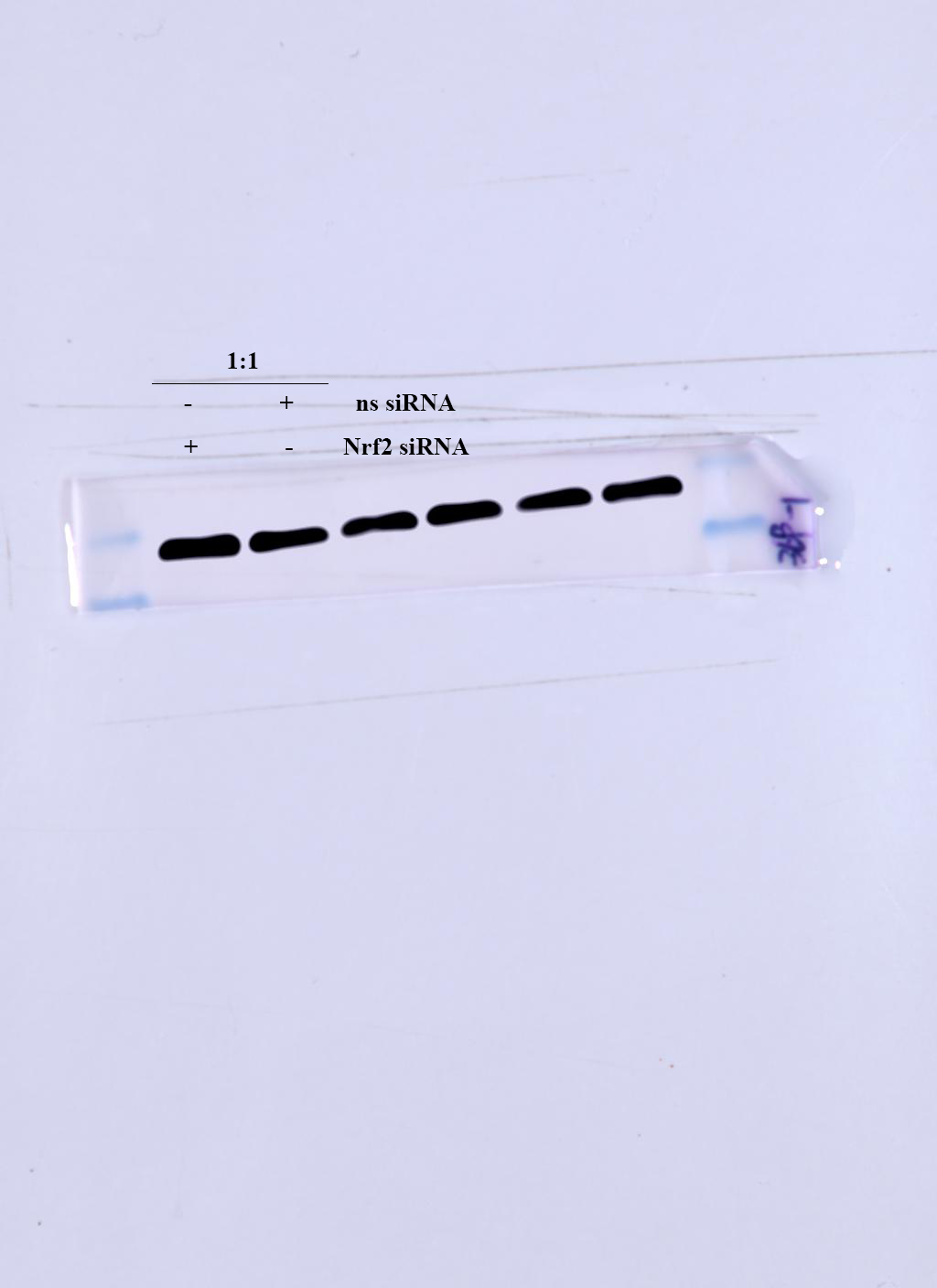

Supplement: Supplementary file 3 [file DataSheet1.ZIP › Fig.4H a┬-actin/Fig.4H orginal images for quantitative analysis-1.tif]

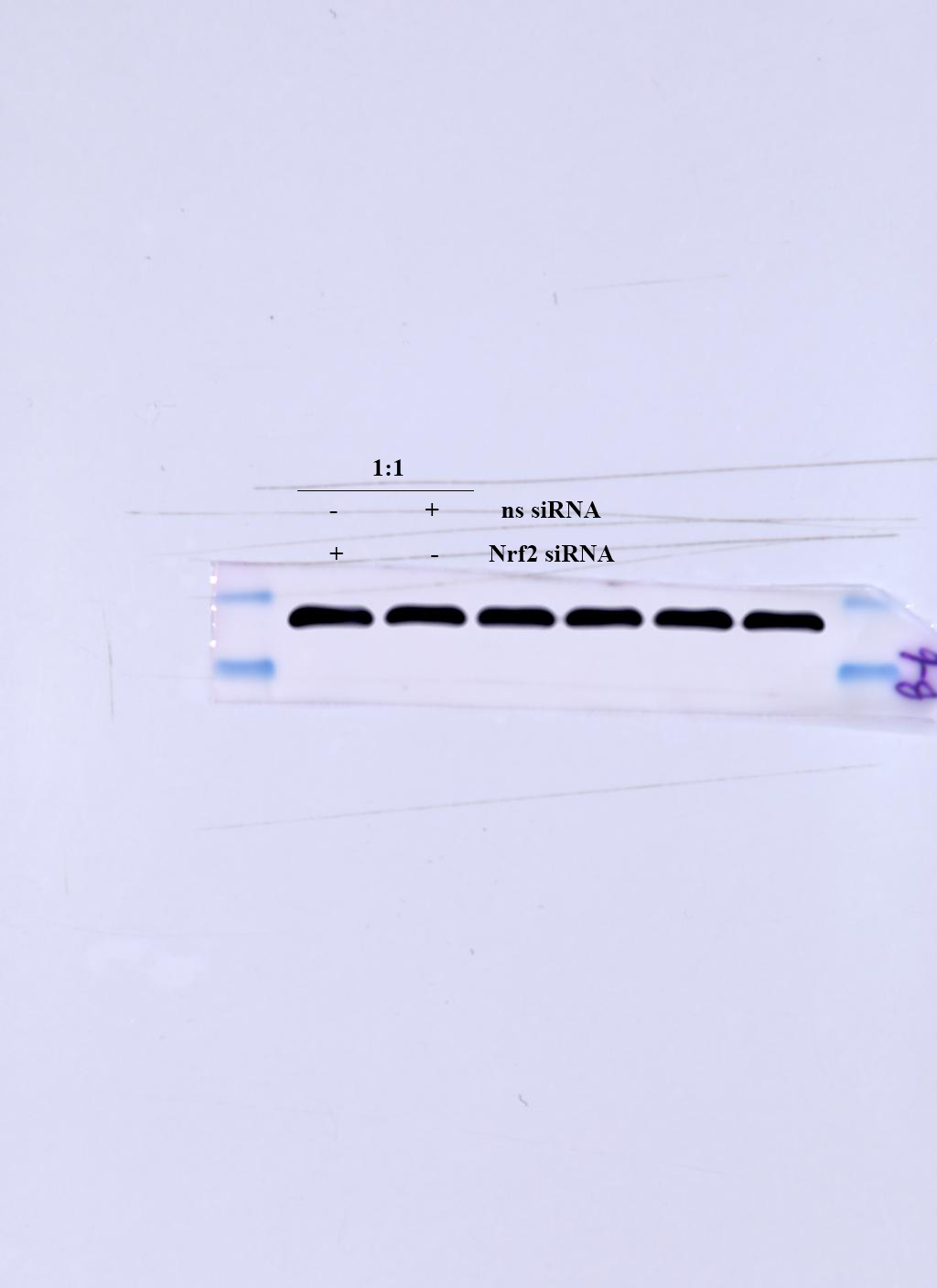

Supplement: Supplementary file 3 [file DataSheet1.ZIP › Fig.4H a┬-actin/Fig.4H orginal images for quantitative analysis-2.tif]

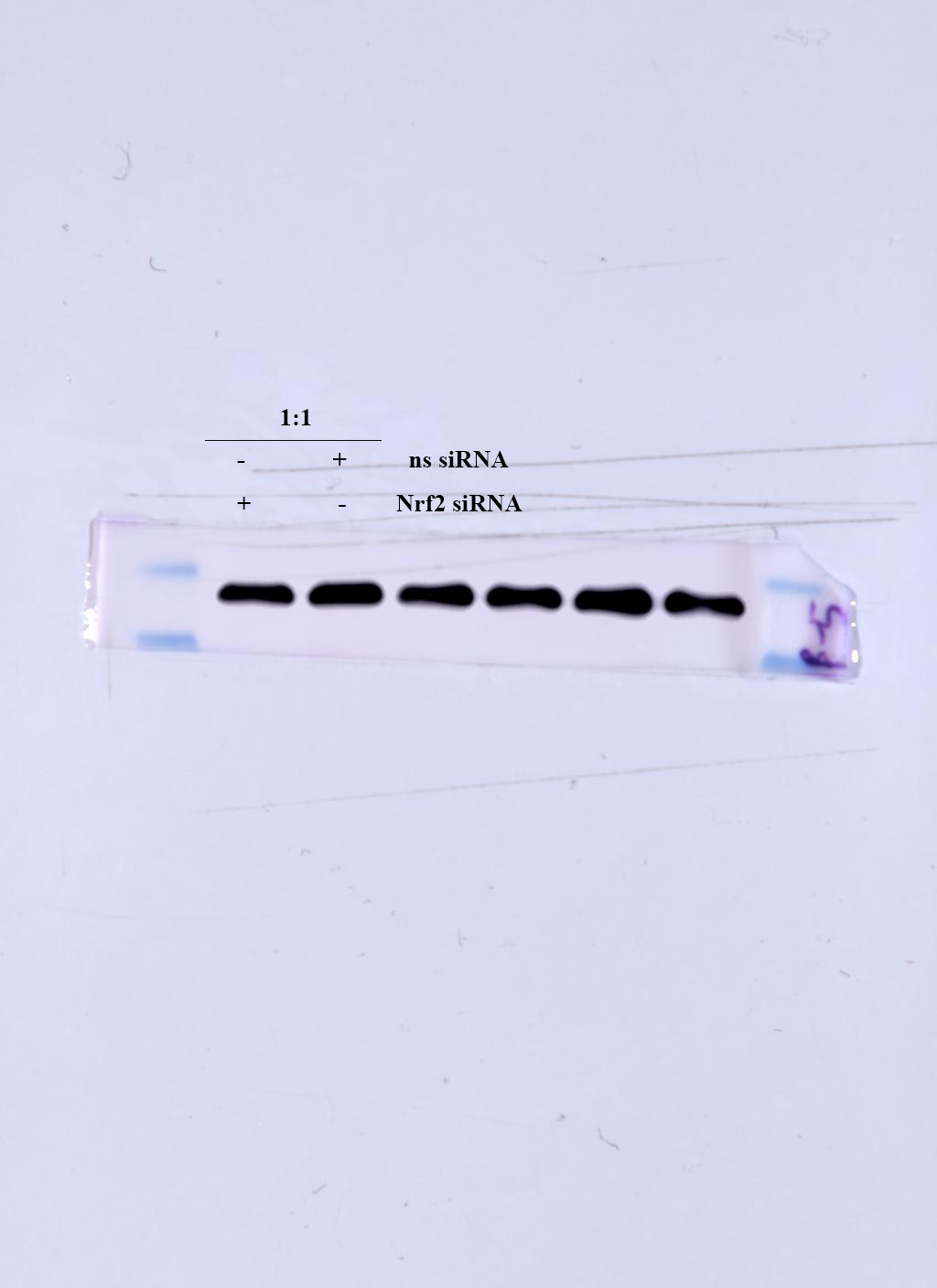

Supplement: Supplementary file 3 [file DataSheet1.ZIP › Fig.4H a┬-actin/Fig.4H orginal images.tif]

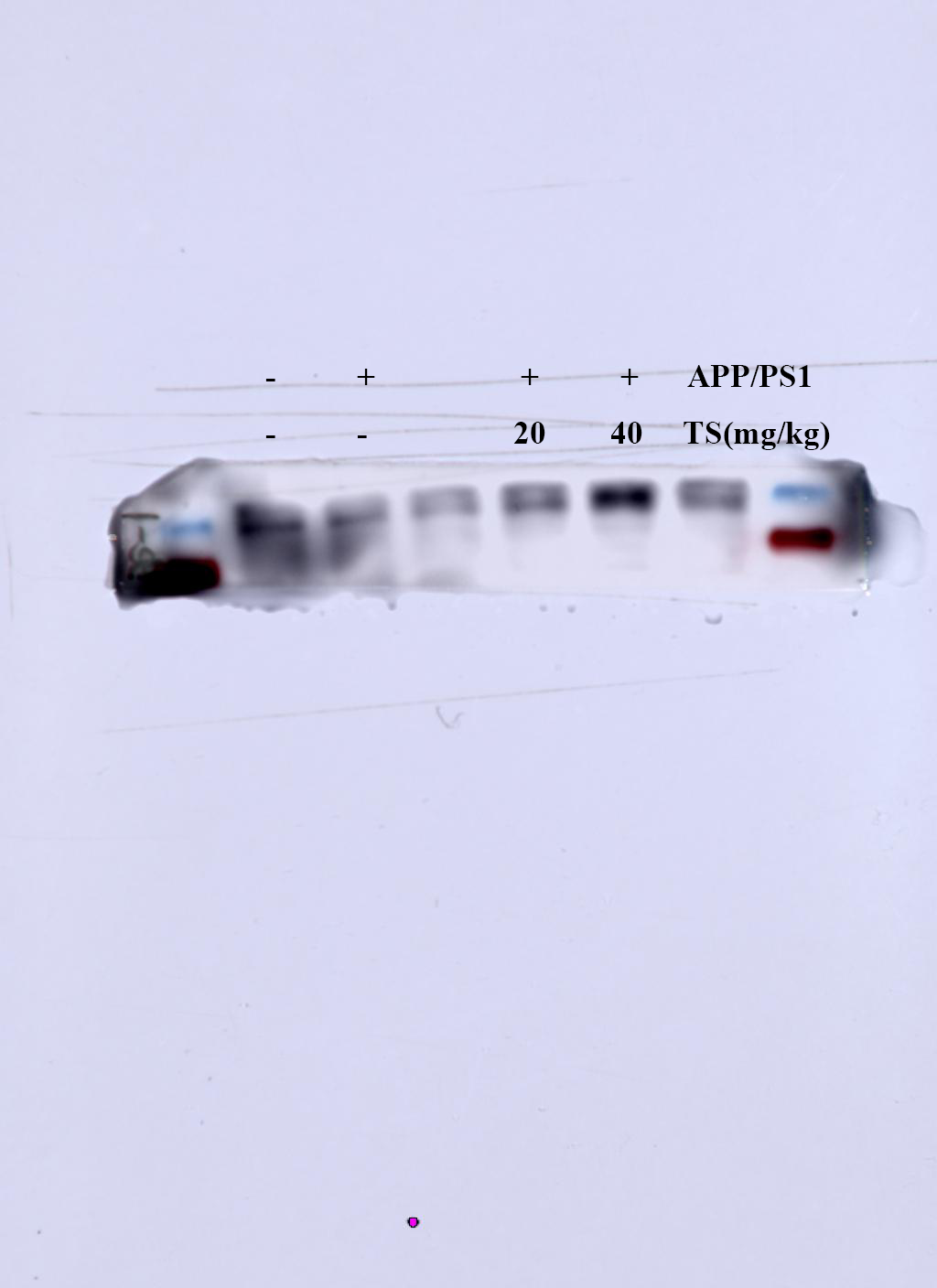

Supplement: Supplementary file 4 [file DataSheet2.ZIP › Fig.5D Beclin1/Fig.5D orginal images for quantitative analysis-1.tif]

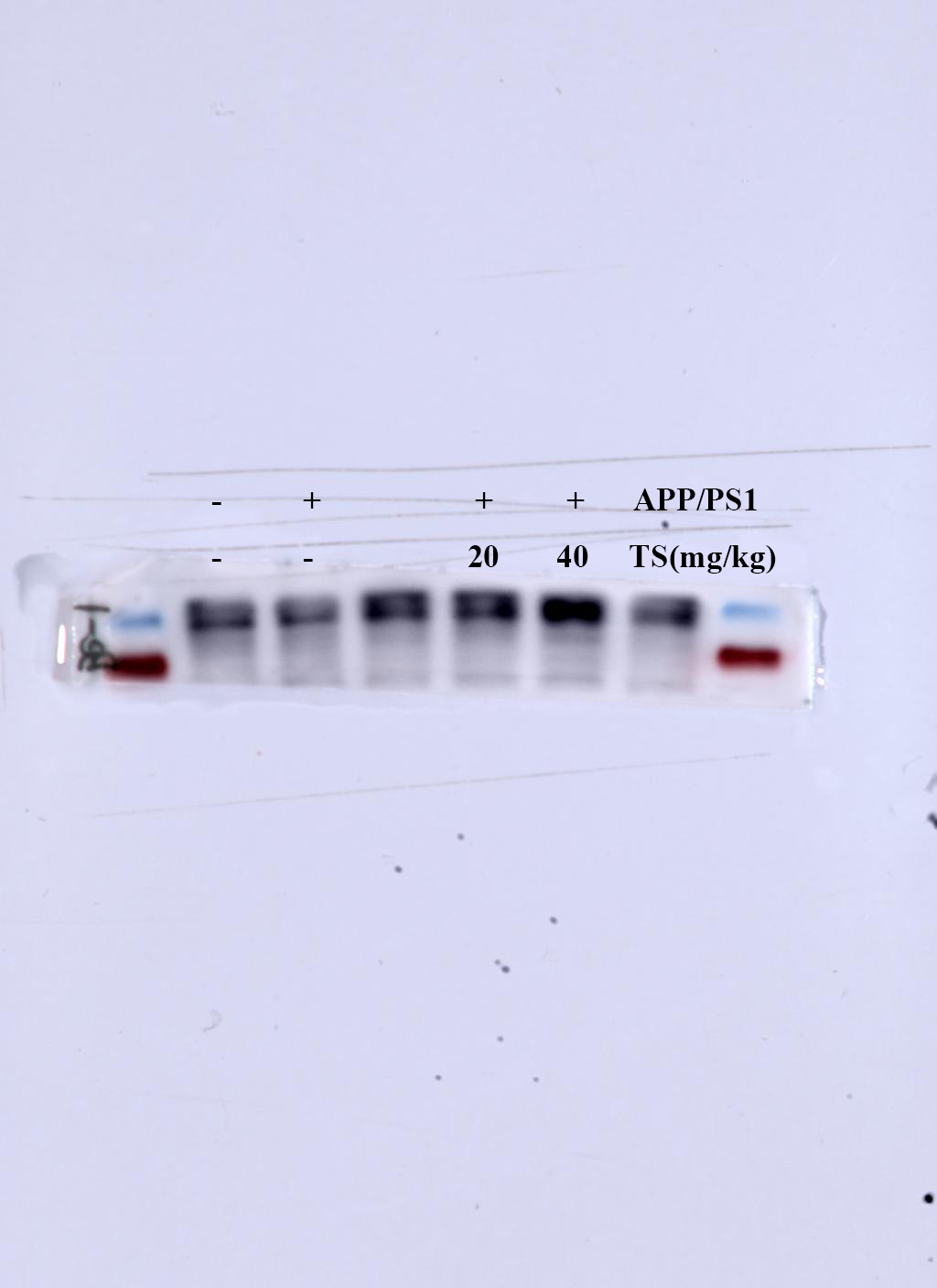

Supplement: Supplementary file 4 [file DataSheet2.ZIP › Fig.5D Beclin1/Fig.5D orginal images for quantitative analysis-2.tif]

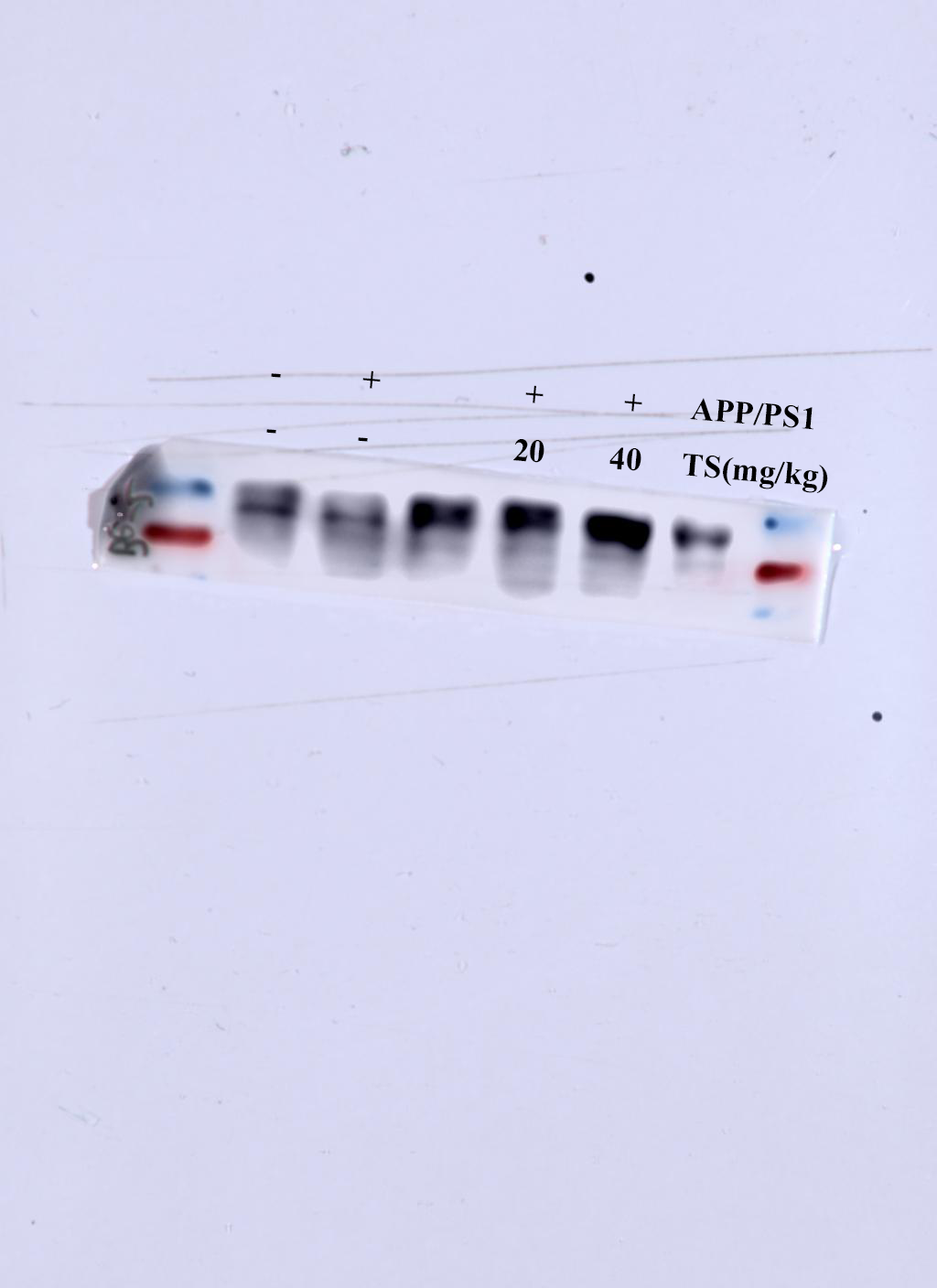

Supplement: Supplementary file 4 [file DataSheet2.ZIP › Fig.5D Beclin1/Fig.5D orginal images.tif]

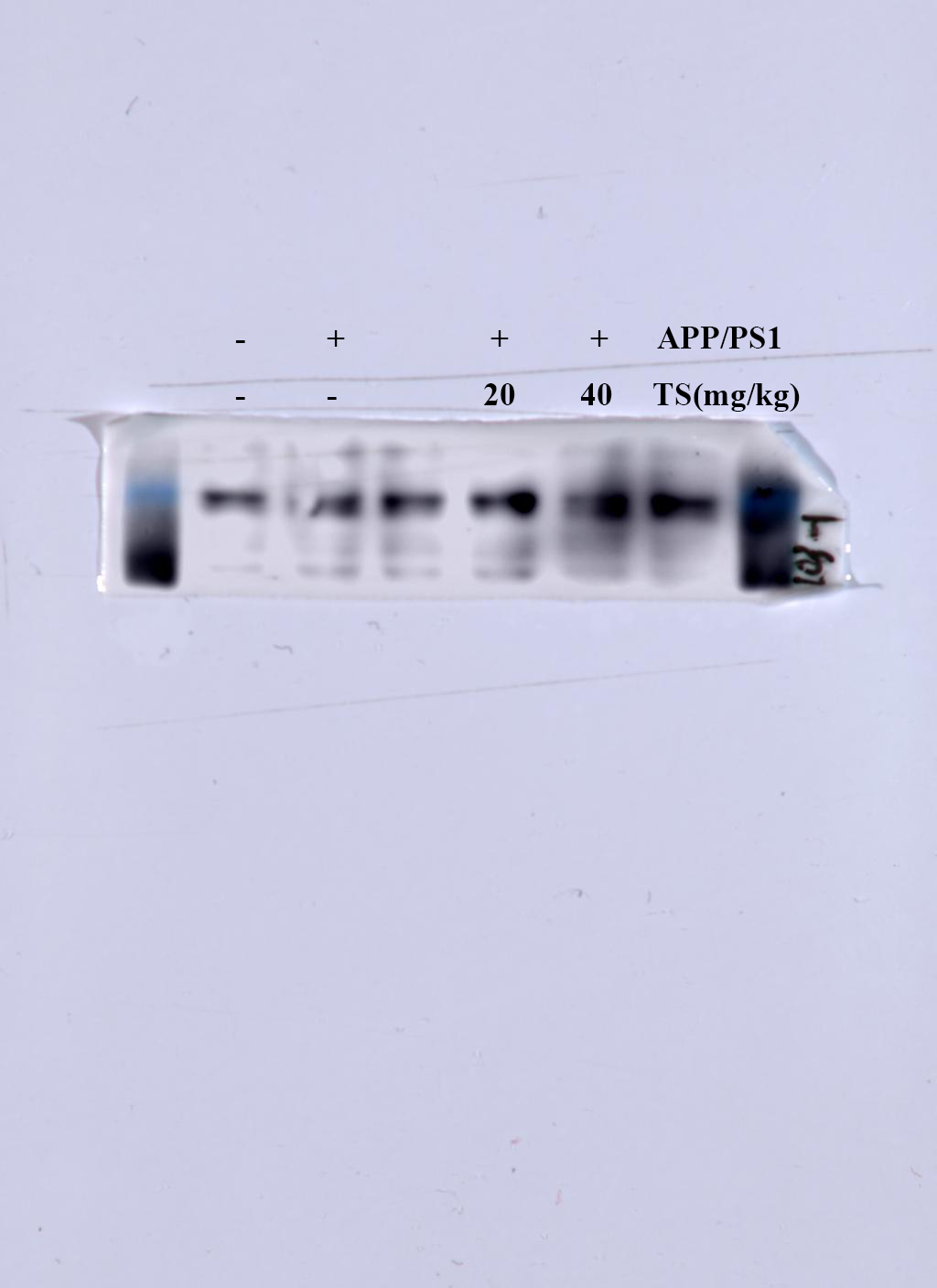

Supplement: Supplementary file 4 [file DataSheet2.ZIP › Fig.5D LC3/Fig.5H orginal images for quantitative analysis-1.tif]

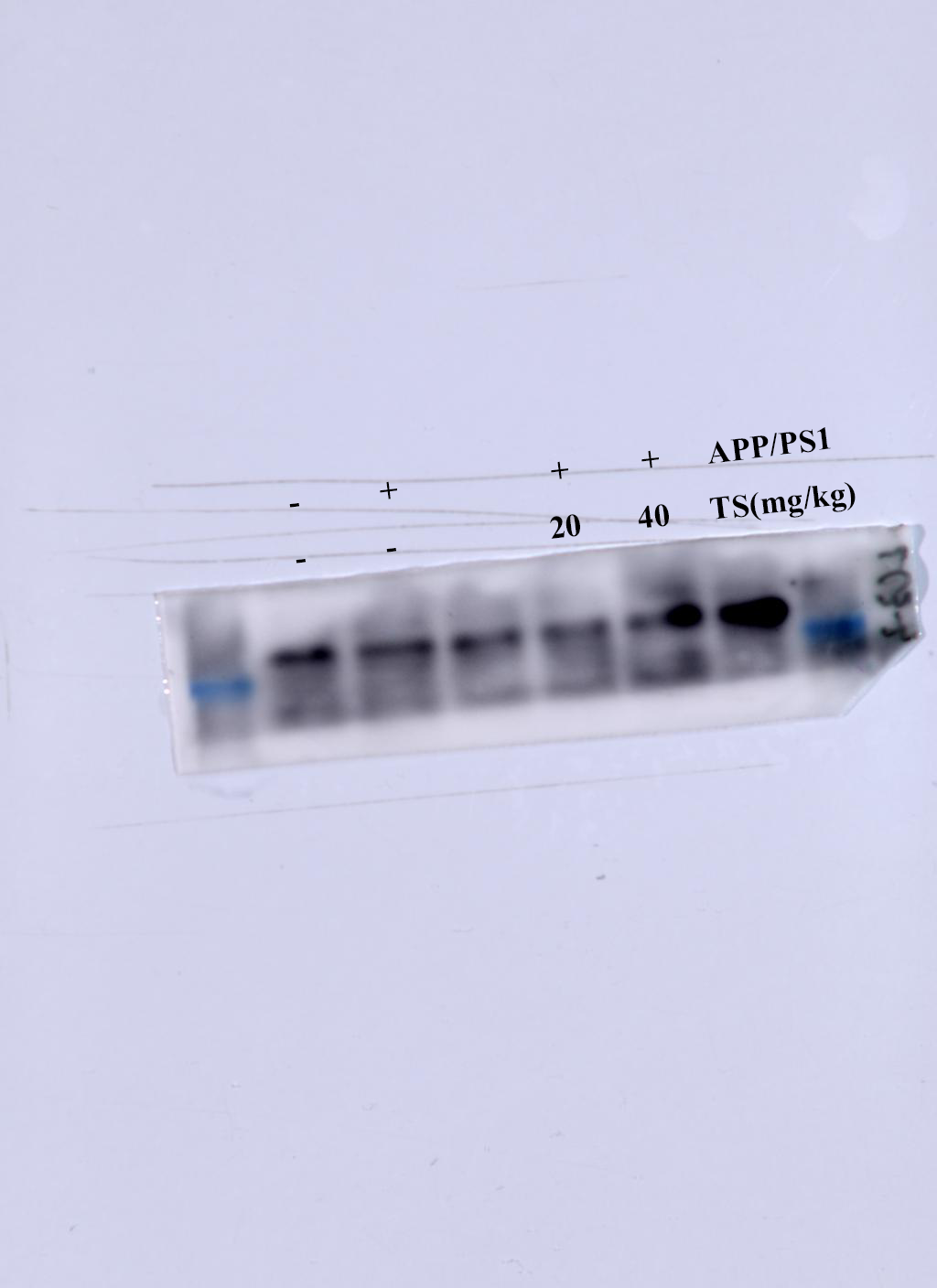

Supplement: Supplementary file 4 [file DataSheet2.ZIP › Fig.5D LC3/Fig.5H orginal images for quantitative analysis-2.tif]

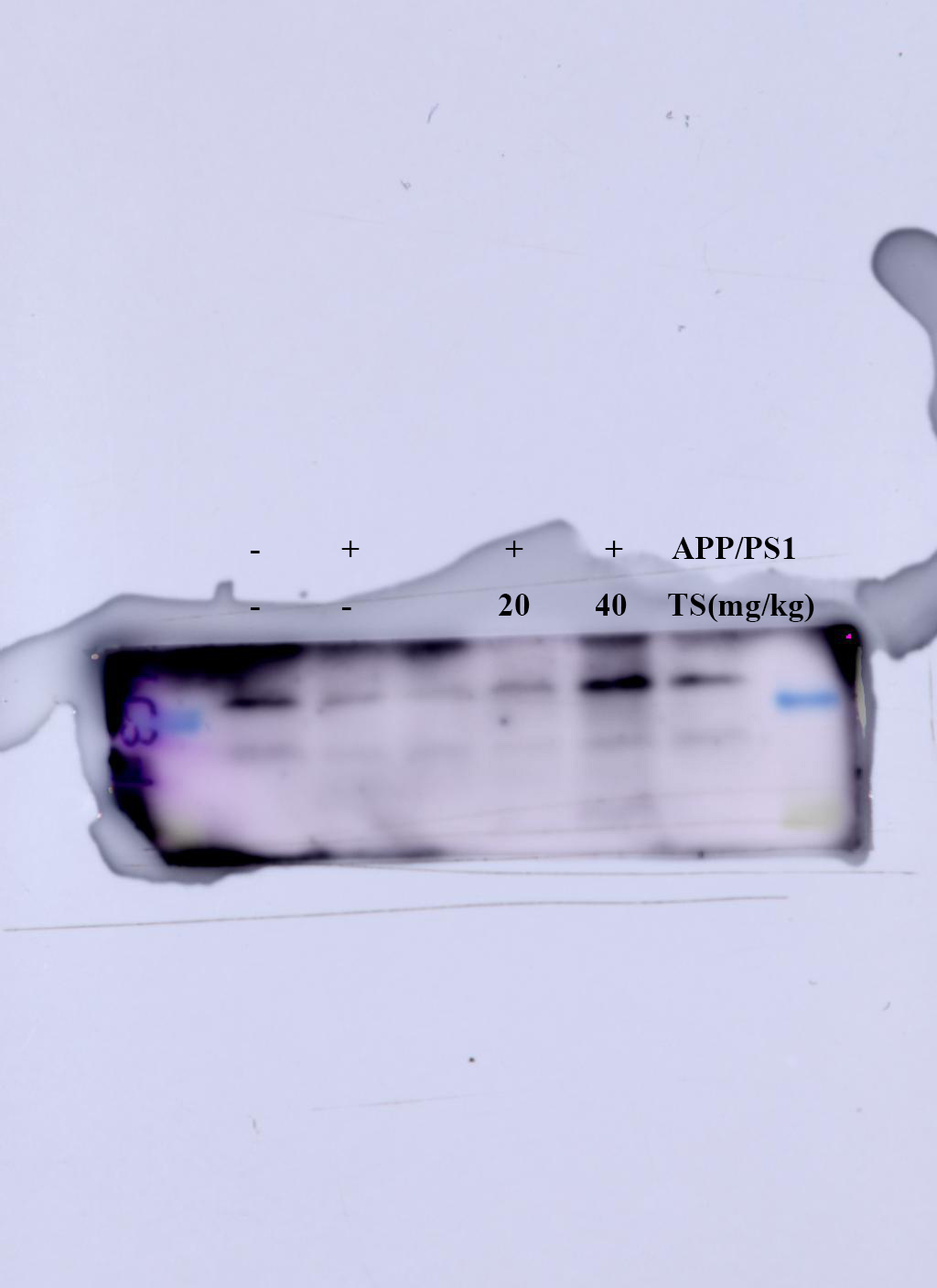

Supplement: Supplementary file 4 [file DataSheet2.ZIP › Fig.5D LC3/LC3-1-2 2021.09.21_19.36.34_Ch+Marker.tif]

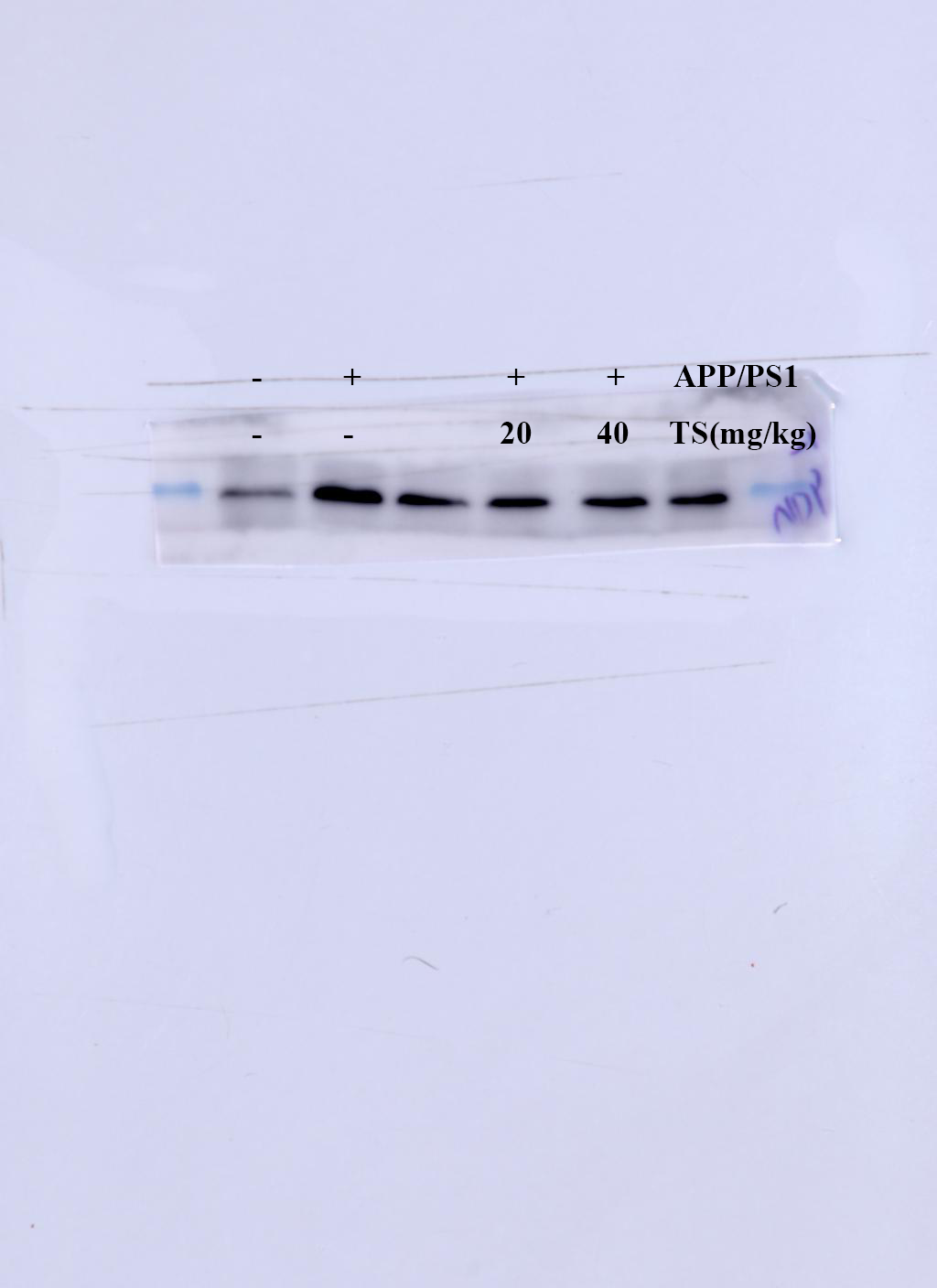

Supplement: Supplementary file 4 [file DataSheet2.ZIP › Fig.5D NDP52/Fig.5D orginal images for quantitative analysis-1.tif]

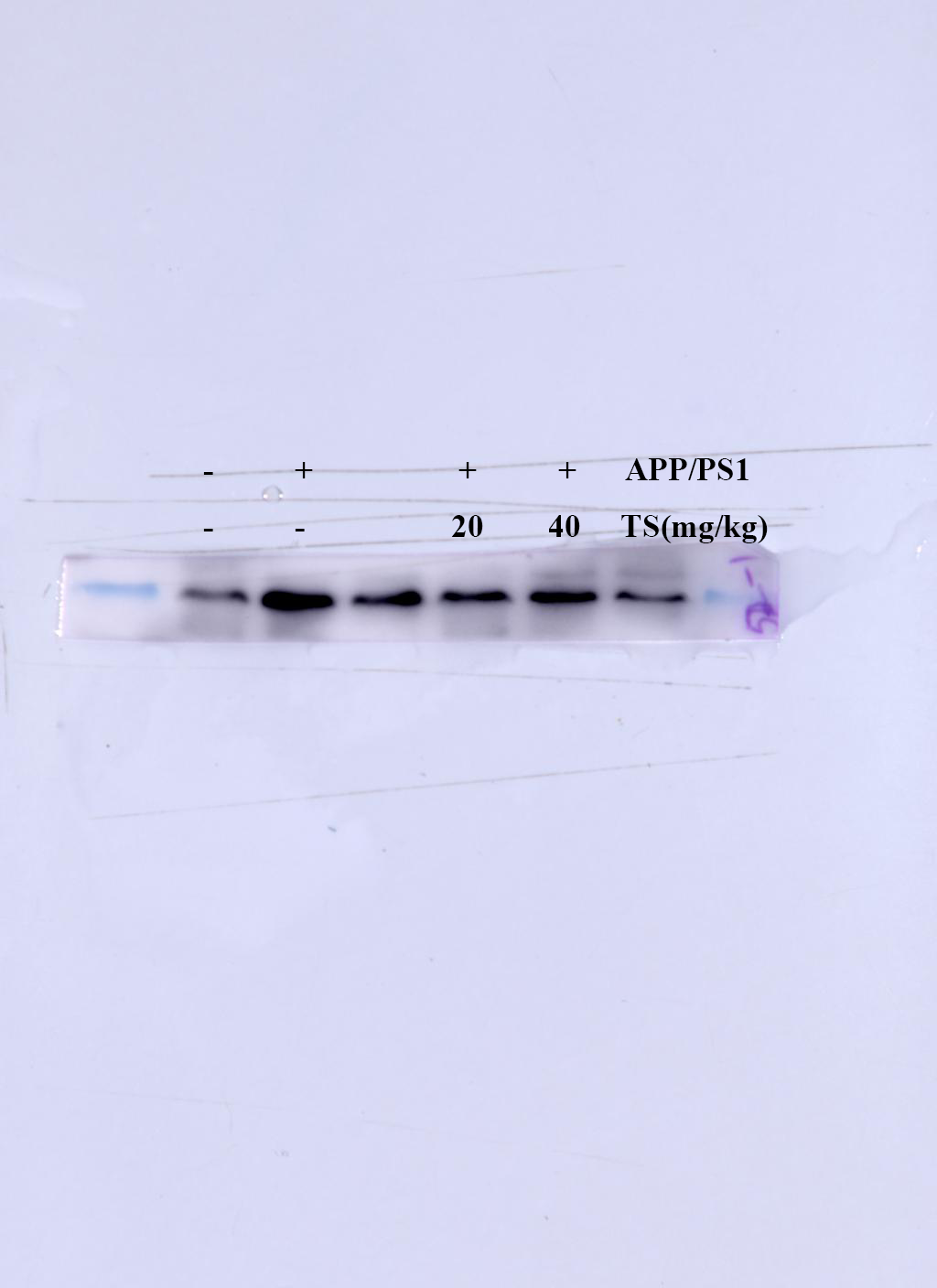

Supplement: Supplementary file 4 [file DataSheet2.ZIP › Fig.5D NDP52/Fig.5D orginal images for quantitative analysis-2.tif]

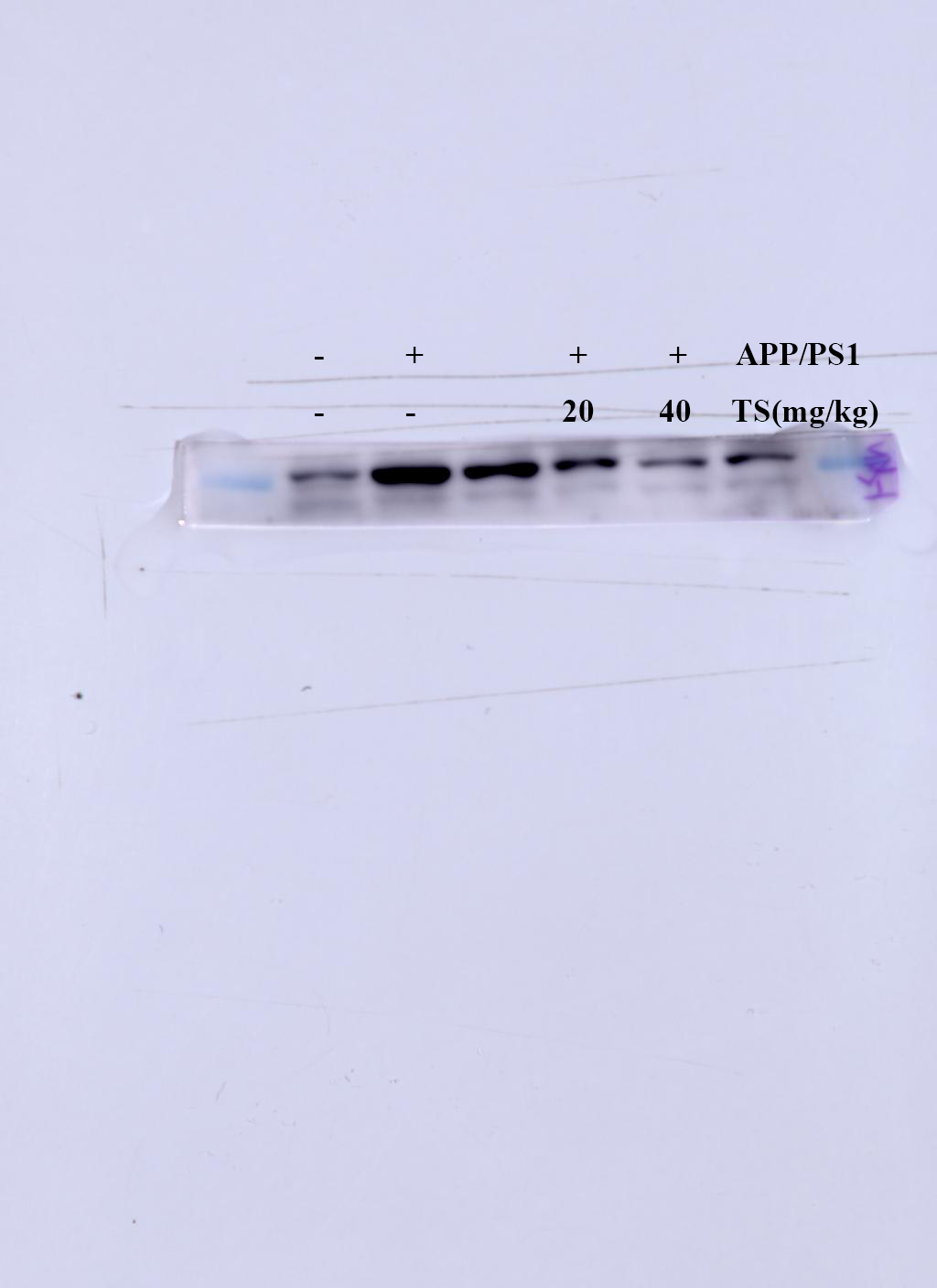

Supplement: Supplementary file 4 [file DataSheet2.ZIP › Fig.5D NDP52/Fig.5D orginal images.tif]

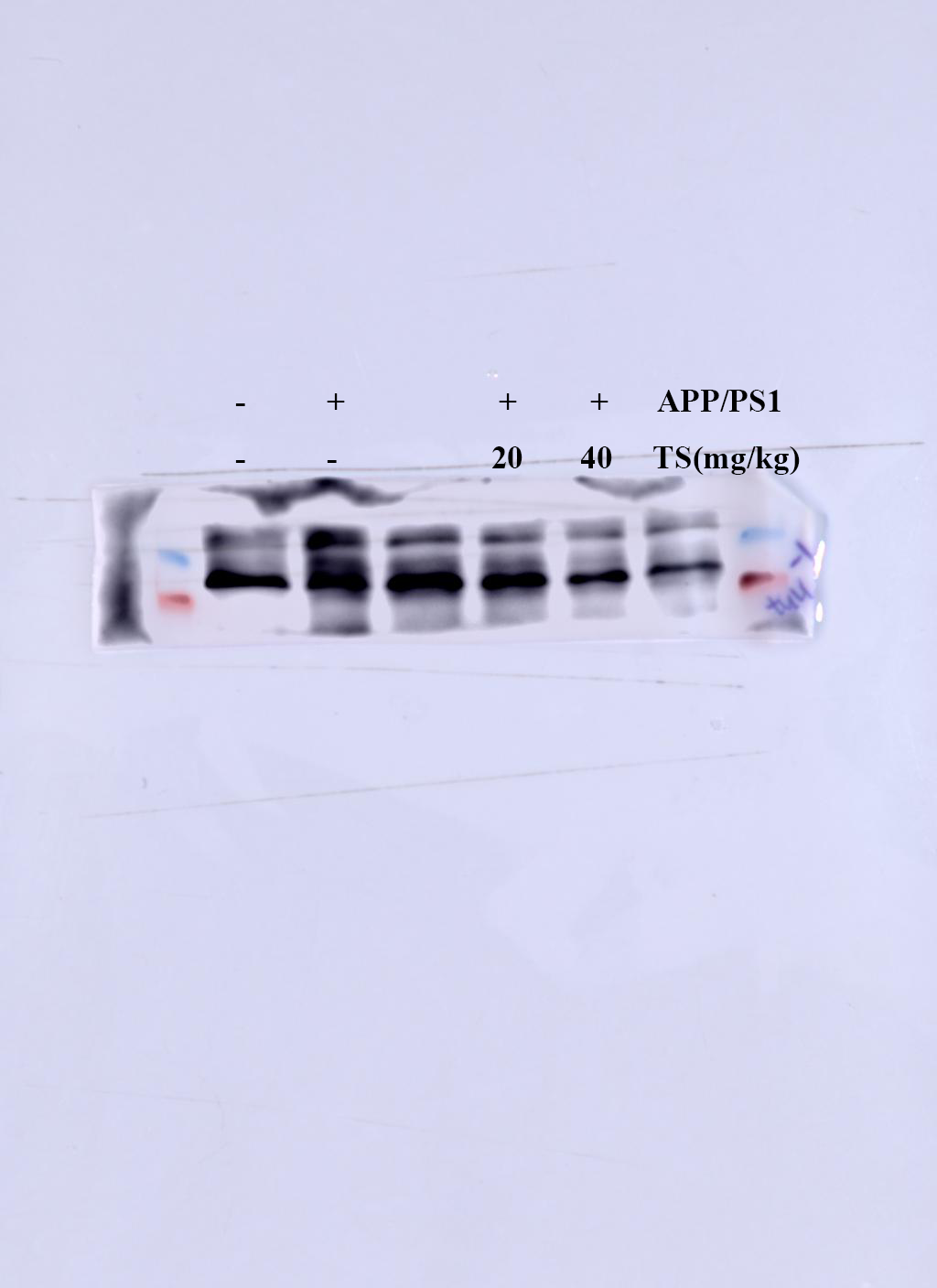

Supplement: Supplementary file 4 [file DataSheet2.ZIP › Fig.5D p-tau/Fig.6A orginal images for quantitative anaylsis-1.tif]

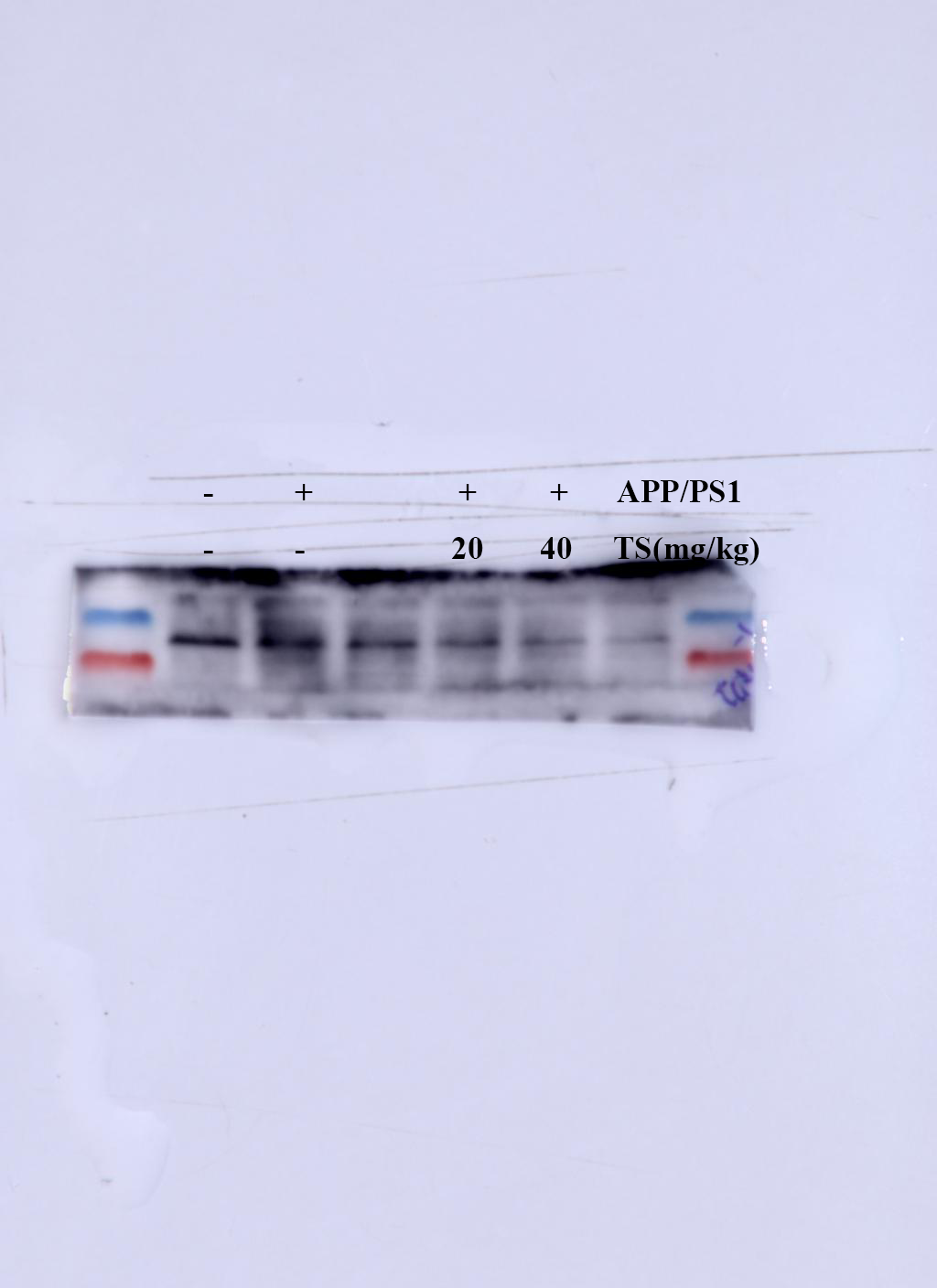

Supplement: Supplementary file 4 [file DataSheet2.ZIP › Fig.5D p-tau/Fig.6A orginal images for quantitative anaylsis-2.tif]

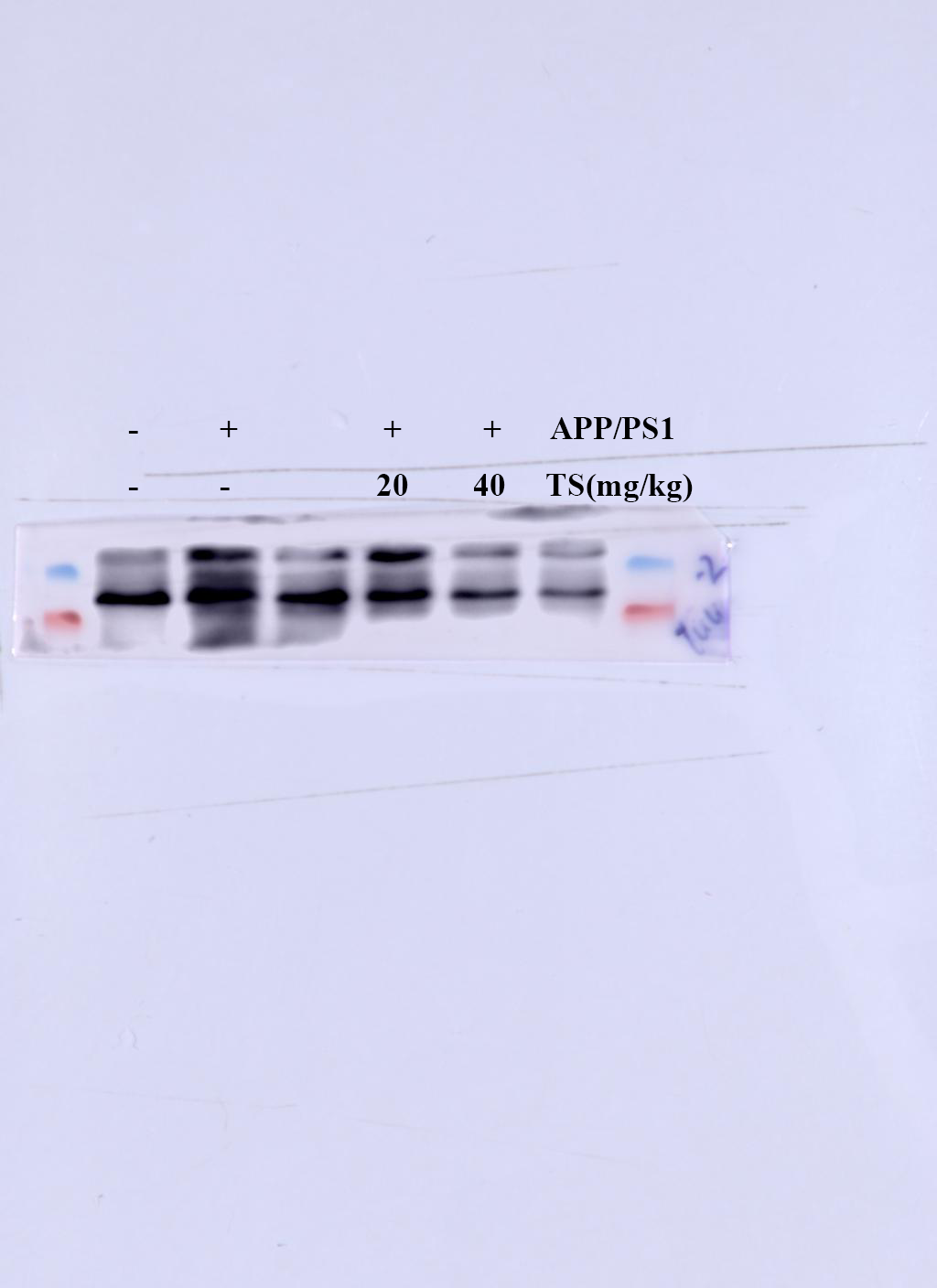

Supplement: Supplementary file 4 [file DataSheet2.ZIP › Fig.5D p-tau/Fig.6A orginal images.tif]

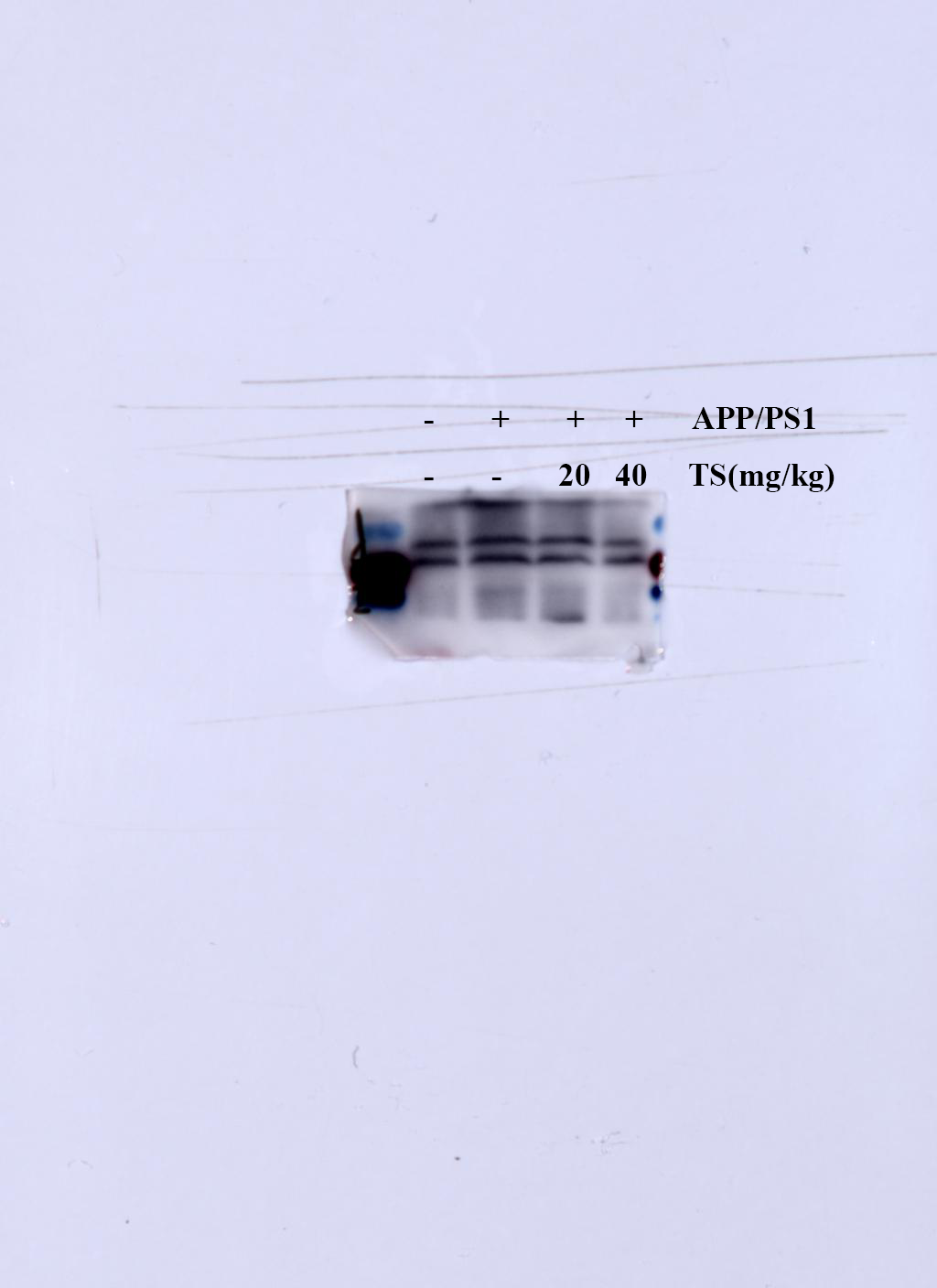

Supplement: Supplementary file 4 [file DataSheet2.ZIP › Fig.5D p62/Fig.5D orginal images for quantitative analysis-1.tif]

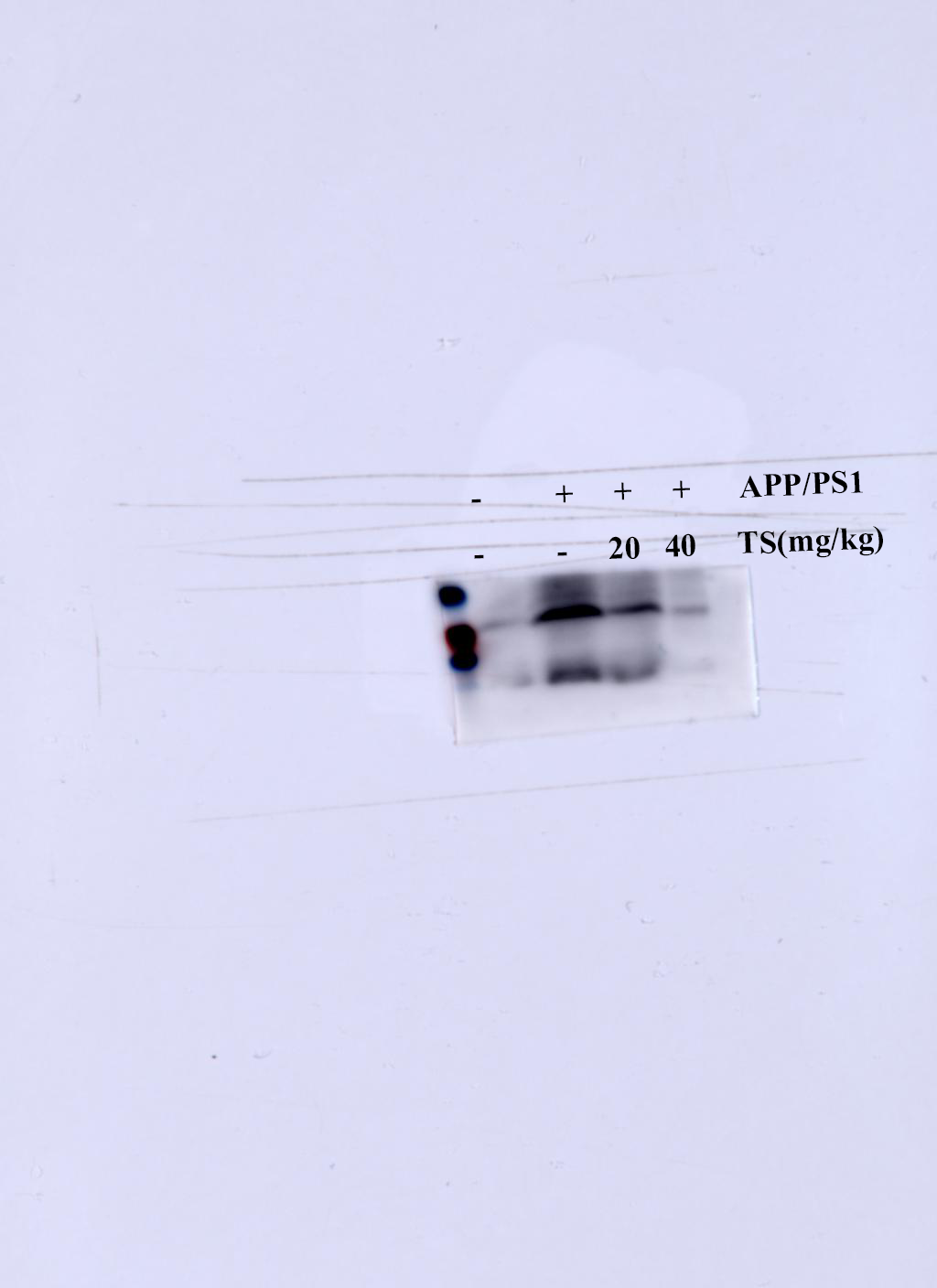

Supplement: Supplementary file 4 [file DataSheet2.ZIP › Fig.5D p62/Fig.5D orginal images for quantitative analysis-2.tif]

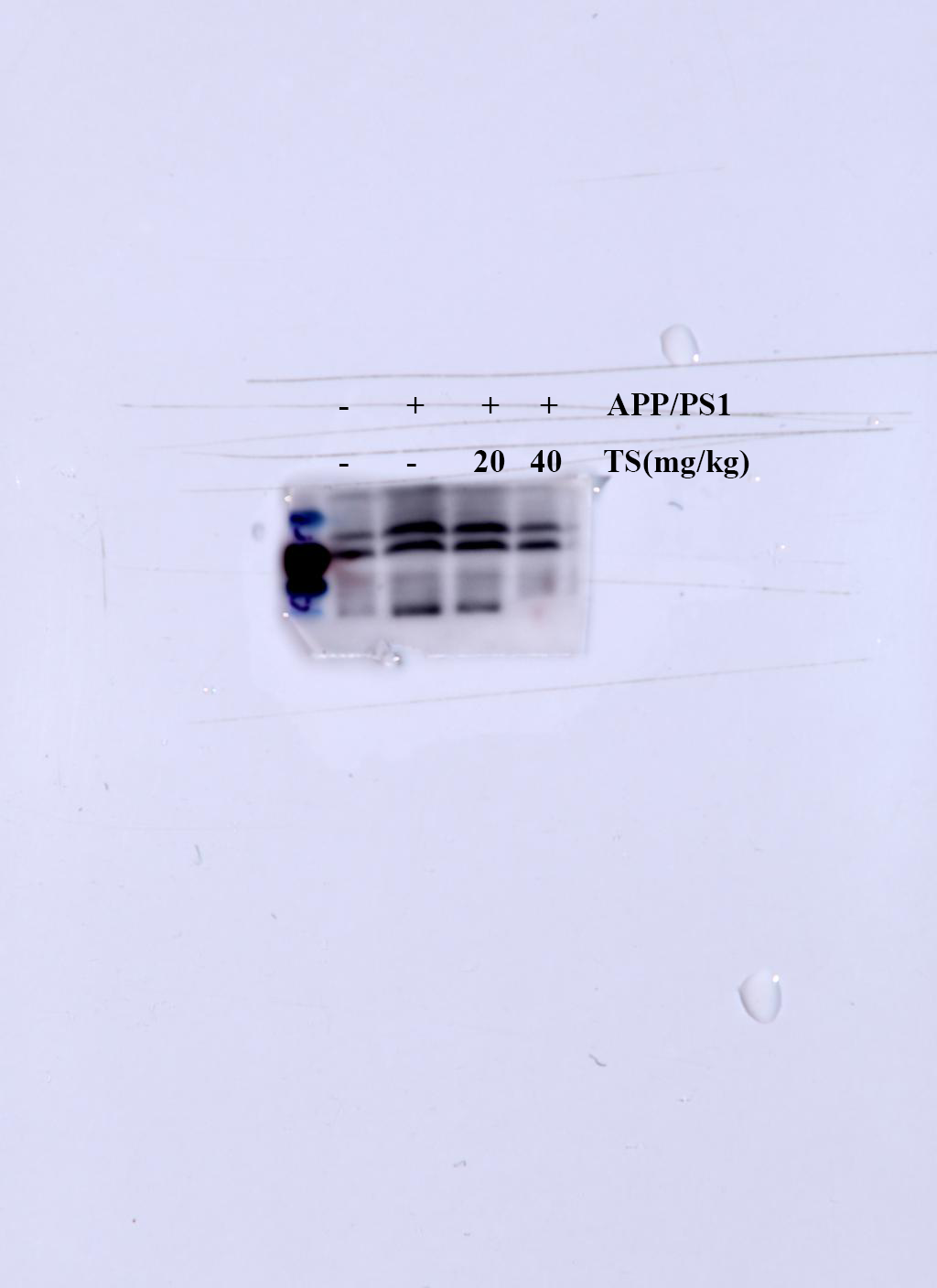

Supplement: Supplementary file 4 [file DataSheet2.ZIP › Fig.5D p62/Fig.5D orginal images.tif]

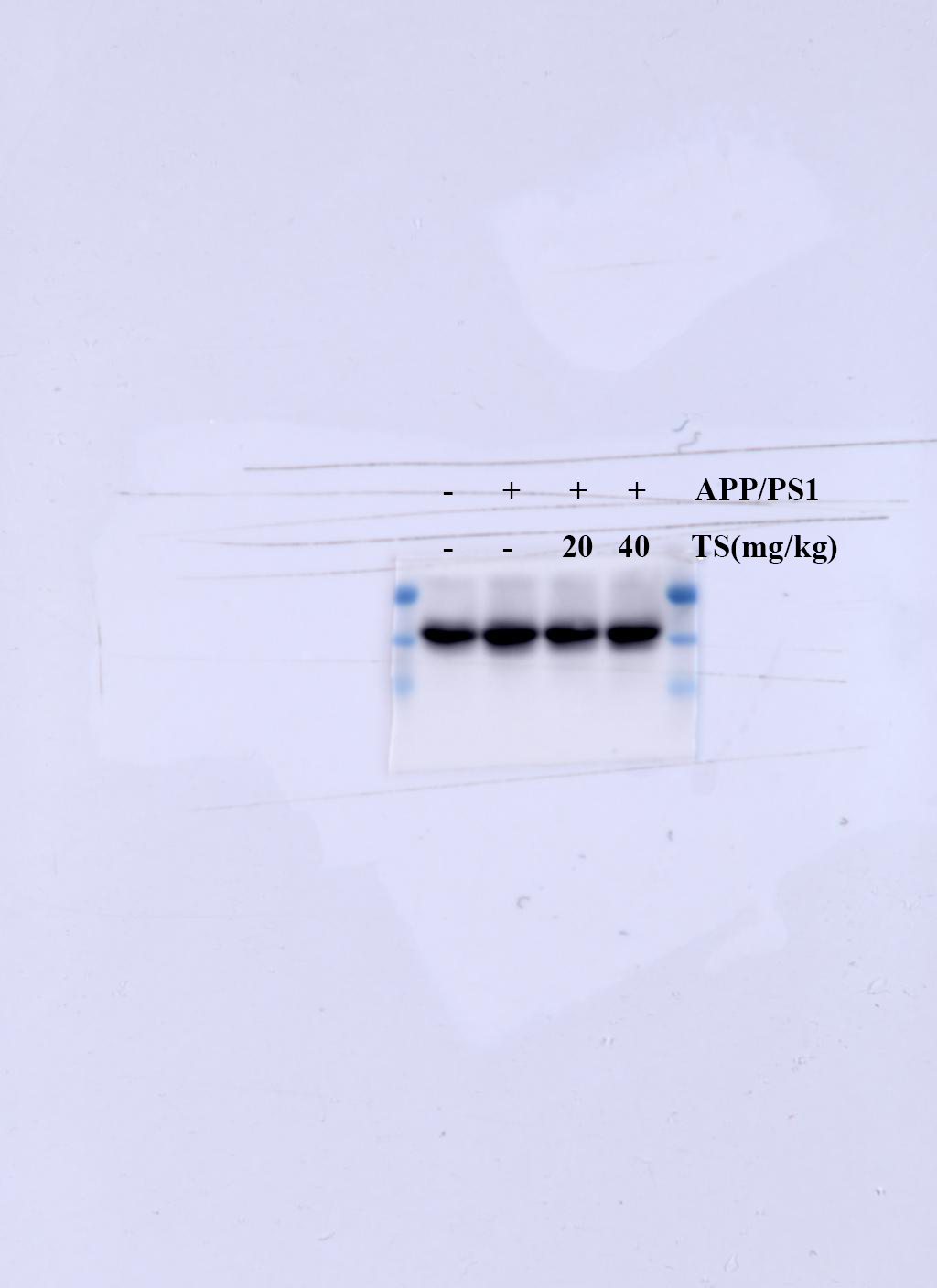

Supplement: Supplementary file 4 [file DataSheet2.ZIP › Fig.5D p62/─┌▓╬/Fig.5D orginal images for quantitative analysis-1.tif]

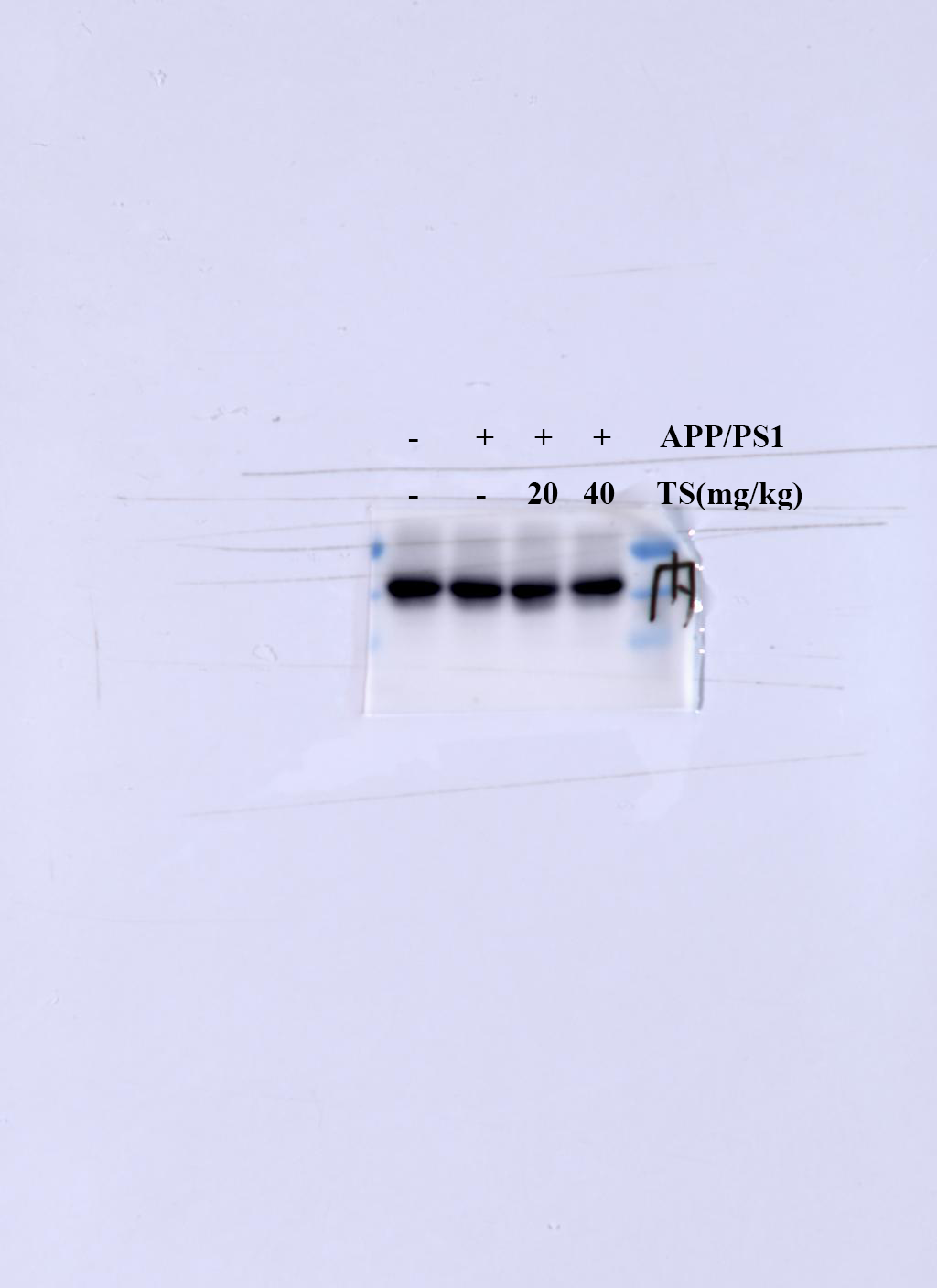

Supplement: Supplementary file 4 [file DataSheet2.ZIP › Fig.5D p62/─┌▓╬/Fig.5D orginal images for quantitative analysis-2.tif]

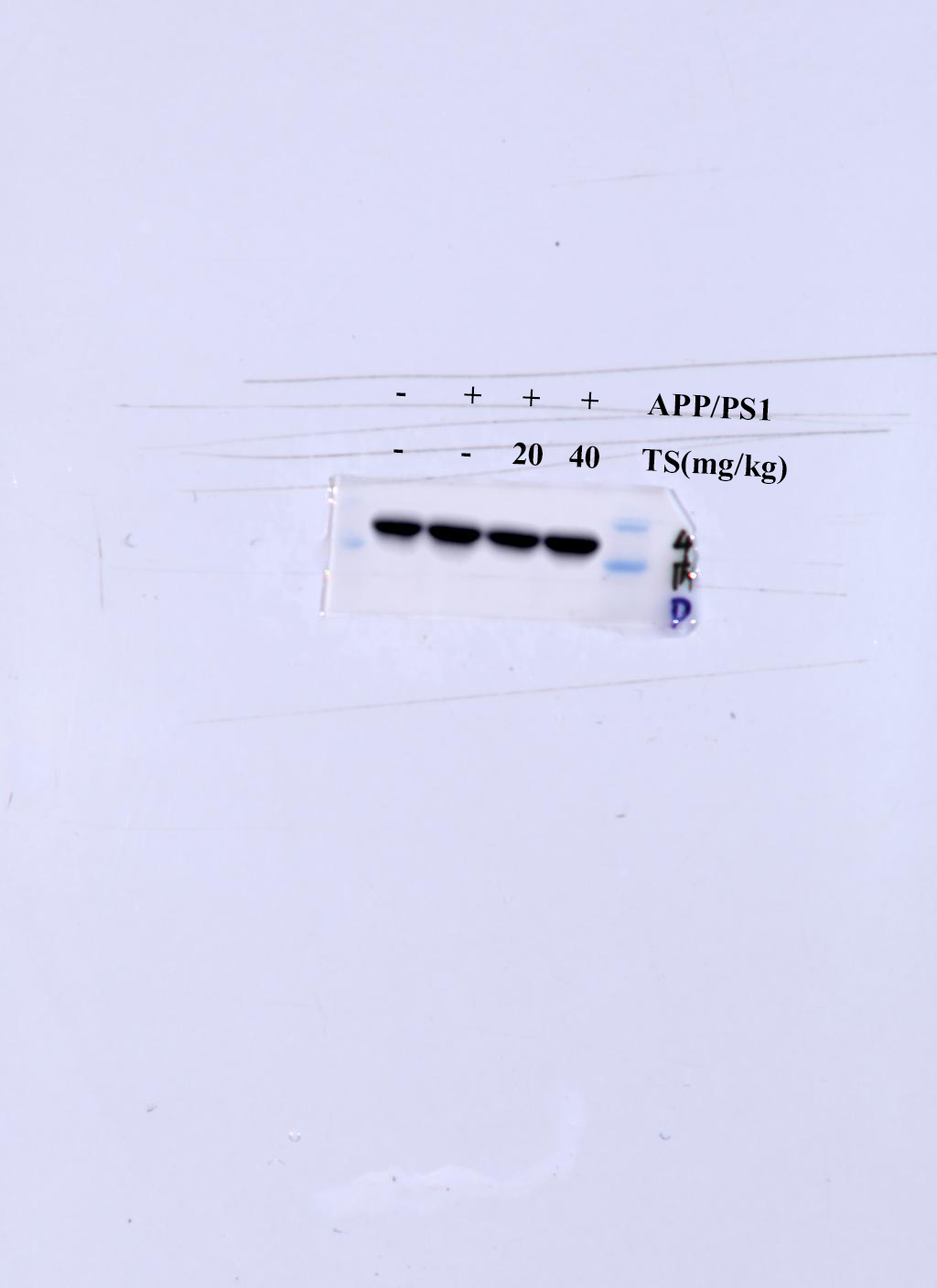

Supplement: Supplementary file 4 [file DataSheet2.ZIP › Fig.5D p62/─┌▓╬/Fig.5D orginal images.tif]

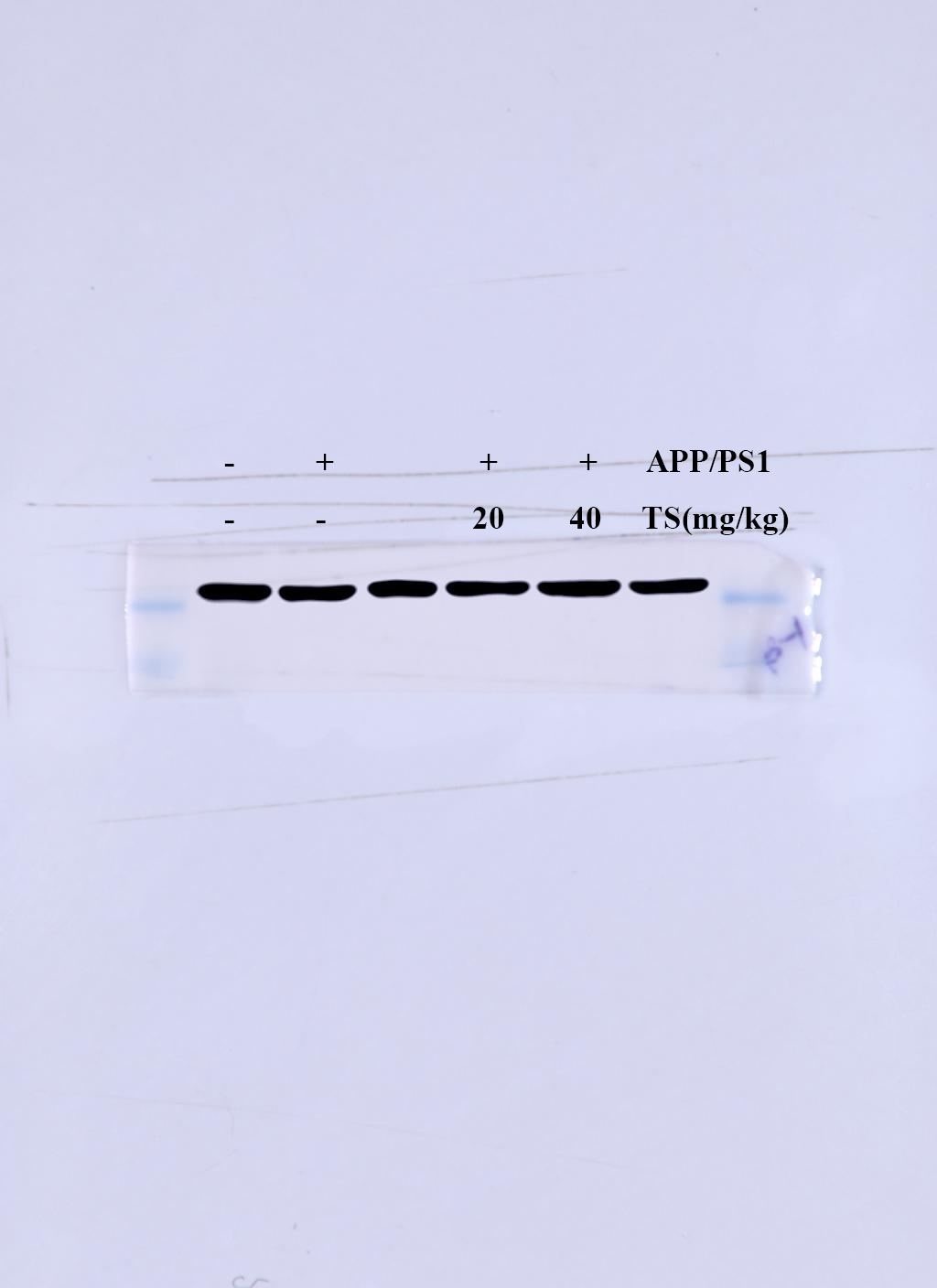

Supplement: Supplementary file 4 [file DataSheet2.ZIP › Fig.5D a┬-actin/Fig.5D orginal images for quantitative analysis-1.tif]

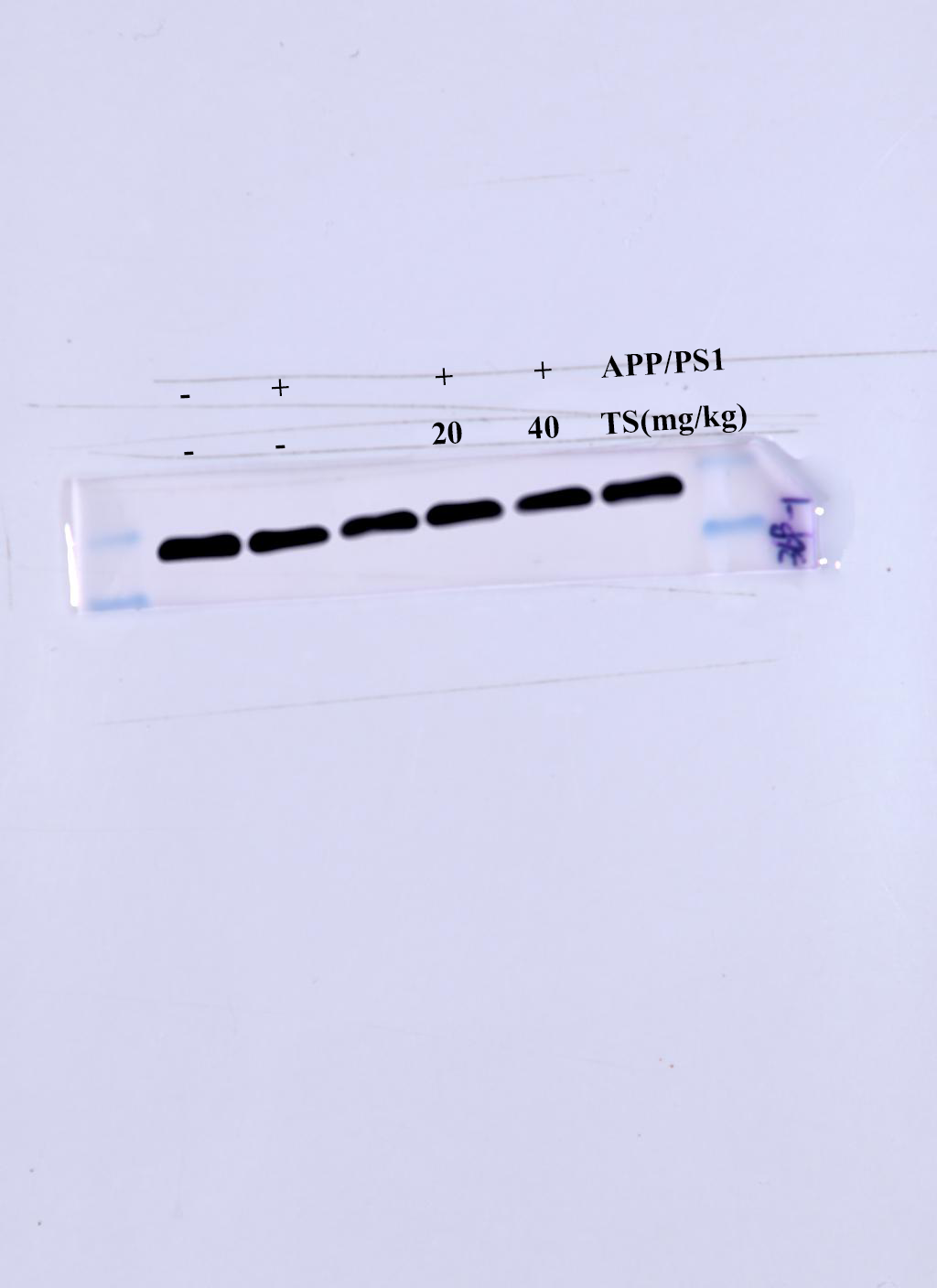

Supplement: Supplementary file 4 [file DataSheet2.ZIP › Fig.5D a┬-actin/Fig.5D orginal images for quantitative analysis-2.tif]

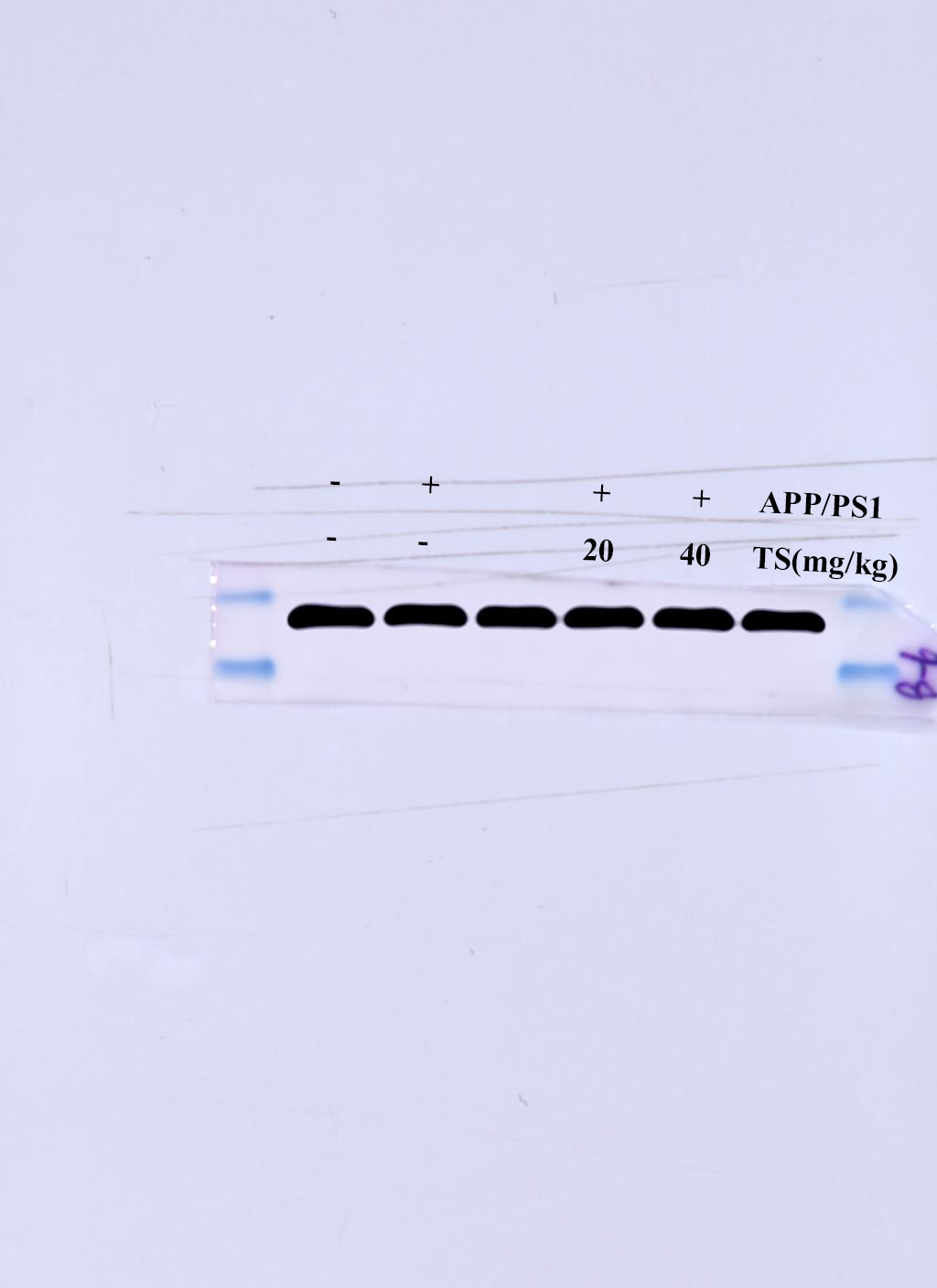

Supplement: Supplementary file 4 [file DataSheet2.ZIP › Fig.5D a┬-actin/Fig.5D orginal images.tif]

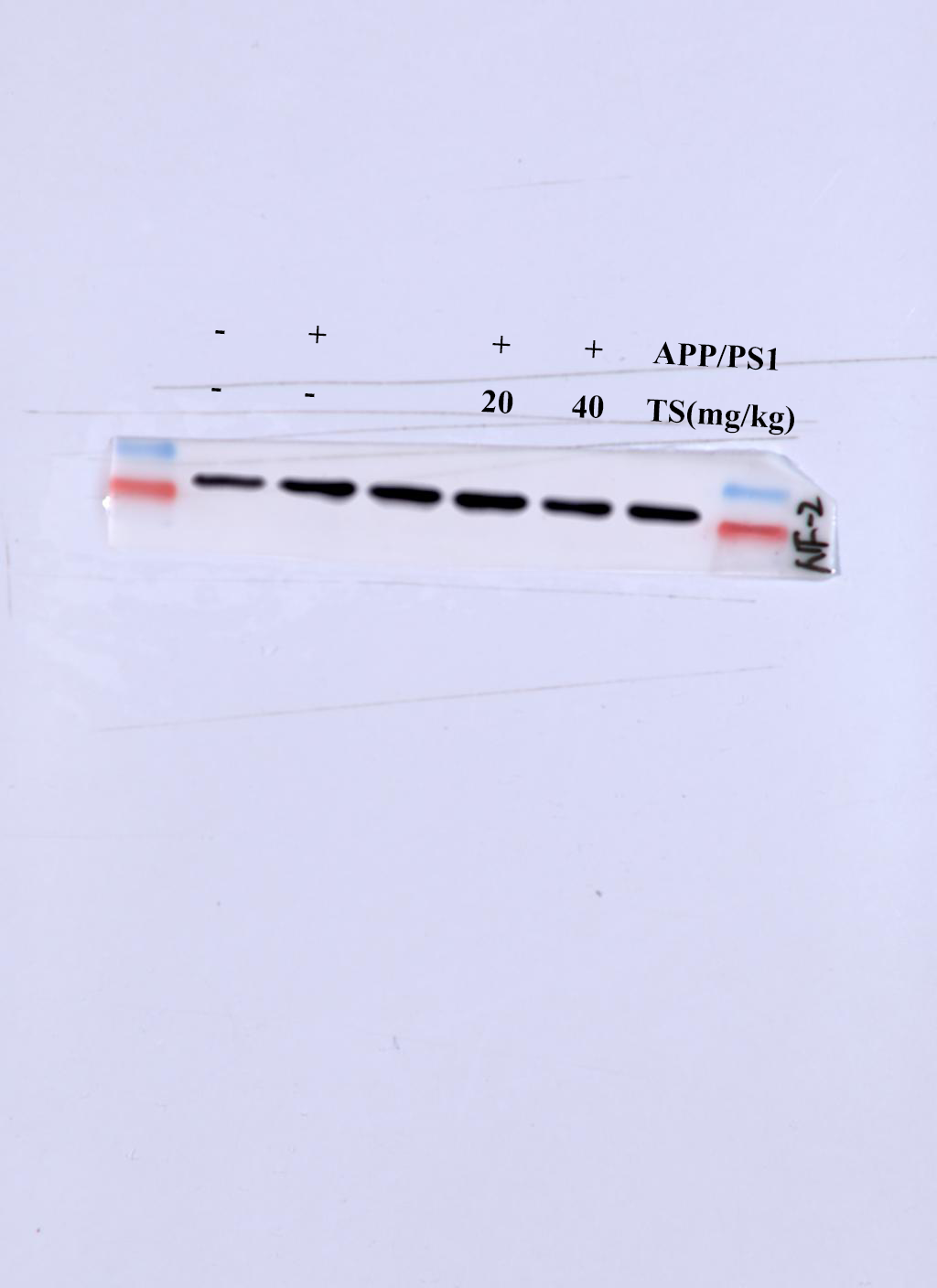

Supplement: Supplementary file 5 [file DataSheet5.ZIP › Fig.9D NF-a╩B/Fig.9D orginal images for analysis-1.tif]

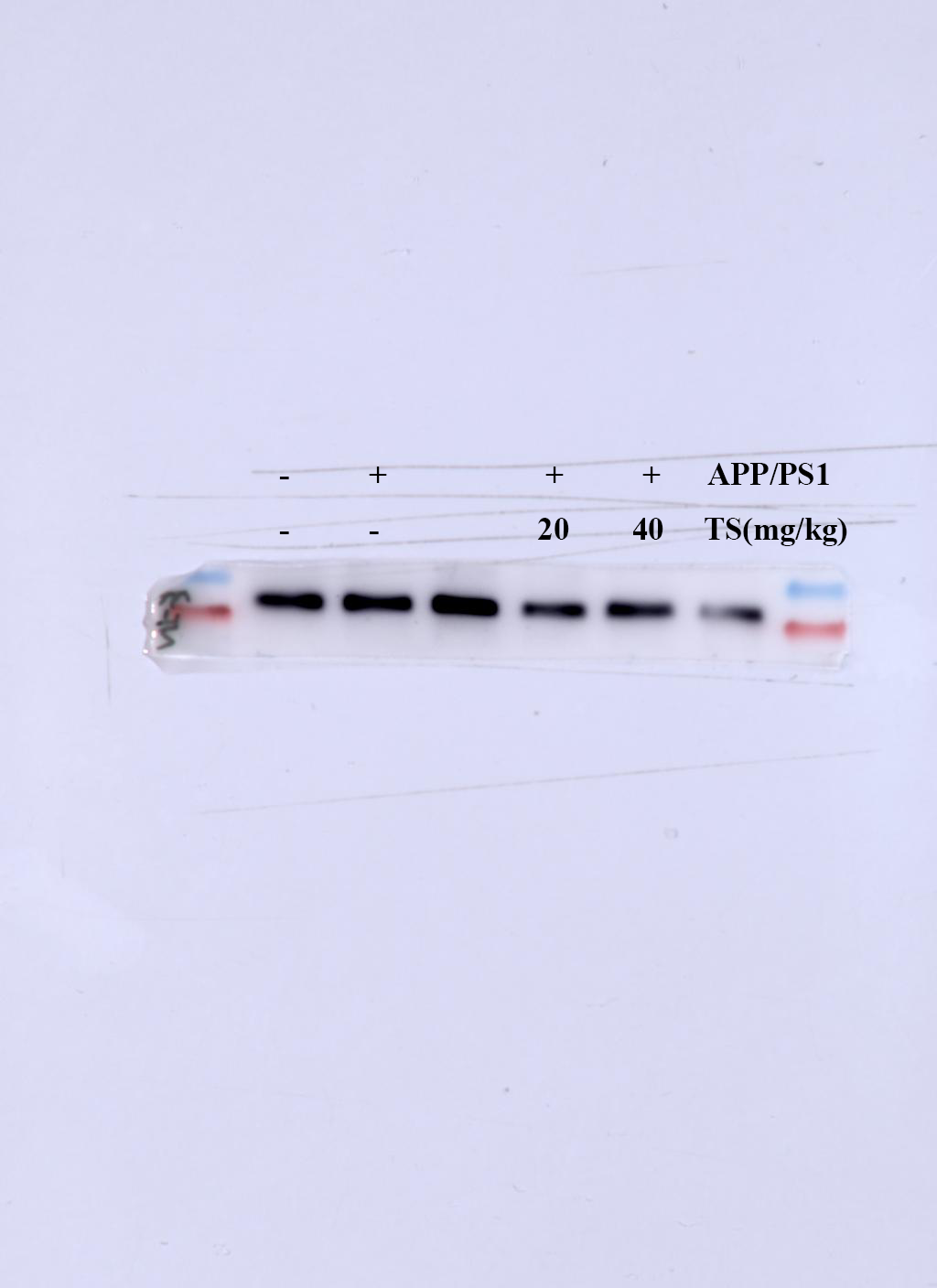

Supplement: Supplementary file 5 [file DataSheet5.ZIP › Fig.9D NF-a╩B/Fig.9D orginal images for analysis-2.tif]

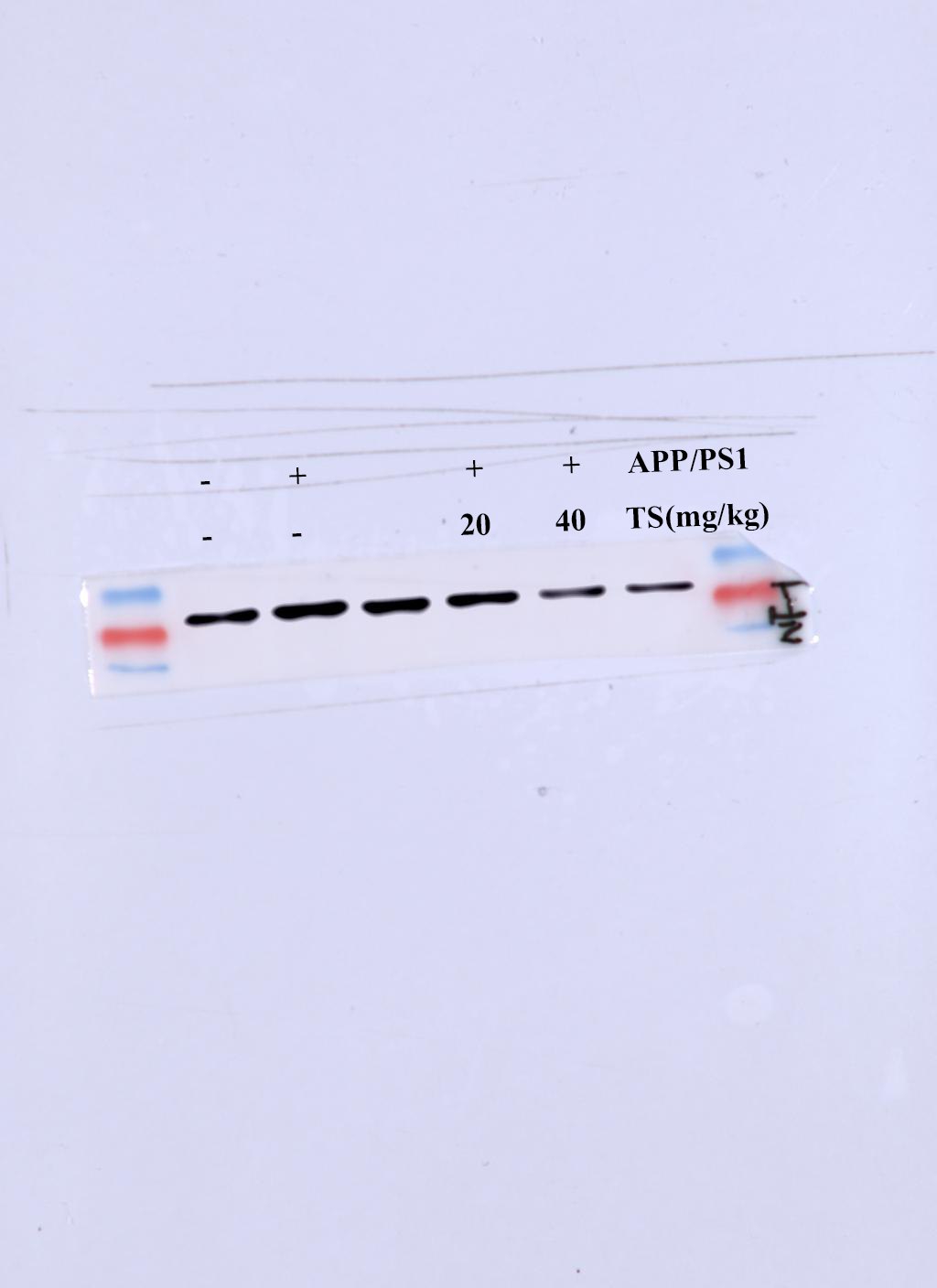

Supplement: Supplementary file 5 [file DataSheet5.ZIP › Fig.9D NF-a╩B/Fig.9D orginal images.tif]

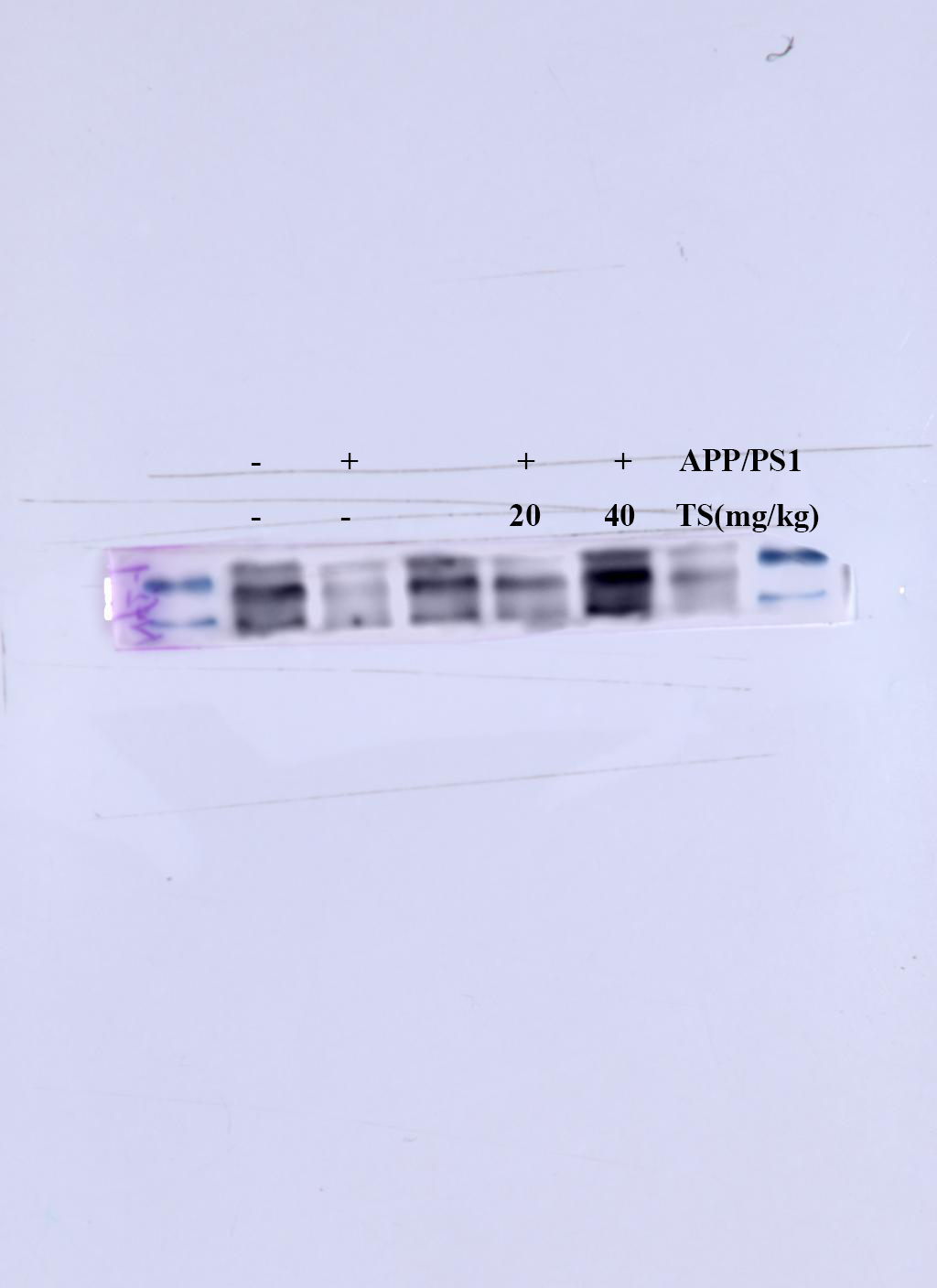

Supplement: Supplementary file 5 [file DataSheet5.ZIP › Fig.9D Nrf2/Fig.9D orgianl images for quantitative analysis-2.tif]

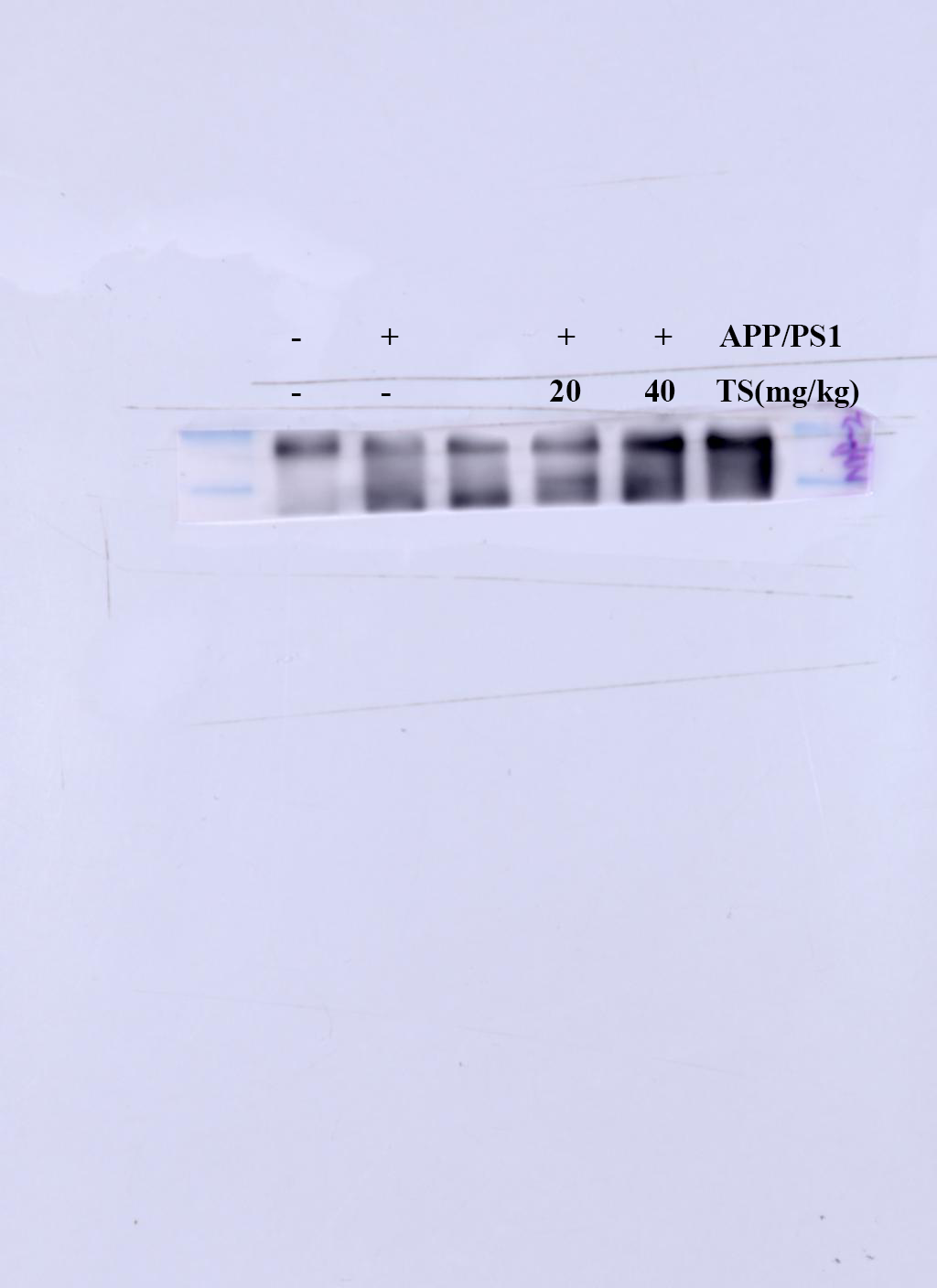

Supplement: Supplementary file 5 [file DataSheet5.ZIP › Fig.9D Nrf2/Fig.9D orgianl images.tif]

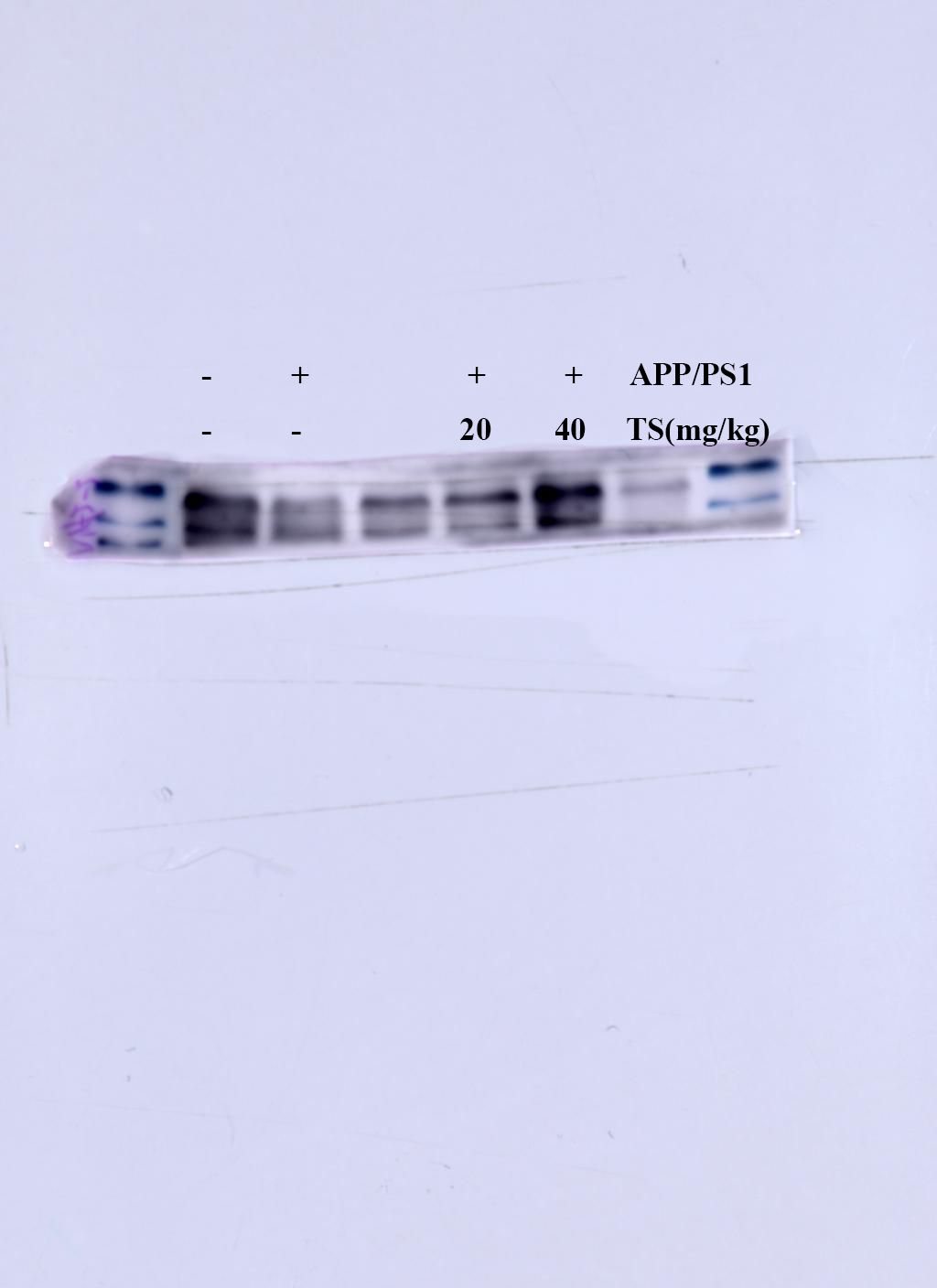

Supplement: Supplementary file 5 [file DataSheet5.ZIP › Fig.9D Nrf2/Fig.9D orginal images for quantitative analysis-1.tif]

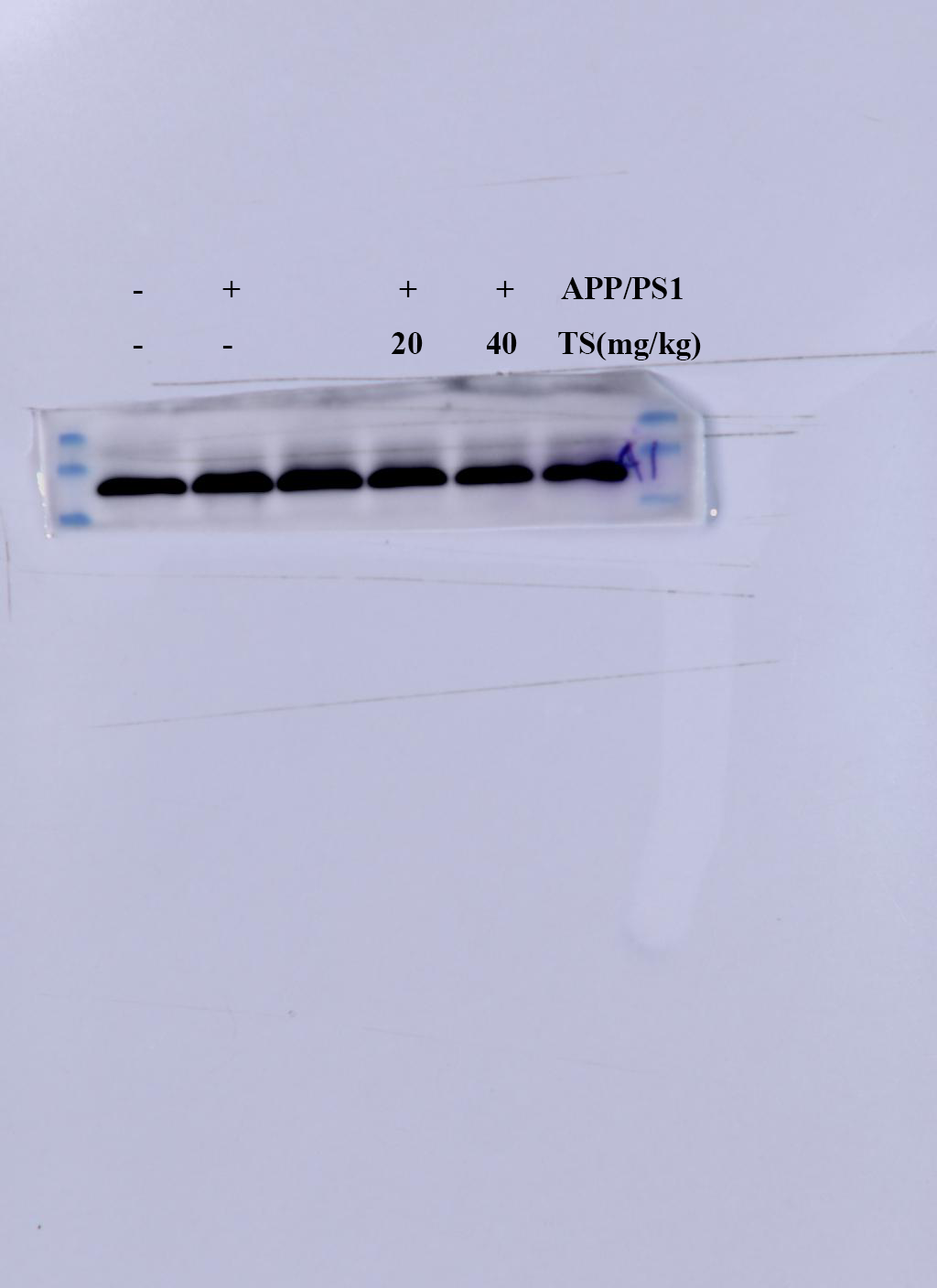

Supplement: Supplementary file 5 [file DataSheet5.ZIP › Fig.9D a┬-actin/Fig.9D orginal images for quantitative analysis-1.tif]

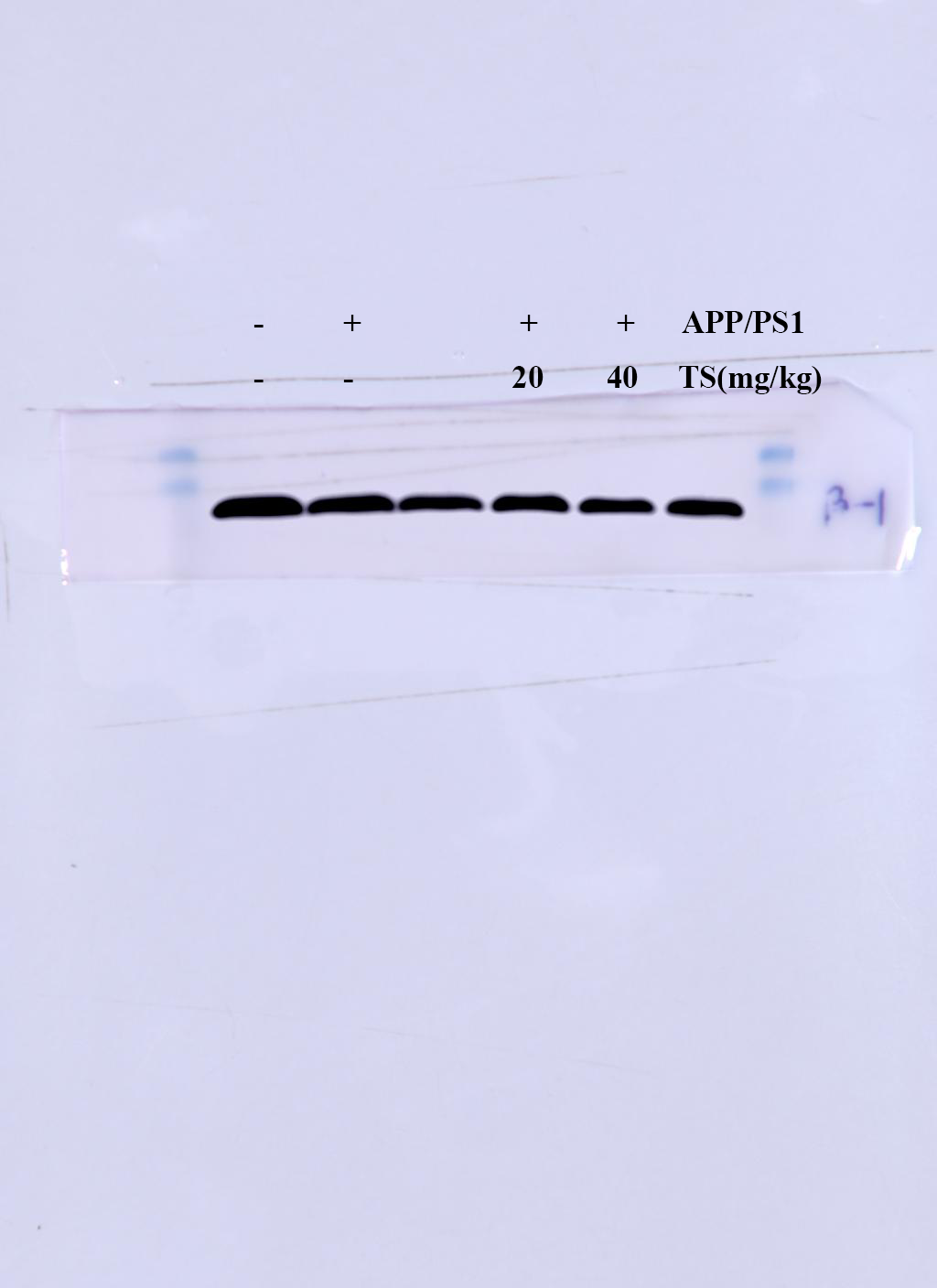

Supplement: Supplementary file 5 [file DataSheet5.ZIP › Fig.9D a┬-actin/Fig.9D orginal images for quantitative analysis-2.tif]

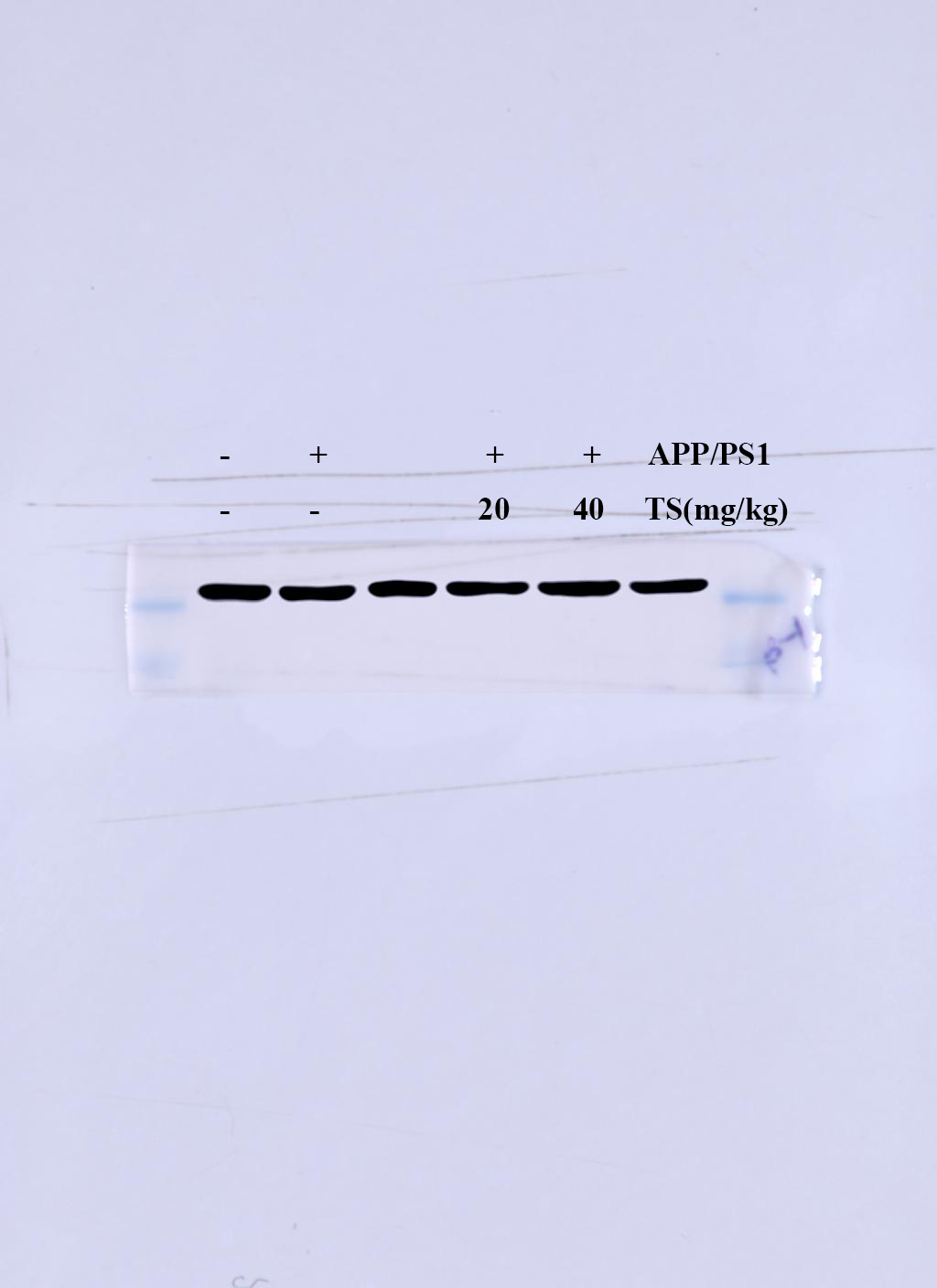

Supplement: Supplementary file 5 [file DataSheet5.ZIP › Fig.9D a┬-actin/Fig.9D orginal images.tif]

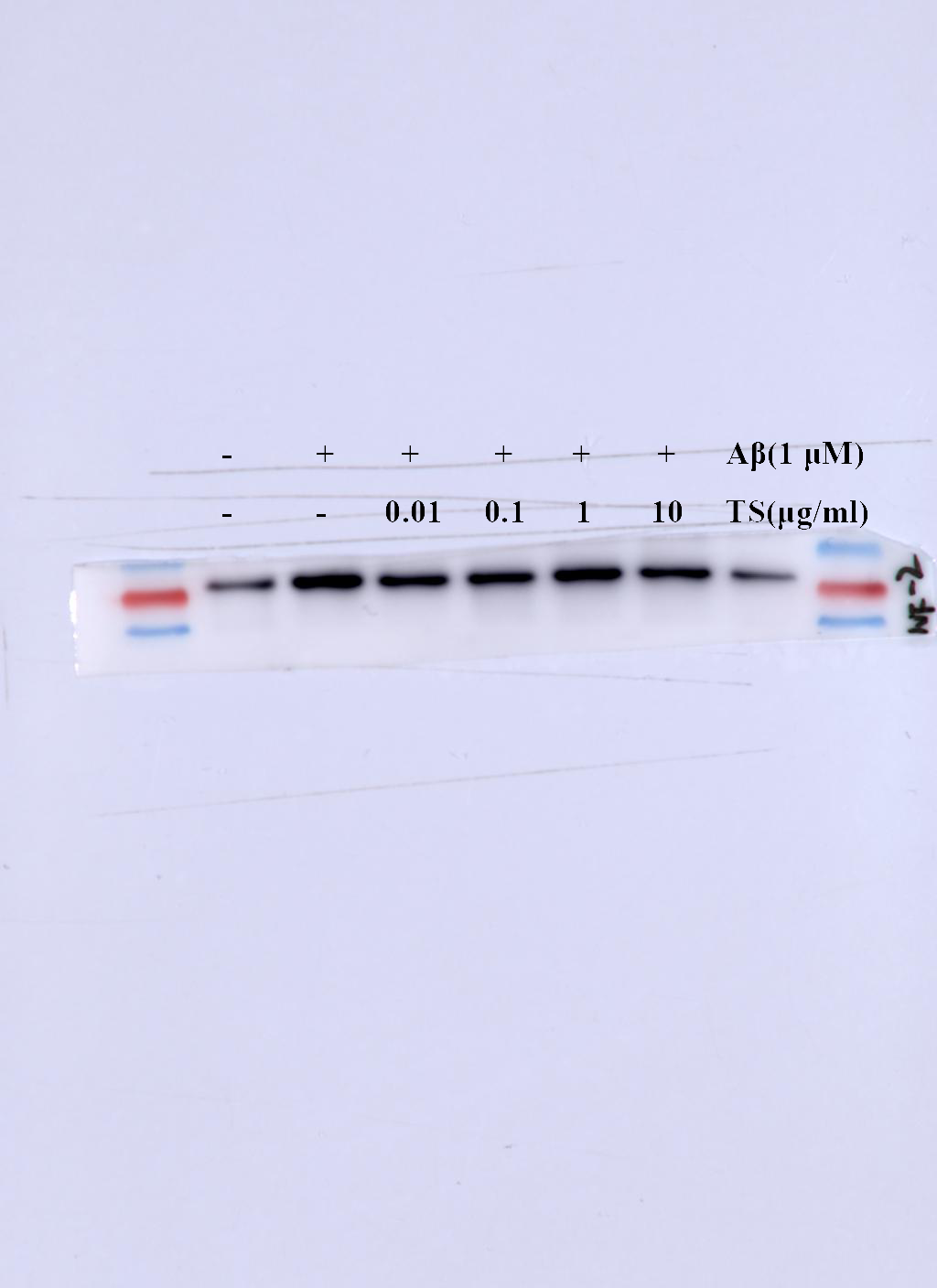

Supplement: Supplementary file 5 [file DataSheet5.ZIP › Fig.9F NF-KB/Fig.9F orginal image for quantitative anaylsis-1.tif]

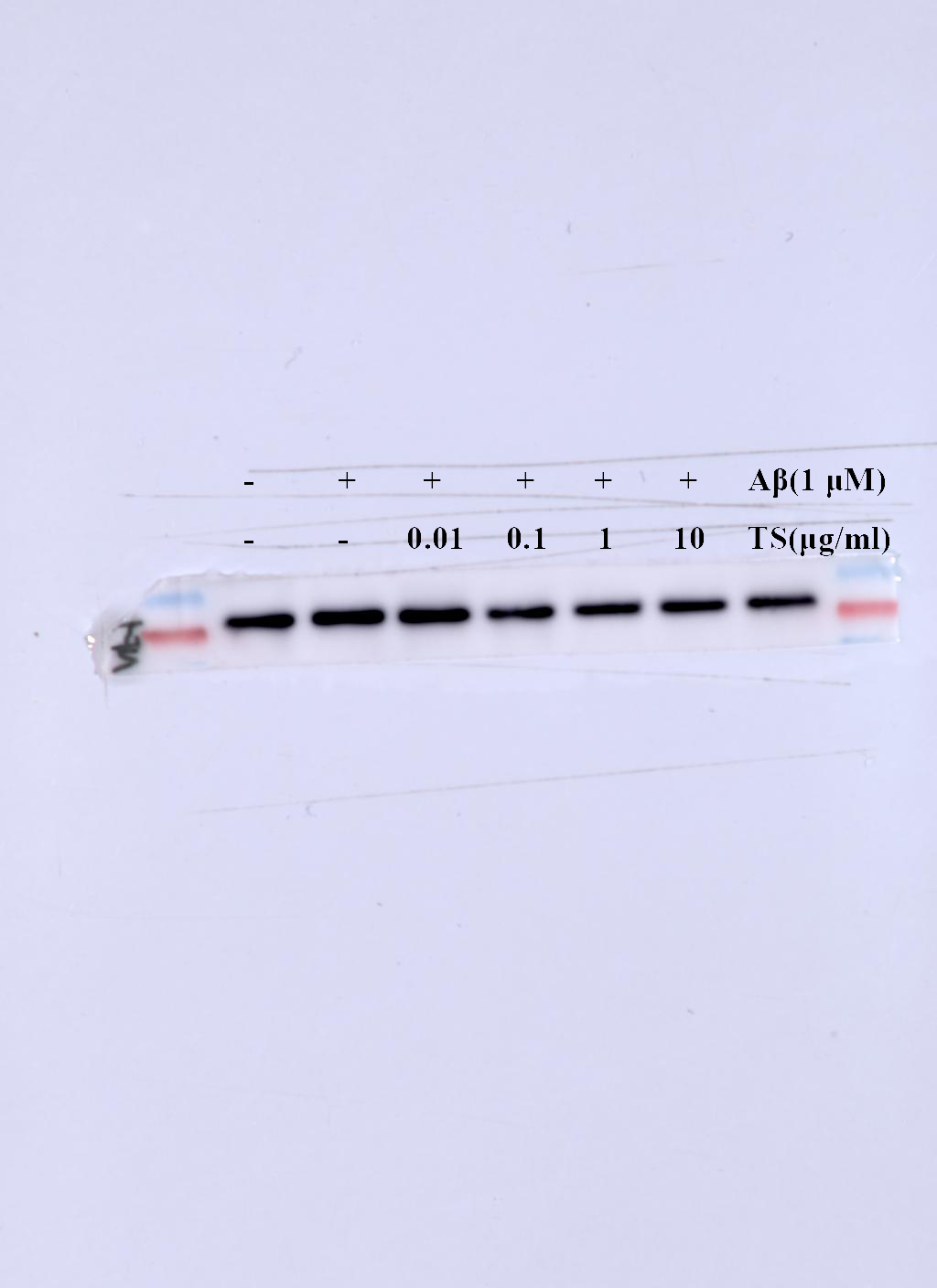

Supplement: Supplementary file 5 [file DataSheet5.ZIP › Fig.9F NF-KB/Fig.9F orginal image for quantitative anaylsis-2.tif]

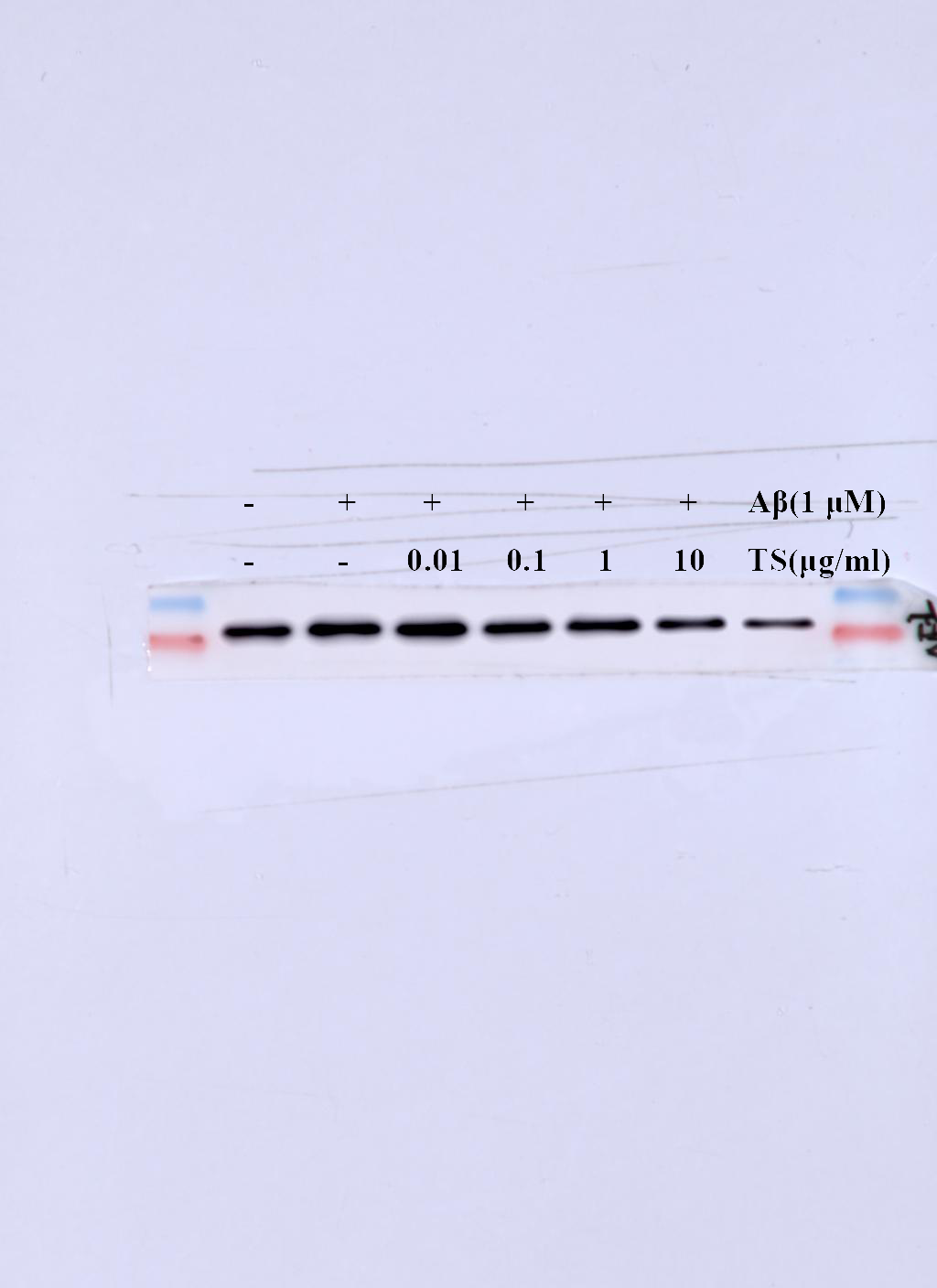

Supplement: Supplementary file 5 [file DataSheet5.ZIP › Fig.9F NF-KB/Fig.9F orginal image for quantitative anaylsis-3.tif]

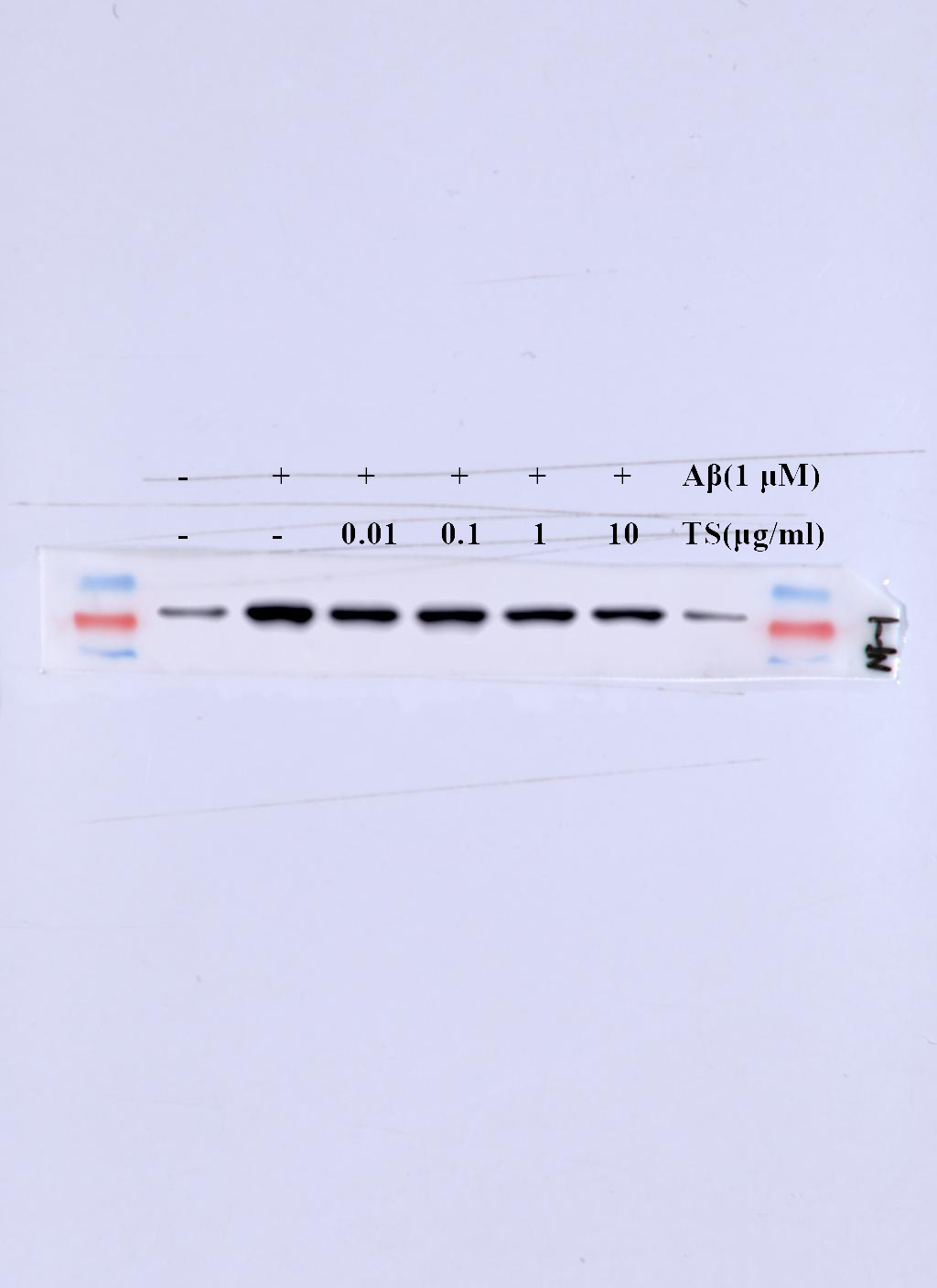

Supplement: Supplementary file 5 [file DataSheet5.ZIP › Fig.9F NF-KB/Fig.9F orginal images.tif]

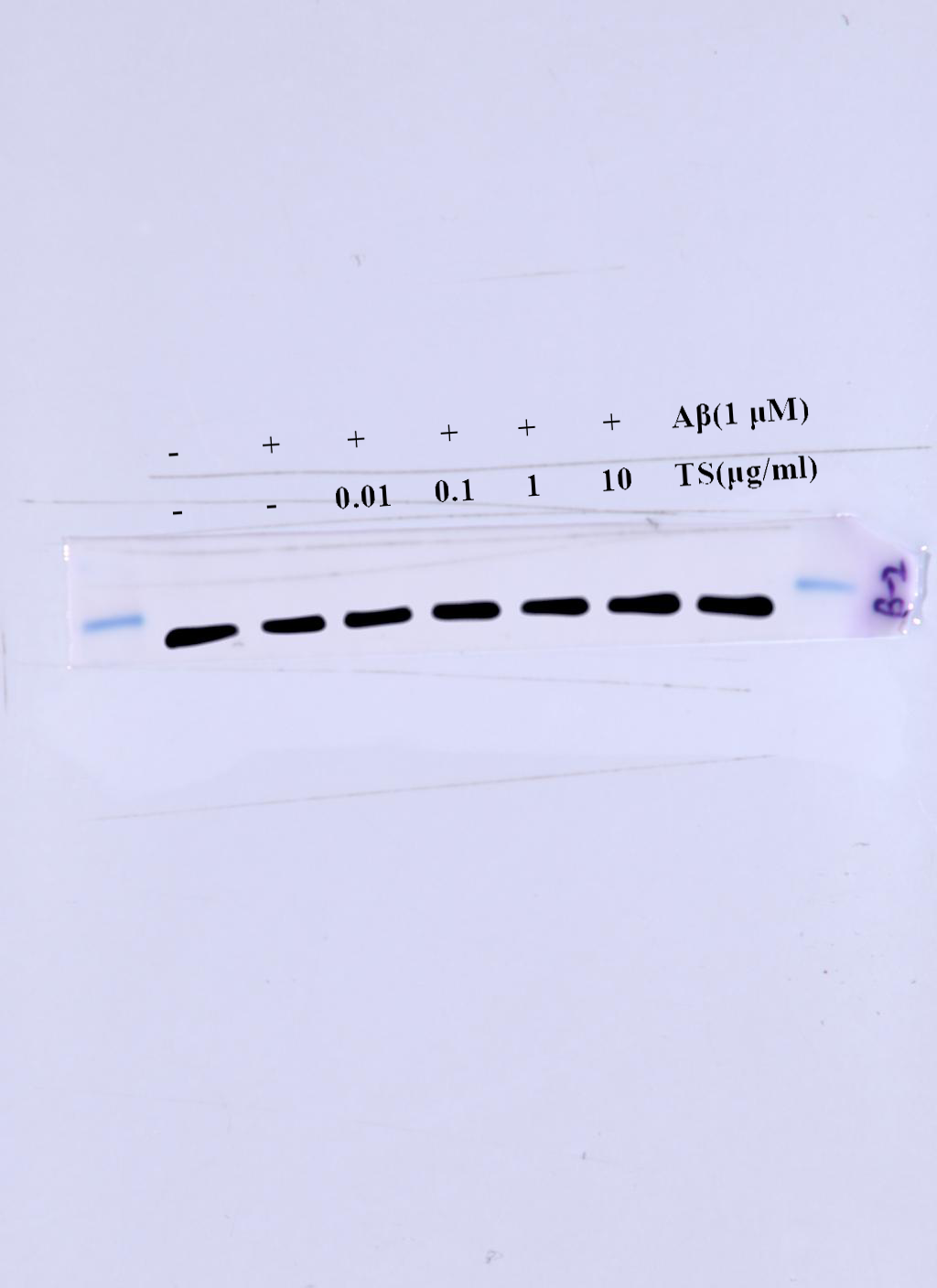

Supplement: Supplementary file 5 [file DataSheet5.ZIP › Fig.9F a┬-actin/Fig.9F orginal images for quantitative analysis-1.tif]

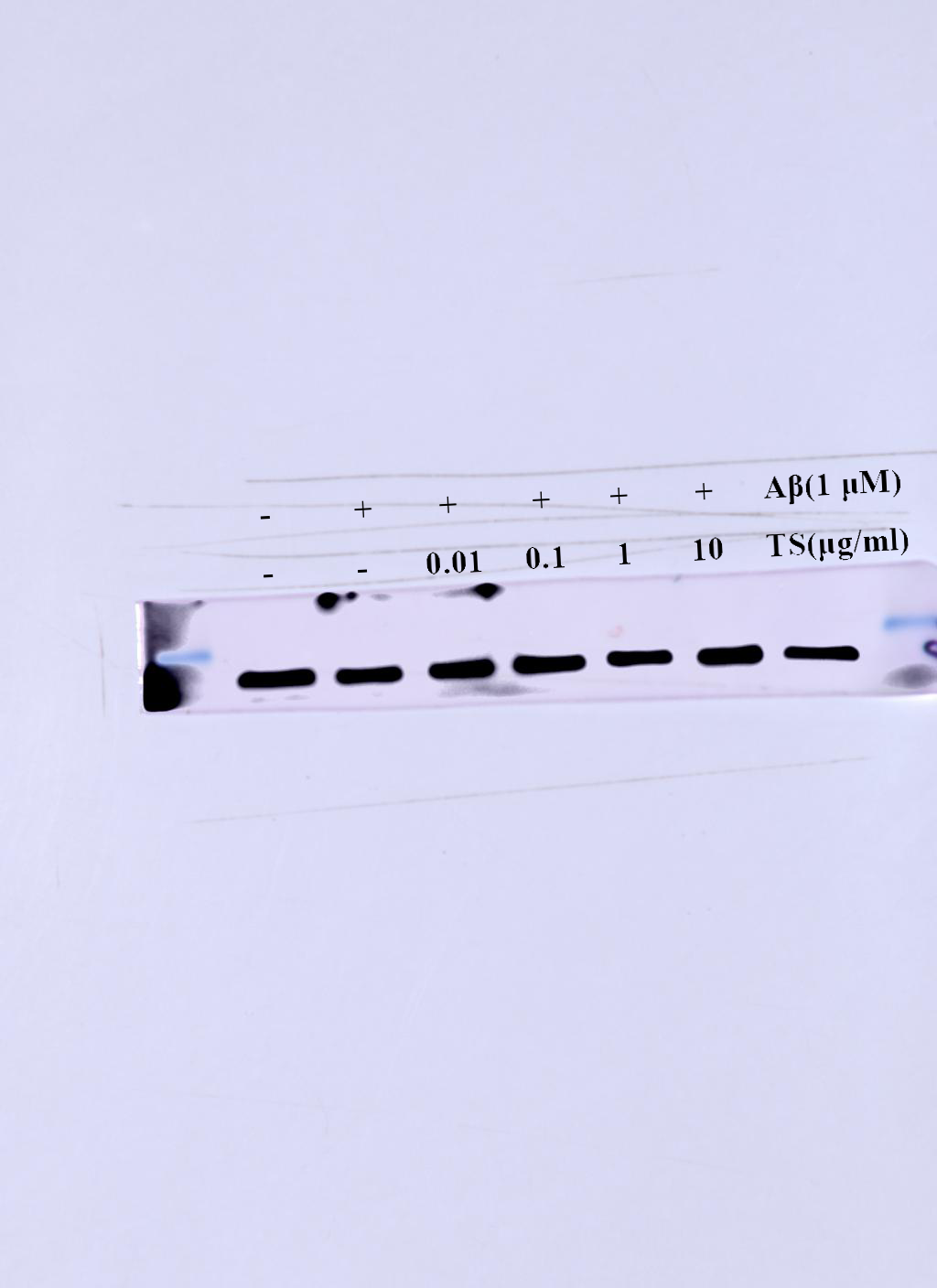

Supplement: Supplementary file 5 [file DataSheet5.ZIP › Fig.9F a┬-actin/Fig.9F orginal images for quantitative analysis-2.tif]
