# Supplementary figures and images for: Therapeutic effects of total saikosaponins from Radix bupleuri against Alzheimer’s disease (part 2 of 2)
Source: Front Pharmacol. 2022 Jul 21;13:940999. doi: 10.3389/fphar.2022.940999 (PMC9351603; doi:10.3389/fphar.2022.940999)

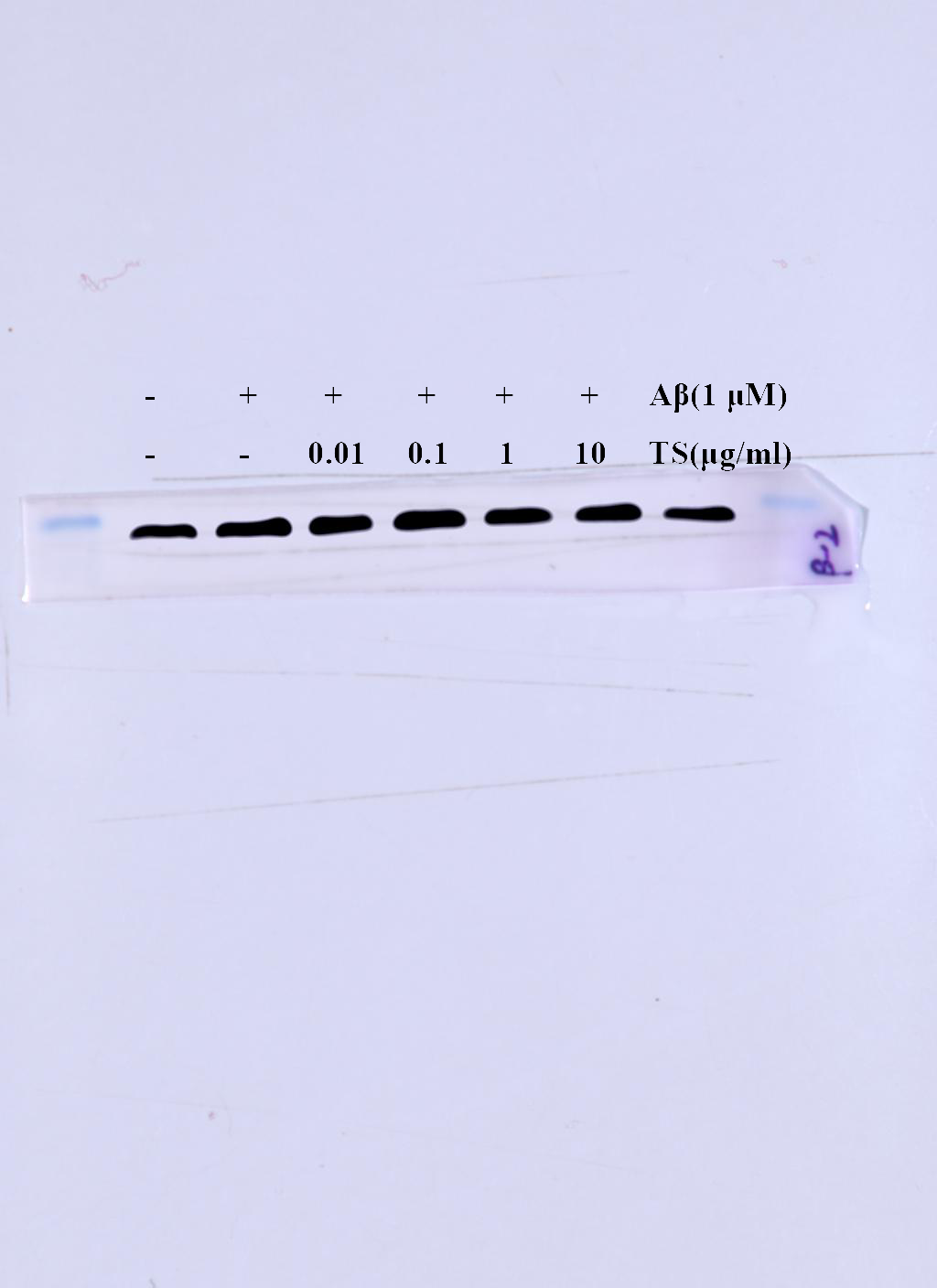

Supplement: Supplementary file 5 [file DataSheet5.ZIP › Fig.9F a┬-actin/Fig.9F orginal images.tif]
